# The draft genome of blunt snout bream (*Megalobrama amblycephala*) reveals the development of intermuscular bone and adaptation to herbivorous diet

**DOI:** 10.1093/gigascience/gix039

**Published:** 2017-05-23

**Authors:** Han Liu, Chunhai Chen, Zexia Gao, Jiumeng Min, Yongming Gu, Jianbo Jian, Xiewu Jiang, Huimin Cai, Ingo Ebersberger, Meng Xu, Xinhui Zhang, Jianwei Chen, Wei Luo, Boxiang Chen, Junhui Chen, Hong Liu, Jiang Li, Ruifang Lai, Mingzhou Bai, Jin Wei, Shaokui Yi, Huanling Wang, Xiaojuan Cao, Xiaoyun Zhou, Yuhua Zhao, Kaijian Wei, Ruibin Yang, Bingnan Liu, Shancen Zhao, Xiaodong Fang, Manfred Schartl, Xueqiao Qian, Weimin Wang

**Affiliations:** 1 College of Fisheries, Key Lab of Freshwater Animal Breeding, Ministry of Agriculture, Key Lab of Agricultural Animal Genetics, Breeding and Reproduction of Ministry of Education, Huazhong Agricultural University, Wuhan 430070, China; 2 Beijing Genomics Institute (BGI)–Shenzhen, Shenzhen 518083, China; 3 Guangdong Haid Group Co., Ltd., Guangzhou 511400, China; 4 Department for Applied Bioinformatics, Institute for Cell Biology and Neuroscience, Goethe University, Frankfurt D-60438, Germany; 5 Physiological Chemistry, University of Würzburg, Biozentrum, Am Hubland, and Comprehensive Cancer Center Mainfranken, University Clinic Würzburg, Würzburg 97070, Germany; 6 Texas A&M Institute for Advanced Study and Department of Biology, Texas A&M University, College Station, TX 77843, USA

**Keywords:** *Megalobrama amblycephala*, whole genome, herbivorous diet, intermuscular bone, transcriptome, gut microflora

## Abstract

The blunt snout bream *Megalobrama amblycephala* is the economically most important cyprinid fish species. As an herbivore, it can be grown by eco-friendly and resource-conserving aquaculture. However, the large number of intermuscular bones in the trunk musculature is adverse to fish meat processing and consumption. As a first towards optimizing this aquatic livestock, we present a 1.116-Gb draft genome of *M. amblycephala*, with 779.54 Mb anchored on 24 linkage groups. Integrating spatiotemporal transcriptome analyses, we show that intermuscular bone is formed in the more basal teleosts by intramembranous ossification and may be involved in muscle contractibility and coordinating cellular events. Comparative analysis revealed that olfactory receptor genes, especially of the beta type, underwent an extensive expansion in herbivorous cyprinids, whereas the gene for the umami receptor *T1R1* was specifically lost in *M. amblycephala*. The composition of gut microflora, which contributes to the herbivorous adaptation of *M. amblycephala*, was found to be similar to that of other herbivores. As a valuable resource for the improvement of *M. amblycephala* livestock, the draft genome sequence offers new insights into the development of intermuscular bone and herbivorous adaptation.

## Background

**Figure 1:**
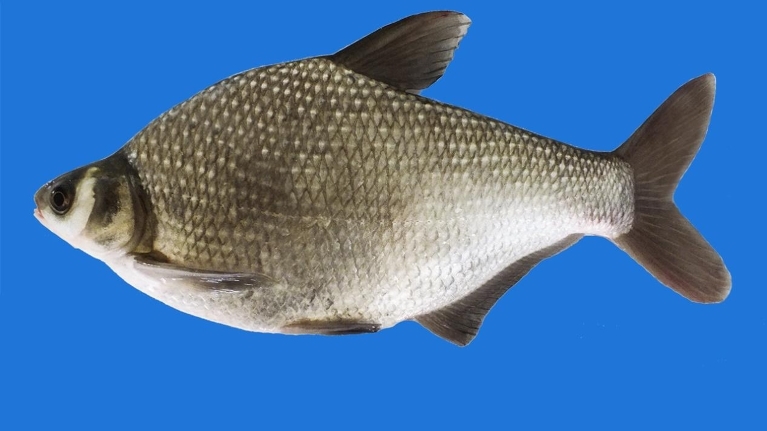
Image of an adult blunt snout bream (*Megalobrama amblycephala*).

Fishery and aquaculture play an important role in global alimentation. Over the past decades, food fish supply has been increasing, with an annual rate of 3.6%, about 2 times faster than the human population [1]. This growth of fish production is meanwhile solely accomplished by an extension of aquaculture as over the past 30 years the total mass of captured fish has remained almost constant [1]. As a consequence of this emphasis on fish breeding, the genomes of various economically important fish species, e.g., Atlantic cod (*Gadus morhua*) [2], rainbow trout (*Oncorhynchus mykiss*) [3], European sea bass (*Dicentrarchus labrax*) [4], yellow croaker (*Larimichthys crocea*) [5], half-smooth tongue sole (*Cynoglossus semilaevis*) [6], tilapia (*Oreochromis niloticus*) [7], and channel catfish (*Ictalurus punctatus*) [8], have been sequenced. Yet, the majority of these species are carnivorous, requiring large inputs of protein from wild caught fish or other precious feed. Reports on draft genomes of herbivorous and omnivorous species, in particular cyprinid fish, are scarce. It is well known that cyprinids are currently the economically most important group of teleosts for sustainable aquaculture. They grow to large population sizes in the wild and already now account for the majority of freshwater aquaculture production worldwide [1]. Among these, the herbivorous blunt snout bream, *Megalobrama amblycephala* (Fishbase Sp. ID: 285) (Fig. 1), a particularly eco-friendly and resource-conserving species, is predominant in aquaculture and has been greatly developed in China (Additional file 1: Fig. S1) [1]. However, most cyprinids, including *M. amblycephala*, have a large number of intermuscular bones (IBs) in the trunk musculature, which have an adverse effect on fish meat processing and consumption. IBs—a unique form of bone occurring only in the more basal teleosts—are completely embedded within the myosepta and are not connected to the vertebral column or any other bones [9, 10]. Our previous study on IB development of *M. amblycephala* revealed that some miRNA-mRNA interaction pairs may be involved in regulating bone development and differentiation [11]. However, the molecular genetic basis and the evolution of this unique structure are still unclear. Unfortunately, the recent sequencing of 2 cyprinid genomes, common carp (*Cyprinus carpio*) [12] and grass carp (*Ctenopharyngodon idellus*) [13], which provided valuable information for their genetic breeding, contributed little to the understanding of IB formation.

In an initial genome survey of *M. amblycephala*, we identified 25 697 single-nucleotide polymorphisms (SNPs) [14], 347 conserved miRNAs [15], and 1136 miRNA-mRNA interaction pairs [11]. However, lack of a whole-genome sequence resource limited a thorough investigation of *M. amblycephala*. Here we report the first high-quality draft genome sequence of *M. amblycephala.* Integrating this novel genome resource with tissue- and developmental stage–specific gene expression information, as well as with meta-genome data to investigate the composition of the gut microbiome (workflow shown in Additional file 1: Fig. S2), provides relevant insights into the function and evolution of 2 key features characterizing this species: The formation of IB and the adaptation to herbivory. By that our study lays the foundation for genetically optimizing *M. amblycephala* to further increase its relevance for securing human food supply.

## Data Description

### Genome assembly and annotation

The *M. amblycephala* genome was sequenced and assembled by a whole-genome shotgun strategy using genomic DNA from a double haploid fish (Additional file 1: Table S1). We assembled a 1.116-Gb reference genome sequence from 142.55 Gb (approximately 130-fold coverage) of clean data (Additional file 1: Tables S1 and S2, Fig. S3) [16]. The contig and scaffold N50 lengths reached 49 Kb and 839 Kb, respectively (Table 1). The largest scaffold spans 8951 Kb, and the 4034 largest scaffolds cover 90% of the assembly. To assess the quality of genome assembly, the short-insert size paired-end library reads and published expressed sequence tags (ESTs) [14] (Additional file 1: Tables S3 and S4) were mapped onto the genome. The results indicated that the assembled error is low. To further estimate the completeness of the assembly and gene prediction, benchmarking universal single-copy orthologs (BUSCO; RRID:SCR_015008) [17] analysis was used, and the results showed that the assembly contains 81.4% complete and 9.1% partial vertebrate BUSCO orthologs (Additional file 1: Table S5).

**Table 1:** Features of the *M. amblycephala* whole-genome sequence.

Total genome size (Mb)	1116
N90 length of scaffold (bp)	20 422
N50 length of scaffold (bp)	838 704
N50 length of contig (bp)	49 400
Total GC content (%)	37.30
Protein-coding genes number	23 696
Average gene length (bp)	15 797
Content of transposable elements (%)	34.18
Number of chromosomes	24
Number of makers in genetic map	5317
Scaffolds anchored on linkage groups (LGs)	1434
Length of scaffolds anchored on LGs (Mb)	779.54 (70%)

The *M. amblycephala* genome has an average GC content of 37.3%, similar to cyprinid *C. carpio* and *Danio rerio* (Additional file 1: Fig. S4). Using a comprehensive annotation strategy combining RNA-seq-derived transcript evidence, *de novo* gene prediction, and sequence similarity to proteins from 5 further fish species, we annotated a total of 23 696 protein-coding genes (Additional file 1: Table S6). Of the predicted genes, 99.44% (23 563 genes) are annotated by functional database. In addition, we identified 1796 non-coding RNAs including 474 miRNAs, 220 rRNA, 530 tRNAs, and 572 snRNAs. Transposable elements (TEs) comprise approximately 34.18% (381.3 Mb) of the *M. amblycephala* genome (Additional file 1: Table S7). DNA transposons (23.80%) and long terminal repeat retrotransposons (LTRs; 9.89%) are the most abundant TEs in *M. amblycephala*. The proportion of LTRs in *M. amblycephala* is the highest in comparison with other teleosts: *G. morhua* (4.88%) [2], *L. crocea* (2.2%) [5], *C. semilaevis* (0.08%) [6], *C. carpio* (2.28%) [12], *C. idellus* (2.58%) [13], and stickleback (*Gasterosteus aculeatus*; 1.9%) [18] (Additional file 1: Tables S7 and S8, Fig. S5). The distribution of divergence between the TEs in *M. amblycephala* peaks at 7% (Additional file 1: Fig. S6), indicating a more recent activity of these TEs when compared with *O. mykiss* (13%) [3] and *C. semilaevis* (9%) [6].

### Anchoring scaffolds and shared synteny analysis

**Figure 2:**
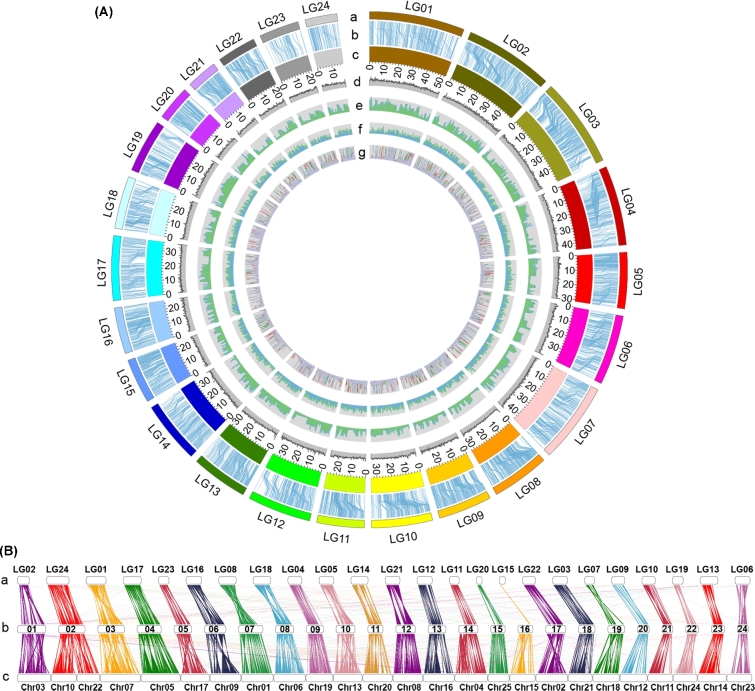
Global view of the *M. amblycephala* genome and syntenic relationship between *C. idellus*, *M. amblycephala*, and *D. rerio*. (**A**) Global view of the *M. amblycephala* genome. From outside to inside, the genetic linkage map (**a**); anchors between the genetic markers and the assembled scaffolds (**b**); assembled chromosomes (**c**); GC content within a 50-kb sliding window (**d**); repeat content within a 500-kb sliding window (**e**); gene distribution on each chromosome (**f**); and different gene expression of 3 transcriptomes (**g**). (**B**) Syntenic relationship between the *C. idellus* (**a**), *M. amblycephala* (**b**), and *D. rerio* (**c**) chromosomes.

Sequencing data from 198 F1 specimens, including the parents, were used as the mapping population to anchor the scaffolds onto 24 pseudo-chromosomes of the *M. amblycephala* genome. Following RAD-seq and sequencing protocol, 1883.5 Mb of 125-bp reads (on average 30.6 Mb and 9.3 Mb of read data for each parent and progeny, respectively) were generated on the HiSeq 2500 next-generation sequencing platform. Based on the SOAP bioinformatic pipeline (SOAPdenovo2, RRID:SCR_014986), we generated 5317 SNP markers for constructing a high-resolution genetic map. The map spans 1701 cM with a mean marker distance of 0.33 cM and facilitated an anchoring of 1434 scaffolds comprising 70% (779.54 Mb) of the *M. amblycephala* genome assembly to form 24 linkage groups (LGs) (Additional file 1: Table S9). Of the anchored scaffolds, 598 could additionally be oriented (678.27 Mb, 87.01% of the total anchored sequences) (Fig. 2A). A subsequent comparison of the gene order between *M. amblycephala* and its close relative *C. idellus* revealed 607 large shared syntenic blocks, encompassing 11 259 genes, and 190 chromosomal rearrangements. The values change to 1062 regions, 13 152 genes, and 279 rearrangements when considering *D. rerio*. The unexpected higher number of genes in syntenic regions shared with the more distantly related *D. rerio* is most likely an effect of the more complete genome assembly of this species compared to *C. idellus*. The rearrangement events are distributed across all *M. amblycephala* linkage groups without evidence for a local clustering (Fig. 2B). The most prominent event is the chromosomal fusion in *M. amblycephala* LG02 that joined 2 *D. rerio* chromosomes, Dre10 and Dre22. The same fusion is observed in *C. idellus* but not in *C. carpio*, suggesting that it probably occurred in a last common ancestor of *M. amblycephala* and *C. idellus* approximately 13.1 million years ago (Additional file 1: Fig. S7).

## Results

### Evolutionary analysis

**Figure 3:**
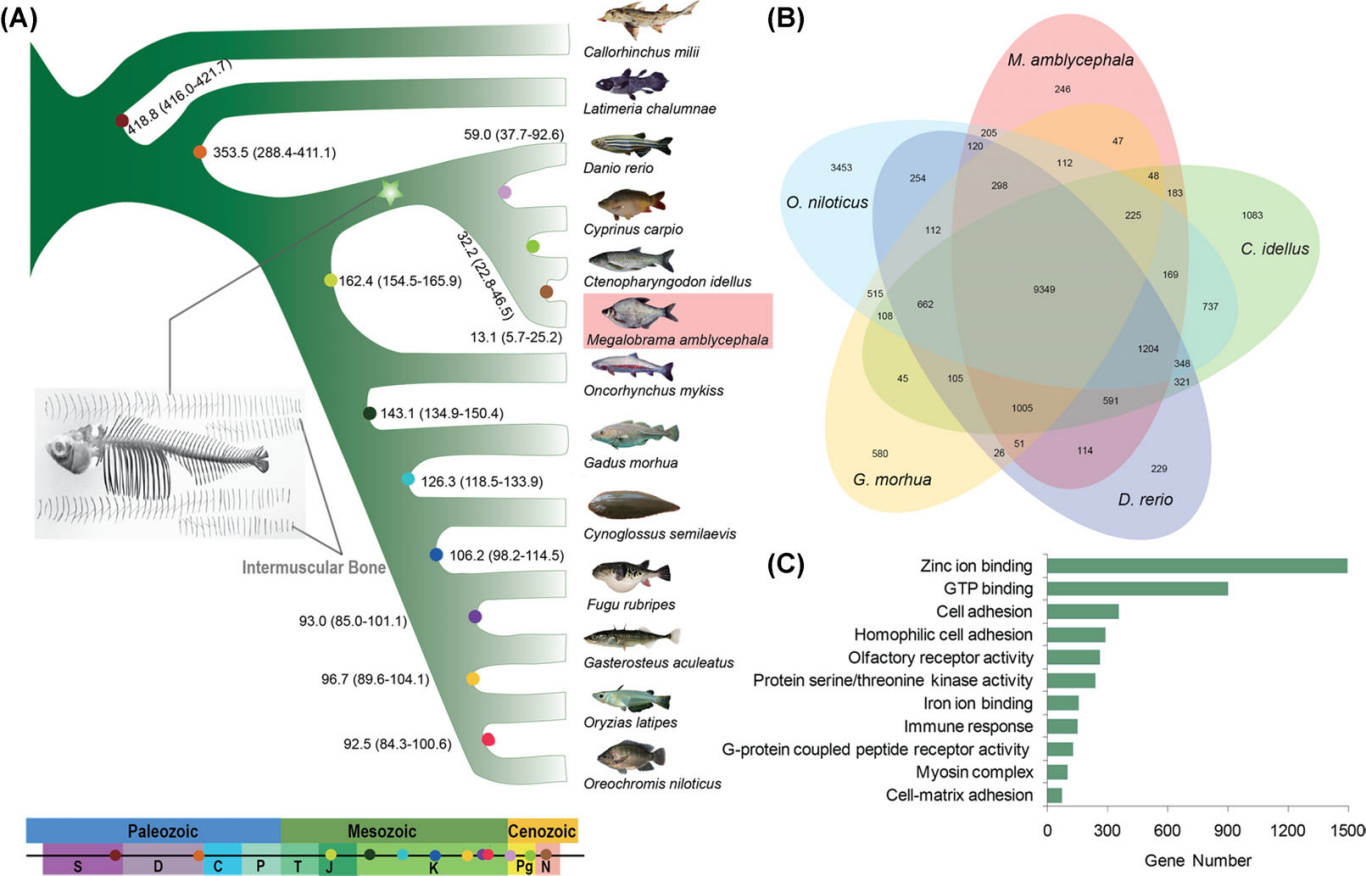
Phylogenetic tree and comparison of orthologous genes in *M. amblycephala* and other fish species. (**A**) Phylogenetic tree of teleosts using 316 single copy orthologous genes. The color circles at the nodes show the estimated divergence times using *O. latipes–F. rubripes* (96.9∼150.9 Mya), *F. rubripes–D. rerio* (149.85∼165.2 Mya), *F. rubripes–C. milii* (416∼421.75 Mya; http://www.timetree.org/) as the calibration time. The pentagram represents 4 cyprinid fish with intermuscular bones. S: Silurian period; D: Devonian period; C: Carboniferous period; P: Permian period in Paleozoic; T: Triassic period; J: Jurassic; K: cretaceous period in Mesozoic; Pg: Paleogene in Cenozoic Era; N: Neogene. (**B**) Venn diagram of shared and unique orthologous gene families in *M. amblycephala* and 4 other teleosts. (**C**) Over-represented GO annotations of cyprinid-specific expansion genes.

A phylogenetic analysis of 316 single-copy orthologous genes in the genomes of 10 other fish species, coelacanth (*Latimeria chalumnae*), and elephant shark (*Callorhinchus milii*) as outgroup served as a basis for investigating the evolutionary trajectory of *M. amblycephala* (Fig. 3A; Additional file 1: Fig. S8). We found 9349 orthologous gene families shared among 5 fish species. A total of 246 are specific to the *M. Amblycephala* (Fig. 3B). To illuminate the evolutionary process resulting in the adaptation to a grass diet, we analyzed the functional categories of expanded genes in the *M. amblycephala* and *C. idellus* lineage (Additional file 1: Fig. S9, Additional file 2: Data Note1), 2 typical herbivores mainly feeding on aquatic and terrestrial grasses. Among the significantly over-represented KEGG pathways (KEGG, RRID:SCR_012773; Fisher's exact test, *P* < 0.01), we find olfactory transduction (ko04740), immune-related pathways (ko04090, ko04672, ko04612, and ko04621), lipid metabolic-related process (ko00590, ko03320, ko00591, ko00565, ko00592, and ko04975), and xenobiotics biodegradation and metabolism (ko00625 and ko00363) (Fig. S10). Indeed, when tracing positively selected genes (PSG) in *M. amblycephala* and *C. idellus* (Additional file 3: Date Note2), we identified 10 candidates involved in starch and sucrose metabolism (ko00500), in citrate cycle (ko00020), and in other types of O-glycan biosynthesis (ko00514). Moreover, 10 genes encoding enzymes involved in lipid metabolism appear positively selected in both fish species (Additional file 1: Table S10).

### Development of intermuscular bones

**Figure 4:**
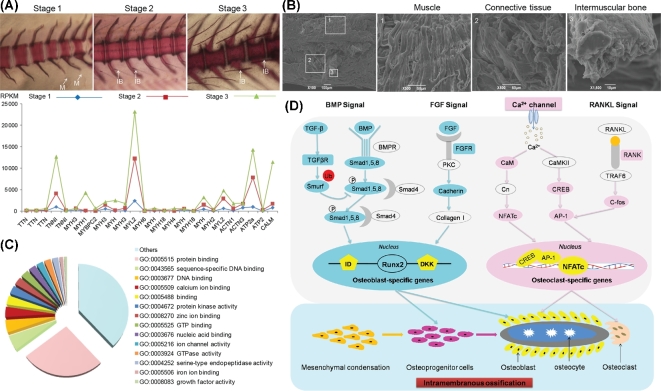
Regulation of genes related to intermuscular bone formation and function identified from developmental stages and adult tissues transcriptome data. (**A**) The gene expression pattern involved in muscle contraction–regulated genes in early developmental stages corresponds to the intermuscular bone formation of *M. amblycephala* (alizarin red staining). M: myosepta. (**B**) Scanning electron microscope photos of muscle tissues, connective tissues, and intermuscular bone. (**C**) Distribution of intermuscular bone–specific genes in GO annotations indicative of abundance in protein binding, calcium ion binding, and GTP binding functions. (**D**) Several developmental signals regulating key steps of osteoblast and osteoclast differentiation in the process of intramembranous ossification. Colored boxes indicate that significantly up-regulated genes in these signals specifically occurred in intermuscular bone.

To explain the genetic basis of IBs, their formation, and their function in cyprinids, we first analyzed the functional annotation of genes that expanded in this lineage (Fig. 3C). Many of these genes are involved in cell adhesion (GO:0 007155, *P* = 5.26E-32, 357 genes), myosin complex (GO:00 16459, *P* = 2.74E-08, 100 genes), and cell-matrix adhesion (GO:0 007160, *P* = 1.59E-21, 69 genes) (Fig. 3C). As a second line of evidence, we performed transcriptome analyses of early developmental stages (stage 1: whole larvae without IBs) and juvenile *M. amblycephala* (stage 2: trunk muscle with partial IBs; stage 3: trunk muscle with completed IBs) (Fig. 4A). Compared with stage 1, 388 and 651 differentially expressed genes (DEGs) are up-regulated in stage 2 and stage 3, respectively. And 249 of them are significantly up-regulated both in stage 2 and stage 3. KEGG analyses indicate that many of these genes are involved in tight junction (ko04530), regulation of actin cytoskeleton (ko04810), cardiac muscle contraction (ko04260), and vascular smooth muscle contraction (ko04270) (Additional file 1: Fig. S11). Specifically, 26 genes encoding proteins related to muscle contraction, including titin, troponin, myosin, actinin, calmodulin, and other Ca^2^^+^-transporting ATPases (Fig. 4A) point to a strong remodeling of the musculature compartment. To confirm that the observed differences in gene expression are indeed linked to IB formation and function and are not simply due to the fact that different developmental stages were compared, we performed differential expression analysis of muscle tissues, IBs, and connective tissues from the same 6-month-old individual of *M. amblycephala* (Fig. 4B; Additional file 1: Fig. S12); 1290 DEGs and 5231 DEGs are significantly up-regulated in IBs compared with connective tissues and muscle, respectively. Twenty-four of these DEGs encode extracellular matrix (ECM) proteins (collagens and intergrin-binding protein), Rho GTPase family (*RhoA*, *Rho GAP*, *Rac*, *Ras*), motor proteins (myosin, dynein, actin), and calcium channel regulation proteins (Additional file 1: Fig. S13 and Table S11). In addition, GO annotations of 963 IB-specific genes indicative of abundance in protein binding (GO:0 005515), calcium ion binding (GO:0 005509), GTP binding (GO:0 005525), and iron ion binding (GO:0 005506) were found (Fig. 4C).

During development of *M. amblycephala*, the first IB appears in muscles of caudal vertebrae as early as 28 days post fertilization (dpf), when body length is 12.95 mm (Additional file 1: Fig. S14). The system then develops and ossifies predominantly from posterior to anterior (Additional file 1: Fig. S15). IBs are present throughout the body within 2 months (Additional file 1: Fig. S16) and develop into multiple morphological types in adults (Additional file 1: Fig. S17). The bone is formed directly without an intermediate cartilaginous stage (Additional file 1: Figs S18 and S19). We also found a large number of mature osteoblasts distributed at the edge of the bone matrix while some osteocytes were apparent in the center of the mineralized bone matrix (Additional file 1: Figs S20 and S21). These primary bone-forming cells predominantly regulate bone formation and function throughout life. Notably, among the genes up-regulated in IB, 35 bone formation regulatory genes were identified (shown in colored boxes in Fig. 4D). In particular, genes involved in bone morphogenetic protein (BMP) signaling including *Bmp3*, *Smad8*, *Smad9*, and *Id2*, in fibroblast growth factor (FGF) signaling including *Fgf2*, *Fgfr1a*, *Fgfbp2*, *Col6a3*, and *Col4a5*, and in Ca^2+^ channels including *Cacna1c*, *CaM*, *Creb5*, and *Nfatc* were highly expressed (>2-fold change) in IB (Additional file 1: Fig. S22).

### Adaptation to herbivorous diet

**Figure 5:**
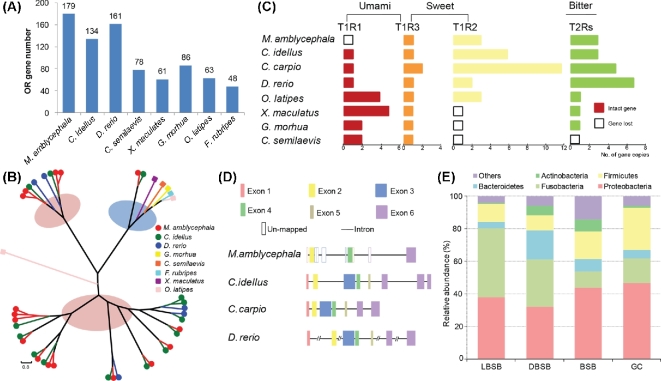
Molecular characteristics of sensory systems and the composition of gut microbiota in *M. amblycephala*. (**A**) Extensive expansion of olfactory receptor genes (ORs) in *M. amblycephala* compared with other teleosts. (**B**) Phylogeny of “beta” type ORs in 8 representative teleost species showing the significant expansion of “beta” ORs in *M. amblycephala* and *C. idellus*. The pink background shows cyprinid-specific “beta” types of ORs. (**C**) Umami, sweet, and bitter taste–related gene families in teleosts with different feeding habits. (**D**) Structure of the umami receptor encoding T1R1 gene in cyprinid fish. (**E**) Relative abundance of microbial flora and taxonomic assignments in juvenile (LBSB), domestic adult (DBSB), and wild adult (BSB) *M. amblycephala* and wild adult *C. idellus* (GC) samples at the phylum level.

Next to the presence of IB, the herbivory of *M. amblycephala* is the second key feature in connection to the use of this species as aquatic livestock. Olfaction, the sense of smell, is crucial for animals to find food. The perception of smell is mediated by a large gene family of olfactory receptor (OR) genes. In the *M. amblycephala* genome, we identified 179 functional olfactory receptor genes (Fig. 5A), and based on the classification of Niimura [19], 158, 117, and 153 receptors for water-borne odorants were identified in *M. amblycephala*, *C. idellus*, and *D. rerio*, respectively (Additional file 1: Table S12). Overall, these receptor repertoires are substantially larger than those of other and carnivorous teleosts (*G. morhua*, *C. semilaevis*, *O. latipes*, *X. maculatus*) (Additional file 1: Figs S23 and S24, Table S12). In addition, we found a massive expansion of beta-type OR genes in the genomes of the herbivorous *M. amblycephala* and *C. idellus*, while very few exist in other teleosts (Fig. 5B; Additional file 1: Table S12).

Taste is also an important factor in the development of dietary habits. Most animals can perceive 5 basic tastes, namely sourness, sweetness, bitterness, saltiness, and umami [20]. *T1R1*, the receptor gene necessary for sensing umami, has been lost in herbivorous *M. amblycephala* but is duplicated in carnivorous *G. morhua* and *C. semilaevis* and omnivorous *O. latipes* and *X. maculatus* (Figs 5C and D; Additional file 1: Figs S25–S6 and Table S13). In contrast, *T1R2*, the receptor gene for sensing sweet, has been duplicated in herbivorous *M. amblycephala* and *C. idellus* and omnivorous *C. carpio* and *D. rerio*, while it has been lost in carnivorous *G. morhua* and *C. semilaevis* (Additional file 1: Fig. S27 and Table S13). Also the *T2R* gene family, most likely important in the course switching to a diet that contains a larger fraction of bitterness-containing food, has been expanded in *M. amblycephala*, *C. idellus*, *C. carpio*, and *D. rerio* (Additional file 1: Fig. S28).

To obtain further insights into the genetic adaptation to herbivorous diet, we focused on further genes that might be associated with digestion. Genes that encode proteases (including pepsin, trypsin, cathepsin, and chymotrypsin) and amylases (including alpha-amylase and glucoamylase) were identified in the genomes of *M. amblycephala*, carnivorous *C. semilaevis and G. morhua* and omnivorous *D. rerio, O. latipes*, and *X. maculatus*, indicating that herbivorous *M. amblycephala* has a protease repertoire that is not substantially different from those of carnivorous and omnivorous fishes (Additional file 1: Table S14). We did not identify any genes encoding potentially cellulose-degrading enzymes including endoglucanase, exoglucanase, and beta-glucosidase in the genome of *M. amblycephala*, suggesting that utilization of the herbivorous diet may largely depend on the gut microbiome. To elucidate this further, we determined the composition of the gut microbial communities of juvenile, domestic, and wild adult *M. amblycephala* and wild adult *C. idellus* using bacterial 16S rRNA sequencing. A total of 549 020 filtered high-quality sequence reads from 12 samples were clustered at a similarity level of 97%. The resulting 8558 operational taxonomic units (OTUs) are dominated at phylum level by Proteobacteria, Fusobacteria, Bacteroidetes, Firmicutes, and Actinobacteria (Fig. 5E; Additional file 1: Table S15). Increasing the resolution to the genus level, the composition and relative abundance of the gut microbiota of wild adult *M. amblycephala* and *C. idellus* are still very similar (Additional file 1: Table S16), and we could identify more than 7% cellulose-degrading bacteria (Additional file 1: Table S17).

## Discussion

The evolutionary trajectory analysis of *M. amblycephala* and other teleosts revealed that *M. amblycephala* has the closest relationship to *C. idellus*. Both the species are herbivorous fish, but which endogenous and exogenous factors affected their feeding habits and how they adapted to their herbivorous diet are not known. Our results from the expanded genes and PSG in the lineage of the 2 herbivores uncovered a number of genes that are involved in glucose, lipid, and xenobiotics metabolism, which would enhance the ability of an herbivore to detoxify the secondary compounds present in grasses that are adverse or even toxic to the organism. Furthermore, the high-fiber but low-energy grass diet requires a highly effective intermediate metabolism that accelerates carbohydrate and lipid catabolism and conversion into energy to maintain physiological functions.

Olfaction and taste are also crucial for animals to find food and to distinguish whether potential food is edible or harmful [21, 22]. The ORs of teleosts are predominantly expressed in the main olfactory epithelium of the nasal cavity [21, 23] and can discriminate, like those of other vertebrates, different kinds of odor molecules. Previous studies have demonstrated that the beta type OR genes are present in both aquatic and terrestrial vertebrates, indicating that the corresponding receptors detect both water-soluble and airborne odorants [19, 21]. In the present study, the search for genes encoding OR showed that herbivorous *M. amblycephala* and *C. idellus* have a large number of beta-type ORs, while other omnivorous and carnivorous fish only have 1 or 2. This might be attributed to their particular herbivorous diet consisting not only of aquatic grasses but also the duckweed and terrestrial grasses, which they ingest from the water surface.

It is known that the receptor for umami is formed by the T1R1/T1R3 heterodimer, while T1R2/T1R3 senses sweet taste [24]. We found that the umami gene *T1R1* was lost in herbivorous *M. amblycephala* but duplicated in the carnivorous *G. morhua* and *C. Semilaevis*. The loss of the *T1R1* gene in *M. amblycephala* might exclude the expression of a functional umami taste receptor. Such situations in other organisms, e.g., the Chinese panda, have previously been related to feeding specialization [25]. Bitterness sensed by the *T2R* is particularly crucial for animals to protect them from poisonous compounds [22]. Interestingly, the bitter receptor *T2R* genes are expanded in herbivorous fish, but few or no copies were found in carnivorous fish. These results not only indicate the genetic adaptation to a herbivorous diet of *M. amblycephala*, but also provide a clear and comprehensive picture of adaptive evolutionary mechanisms of sensory systems in other fish species with different trophic specializations.

It has been reported that some insects such as *Tenebrio molitor* [26] and *Neotermes koshunensis* [27] and the mollusc *Corbicula japonica* [28] have genes encoding endogenous cellulose degradation–related enzymes. However, so far all analyzed herbivorous vertebrates lack these genes and always rely on their gut microbiome to digest food [25, 29]. In herbivorous *M. amblycephala* and *C. idellus*, we also did not find any homologs of digestive cellulase genes. Interestingly, our work on the composition of gut microbiota of the 2 fish species identifies more than 7% cellulose-degrading bacteria, suggesting that the cellulose degradation of herbivorous fish largely depends on their gut microbiome.

IB has evolved several times during teleost evolution [9, 30]. The developmental mechanisms and ossification processes forming IB are dramatically distinct from other bones such as ribs, skeleton, vertebrae, or spines. These usually develop from cartilaginous bone and are derived from the mesenchymal cell population by endochondral ossification [31, 32]. However, IBs form directly by intramembranous ossification and differentiate from osteoblasts within connective tissue, forming segmental, serially homologous ossifications in the myosepta. Although various methods of ossification of IB have been proposed, few experiments have been conducted to confirm the ossification process, and little is known about the potential role of IB in teleosts. Based on our findings of expanded genes in cyprinid lineage and evidence from the transcriptome of the developmental stages of IB formation, a number of genes were found to interact dynamically to mediate efficient cell motility, migration, and muscle construction [33–36]. In addition, transcriptome analyses of 3 tissues indicated that ECM, Rho GTPase, and motor and calcium channel regulation protein displayed high expression in IB. It is known that ECM proteins bound to integrins influence cell migration by actomyosin-generated contractile forces [34, 37]. Rho GTPases, acting as molecular switches, are also involved in regulating the actin cytoskeleton and cell migration, which in turn initiates intracellular signaling and contributes to tissue repair and regeneration [38–40]. Thus, our results provide molecular evidence that IBs might play significant roles not only in regulating muscle contraction but also in active remodeling at the bone-muscle interface and coordination of cellular events.

Some major developmental signals including BMP, FGF, and WNT, together with calcium/calmodulin signaling [31, 41–43], are essential for regulating the differentiation and function of osteoblasts and osteocytes and for regulating the RANKL signaling pathway for osteoclasts [44]. In agreement with this concept, we found that 35 bone formation regulatory genes involved in these signals were highly up-regulated in IBs. Among these signaling pathways, in particular, *Bmp*, *Fgf2*, and *Fgfr1* are closely related to intramembranous bone development and affect the expression and activity of other osteogenesis-related transcription factors [31, 45]. The calcium-sensitive transcription factor *NFATc1*, together with *CREB*, induces the expression of osteoclast-specific genes [46]. Taken together, these results suggest that IB indeed undergoes an intramembranous ossification process, is regulated by bone-specific signaling pathways, and underlies a homeostasis of maintenance, repair, and remodeling.

## Conclusions

Our results provide novel functional insights into the evolution of cyprinids. Importantly, the *M. amblycephala* genome data come up with novel insights, shedding light on the adaptation to herbivorous nutrition and the evolution and formation of IB. Our results on the evolution of gene families, as well as the digestive and sensory system, and our microbiome meta-analysis and transcriptome data provide powerful evidence and a key database for future investigations to increase the understanding of the specific characteristics of *M. amblycephala* and other fish species.

## Methods

### Sampling and DNA extraction

DNA for genome sequencing was derived from a double haploid fish from the *M. amblycephala* genetic breeding center at Huazhong Agricultural University (Wuhan, Hubei, China). Fish blood was collected from adult female fish caudal vein using sterile injectors with pre-added anticoagulant solutions following anesthetized with MS-222 and sterilization with 75% alcohol. Genomic DNA was extracted from the whole blood.

### Genomic sequencing and assembly

Libraries with different insert sizes of 170 bp, 500 bp, 800 bp, 2 Kb, 5 Kb, 10 Kb, and 20 Kb were constructed from the genomic DNA at BGI-Shenzhen. The libraries were sequenced using a HiSeq2000 instrument. In total, 11 libraries, sequenced in 23 lanes, were constructed. To obtain high-quality data, we applied filtering criteria for the raw reads. As a result, 142.55 Gb of filtered data were used to complete the genome assembly using SOAPdenovo_V2.04 (SOAPdenovo2, RRID:SCR_014986) [16]. Only filtered data were used in the genome assembly. First, the short–insert size library data were used to construct a de Bruijn graph. The tips, merged bubbles, and connections with low coverage were removed before resolving the small repeats. Second, all high-quality reads were realigned with the contig sequences. The number of shared paired-end relationships between pairs of contigs was calculated and weighted with the rate of consistent and conflicting paired ends before constructing the scaffolds in a stepwise manner from the short–insert size paired ends to the long–insert size paired ends. Third, the gaps between the constructed scaffolds were composed mainly of repeats, which were masked during scaffold construction. These gaps were closed using the paired-end information to retrieve read pairs in which 1 end mapped to a unique contig and the other was located in the gap region. Subsequently, local assembly was conducted for these collected reads. To assess the genome assembly quality, approximately 42.82 Gb of Illumina reads generated from short–insert size libraries were mapped onto the genome. Bwa0.5.9-r16 software (BWA, RRID:SCR_010910) [47] with default parameters was used to assess the mapping ratio, and SOAP coverage 2.27 was used to calculate the sequencing depth. We also assessed the accuracy of the genome assembly by Trinity (Trinity, RRID:SCR_013048) [48], including number of ESTs and new mRNA reads from early stages of embryos and multiple tissues, by aligning the scaffolds to the assembled transcriptome sequences.

After obtaining K-mers from the short–insert size (<1 Kb) reads with just 1 bp slide, frequencies of each K-mer were calculated. The K-mer frequency fits the Poisson distribution when a sufficient amount of data is present. The total genome size was deduced from these data in the following way: genome size = K–mer num/Peak_depth.

### Genome annotation

The genome was searched for repetitive elements using Tandem Repeats Finder (version 4.04) [49]. TEs were identified using homology-based approaches. The Repbase (version 16.10) [50] database of known repeats and a *de novo* repeat library generated by RepeatModeler (RepeatModeler, RRID:SCR_015027) were used. This database was mapped using the software of RepeatMasker (RepeatMasker, RRID:SCR_012954; version 3.3.0). Four types of non-coding RNAs (microRNAs, transfer RNAs, ribosomal RNAs, and small nuclear RNAs) were also annotated using tRNAscan-SE (version 1.23) and the Rfam database45 (Rfam, RRID:SCR_007891; release 9.1) [51].

For gene prediction, *de novo* gene prediction, homology-based methods, and RNA-seq data were used to perform gene prediction. For the sequence similarity–based prediction, *D. rerio, G. aculeatus, O. niloticus, O. latipes*, and *G. morhua* protein sequences were downloaded from Ensembl (Ensembl, RRID:SCR_002344; release 73) and were aligned to the *M. amblycephala* genome using TBLASTN (TBLASTN, RRID:SCR_011822). Then homologous genome sequences were aligned against the matching proteins using GeneWise [52] to define gene models. Augustus was employed to predict coding genes using appropriate parameters in *de novo* prediction. For the RNA-seq-based prediction, we mapped transcriptome reads to the genome assembly using TopHat (TopHat, RRID:SCR_013035) [53]. Then, we combined TopHat mapping results together and applied Cufflinks (Cufflinks, RRID:SCR_014597) [54] to predict transcript structures. All predicted gene structures were integrated by GLEAN [55] to obtain a consensus gene set. Gene functions were assigned to the translated protein-coding genes using the Blastp tool (BLASTP, RRID:SCR_001010), based on their highest match to proteins in the SwissProt and TrEMBL [56] databases (UniProt, RRID:SCR_002380; Uniprot release 2011–01). Motifs and domains in the protein-coding genes were determined by InterProScan (InterProScan, RRID:SCR_005829; version 4.7) searches against 6 different protein databases: ProDom, PRINTS, Pfam, SMART, PANTHER, and PROSITE. Gene ontology (GO: RRID:SCR_002811) [57] IDs for each gene were obtained from the corresponding InterPro entries. All genes were aligned against the KEGG (KEGG, RRID:SCR_012773; release 58) [58] database, and the pathway in which the gene might be involved was derived from the matched genes in KEGG. tRNA genes were *de novo* predicted by tRNAscan-SE software (tRNAscan-SE, RRID:SCR_010835) [59], with eukaryote parameters on the repeat pre-masked genome. The rRNA fragments were identified by aligning the rRNA sequences using BlastN at E-value 1e-5 (BLASTN, RRID:SCR_001598). The snRNA and miRNA were searched by the method of aligning and searching with INFERNAL (Infernal, RRID:SCR_011809; version 0.81) [60] against the Rfam database (Rfam, RRID:SCR_007891; release 9.1).

### Genetic map construction

To anchor the scaffolds into pseudo-chromosomes, 198 F1 population individuals were used to obtain the genetic map. Each of the individual genomic DNA was digested with the restriction endonuclease EcoR I, following the RAD-seq protocol [61]. The SNP calling process was carried out using the SOAP bioinformatic pipeline. The RAD-based SNP calling was done by SOAPsnp software (SOAPsnp, RRID:SCR_010602) [62] after each individual's paired-end RAD read was mapped onto the assembled reference genome with the alignment software SOAP2 (SOAPaligner/soap2, RRID:SCR_005503) [63]. The potential SNP markers were used for the linkage analysis if the following criteria were satisfied: for parents—sequencing depth ≥ 8 and ≤ 100, base quality ≥ 25, copy number ≤ 1.5; for progeny—sequencing depth ≥5, base quality ≥20, copy number ≤1.5. If the markers were showing significantly distorted segregation (*P* < 0.01), they were excluded from the map construction. Linkage analysis was performed only for markers present in at least 80% of the genomes, using JoinMap 4.0 software (JOINMAP, RRID:SCR_009248) with cross pollination (CP) population-type codes and applying the double pseudo-test cross strategy [64]. The linkage groups were formed at a logarithm of odds threshold of 6.0 and ordered using the regression mapping algorithm.

### Construction of gene families

We identified gene families using TreeFam software (Tree families database, RRID:SCR_013401) [65] as follows: Blast was used to compare all the protein sequences from 13 species: *M. amblycephala*, *C. idellus, C. semilaevis*, *C. carpio*, *D. rerio*, *Callorhinchus milii*, *G. morhua, G. aculeatus, Latimeria chalumnae*, *Oncorhynchus mykiss, O. niloticus, O. latipes*, and *Fugu rubripes*, with the E-value threshold set as 1e-7. In the next step, HSP segments of each protein pair were concatenated by Solar software. H-scores were computed based on Bit-scores, and these were taken to evaluate the similarity among genes. Finally, gene families were obtained by clustering of homologous gene sequences using Hcluster_sg (version 0.5.0). Specific genes of *M. amblycephala* were those that did not cluster with other vertebrates that were chosen for gene family construction and those that did not have homologs in the predicted gene repertoire of the compared genomes. If these genes had functional motifs, they were annotated by GO.

### Phylogenetic tree reconstruction and divergence time estimation

The coding sequences of single-copy gene families conserved among *M. amblycephala*, *C. idellus, C. carpio, D. rerio, C. semilaevis, G. morhua, G. aculeatus, Latimeria chalumnae, O. mykiss, O. niloticus, O. latipes, C. milii*, and *Fugu rubripes* (Ensembl Gene version 77) were extracted and aligned with guidance from amino-acid alignments created by the MUSCLE program (MUSCLE, RRID:SCR_011812) [66]. The individual sequence alignments were then concatenated to form 1 supermatrix. PhyML (PhyML, RRID:SCR_014629) [67, 68] was applied to construct the phylogenetic tree under an HKY85+ gamma model for nucleotide sequences. Approximate likelihood ratio test (aLRT) values were taken to assess the branch reliability in PhyML. The same set of codon sequences at position 2 was used for phylogenetic tree construction and estimation of the divergence time. The PAML mcmctree program (PAML, RRID:SCR_014932; PAML version 4.5) [69, 70] was used to determine divergence times with the approximate likelihood calculation method and the correlated molecular clock and REV substitution model.

### Gene family expansion and contraction analyses

Protein sequences of *M. amblycephala* and 11 other related species (Ensembl Gene version 77) were used in BLAST searches to identify homologs. We identified gene families using CAFÉ [71], which employs a random birth and death model to study gene gains and losses in gene families across a user-specified phylogeny. The global parameter λ, which describes both the gene birth (λ) and death (μ = −λ) rate across all branches in the tree for all gene families, was estimated using maximum likelihood. A conditional *P*-value was calculated for each gene family, and families with conditional *P-*values of less than the threshold (0.05) were considered to have a notable gain or loss. We identified branches responsible for low overall *P*-values of significant families.

### Detection of positively selected genes

We calculated Ka/Ks ratios for all single copy orthologs of *M. amblycephala* and *C. semilaevis, D. rerio, G. morhua, O. niloticus*, and *C. carpio*. Alignment quality was essential for estimating positive selection. Thus, orthologous genes were first aligned by PRANK [72], which is considerably conservative for inferring positive selection. We used Gblocks [73] to remove ambiguously aligned blocks within PRANK alignments and employed “codeml” in the PAML package with the free-ratio model to estimate Ka, Ks, and Ka/Ks ratios on different branches. The differences in mean Ka/Ks ratios for single-copy genes between *M. amblycephala* and each of the other species were compared using paired Wilcoxon rank sum tests. Genes that showed values of Ka/Ks higher than 1 along the branch leading to *M. amblycephala* were reanalyzed using the codon-based branch site tests implemented in PAML (PAML, RRID:SCR_014932). The branch site model allowed ω to vary both among sites in the protein and across branches, and it was used to detect episodic positive selection.

### Developmental process of intermuscular bone in *M. amblycephala*

To better understand the number and morphological types of IBs in adult *M. amblycephala*, specimens with a body length ranging from 15.5 to 20.5 cm were collected, and each individual was wrapped in gauze and boiled. The fish body was divided into 2 sections: anterior (snout to cloaca) and posterior (cloaca to base of caudal fin), and the length of each section was measured. The IBs were retrieved, counted, arranged in order, and photographed with a digital camera. Fertilized *M. amblycephala* eggs were brought from hatching facilities at the Freshwater Fish Genetics Breeding Center of Huazhong Agricultural University (Wuhan, Hubei, China) to our laboratory. *M. amblycephala* larvae were maintained in a re-circulating aquaculture system at 23°C ± 1°C with a 14-hour photoperiod. To explore the early development of IBs, larvae at different stages from 15 to 40 dpf were collected and fixed in 4% paraformaldehyde and transferred to 70% ethanol for storage. Specimens were stained with alizarin red for bone following the method described by Dawson [74]. The appearance of the red color was recorded as the appearance of IB because bone ossification is accompanied by the uptake of alizarin red, resulting in red staining of the mineralized bone matrix. Myosepta, either not yet ossified or poorly ossified, are not visible with alizarin red staining. For histologic analysis, specimens were paraffin-embedded and sectioned following standard protocols. Sections were stained with hematoxylin and eosin and Masson trichrome [75] and photographed using a Nikon microscope (Nikon, Tokyo, Japan) with a DP70 digital camera (Olympus, Japan). Scanning electron microscopy (SEM) and transmission electron microscopy (TEM) were also conducted to analyze the ultrastructure of IB. The specimens were fixed with 2.5% (v/v) glutaraldehyde in a solution of 0.1 M sodium cacodylate buffer (pH 7.3) for 2 hours at room temperature. The SEM and TEM samples were prepared according to a standard protocol described by Ott [76]. The samples were then visualized with a JSM-6390LV scanning electron microscope (SEM, Japan), and the stained ultrathin sections with an H-7650 transmission electron microscope (Hitachi, Japan).

### RNA sequencing analysis

*M. amblycephala* specimens belonging to 3 different developmental stages of IBs (stage 1: whole larvae without distribution of IB; stage 2: muscle tissues with partial distribution of IBs; stage 3: muscle tissues with completed distribution of IBs that were identified under microscope and immediately frozen in liquid nitrogen). In addition, dorsal white muscle, IBs, and connective tissue surrounding the IBs from 6-month-old fish were also collected. RNA was extracted from total fish samples at different stages and from individual muscle, connective tissue, and intermuscular bone samples of *M. amblycephala* using RNAisoPlus Reagent (TaKaRa, China) according to the manufacturer's protocol. The integrity and purity of the RNA was determined by gel electrophoresis and Agilent 2100 BioAnalyzer (Agilent Technologies, Palo Alto, CA, USA) before preparing the libraries for sequencing. Paired-end RNA sequencing was performed using the Illumina HiSeq 2000 platform. Low-quality score reads were filtered, and the clean data were aligned to the reference genome using Bowtie [77]. Gene and isoform expression levels were quantified by a software package: RSEM (RNASeq by Expectation Maximization; RSEM, RRID:SCR_013027) [78]. Gene expression levels were calculated by using the reads per kilobase transcriptome per million mapped reads (RPKM) method [79] and adjusted by a scaling normalization method [80]. We detected DEGs from 3 stages of IBs with software NOIseq and 3 different tissues with PossionDis as requested. NOIseq is based on a noisy distribution model, performed as described by Tarazona [81]. The parameters were set as: fold change ≥ 2.00 and probability ≥ 0.7. PossionDis is based on the Poisson distribution, performed as described by Audic [82]. The parameters were set as: fold change ≥ 2.00 and FDR ≤ 0.001. Annotation of DEGs was mapped to GO categories in the database, and the numbers of genes for every term were calculated to identify GO terms that were significantly enriched in the input list of DEGs. The calculated *P*-value was adjusted by the Bonferroni Correction, taking corrected *P*-value ≤ 0.05 as a threshold. KEGG automatic annotation was used to perform pathway enrichment analysis of DEGs.

### Prediction of olfactory receptor genes

Olfactory receptor genes were identified by previously described methods, with the exception of a first-round TBLASTN (TBLASTN, RRID:SCR_011822) [83] search, in which 1417 functional olfactory receptor genes from *H. sapiens*, *D. rerio*, *L. chalumnae*, *Lepisosteus oculatus*, *L. vexillifer*, *O. niloticus*, *O. latipes*, *F. rubripes*, and *Xenopus tropicalis* were used as queries. We then predicted the structure of sequenced genes using the blast-hit sequence with the software GeneWise [52], extending in both 3^΄^ and 5^΄^ directions along the genome sequences. The results were further confirmed by non-redundant (NR) annotation. Then the coding sequences from the start (ATG) to stop codons were extracted, while sequences that contained interrupting stop codons or frame shifts were regarded as pseudogenes. To construct phylogenetic trees, the amino acid sequences encoded by olfactory receptor genes were first aligned using the program MUSCLE nested in MEGA 5.10 (MEGA Software, RRID:SCR_000667) [84]. We then constructed the phylogenetic tree using the neighbor-joining method with Poisson correction distances using the program MEGA 5.10. We also identified and compared the genes for 5 basic tastes (sour, sweet, bitter, umami, and salty) using a similar method as in OR gene identification.

### Gut microbiota analysis

To characterize the microbial diversity of herbivorous *M. amblycephala*, 12 juvenile (LBSB), domestic adult (DBSB), and wild adult *M. amblycephala* (BSB) and wild adult *C. idellus* (GC) intestinal fecal samples were collected. Bacterial genomic DNA was extracted from the 200-mg gut content of each sample using a QIAamp DNA Stool Mini Kit (Qiagen, Valencia, USA). Quality and integrity of each DNA sample were determined by 1% agarose gel electrophoresis in Tris-acetate-EDTA buffer. DNA concentration was quantified using a NanoDrop ND-2000 spectrophotometer (Thermo Scientific, Waltham, MA, USA). To determine the diversity and composition of the bacterial communities of each sample, 20 μg of genomic DNA were sequenced using the Illumina MiSeq sequencing platform. Polymerase chain reaction amplifications were conducted from each sample to produce the V4 hypervariable region (515F and 806 R) of the 16S rRNA gene according to the previously described method [86]. We used the UPARSE pipeline [87] to pick OTUs at an identity threshold of 97% and picked representative sequences for each OTU and used the Ribosomal Database Project (RDP) classifier to assign taxonomic data to each representative sequence.

### Additional files

Additional file 1: Tables S1 to S17 and Figs S1 to S28.

Additional file 2: Data Note 1: Expanded genes in the *M. amblycephala* and *C. idellus* lineage.

Additional file 3: Data Note 2: Positively selected genes in the *M. amblycephala* and *C. idellus* genomes.

### Abbreviations

BMP: bone morphogenetic protein; BUSCO: benchmarking universal single-copy orthologs; DEGs: differentially expressed genes; dpf: days post fertilization; ECM: extracellular matrix; FGF: fibroblast growth factor; IB: intermuscular bone; LG: linkage group; LTR: long terminal repeat retrotransposon; PSG: positively selected gene; OR: olfactory receptor; SEM: scanning electron microscopy; SNP: single-nucleotide polymorphism; TEM: transmission electron microscopy; TE: transposable element.

## Supplementary Material

AReviewer-2_Attachment-(Revision-2).pdf

GIGA-D-16-00088_Original-Submission.pdf

GIGA-D-16-00088_Revision-1.pdf

GIGA-D-16-00088_Revision-2.pdf

GIGA-D-16-00088_Revision-3.pdf

Response-to-Reviewer-Comments_Original-Submission.pdf

Response-to-Reviewer-Comments_Revision-1.pdf

Response-to-Reviewer-Comments_Revision-2.pdf

Reviewer-1-Report-(Original-Submission)-.pdf

Reviewer-1-Report-(Revision-1).pdf

Reviewer-2-Report-(Original-Submission).pdf

Reviewer-2-Report-(Revision-1).pdf

Reviewer-2-Report-(Revision-2).pdf

Reviewer-2_Attachment-(Original-Submission).pdf

Additional files

## Availability of data and materials

Datasets and source images supporting the results of this article are available in the GigaDB repository associated with this publication [88]. Raw whole-genome sequencing and RAD-seq data have been deposited at NCBI in the SRA under accession number SRP090157 (BioProject Number: PRJNA343584).
