# Supplementary material for: The draft genome of blunt snout bream (Megalobrama amblycephala) reveals the development of intermuscular bone and adaptation to herbivorous diet
Source: Gigascience. 2017 May 23;6(7):1–13. doi: 10.1093/gigascience/gix039 (PMC5570040; doi:10.1093/gigascience/gix039)
Supplement: AReviewer-2_Attachment-(Revision-2).pdf [file gix039_AReviewer-2_Attachment-(Revision-2).pdf]

REVIEW R2

The draft genome of *Megalobrama amblycephala* reveals the development of intermuscular bone and adaptation to herbivorous diet by Liu H., et al.

The manuscript has been improved a lot and reads now much better. The workflow illustrated now in Additional file 1, Figure S2 looks great and indeed shows immediately the work done. Thank you for providing this.

Comments which are left to the Authors to take them into consideration or not:

Concerning Database submission:

Will this ftp site be available like the data in NCBI or will the data be taken out after a while? Besides this, I could not get access to the data. Most probably because data will be available only after acceptance? Nevertheless, there are public databases (also at NCBI or ENA) data could have been submitted to (data also available only after acceptance). Transcriptome Assembly for example could be submitted to the NCBI TSA database, the genome data to the WGS and so on.

Besides this the sentence at line 552 is not clear. NCBI SRA database accession numbers start with SRP (not provided) while the number provided PRJNA343584 is for the BioProject database.

Recommendation from NCBI: "*Currently, there is no requirement for authors to use a specific SRA accession in their publications. If there was, we would recommend that authors use* ***the SRP (study) accession****, which would provide the reader/user with a complete overview of the study and a set of links to all the data from that study."*

Minor comments:

1. Have look at the sentence at line 104-107: "to assess the genome assembly quality ….."
2. Line 151 "single –copy genes"?
3. 153: "outgroup" not "out group"
4. Rephrase line 552.
5. 3 Additional file 2 Data note 1. Typo: "Expansion" instead of "Expasion"
6. Figure 2B not mentioned in the text.
