# Supplementary material for: The draft genome of blunt snout bream (Megalobrama amblycephala) reveals the development of intermuscular bone and adaptation to herbivorous diet
Source: Gigascience. 2017 May 23;6(7):1–13. doi: 10.1093/gigascience/gix039 (PMC5570040; doi:10.1093/gigascience/gix039)
Supplement: GIGA-D-16-00088_Original-Submission.pdf [file gix039_GIGA-D-16-00088_Original-Submission.pdf]

GigaScience

The draft genome of Megalobrama amblycephala reveals the development of intermuscular bone and adaptation to herbivorous diet

--Manuscript Draft--

**Manuscript Number:**	GIGA-D-16-00088
**Full Title:**	The draft genome of Megalobrama amblycephala reveals the development of intermuscular bone and adaptation to herbivorous diet
**Article Type:**	Research
**Funding Information:**	
**Abstract:**	Background: The blunt snout bream, Megalobrama amblycephala, is the economically most important cyprinid fish species. As an herbivore, it can be grown by eco-friendly and resource-conserving aquaculture. However, the large number of intermuscular bones in the trunk musculature is adverse to fish meat processing and consumption.<br>Results: As a first towards optimizing this aquatic lifestock, we present a 1.116-Gb draft genome of M. amblycephala, with 779.54 Mb anchored on 24 linkage groups. Integrating spatiotemporal transcriptome analyses we show that intermuscular bone is formed in the more basal teleosts by intramembranous ossification, and may play a role in muscle contractibility and coordinating cellular events. Comparative analysis revealed that olfactory receptor genes, especially of the beta type, underwent an extensive expansion in herbivorous cyprinids, whereas the gene for the umami receptor T1R1 was specifically lost in M. amblycephala. The composition of gut microflora, which contributed to the herbivorous adaptation of M. amblycephala, was found to be similar to that of other herbivores.<br>Conclusions: As a valuable resource for improvement of M. amblycephala lifestock, the draft genome sequence offers new insights into the development of intermuscular bone and herbivorous adaptation.
**Corresponding Author:**	Weimin Wang<br><br>CHINA
**Corresponding Author Secondary Information:**	
**Corresponding Author's Institution:**	
**Corresponding Author's Secondary Institution:**	
**First Author:**	Han Liu
**First Author Secondary Information:**	
**Order of Authors:**	Han Liu
	Chunhai Chen
	Zexia Gao
	Jiumeng Min
	Yongming Gu
	Jianbo Jian
	Xiewu Jiang
	Huimin Cai
	Ingo Ebersberger
	Meng Xu
	Xinhui Zhang
	Jianwei Chen

Powered by Editorial Manager® and ProduXion Manager® from Aries Systems Corporation

	Wei Luo
	Boxiang Chen
	Junhui Chen
	Hong Liu
	Jiang Li
	Ruifang Lai
	Mingzhou Bai
	Jin Wei
	Shaokui Yi
	Huanling Wang
	Xiaojuan Cao
	Xiaoyun Zhou
	Yuhua Zhao
	Kaijian Wei
	Ruibin Yang
	Bingnan Liu
	Shancen Zhao
	Xiaodong Fang
	Manfred Schartl
	Xueqiao Qian
	Weimin Wang
**Order of Authors Secondary Information:**	
**Opposed Reviewers:**	Byrappa Venkatesh, Dr.<br>Prof.<br>mcbbv@imcb.a-star.edu.sg
	Yaping Wang, Dr.<br>Prof.<br>wangyp@ihb.ac.cn
	Shaojun Liu, Dr.<br>Prof.<br>lsj@hunnu.edu.cn
**Additional Information:**	
**Question**	**Response**
Are you submitting this manuscript to a special series or article collection?	No
**Experimental design and statistics**<br><br>Full details of the experimental design and statistical methods used should be given in the Methods section, as detailed in our Minimum Standards Reporting Checklist. Information essential to interpreting the data presented should be made available in the figure legends.	Yes

Powered by Editorial Manager® and ProduXion Manager® from Aries Systems Corporation

Have you included all the information requested in your manuscript?	
**Resources**<br><br>A description of all resources used, including antibodies, cell lines, animals and software tools, with enough information to allow them to be uniquely identified, should be included in the Methods section. Authors are strongly encouraged to cite Research Resource Identifiers (RRIDs) for antibodies, model organisms and tools, where possible.<br><br>Have you included the information requested as detailed in our Minimum Standards Reporting Checklist?	Yes
**Availability of data and materials**<br><br>All datasets and code on which the conclusions of the paper rely must be either included in your submission or deposited in publicly available repositories (where available and ethically appropriate), referencing such data using a unique identifier in the references and in the “Availability of Data and Materials” section of your manuscript.<br><br>Have you have met the above requirement as detailed in our Minimum Standards Reporting Checklist?	Yes

Manuscript

Click here to download Manuscript 1 2016.9.8 Manuscript of Megalobrama amblycephala.docx

Click here to view linked References

The draft genome of *Megalobrama amblycephala* reveals the development of intermuscular bone and adaptation to herbivorous diet

Han Liu[1†], Chunhai Chen[2†], Zexia Gao[1†], Jiumeng Min[2†], Yongming Gu[3†], Jianbo Jian[2†], Xiewu Jiang[3], Huimin Cai[2], Ingo Ebersberger[4], Meng Xu[2], Xinhui Zhang[1], Jianwei Chen[2], Wei Luo[1], Boxiang Chen[1,3], Junhui Chen[2], Hong Liu[1], Jiang Li[2], Ruifang Lai[1], Mingzhou Bai[2], Jin Wei[1], Shaokui Yi[1], Huanling Wang[1], Xiaojuan Cao[1], Xiaoyun Zhou[1], Yuhua Zhao[1], Kaijian Wei[1], Ruibin Yang[1], Bingnan Liu[3], Shancen Zhao[2], Xiaodong Fang[2], Manfred Schartl[5,*], Xueqiao Qian[3,*], Weimin Wang[1,*]

[*]Correspondence: wangwm@mail.hzau.edu.cn; xueqiaoqian@263.net; phch1@biozentrum.uni-wuerzburg.de

[†]Equal contributors

[1]College of Fisheries, Key Lab of Freshwater Animal Breeding, Ministry of Agriculture, Key Lab of Agricultural Animal Genetics, Breeding and Reproduction of Ministry of Education, Huazhong Agricultural University, Wuhan 430070, China

[2]Beijing Genomics Institute (BGI)-Shenzhen, Shenzhen 518083, China

[3]Guangdong Haid Group Co., Ltd., Guangzhou 511400, China

[4]Dept. for Applied Bioinformatics, Institute for Cell Biology and Neuroscience, Goethe University, Frankfurt D-60438, Germany

[5]Physiological Chemistry, University of Würzburg, Biozentrum, Am Hubland, and Comprehensive Cancer Center Mainfranken, University Clinic Würzburg, Würzburg 97070, Germany

Abstract

Background: The blunt snout bream, *Megalobrama amblycephala*, is the economically most important cyprinid fish species. As an herbivore, it can be grown by eco-friendly and resource-conserving aquaculture. However, the large number of intermuscular bones in the trunk musculature is adverse to fish meat processing and consumption.

Results: As a first towards optimizing this aquatic lifestock, we present a 1.116-Gb draft genome of *M. amblycephala*, with 779.54 Mb anchored on 24 linkage groups. Integrating spatiotemporal transcriptome analyses we show that intermuscular bone is formed in the more basal teleosts by intramembranous ossification, and may play a role in muscle contractibility and coordinating cellular events. Comparative analysis revealed that olfactory receptor genes, especially of the beta type, underwent an extensive expansion in herbivorous cyprinids, whereas the gene for the umami receptor *T1R1* was specifically lost in *M. amblycephala*. The composition of gut microflora, which contributed to the herbivorous adaptation of *M. amblycephala,* was found to be similar to that of other herbivores.

Conclusions: As a valuable resource for improvement of *M. amblycephala* lifestock, the draft genome sequence offers new insights into the development of intermuscular bone and herbivorous adaptation.

Keywords: *Megalobrama amblycephala*, whole genome, herbivorous diet, intermuscular bone, transcriptome, gut microflora

Background

Fishery and aquaculture play an important role in global alimentation. Over the past decades food fish supply has been increasing with an annual rate of 3.6 percent, about 2 times faster than the human population [1]. This growth of fish production is meanwhile solely accomplished by an extension of aquaculture, as over the past thirty years the total mass of captured fish has remained almost constant [1]. As a consequence of this emphasis on fish breeding, the genomes of various economically important fish species, e.g. cod (*Gadus morhua*) [2], rainbow trout (*Oncorhynchus mykiss*) [3], yellow croaker (*Larimichthys crocea*) [4], tilapia (*Oreochromis niloticus*) [5] and half-smooth tongue sole (*Cynoglossus semilaevis*) [6], have been sequenced. Yet, the majority of these species are carnivorous requiring large inputs of protein from wild caught fish or other precious feed. The focus of aquacultures is, however, gradually shifting towards more resource friendly herbivorous and omnivorous species, and in particular cyprinid fish. Consequently, cyprinids are currently the economically most important group of teleosts for sustainable aquaculture. They grow to large population sizes in the wild and already now account for the majority of freshwater aquaculture production worldwide [1]. Among these, the herbivorous *Megalobrama amblycephala*, a particularly eco-friendly and resource-conserving species, is predominant in aquaculture and has been greatly developed in China. However, most cyprinids, including *M. amblycephala*, have a large number of intermuscular bones (IBs) in the trunk musculature, which have an adverse effect on fish meat processing and consumption. IBs—a unique form of bone occurring only in the more basal teleosts—are completely embedded within the myosepta and are not connected to the vertebral column or any other bones [7, 8]. To date little is known about the molecular genetic basis of IB and its formation during development. Similarly the evolution of this unique structure remains obscure. Unfortunately, the recent sequencing of two cyprinid genomes common carp (*Cyprinus carpio*) [9] and grass carp (*Ctenopharyngodon idellus*) [10], which provided valuable information for their genetic breeding, contributed little to the understanding of IB formation.

In an initial genome survey of *M. amblycephala*, we identified 25,697 SNPs [11], 347 conserved miRNAs and 22 novel miRNAs [12]. However, lack of a whole genome sequence resource limited a thorough investigation of *M. amblycephala*. Here we report the first

high-quality draft genome sequence of *M. amblycephala*. Integrating this novel genome resource with tissue- and developmental stage-specific gene expression information, as well as with meta-genome data to investigate the composition of the gut microbiome provides relevant insights into the function and evolution of two key features characterizing this species: The formation of IB and the adaptation to herbivory. By that our study lays the foundation for genetically optimizing *M. amblycephala* to further increase its relevance for securing human food supply.

Data description

Genome Assembly and Annotation

The *M. amblycephala* genome was sequenced and assembled by a whole-genome shotgun strategy using genomic DNA from a double-haploid line (Additional file 1: Table S1). We assembled a 1.116 Gb reference genome sequence from 142.55 Gb (approximately 130-fold coverage) of clean data [13] (Additional file 1: Tables S1 and S2, Figure S1). The contig and scaffold N50 lengths reached 49 Kb and 839 Kb, respectively (Table 1). The largest scaffold spans 8,951 Kb and the 4,034 largest scaffolds cover 90% of the assembly (Additional file 1: Tables S3 and S4). The mapping of paired end sequence data from the short-insert size WGS libraries (Additional file 1: Table S5), as well as of published ESTs [11] (Additional file 1: Tables S6 and S7) against the genome assembly indicated that number and extent of misassemblies is low and comparable to those of other sequenced fish species (Additional file 1: Table S8).

Using a comprehensive annotation strategy combining RNA-seq derived transcript evidence, *de-novo* gene prediction and sequence similarity to proteins from five further fish species, we annotated a total of 23,696 protein-coding genes (Additional file 1: Table S9). Of the predicted genes, 99.94% (23,681 genes) are supported by transcript data and/or by the existence of homologs in other species (Additional file 1: Table S10). In addition, we identified 1,796 non-coding RNAs including 474 miRNAs, 220 rRNA, 530 tRNAs, and 572 snRNAs (Additional file 1: Table S11). Transposable elements (TEs) comprise approximately 34% (381.3 Mb) of the *M. amblycephala* genome (Additional file 1: Tables S12 and S13). DNA transposons (23.80%) and long terminal repeat retrotransposons (LTRs) (9.89%) are the most abundant TEs in *M. amblycephala*. The proportion of LTRs in *M. amblycephala* is highest in comparison with other teleosts: *G. morhua* (4.88%) [2], *C. semilaevis* (0.08%) [6] and *L. crocea* (2.2%) [4], *C. carpio*

(2.28%) [9], *C. idellus* (2.58%) [10], stickleback (*Gasterosteus aculeatus*) (1.9%) [14] (Additional file 1: Tables S13 and S14, Figures S4 and S5). Notably, the distribution of divergence between the TEs in *M. amblycephala* peaks at only 7% (Additional file 1: Figure S6), indicating a more recent activity of these TEs when compared with *O. mykiss* (13%) [3] and *C. semilaevis* (9%) [6].

Anchoring Scaffolds and Shared Synteny Analysis

We constructed a high-resolution genetic map based on 5,317 single-nucleotide polymorphism (SNP) markers extracted from 198 individuals. The map spans 1,701 cM with a mean marker distance of 0.33 cM and facilitated an anchoring of 1,434 scaffolds comprising 70% (779.54 Mb) of the *M. amblycephala* genome assembly to form 24 linkage groups (Additional file 1: Table S15). Of the anchored scaffolds, 598 could additionally be oriented (678.27 Mb, 87.01% of the total anchored sequences) (Figure 1A, Additional file 1: Table S15). A subsequent comparison of the gene order between *M. amblycephala* and its close relative *C.idellus* revealed 607 large shared syntenic blocks encompassing 11,259 genes, and 190 chromosomal rearrangements. The values change to 1,062 regions, 13,152 genes and 279 rearrangements when considering zebrafish (*Danio rerio*) (Additional file 1: Table S16). The unexpected higher number of genes in syntenic regions shared with the more distantly related *D. rerio* is most likely an effect of the more complete genome assembly of this species compared to *C. idellus*. The rearrangement events are distributed across all *M. amblycephala* linkage groups without evidence for a local clustering (Figure 1B). The most prominent event is a chromosomal fusion in *M. amblycephala* that joined two ancestral chromosomes represented by *D. rerio* chromosomes Dre10 and Dre22. The same fusion is observed in *C. idellus* but not in *C. carpio* suggesting that it probably occurred in a last common ancestor of *M. amblycephala* and *C. idellus*, approximately 13.1 million years ago (Additional file 1: Figures S7 and S8).

Results

Evolutionary Analysis

A phylogenetic analysis of 316 single-copy genes with one to one orthologs in the genomes of 10 other fish species, and coelacanth (*Latimeria chalumnae*) and elephant shark (*Callorhinchus milii*), as out group served as a basis for investigating the evolutionary trajectory of *M. amblycephala*

(Figure 2A, Additional file 1: Figures S9 and S10). To illuminate the evolutionary process resulting in the adaptation to a grass diet, we analyzed the functional properties of expanded gene families in the *M. amblycephala* and *C. idellus* lineages (Additional file 1: Figure S11), two typical herbivores mainly feeding on aquatic and terrestrial grasses. Among the significantly over-represented KEGG pathways (Fisher's exact test, $P<0.01$), we find olfactory transduction (ko04740), immune-related pathways (ko04090, ko04672, ko04612 and ko04621), lipid metabolic related process (ko00590, ko03320, ko00591, ko00565, ko00592 and ko04975), as well as xenobiotics biodegradation and metabolism (ko00625 and ko00363) (Additional file 1: Tables S17 and S18, Figure S12). This indicates that an adaptation to herbivory goes hand in hand with coping with plant secondary metabolites that are adverse or even toxic to the organism. Furthermore, the high-fiber but low-energy grass diet requires a highly effective intermediate metabolism that accelerates carbohydrate and lipid catabolism and conversion into energy to maintain physiological functions. Indeed, when tracing positively selected genes in *M. amblycephala* and *C. idellus* (Additional file 1: Table S19), we identified many candidates involved in starch and sucrose metabolism (ko00500), citrate cycle (ko00020) and other types of O-glycan biosynthesis (ko00514). Moreover, 20 genes encoding enzymes involved in lipid and carbohydrate metabolism appear positively selected in both fish species (Additional file 1: Table S20).

Development of Intermuscular Bones

To explain the genetic basis of intermuscular bones (IBs), their formation and their function in cyprinids, we first analyzed the functional annotation of gene families that expanded in this lineage (Figure 2B). Interestingly, many of these gene families are involved in cell adhesion (GO: 0007155, P=5.26E-32, 357 genes), myosin complex (GO:0016459, P=2.74E-08, 100 genes) and cell-matrix adhesion (GO:0007160, P=1.59E-21, 69 genes), which interact dynamically to mediate efficient cell motility, migration and muscle construction [15-17] (Figure 2C, Additional file 1: Tables S21 and S22).

As a second line of evidence, we performed comparative transcriptome analyses of early developmental stages (stage1: whole larvae without IBs) and juvenile *M. amblycephala* (stage2:

trunk muscle with partial IBs; stage3: trunk muscle with completed IBs) (Figure 3A). We found 249 genes significantly up-regulated in stages 2 and 3 (with IB) compared to stage 1 (no IB). Notably, many of these genes belong to KEGG pathways involved in tight junction (ko04530), regulation of actin cytoskeleton (ko04810), cardiac muscle contraction (ko04260) and vascular smooth muscle contraction (ko04270) (Additional file 1: Figure S14). These genes play important roles in cell motility and muscle contraction [16, 18, 19], which resembles the findings from the gene family expansion analysis. Specifically, some of these genes encoding proteins related to muscle contraction, including titin, troponin, myosin, actinin, calmodulin and other Ca^{2+} transporting ATPases (Figure 3A) point to a strong remodeling of the musculature compartment.

To confirm that the observed differences in gene expression are indeed linked to IB formation and function and are not simply due to the fact that different developmental stages were compared, we eventually extended the comparative transcriptome analysis to muscle tissues, IB, and connective tissues from the same six months old individual of *M. amblycephala* (Figure 3B, Additional file 1: Figures S15-17). Among the genes that are significantly up-regulated in the IB samples many encode extracellular matrix (ECM) proteins (collagens and intergrin-binding protein), Rho GTPase family (*RhoA*, *Rho GAP*, *Rac*, *Ras*), motor proteins (myosin, dynein, actin), and calcium channel regulation protein (Additional file 1: Figure S18 and Table S23). Interestingly, it has been demonstrated that ECM proteins bound to integrins influence cell migration by actomyosin-generated contractile forces [17, 20]. Rho GTPases, acting as molecular switches, also play a pivotal role in regulating the actin cytoskeleton and cell migration, which in turn initiates intracellular signaling and contributes to tissue repair and regeneration [21-23].

During development of *M. amblycephala*, the first IB appears in muscles of caudal vertebrae as early as 28 days post fertilization (dpf) when body length is 12.95 mm (Additional file 1: Figure S19). The system then develops and ossifies predominantly from posterior to anterior (Additional file 1: Figure S20). IBs are present throughout the body within two months (Additional file 1: Figure S21) and develop into multiple morphological types in adults (Additional file 1: Figure S22). The bone is formed directly without an intermediate cartilaginous stage (Additional file 1: Figures S23 and S24). We also found a large number of mature osteoblasts distributed at the edge of the bone matrix while some osteocytes were apparent in the center of the mineralized bone

matrix (Additional file 1: Figures S25 and S26). These primary bone-forming cells predominantly regulate bone formation and function throughout life. Notably, among the genes up-regulated in IB, 35 bone formation regulatory genes were identified (Figure 3D). In particular, genes involved in Bmp signaling including *Bmp3*, *Smad8*, *Smad9*, and *Id2*, in FGF signaling including *Fgf2*, *Fgfr1a*, *Fgfbp2*, *Col6a3*, and *Col4a5*, and in Ca^{2+} channels including *Cacna1c*, *CaM*, *Creb5*, and *Nfatc* were highly expressed (>2-fold change) in IB (Additional file 1: Figure S27). It has been demonstrated that *Bmp*, *Fgf2*, and *Fgfr1* play significant roles in intramembranous bone development and affect the expression and activity of other osteogenesis related transcription factors [24, 25]. The calcium-sensitive transcription factor *NFATc1* together with *CREB* induces the expression of osteoclast-specific genes [26].

Adaptation to Herbivorous Diet

Next to the presence of IB, the strict herbivory of *M. amblycephala* is the second key feature in connection to the use of this species as aquatic lifestock. Olfaction, the sense of smell, is crucial for animals to find food. The perception of smell is mediated by a large gene family of olfactory receptor (OR) genes. The ORs of teleosts are predominantly expressed in the main olfactory epithelium of the nasal cavity [27, 28] and can discriminate, like those of other vertebrates, different kinds of odor molecules. However, compared to mammals, e.g. humans having around 400 ORs [29] the OR repertoires in teleosts are considerably small. They range from only about 48 in *Fugu rubripes* up to 161 in *D. rerio* (Figure 4A). In the *M. amblycephala* genome, we identified 179 functional olfactory receptor (OR) genes (Figure 4A), and based on the classification of Niimura [30], 158, 117 and 153 receptors for water-borne odorants were identified in *M. amblycephala*, *C. idellus* and *D. rerio*, respectively (Additional file 1: Table S24). Overall, these receptor repertoires are substantially larger than those of other and carnivorous teleosts (*G. morhua*, *C. semilaevis*, *O. latipes*, *X. maculatus*) (Additional file 1: Figures S28 and S29, Table S24). This suggests that olfaction—probably for food choice—plays a particularly important role in the cyprinid species. Previous studies have demonstrated that the beta type OR genes are present in both aquatic and terrestrial vertebrates, indicating that the corresponding receptors detect both water-soluble and airborne odorants [28, 30]. Intriguingly, we found a

massive expansion of beta-type OR genes in the genomes of the herbivorous *M. amblycephala* and *C. idellus*, while very few exist in other teleosts (Figure 4B, Additional file 1: Tables S24 and S25).

Taste is also an important factor in the development of dietary habits. Most animals can perceive five basic tastes, namely sourness, sweetness, bitterness, saltiness and umami [31]. Interestingly, *T1R1*, the receptor gene necessary for sensing umami, has been lost in herbivorous *M. amblycephala* but is duplicated in carnivorous *G. morhua*, *C. semilaevis* and omnivorous *O. latipes* and *X. maculatus* (Figures 4C and 4D, Additional file 1: Figures S30-32 and Table S26). In contrast, *T1R2*, the receptor gene for sensing sweet, has been duplicated in herbivorous *M. amblycephala* and *C. idellus*, omnivorous *C. carpio* and *D. rerio*, while it has been lost in carnivorous *G. morhua* and *C. semilaevis* (Additional file 1: Figure S33 and Table S26). Bitterness sensed by the *T2R* is particularly crucial for animals to protect them from poisonous compounds [32]. Probably in the course switching to a diet that contains a larger fraction of bittern containing food, also the *T2R* gene family in *M. amblycephala*, *C. idellus*, *C. carpio* and *D. rerio* has been expanded (Additional file 1: Figure S34).

To obtain further insights into the genetic adaptation to herbivorous diet, we focused on further genes that might be associated with digestion. Genes that encode proteases (including pepsin, trypsin, cathepsin and chymotrypsin) and amylases (including alpha-amylase and glucoamylase) were identified in the genomes of *M. amblycephala*, carnivorous *C. semilaevis, G. morhua* and omnivorous *D. rerio*, *O. latipes* and *X. maculatus*, indicating that herbivorous *M. amblycephala* has a protease repertoire that is not substantially different from those of carnivorous and omnivorous fishes (Additional file 1: Table S27). We did not identify any genes encoding potentially cellulose-degrading enzymes including endoglucanase, exoglucanase and beta-glucosidase in the genome of *M. amblycephala*, suggesting that utilization of the herbivorous diet may largely depend on the gut microbiome. To elucidate this further, we determined the composition of the gut microbial communities of juvenile, domestic, wild adult *M. amblycephala* and wild adult *C. idellus* using bacterial 16S rRNA sequencing (Additional file 1: Figure S35). A total of 549,020 filtered high quality sequence reads from 12 samples were clustered at a similarity level of 97%. The resulting 8,558 operational taxonomic units (OTUs) are dominated at phylum

level by Proteobacteria, Fusobacteria, Bacteroidetes, Firmicutes and Actinobacteria (Figure 4E, Additional file 1: Table S28). Increasing the resolution to the genus level, the composition and relative abundance of the gut microbiota of wild adult *M. amblycephala* and *C. idellus* are still very similar (Additional file 1: Table S29) and we could identify more than 7% cellulose-degrading bacteria (Additional file 1: Table S30). This indicates that indeed the gut microbiome plays a prominent role in the digestion of plant material, and thus in the adaptation to herbivory.

Discussion

M. amblycephala is the economically most important species for freshwater aquaculture. In addition to its various superior properties, expecially its herbivorous diet, it is also an excellent model to study intermuscular bones (IB) formation. Here we make available draft genome of *M. amblycephala* with more than 70% of genome data anchored on 24 linkage groups. Comparative analyses of genome structure revealed high synteny with three other cyprinid fish and uncovered a chromosomal fusion event in *M. amblycephala* that joined two ancestral chromosomes (Figure 1B), which supports the previous results in *C. idellus* [10] and also provides novel scientific insights into the evolution of chromosome fusion events in cyprinids.

The evolutionary trajectory analysis of *M. amblycephala* and other teleosts revealed that *M. amblycephala* has the closest relationship to *C. idellus* (Figure 2A). Both the species are herbivorous fish but which endogenous and exogenous factors affected their feeding habits and how they adapted to their herbivorous diet is not known. Olfaction and taste are crucial for animals to find food and to distinguish whether potential food is edible or harmful [28, 32]. The search for genes encoding OR showed that herbivorous *M. amblycephala* and *C. idellus* have a large number of beta-type OR, while other omnivorous and carnivorous fish only have one or two. This might be attributed to their particular herbivorous diet consisting not only of aquatic grasses but also the duckweed and terrestrial grasses, which they ingest from the water surface. Previous studies have demonstrated that the receptor for umami is formed by the T1R1/T1R3 heterodimer, while T1R2/T1R3 senses sweet taste [33]. We found that the umami gene *T1R1* was lost in herbivorous *M. amblycephala* but duplicated in the carnivorous *G. morhua* and *C. semilaevis*

(Figure 4C). The loss of the *T1R1* gene in *M. amblycephala* excludes the expression of a functional umami taste receptor. Such situations in other organism, e.g. the Chinese panda, have previously been related to feeding specialization [13, 34]. Interestingly, the sweetness receptor *T1R2* and bitter receptor *T2R* genes are expanded in the herbivorous fish but few or no copy was found in carnivorous fish. Collectively, these results not only indicate the genetic adaptation to herbivorous diet of *M. amblycephala*, but also provided a clear and comprehensive picture of adaptive evolutionary mechanisms of sensory systems in other fish species with different trophic specializations.

Some insects such as *Tenebrio molitor* [35] and *Neotermes koshunensis* [36], and the mollusc *Corbicula japonica* [37] have genes encoding endogenous cellulose degradation-related enzymes. However, all so far analyzed herbivorous vertebrates lack these genes and always rely on their gut microbiome to digest food [13, 38]. In herbivorous *M. amblycephala* and *C. idellus*, we also did not find any homologues of digestive cellulase genes. Interestingly, our work on the composition of gut microbiota of the two fish species identifies more than 7% cellulose-degrading bacteria, suggesting that the cellulose degradation of herbivorous fish largely depend on their gut microbiome.

Intermuscular bones (IBs) have evolved several times during teleost evolution [7, 39]. The developmental mechanisms and ossification processes forming IBs are dramatically distinct from other bones such as ribs, skeleton, vertebrae or spines. These usually develop from cartilaginous bone and are derived from the mesenchymal cell population by endochondral ossification [24, 40]. However, IBs form directly by intramembranous ossification and differentiate from osteoblasts within connective tissue, forming segmental, serially homologous ossifications in the myosepta. Although various methods of ossification of IB have been proposed, few experiments have been conducted to confirm the ossification process and little is known about the potential role of IB in teleosts. Based on our findings of over-represented functional properties of expanded gene families in cyprinid lineage (Figure 2C) and evidence from the comparative transcriptome analyses of early developmental stages of IB formation (Figure 3A), we provide molecular evidence that IB might play significant roles not only in regulating muscle contraction but also in active remodeling at the bone-muscle interface and coordination of cellular events.

It has previously been found that some major developmental signals including bone morphogenetic proteins (BMPs), fibroblast growth factors (FGFs), and WNT, together with calcium/calmodulin signaling [24, 41-43], are essential for regulating the differentiation and function of osteoblasts and osteocytes and for regulating the RANKL signaling pathway for osteoclasts [44] in intramembranous bone development. In agreement with this concept, our comparative transcriptome analysis of muscle, IB and connective tissues uncovered that 35 bone formation regulatory genes involved in these signals were highly up-regulated in IB. Taken together, these results suggest that IB indeed undergoes an intramembranous ossification process, is regulated by bone-specific signaling pathways, and underlies a homeostasis of maintenance, repair and remodeling.

Conclusions

Our results provide novel functional insights into the evolution of cyprinids. Importantly, the *M. amblycephala* genome data come up with novel insights shedding light on the adaptation to herbivorous nutrition and evolution and formation of IB. Our results on the evolution of gene families, digestive and sensory system, as well as our microbiome meta-analysis and transcriptome data provide powerful evidence and a key database for future investigations to increase the understanding of the specific characteristics of *M. amblycephala* and other fish species.

Methods

Sampling and DNA Extraction

DNA for genome sequencing was derived from a double haploid line from the *M. amblycephala* genetic breeding center at Huazhong Agricultural University (Wuhan, Hubei, China). Fish blood was collected from adult female fish caudal vein using sterile injectors with pre-added anticoagulant solutions following anesthetized with MS-222 and sterilization with 75% alcohol. Genomic DNA was extracted from the whole blood. All experimental procedures involving fish were performed in accordance with the guidelines and regulations of the National Institute of Health Guide for the Care and Use of Laboratory Animals. The experiments were also approved by the Animal Care and Use Committee of Huazhong Agricultural University.

Genomic Sequencing and Assembly

Libraries with different insert sized inserts of 170 bp, 500 bp, 800 bp, 2 Kb, 5 Kb, 10 Kb and 20 Kb were constructed from the genomic DNA at BGI-Shenzhen. The libraries were sequenced using a HiSeq2000 instrument. In total, 11 libraries, sequenced in 23 lanes were constructed. To obtain high quality data, we applied filtering criteria for the raw reads. As a result, 142.55 Gb (129.59X) of filtered data were used to complete the genome assembly using SOAPdenovo-1.05 [13]. Only filtered data were used in the genome assembly. First, the short insert size library data were used to construct a de Bruijn graph. The tips, merged bubbles and connections with low coverage were removed before resolving the small repeats. Second, all high-quality reads were realigned with the contig sequences. The number of shared paired-end relationships between pairs of contigs was calculated and weighted with the rate of consistent and conflicting paired ends before constructing the scaffolds in a stepwise manner from the short–insert size paired ends to the long–insert size paired ends. Third, the gaps between the constructed scaffolds were composed mainly of repeats, which were masked during scaffold construction. These gaps were closed using the paired-end information to retrieve read pairs in which one end mapped to a unique contig and the other was located in the gap region. Subsequently, local assembly was conducted for these collected reads.

Genome Annotation

The genome was searched for repetitive elements using Tandem Repeats Finder (version 4.04) [45]. TEs were identified using homology-based approaches. The Repbase (version 16.10) [46] database of known repeats and a *de novo* repeat library generated by RepeatModeler were used. This database was mapped using the software of RepeatMasker (version 3.3.0). Four types of non-coding RNAs (microRNAs, transfer RNAs, ribosomal RNAs and small nuclear RNAs) were also annotated using tRNAscan-SE (version 1.23) and the Rfam database45 (Release 9.1) [47]. For gene prediction, *de novo* gene prediction, homology-based methods and RNA-seq data were used to perform gene prediction. For the sequence similarity based prediction, *D. rerio, G. aculeatus, O. niloticus, O. latipes* and *G. morhua* protein sequences were downloaded from Ensembl (release 73) and were aligned to the *M. amblycephala* genome using TBLASTN. Then homologous genome sequences were aligned against the matching proteins using GeneWise [48]

to define gene models. Augustus was employed to predict coding genes using appropriate parameters in *de novo* prediction. For the RNA-seq based prediction, we mapped transcriptome reads to the genome assembly using TopHat [49]. Then, we combined TopHat mapping results together and applied Cufflinks [50] to predict transcript structures. All predicted gene structures were integrated by GLEAN [51] (http://sourceforge.net/projects/glean-gene/) to obtain a consensus gene set.

Phylogenetic Tree Reconstruction and Divergence Time Estimation

The coding sequences of single-copy gene families conserved among *M. amblycephala*, *C. idellus, C. carpio, D. rerio, C. semilaevis, G. morhua, G. aculeatus, Latimeria chalumnae, O. mykiss, O. niloticus, O. latipes*, *C. milii* and *Fugu rubripes* (Ensembl Gene v.77) were extracted and aligned with guidance from amino-acid alignments created by the MUSCLE program [52]. The individual sequence alignments were then concatenated to form one supermatrix. PhyML [53, 54] was applied to construct the phylogenetic tree under an HKY85+gamma model for nucleotide sequences. ALRT values were taken to assess the branch reliability in PhyML. The same set of codon sequences at position 2 was used for phylogenetic tree construction and estimation of the divergence time. The PAML mcmctree program (PAML version 4.5) [55, 56] was used to determine divergence times with the approximate likelihood calculation method and the 'correlated molecular clock' and 'REV' substitution model.

Gene Family Expansion and Contraction Analysis

Protein sequences of *M. amblycephala* and 11 other related species (Ensembl Gene v.77)) were used in BLAST searches to identify homologs. We identified gene families using CAFÉ [57], which employs a random birth and death model to study gene gains and losses in gene families across a user-specified phylogeny. The global parameter λ, which describes both the gene birth (λ) and death ($\mu = -\lambda$) rate across all branches in the tree for all gene families, was estimated using maximum likelihood [53]. A conditional *P*-value was calculated for each gene family, and families with conditional *P*-values less than the threshold (0.05) were considered as having notable gain or loss. We identified branches responsible for low overall *P*-values of significant families.

Detection of Positively Selected Genes

We calculated Ka/Ks ratios for all single copy orthologs of *M. amblycephala* and *C. semilaevis, D.*

rerio, G. morhua, O. niloticus and C. carpio. Alignment quality was essential for estimating positive selection. Thus, orthologous genes were first aligned by PRANK [58], which is considerably conservative for inferring positive selection. We used Gblocks [59] to remove ambiguously aligned blocks within PRANK alignments and employed 'codeml' in the PAML package with the free-ratio model to estimate Ka, Ks, and Ka/Ks ratios on different branches. The differences in mean Ka/Ks ratios for single-copy genes between *M. amblycephala* and each of the other species were compared using paired Wilcoxon rank sum tests. Genes that showed values of Ka/Ks higher than 1 along the branch leading to *M. amblycephala* were reanalyzed using the codon based branch-site tests implemented in PAML. The branch-site model allowed ω to vary both among sites in the protein and across branches, and was used to detect episodic positive selection.

Prediction of Olfactory Receptor Genes

Olfactory receptor genes were identified by previously described methods [60], with the exception of a first-round TBLASTN [61] search, in which 1,417 functional olfactory receptor genes from *H. sapiens*, *D. rerio*, *L. chalumnae*, *Lepisosteus oculatus*, *L. vexillifer*, *O. niloticus*, *O. latipes*, *F. rubripes* and *Xenopus tropicalis* were used as queries. We then predicted the structure of sequenced genes using the blast-hit sequence with the software GeneWise extending in both 3' and 5' directions along the genome sequences. To construct phylogenetic trees, the amino-acid sequences encoded by olfactory receptor genes were first aligned using the program MUSCLE nested in MEGA 5.10 [62]. We then constructed the phylogenetic tree using the neighbor-joining method [63] with Poisson correction distances using the program MEGA 5.10.

RNA Sequencing Analysis

RNA was extracted from total fish samples at different stages and from individual muscle, connective tissue, and intermuscular bone samples of adult *M. amblycephala*. Paired-end RNA sequencing was performed using the Illumina HiSeq 2000 platform. Low quality score reads were filtered and the clean data were aligned to the reference genome using Bowtie [64]. Genes and isoforms expression level were quantified by a software package: RSEM (RNASeq by Expectation Maximization) [65]. Gene expression levels were calculated by using the RPKM method (Reads per kilobase transcriptome per million mapped reads) [66] and adjusted by a

scaling normalization method [67]. Differentially expressed genes (DEGs) were detected using DESeq [68]. Annotation of DEGs were mapped to GO categories in the database (http://www.geneontology.org/) and the number of genes for every term were calculated to identify GO terms that were significantly enriched in the input list of DEGs. The calculated *P*-value was adjusted by the Bonferroni Correction, taking corrected *P*-value ≤ 0.05 as a threshold. KEGG [69] automatic annotation was used to perform pathway enrichment analysis of DEGs.

Additional file

Additional file 1: Supplementary Material contains Supplementary Figs. S1–S35, Supplementary Tables S1–S30, Supplementary Note, and Supplementary References.

Competing interests

The authors declare that they have no competing interests.

Authors' Contributions

W.W. initiated and conceived the project and provided scientific input. X.Q. organized financial support and designed the project. M.S. discussed the data, wrote and modified the paper. H.L. and C.C. conducted the biological experiments, analyzed the data and wrote the paper with input from other authors. I.E. wrote and modified the manuscript and discussed the data. The RAD sequence data analyses and the genetic map construction were performed by Z.G., Y.G., J.J. and X.J. Genome assembly and annotation were performed by J.M., H.C., M.X. and J.C. X.Z., W.L., R.L., B.C., J.W., H.L., S.Y., H.W., X.C., X.Z., Y.Z., K.W., R.Y. and B. L. carried out the samples preparation and data collection. J.L. and J.C. identified the gene families and analyzed the RNA-seq data. M.B. coordinated the project. S.Z. and X.F. modified the manuscript and discussed the data. All authors read the manuscript and provided comments and suggestions for improvements. The authors declare no competing financial interests.

Acknowledgements

This work was supported by the Modern Agriculture Industry Technology System Construction Projects of China titled as—Staple Freshwater Fishes Industry Technology System (No. CARS-46-05), Guangdong Haid Group Co., Ltd, the Fundament Research Funds for the Central Universities (2662015PY019), the International Scientific and Technology Cooperation Program

of Wuhan City (2015030809020365).

Data access

The *M. amblycephala* genome sequence and related data have been deposited at the *M. amblycephala* genome database and can be downloaded from http://bream.hzau.edu.cn/page/species/download.html.

References

1. FAO Fisheries and Aquaculture Department. The State of World Fisheries and Aquaculture 2012 (Food and Agriculture Organization of the United Nations, Rome, 2014).

2. Star B, Nederbragt AJ, Jentoft S, Grimholt U, Malmstrøm M, Gregers TF, et al. The genome sequence of Atlantic cod reveals a unique immune system. Nature. 2011; 477:207–10.

3. Berthelot C, Brunet F, Chalopin D, Juanchich A, Bernard M, Noël B, et al. The rainbow trout genome provides novel insights into evolution after whole-genome duplication in vertebrates. Nat. Commun. 2014; 5, 3657.

4. Wu C, Zhang D, Kan M, Lv Z, Zhu A, Su Y, et al. The draft genome of the large yellow croaker reveals well-developed innate immunity. Nat. Commun. 2014; 5, 5227.

5. Brawand D, Wagner CE, Li YI, Malinsky M, Keller I, Fan S, et al. The genomic substrate for adaptive radiation in African cichlid fish. Nature. 2014; 513:375–82.

6. Chen S, Zhang G, Shao C, Huang Q, Liu G, Zhang P, et al. Whole-genome sequence of a flatfish provides insights into ZW sex chromosome evolution and adaptation to a benthic lifestyle. Nat. Genet. 2014; 46:253–60.

7. Gemballa S, Britz R. Homology of intermuscular bones in acanthomorph fishes. Am. Mus. Novit. 1998; 3241:1–25.

8. Danos N, Ward AB. The homology and origins of intermuscular bones in fishes: Phylogenetic or biomechanical determinants? Biol. J. Linn. Soc. 2012; 106:607–22.

9. Xu P, Zhang X, Wang X, Li J, Liu G, Kuang Y, et al. Genome sequence and genetic diversity of the common carp, *Cyprinus carpio*. Nat. Genet. 2014; 46:1212–9.

10. Wang Y, Lu Y, Zhang Y, Ning Z, Li Y, Zhao Q, et al. The draft genome of the grass carp (*Ctenopharyngodon idellus*) provides insights into its evolution and vegetarian adaptation. Nat. Genet. 2015; 47:625–31.

11. Gao Z, Luo W, Liu H, Zeng C, Liu X, Yi S, et al. Transcriptome analysis and SSR/SNP markers information of the blunt snout bream (*Megalobrama amblycephala*). PLoS One. 2012; 7, e42637.

12. Yi S, Gao ZX, Zhao H, Zeng C, Luo W, Chen B, et al. Identification and characterization of microRNAs involved in growth of blunt snout bream (*Megalobrama amblycephala*) by Solexa sequencing. BMC Genomics. 2013; 14, 754.

13. Li R, Fan W, Tian G, Zhu H, He L, Cai J, et al. The sequence and *de novo* assembly of the giant panda genome. Nature. 2010; 463:311–7.

14. Jones FC, Grabherr MG, Chan YF, Russell P, Mauceli E, Johnson J, et al. The genomic basis of adaptive evolution in threespine sticklebacks. Nature. 2012; 484:55–61.

15. Etienne-Manneville S. Actin and microtubules in cell motility: Which one is in control? Traffic. 2004; 5:470–7.

16. Even-Ram S, Doyle AD, Conti MA, Matsumoto K, Adelstein RS, Yamada KM. Myosin IIA regulates cell motility and actomyosin-microtubule crosstalk. Nat. Cell Biol. 2007; 9:299–309.

17. Sheetz MP, Felsenfeld DP, Galbraith CG. Cell migration : Regulation of force on extracellular- complexes. Trends Cell Biol. 1998; 8:51–4.

18. Gunst SJ, Zhang W. Actin cytoskeletal dynamics in smooth muscle: a new paradigm for the regulation of smooth muscle contraction. Am. J. Physiol. Cell Physiol. 2008; 295:C576–87.

19. Webb RC. Smooth muscle contraction and relaxation. Adv. Physiol. Educ. 2003; 27:201–6.

20. Ulrich TA, De Juan Pardo EM, Kumar S. The mechanical rigidity of the extracellular matrix regulates the structure, motility, and proliferation of glioma cells. Cancer Res. 2009; 69:4167–74.

21. Ridley AJ. Rho GTPases and cell migration. J. Cell Sci. 2001; 114:2713–22.

22. Etienne-Manneville S, Hall A. Rho GTPases in Cell Biology. Nature. 2002; 420:629–35.

23. Ridley AJ. Cell Migration: Integrating Signals from Front to Back. Science. 2003; 302:1704–9.

24. Ornitz D, Marie P. FGF signaling pathways in endochondral and intramembranous bone development and human genetic disease. Genes Dev. 2002; 16:1446–65.

25. Fakhry A, Ratisoontorn C, Vedhachalam C, Salhab I, Koyama E, Leboy P, et al. Effects of FGF-2/-9 in calvarial bone cell cultures: Differentiation stage-dependent mitogenic effect, inverse regulation of BMP-2 and noggin, and enhancement of osteogenic potential. Bone. 2005; 36:254–66.

26. Sato K, Suematsu A, Nakashima T, Takemoto-Kimura S, Aoki K, Morishita Y, et al. Regulation of osteoclast differentiation and function by the CaMK-CREB pathway. Nat. Med. 2006; 12:1410–6.

27. Niimura Y, Nei M. Evolutionary dynamics of olfactory and other chemosensory receptor genes in vertebrates. J. Hum. Genet. 2006; 51:505–17.

28. Nei M, Niimura Y, Nozawa M. The evolution of animal chemosensory receptor gene repertoires: roles of chance and necessity. Nat. Rev. Genet. 2008; 9:951–63.

29. Lopez C, Raper J. Cloning and functional characterization of odorant receptors expressed in the zebrafish olfactory system. FASEB J. 2015; 29:727–37.

30. Niimura Y. On the origin and evolution of vertebrate olfactory receptor genes: comparative genome analysis among 23 chordate species. Genome Biol. Evol. 2009; 1:34–44.

31. Lindemann B. Receptors and transduction in taste. Nature. 2001; 413:219–25.

32. Chandrashekar J, Mueller KL, Hoon MA, Adler E, Feng L, Guo W, et al. T2Rs function as bitter taste receptors. Cell. 2000; 100:703–11.

33. Nelson G, Chandrashekar J, Hoon MA, Feng L, Zhao G, Ryba NJ, et al. An amino-acid taste receptor. Nature. 2002; 416:199–202.

34. Jiang P, Josue J, Li X, Glaser D, Li W, Brand JG, et al. From the Cover: Major taste loss in carnivorous mammals. Proc. Natl. Acad. Sci. USA. 2012; 109:4956–61.

35. Ferreira AHP, Marana SR, Terra WR, Ferreira C. Purification, molecular cloning, and properties of a β-glycosidase isolated from midgut lumen of Tenebrio molitor (Coleoptera) larvae. Insect Biochem. Mol. Biol. 2001; 31:1065–76.

36. Tokuda G, Saito H, Watanabe H. A digestive β-glucosidase from the salivary glands of the termite, *Neotermes koshunensis* (Shiraki): Distribution, characterization and isolation of its precursor cDNA by 5'- and 3'-RACE amplifications with degenerate primers. Insect Biochem. Mol. Biol. 2002; 32:1681–9.

37. Sakamoto K, Uji S, Kurokawa T, Toyohara H. Molecular cloning of endogenous β-glucosidase from common Japanese brackish water clam Corbicula japonica. Gene. 2009; 435:72–9.

38. Zhu L, Wu Q, Dai J, Zhang S, Wei F. Evidence of cellulose metabolism by the giant panda gut microbiome. Proc. Natl. Acad. Sci. USA. 2011; 108:17714–9.

39. Bird NC, Mabee PM. Developmental morphology of the axial skeleton of the zebrafish, *Danio rerio* (Ostariophysi: Cyprinidae). Dev. Dyn. 2003; 228:337–57.

40. Ortega N, Behonick DJ, Werb Z. Matrix remodeling during endochondral ossification. Trends Cell Biol. 2004; 14:86–93.

41. Chen G, Deng C, Li YP. TGF-β and BMP signaling in osteoblast differentiation and bone formation. Int. J. Biol. Sci. 2012; 8:272–88.

42. Harada S, Rodan GA. Control of osteoblast function and regulation of bone mass. Nature. 2003; 423:349–55.

43. Long F. Building strong bones: molecular regulation of the osteoblast lineage. Nat. Rev. Mol. Cell Biol. 2011; 13:27–38.

44. Boyle WJ, Simonet WS, Lacey DL. Osteoclast differentiation and activation. Nature. 2003; 423:337–42.

45. Benson G. Tandem repeats finder: A program to analyze DNA sequences. Nucleic Acids Res. 1999; 27:573–80.

46. Jurka J, Kapitonov VV, Pavlicek A, Klonowski P, Kohany O, Walichiewicz J. Repbase Update, a database of eukaryotic repetitive elements. Cytogenet. Genome Res. 2005; 110:462–7.

47. Griffiths-Jones S, Moxon S, Marshall M, Khanna A, Eddy SR, Bateman A. Rfam: Annotating non-coding RNAs in complete genomes. Nucleic Acids Res. 2005; 33:121–4.

48. Birney E, Clamp M, Durbin R. GeneWise and Genomewise. Genome Res. 2004; 14:988–95.

49. Trapnell C, Pachter L, Salzberg SL. TopHat: Discovering splice junctions with RNA-Seq. Bioinformatics. 2009; 25:1105–11.

50. Trapnell C, Williams BA, Pertea G, Mortazavi A, Kwan G, Van Baren MJ, et al. Transcript assembly and quantification by RNA-Seq reveals unannotated transcripts and isoform switching during cell differentiation. Nat. Biotechnol. 2010; 28:511–5.

51. Elsik CG, Mackey AJ, Reese JT, Milshina NV, Roos DS, Weinstock GM. Creating a honey bee consensus gene set. Genome Biol. 2007; 8, R13.

52. Edgar RC. MUSCLE: Multiple sequence alignment with high accuracy and high throughput. Nucleic Acids Res. 2004; 32:1792–7.

53. Guindon S, Dufayard JF, Lefort V, Anisimova M, Hordijk W, Gascuel O. New algorithms and methods to estimate maximum-likelihood phylogenies: Assessing the performance of PhyML 3.0. Syst. Biol. 2010; 59:307–21.

54. Guindon S, Gascuel O. A simple, fast, and accurate algorithm to estimate large phylogenies by maximum likelihood. Syst. Biol. 2003; 52:696–704.

55. Yang Z. PAML 4: Phylogenetic analysis by maximum likelihood. Mol. Biol. Evol. 2007; 24:1586–91.

56. Yang Z, Rannala B. Bayesian estimation of species divergence times under a molecular clock using multiple fossil calibrations with soft bounds. Mol. Biol. Evol. 2006; 23:212–26.

57. Hahn MW, Demuth JP, Han SG. Accelerated rate of gene gain and loss in primates. Genetics. 2007; 177:1941–9.

58. Löytynoja A, Goldman N. An algorithm for progressive multiple alignment of sequences with insertions. Proc. Natl. Acad. Sci. USA. 2005; 102:10557–62.

59. Talavera G, Castresana J. Improvement of phylogenies after removing divergent and ambiguously aligned blocks from protein sequence alignments. Syst. Biol. 2007; 56:564–77.

60. Niimura Y. Evolutionary dynamics of olfactory receptor genes in chordates: interaction between environments and genomic contents. Hum. Genomics. 2009; 4:107–18.

61. Altschul S, Madden T, Schaffer A, Zhang J, Zhang Z, Miller W, et al. Gapped BLAST and PSI-BLAST: a new generation of protein database search programs. Nucleic Acids Res. 1997; 25:3389–402.

62. Kumar S, Nei M, Dudley J, Tamura K. MEGA: A biologist-centric software for evolutionary analysis of DNA and protein sequences. Brief. Bioinform. 2008; 9:299–306.

63. Saitou N, Nei M. The neighbor-joining method: a new method for reconstructing phylogenetic trees. Mol. Biol. Evol. 1987; 4:406–25.

64. Langmead B, Salzberg SL. Fast gapped-read alignment with Bowtie 2. Nat. Methods. 2012; 9:357–9.

65. Li B, Dewey CN. RSEM: accurate transcript quantification from RNA-Seq data with or without a reference genome. BMC Bioinformatics. 2011; 12, 323.

66. Mortazavi A, Williams BA, McCue K, Schaeffer L, Wold B. Mapping and quantifying mammalian transcriptomes by RNA-Seq. Nat. Methods. 2008; 5:621–8.

67. Robinson MD, Oshlack A. A scaling normalization method for differential expression analysis of RNA-seq data. Genome Biol. 2010; 11, R25.

68. Anders S, Huber W. Differential expression analysis for sequence count data. Genome Biol. 2010; 11, R106.

69. Kanehisa M, Araki M, Goto S, Hattori M, Hirakawa M, Itoh M, et al. KEGG for linking

genomes to life and the environment. Nucleic Acids Res. 2008; 36:480–4.

Figure Legends

Figure 1 Global view of the *M. amblycephala* genome and syntenic relationship between *Ctenopharyngodon idellus*, *M. amblycephala* and *Danio rerio*. (**A**) Global view of the *M. amblycephala* genome. From outside to inside, the genetic linkage map (a); Anchors between the genetic markers and the assembled scaffolds (b); Assembled chromosomes (c); GC content within a 50-kb sliding window (d); Repeat content within a 500-kb sliding window (e); Gene distribution on each chromosome (f); Different gene expression of three transcriptomes (g). (**B**) Syntenic relationship between *C. idellus* (a), *M. amblycephala* (b) and *D. rerio* (c) chromosomes.

Figure 2 Phylogenetic tree and comparison of orthologous genes in *M. amblycephala* and other fish species. (**A**) Phylogenetic tree of teleosts using 316 single copy orthologous genes. The color circles at the nodes shows the estimated divergence times using *O. latipes–F. rubripes* [96.9~150.9Mya], *F. rubripes–D. rerio* [149.85~165.2Mya], *F. rubripes–C. milii* [416~421.75Mya] (http://www.timetree.org/) as the calibration time. Pentagram represents four cyprinid fish with intermuscular bones. S, silurian period; D, devonian period; C, carboniferous period; P, permian periodin Paleozoic; T, triassic period; J, jurassic and k-cretaceous period in Mesozoic; Pg, paleogene in Cenozoic Era, N, Neogene. (**B**) Venn diagram of shared and unique orthologous gene families in *M. amblycephala* and four other teleosts. (**C**) Over-represented GO annotations of cyprinid-specific expansion gene families.

Figure 3 Regulation of genes related to intermuscular bone formation and function identified from developmental stages and adult tissues transcriptome data. (**A**) Gene expression pattern involved in muscle contraction regulated genes in early developmental stages corresponds to intermuscular bone formation of *M. amblycephala*, (alizarin red staining). M, myosepta; IB, intermusucular bone. (**B**) Scanning electron microscope photos of muscle tissues, connective tissues, and intermuscular bone. (**C**) Distribution of intermuscular bone specific genes in GO annotations indicative of abundance in protein binding, calcium ion binding, GTP binding functions. (**D**) Several developmental signals regulating key steps of osteoblast and osteoclast differentiation in the process of intramembranous ossification. Colored boxes indicate significantly up-regulated genes in these signals specifically occurred in intermuscular bone.

Figure 4 Molecular characteristics of sensory systems and the composition of gut microbiota in *M. amblycephala*. (**A**) Extensive expansion of olfactory receptor genes (ORs) in *M. amblycephala* compared with other teleosts. (**B**) Phylogeny of 'beta' type ORs in eight representative teleost species showing the significant expansion of 'beta' ORs in *M. amblycephala* and *C. idellus*. The pink background shows cyprinid-specific 'beta' types of ORs. (**C**) Umami, sweet and bitter tastes related gene families in teleosts with different feeding habits. (**D**) Structure of the umami receptor encoding T1R1 gene in cyprinid fish. (**E**) Relative abundance of microbial flora and taxonomic assignments in juvenile (LBSB), domestic adult (DBSB), wild adult (BSB) *M. amblycephala* and wild adult *C. idellus* (GC) samples at the phylum level.

Table

Table 1 Features of the *Megalobrama amblycephala* whole genome sequence

Total genome size (Mb)	1,116
N90 length of scaffold (bp)	20,422
N50 length of scaffold (bp)	838,704
N50 length of contig (bp)	49,400
Total GC content (%)	37.30
Protein-coding genes number	23,696
Average gene length (bp)	15,797
Content of transposable elements (%)	34.18
Number of chromosomes	24
Number of makers in genetic map	5,317
Scaffolds anchored on linkage groups (LGs)	1,434
Length of scaffolds anchored on LGs (Mb)	779.54 (70%)

Figure1

Click here to download Figure 2 Figure 1.pdf

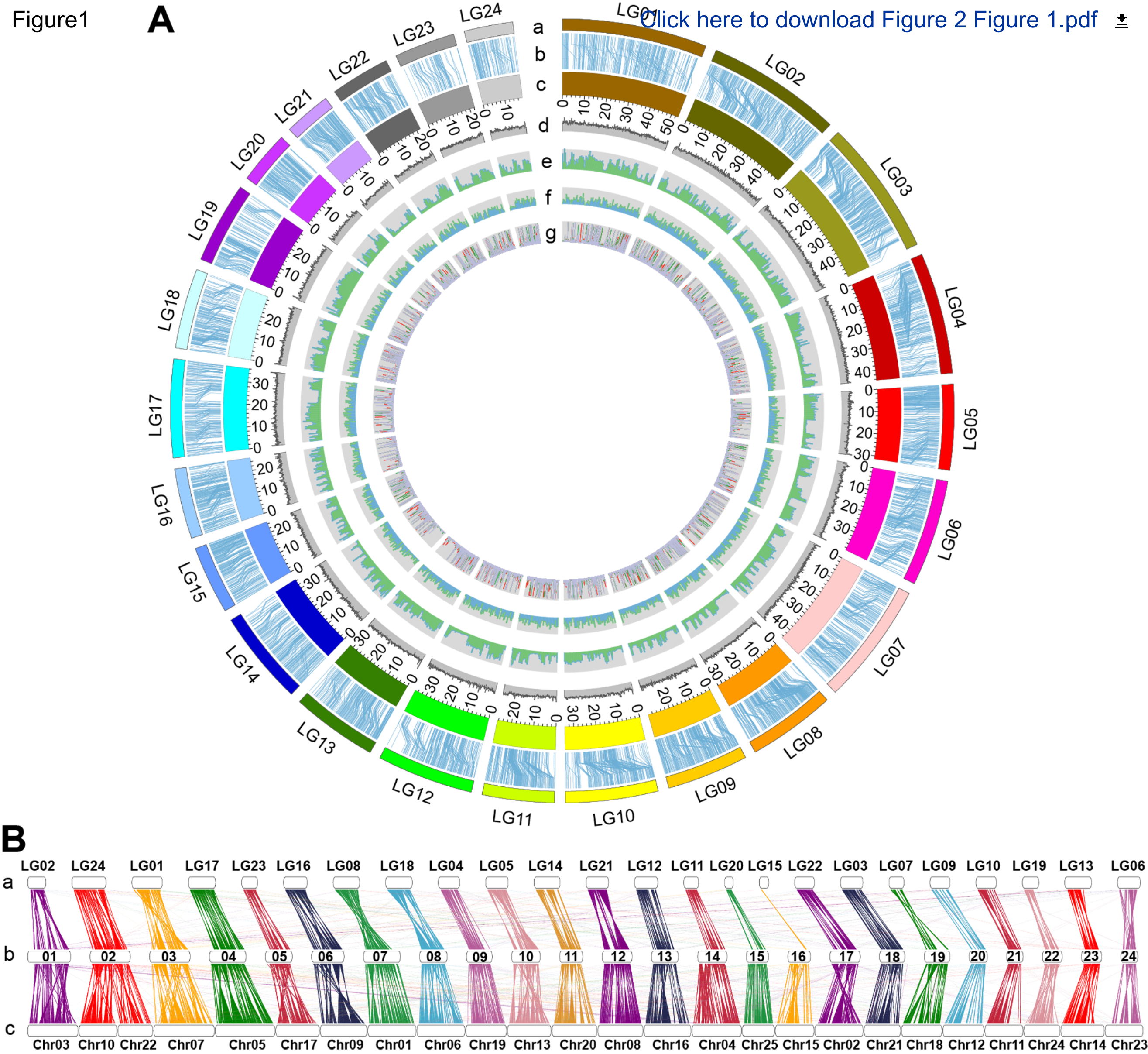

Figure2

Click here to download Figure 2 Figure 2.pdf

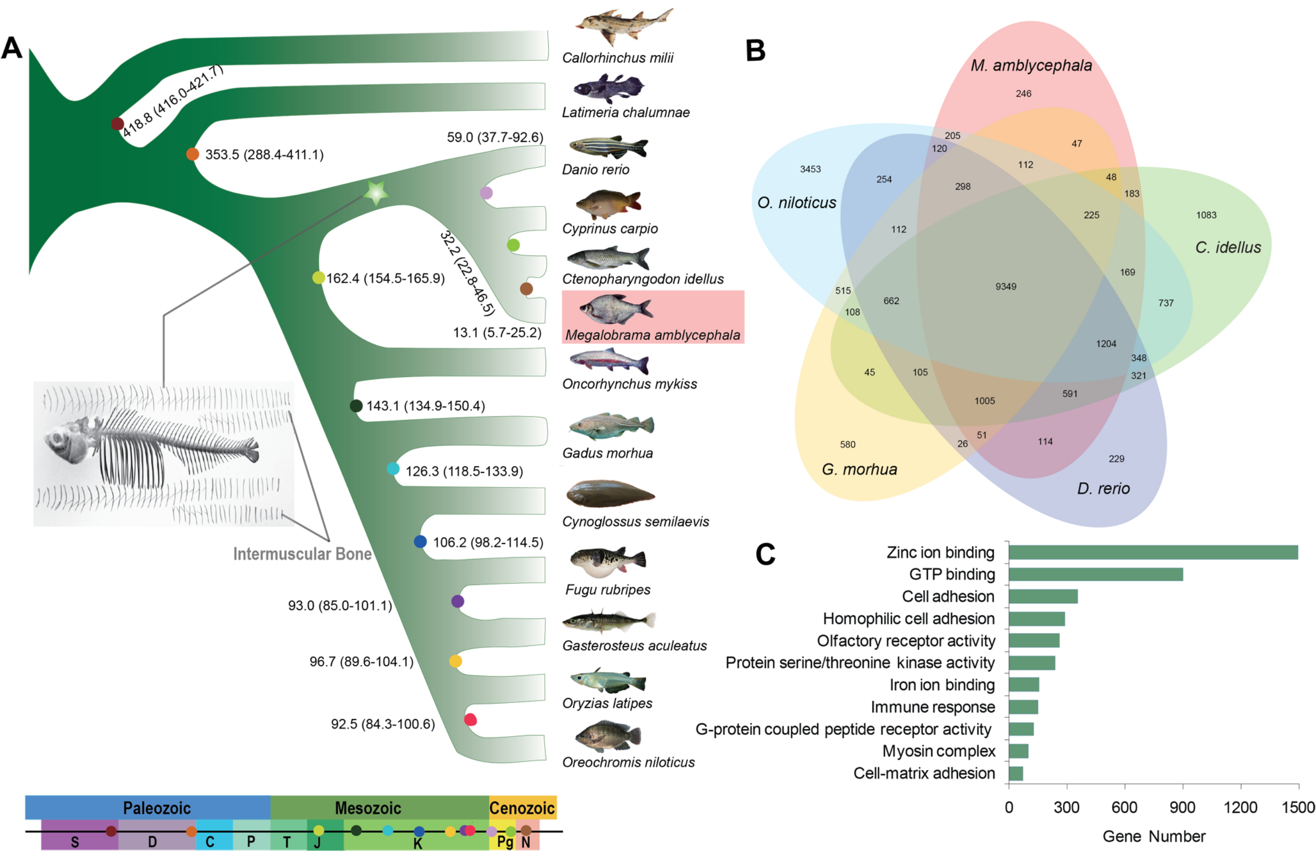

Figure 3

Click here to download Figure 2 Figure 3.pdf

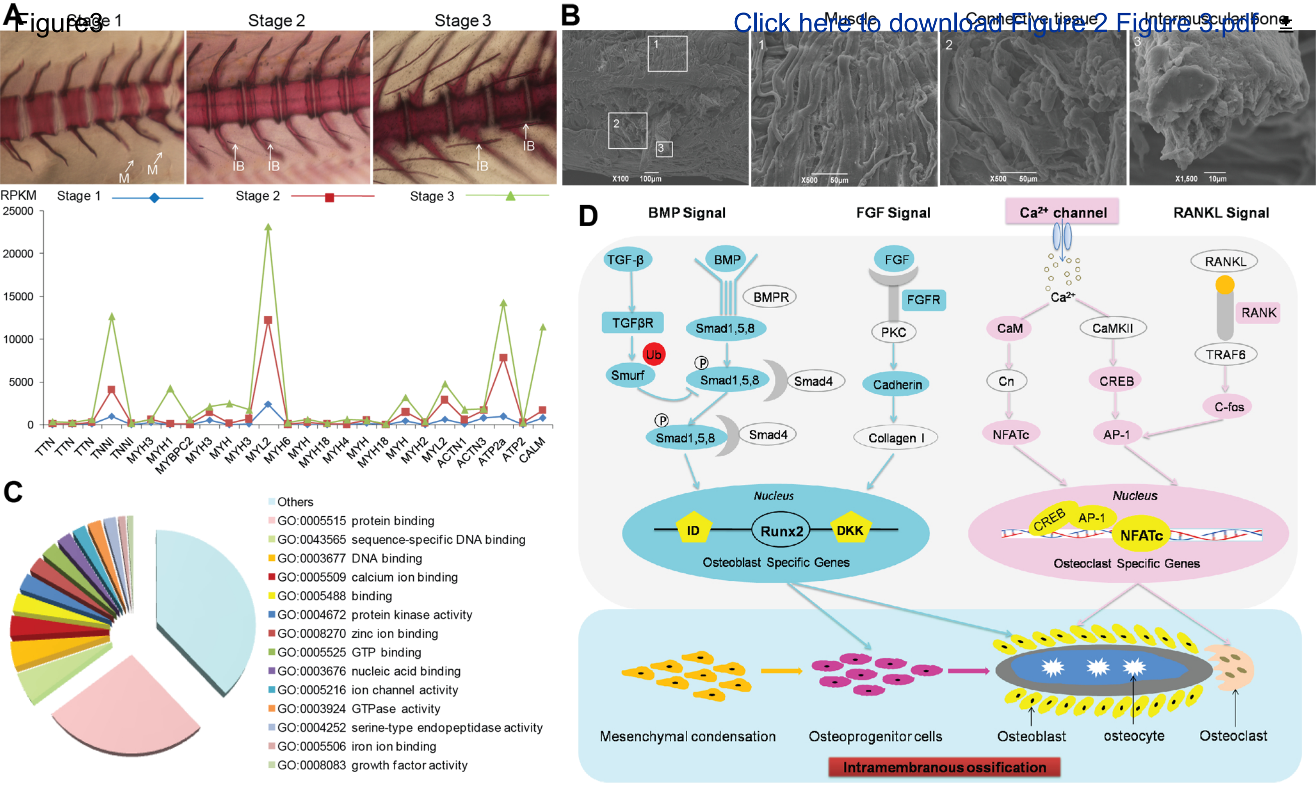

Figure4

Click here to download Figure 2 Figure 4.pdf

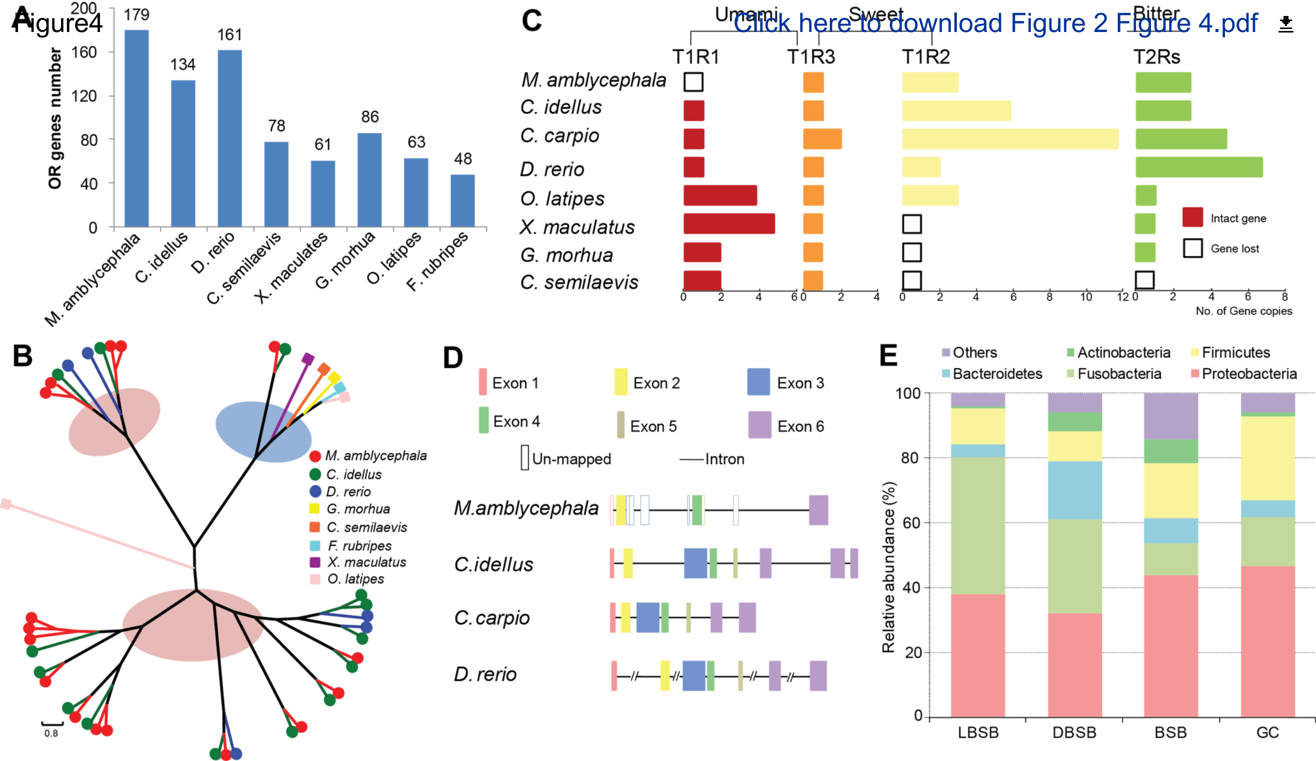

Click here to access/download
Supplementary Material
3 2016.9.8 Supplementary Material.pdf

Click here to access/download
Supplementary Material
3 Supplementary Note 1.xlsx
