# Supplementary material for: The draft genome of blunt snout bream (Megalobrama amblycephala) reveals the development of intermuscular bone and adaptation to herbivorous diet
Source: Gigascience. 2017 May 23;6(7):1–13. doi: 10.1093/gigascience/gix039 (PMC5570040; doi:10.1093/gigascience/gix039)
Supplement: GIGA-D-16-00088_Revision-1.pdf [file gix039_GIGA-D-16-00088_Revision-1.pdf]

GigaScience

The draft genome of Megalobrama amblycephala reveals the development of intermuscular bone and adaptation to herbivorous diet

--Manuscript Draft--

Manuscript Number:	GIGA-D-16-00088R1
Full Title:	The draft genome of Megalobrama amblycephala reveals the development of intermuscular bone and adaptation to herbivorous diet
Article Type:	Research
Funding Information:	
Abstract:	Background: The blunt snout bream, Megalobrama amblycephala, is the economically most important cyprinid fish species. As an herbivore, it can be grown by eco-friendly and resource-conserving aquaculture. However, the large number of intermuscular bones in the trunk musculature is adverse to fish meat processing and consumption.<br>Results: As a first towards optimizing this aquatic livestock, we present a 1.116-Gb draft genome of M. amblycephala, with 779.54 Mb anchored on 24 linkage groups. Integrating spatiotemporal transcriptome analyses we show that intermuscular bone is formed in the more basal teleosts by intramembranous ossification, and may be involved in muscle contractibility and coordinating cellular events. Comparative analysis revealed that olfactory receptor genes, especially of the beta type, underwent an extensive expansion in herbivorous cyprinids, whereas the gene for the umami receptor T1R1 was specifically lost in M. amblycephala. The composition of gut microflora, which contributes to the herbivorous adaptation of M. amblycephala, was found to be similar to that of other herbivores.<br>Conclusions: As a valuable resource for improvement of M. amblycephala livestock, the draft genome sequence offers new insights into the development of intermuscular bone and herbivorous adaptation.
Corresponding Author:	Weimin Wang<br><br>CHINA
Corresponding Author Secondary Information:	
Corresponding Author's Institution:	
Corresponding Author's Secondary Institution:	
First Author:	Han Liu
First Author Secondary Information:	
Order of Authors:	Han Liu
	Chunhai Chen
	Zexia Gao
	Jiumeng Min
	Yongming Gu
	Jianbo Jian
	Xiewu Jiang
	Huimin Cai
	Ingo Ebersberger
	Meng Xu
	Xinhui Zhang
	Jianwei Chen

Powered by Editorial Manager® and ProduXion Manager® from Aries Systems Corporation

	Wei Luo
	Boxiang Chen
	Junhui Chen
	Hong Liu
	Jiang Li
	Ruifang Lai
	Mingzhou Bai
	Jin Wei
	Shaokui Yi
	Huanling Wang
	Xiaojuan Cao
	Xiaoyun Zhou
	Yuhua Zhao
	Kaijian Wei
	Ruibin Yang
	Bingnan Liu
	Shancen Zhao
	Xiaodong Fang
	Manfred Schartl
	Xueqiao Qian
	Weimin Wang
**Order of Authors Secondary Information:**	
**Response to Reviewers:**	24 November 2016<br>Dr. Hans Zauner<br>Journal: GigaScience<br><br>Dear Dr. Zauner,<br>Manuscript No.: GIGA-D-16-00088<br>Title: "The draft genome of Megalobrama amblycephala reveals the development of intermuscular bone and adaptation to herbivorous diet"<br>Author(s): Han Liu, Chunhai Chen, Zexia Gao, Jiumeng Min, Yongming Gu, Jianbo Jian, Xiewu Jiang, Huimin Cai, Ingo Ebersberger, Meng Xu, Xinhui Zhang, Jianwei Chen, Wei Luo, Boxiang Chen, Junhui Chen, Hong Liu, Jiang Li, Ruifang Lai, Mingzhou Bai, Jin Wei, Shaokui Yi, Huanling Wang, Xiaojuan Cao, Xiaoyun Zhou, Yuhua Zhao, Kaijian Wei, Ruibin Yang, Bingnan Liu, Shancen Zhao, Xiaodong Fang, Manfred Schartl, Xueqiao Qian, Weimin Wang<br><br>We have carefully read the referees’ comments which you forwarded to us with your email of 2 November 2016. We would like to express our sincere thanks to the reviewers for the constructive and positive comments. We have addressed all their suggestions, and the manuscript has been edited accordingly. Please find below our responses to the comments of the referees. The major amendments are highlighted in red in the revised manuscript. Responses to each of the reviewers’ comments are detailed below in this letter. Because of the amendments, the page and the line numbers referred to by the referees have now changed in the edited version of the manuscript. Please note that all raw reads of genome sequencing and RAD-seq have been deposited at NCBI. Other data including assemblies, annotations, RNA-seq data, microbiome data and embedded image data (intermuscular bones and histological stainings) were uploaded to the indicated ftp. We hope that with the amendments made in response to the reviewers’ comments, the manuscript is now acceptable for

Powered by Editorial Manager® and ProduXion Manager® from Aries Systems Corporation

publication in GigaScience.
I look forward to hearing from you soon.

Yours sincerely,

Weimin Wang (PhD) (Correspondence author)
College of Fisheries
Huazhong Agricultural University
Wuhan 430070, P. R. China
E-mail address: wangwm@mail.hzau.edu.cn
Tel: +86-27-8728 4292; Fax: +86-27-8728 4292

Response to Reviewers
Reviewer Reports
Reviewer 1:
It is clear that a lot of work has gone into the creation of a draft genome for Megalobrama amblycephala. The creation of a genetic map to help anchor the assembly is significant and I am please to see it. Their results and discussion flow logically from the genome assembly and annotation as compared with other fish species. Although every bioinformatician has their favorite open source program, the software used in the construction of the assembly and annotation are well known and documented.
I recommend acceptance with the following comments and strong recommendations.
1) All raw data for the assembly and annotation be submitted to a public database.

Author response: The raw sequencing data have been deposited at NCBI under the accession number SRP090157 and raw data is available from the SRA under bioproject number PRJNA343584. We also have uploaded the assemblies, annotations, RNA-seq and microbiome data to the ftp (ftp://user28@climb.genomics.cn).

2) All raw data and markers for the genetic map be submitted to a public database or made readily downloadable.

Author response: All the raw data and markers for the genetic map have been deposited at NCBI in the SRA under bioproject number PRJNA343584 and biosample number SRS1797758.

3) The genome assembly be checked for contamination. This can be easily done with programs like blobtools and megablast. If contamination is found please remove it prior to submitting to the public databases and update the paper accordingly. In this way we do not contaminate and propagate contamination in our common public databases.

Author response: We have checked the genome assembly for contamination. The results are shown as bellow:
Scaffold_IDStartEndCover_LenScaffold_LenCoverage (%)Tax_IDOrganism
scaffold16634177975695878.911148Synechocystis sp.
scaffold1939712958345590850.11111780Stanieria cyanosphaera
scaffold1189010239429352356.021287681Eutypa lata
Only one gene MamblycephalaGene23156.1 is involved in the contamination region. We have now shielded them and updated the gene and genome data in the indicated database (ftp://user28@climb.genomics.cn).

4) Consider swapping out some of the language of "plays a role" with phrases like "are involved in" or "have reported to" or "have a role in" etc.;

Author response: We have replaced the expression of "plays a role" by "be involved in" or "be associated with" throughout the whole manuscript.

5) On Line 241, you have written bittern, I suspect you either meant bitter or bitterness.

Author response: This was a language error on the out side and it should read bitterness. We now have amended it in the revised manuscript.

6) I downloaded and examined the annotation file Megalobrama_amblycephala.gff. It only contains transcript models and no gene models. You state in the paper there were 23,696 protein coding genes. This number corresponds to the number of mRNA models you have in this gff file. This struck me as odd as there are usually isoforms for gene models. This file also did not contain any functional annotation. Please create a gff3 file that contains genes models and include functional annotation as this will be of great value to researchers.

Author response: We have created a gff3 file that contains genes models and functional annotations. This will be available in the GigaDB repository associated with the paper publication. The format of this file as shown below:

This is an important work for the development of sustainable aquaculture. Thank you.

Reviewer 2:
The draft genome of Megalobrama amblycephala reveals the development of intermuscular bone and adaptation to herbivorous diet by Liu H., et al. The present manuscript by Liu H., et al. can be divided into three main parts. The first part deals with the genome sequencing of one of the economically important cyprinid fish species Megalobrama amblycephala. Authors have generated a draft genome of 1.116 GB and managed to link 779.54 Mb to 24 linkage groups. Linkage groups were also constructed in the present work. Authors further investigate comparative genomics and phylogenetic analysis using the generated data. The second part investigates the characteristics of the feeding strategy of Megalobrama amblycephala being an herbivore species.

1) This comprised analysis of “expanded gene families” (this expression is rather unfortunate and not clear. It does not show which gene families finally were looked at and supplementary material like figure S11 does not say much) as well as the gut microbial community, but no differential expression analysis (mRNA). In the method section the experimental set up and analysis of the gut microbial study is missing.

Author response: We have now clarified these questions according to the reviewer’s suggestions. The expanded genes in the M. amblycephala and C. idellus lineage are now listed in the Additional file 2: Data Note1. The gut microbial community of the larvae (LBSB), domestic adult (DBSB), wild adult (BSB) M. amblycephala and wild adult C. idellus (GC) at phylum level and genus level are shown in Figure 4E and Additional file 1: Table S16, respectively. The sequences information and alpha diversity indexes are shown in Table S15 in the revised Additional file 1. We have added the experimental set up and analysis of the gut microbial study in the method section of the revised manuscript.

2) The third part studies the development of intermuscular bones comprising the analysis of “expanded gene families” (again figure 2B does not explain the “expanded gene families”) and transcriptome analysis of early developmental stages.

Author response: We apologize for the mistake. Reference should have been made to Figure 2C and not to Figure 2B. This has been corrected in the revised manuscript.

3) Authors present a huge amount of data and analysis but finally do describe and discuss only a little part of their analysis mainly in form of a very small set of genes. The manuscript is well, but not straightforward written. The structure has to be revised and the outcome better worked out.

Powered by Editorial Manager® and ProduXion Manager® from Aries Systems Corporation

Author response: We have revised our manuscript according to your suggestions and modified and renumbered the figures and tables clearly.

INTRODUCTION
1) LINE 64: Today many genomes (draft and nearly complete) are available. It is suggested to categorize them into fresh water, Mediterranean and Atlantic sea important aquacultured species. One species e.g. important for the Mediterranean aquaculture, The European sea bass (Dicentrarchus labrax) which was recently sequenced is not listed (European sea bass genome and its variation provide insights into adaptation to euryhalinity and speciation. Nature communications, 2014 5, 5770.)

Author response: This important species, the European sea bass, has been listed now in the revised manuscript (Line 65).

2) LINE 68-71: Please re-phrase the sentence. It is not true that the focus of aquaculture is focusing on herbivorous species. It is true however, that the usage of alternative feed is pursued but not the culture of herbivorous species. In contrast several projects are working on new species (including carnivores) for aquaculture purposes.

Author response: This sentence has been re-phrased as "Reports on draft genomes of some resource friendly herbivorous and omnivorous species, in particular cyprinid fish are scarce" in the revised manuscript (Line 69-70).

3) LINE 79 and LINE 84-85: Please refer to Wan et al., 2016 "Dynamic mRNA and miRNA expression analysis in response to intermuscular bone development of blunt snout bream (Megalobrama amblycephala)", Scientific Reports.

Author response: We have added this research paper as a reference in the revised manuscript (Line 80-83 and Line 88-89).

DATA DESCRIPTION
1) Description of generated SNP linkage map appears here, but does not appear in the method section. Please accomplish the method section.

Author response: We have added such information in detail now in Data Description section (Line 128-133) and also added the description of the genetic map construction in the method section (Line 403-416) in the revised manuscript.

ANALYSES SECTION
1) According to GigaScience Authors guide: "This section should provide details of all of the experiments and analyses that are required to support the conclusions of the paper. The authors should make clear the goal of each analysis and state the basic findings". Information about analyses (except the linkage map analysis) is partially found in the section named "Results" as well as in the "Method" section.

Author response: We have now modified this section according to your suggestions. We stated the goal and main finding of each analysis in the Results section and also removed the reporting of findings from the Method section.

2) LINES147-153: It is not clear why the enrichment analyses shows that the adaptation to herbivory goes "hand in hand" with the coping with plant secondary metabolites. Do authors have a comparable analysis of a carnivore or omnivore teleosts?

Author response: This sentence was ambiguous and we apologize for the confusion that it generated. We have now changed this expression to "These genes encoding proteins involved in biodegradation of xenobiotics would enhance the ability of an

herbivore to detoxify the secondary compounds present in grasses that are adverse or even toxic to the organism." (Line 161-164).

3) LINE 170: What do authors mean by the expression "comparative transcriptome analysis"?

Author response: This expression was not accurate. We have now modified the expression and write "differential genes expression (DGE) analysis" or "transcriptome analysis" at the appropriate positions of the manuscript.

4) LINE 174: "notable". This result is not really surprising. Many other transcriptome studies in teleost have also shown that at the later stages, where the larvae is mainly growing, mostly muscle genes are up-regulated when compared to earlier stages.

Author response: We agree. This word has been deleted in the revised manuscript.

5) LINE 183: change the expression "eventually extended".

Author response: This expression has been changed to "we performed differential genes expression (DGE) analysis of" in the revised manuscript (Line 192).

6) LINE 203. The link to Figure 3D is not clear. Is Figure 3D showing all 35 identified genes?

Author response: Figure 3D shows several developmental signals regulating key steps of osteoblast and osteoclast differentiation in the process of intramembranous ossification. In Figure 3D, only the colored boxes indicate these 35 identified genes. To make this more clear, we have amended this sentence to "35 bone formation regulatory genes were identified (shown in colored boxes in Figure 3D)" (Line 212).

DISCUSSION
Mainly a repetition of the previous paragraphs.
1) LINE 134 and 271: Authors did not show that Dre10 and Dre22 are 'ancestral', just that those two chromosomes fused to one chromosome in blunt snout bream as this species has 24 chromosomes while zebrafish has 25 chromosomes. Please take into account publications like Nakatani, Y., H. Takeda, Y. Kohara, and S. Morishita, 2007 Reconstruction of the vertebrate ancestral genome reveals dynamic genome reorganization in early vertebrates. Genome Res. 17: 1254–1265 and. Hufton, A. L., D. Groth, M. Vingron, H. Lehrach, A. J. Poustka et al., 2008 Early vertebrate whole genome duplications were predated by a period of intense genome rearrangement. Genome Res. 18: 1582–1591.

Author response: According to the referee's suggestion and taking into account the two references, we have modified this sentence to "The most prominent event is a chromosomal fusion in M. amblycephala LG02 that joined two D. rerio chromosomes, Dre10 and Dre22".

2) LINE 311: 'comparative transcriptome" This expression leads the reader to the false impression that more than one species was studied. However authors investigated here in differential expression.

Author response: The expression "comparative transcriptome" has been amended to "differential genes expression (DGE) analysis" or "transcriptome analysis" in the appropriate positions of the manuscript.

METHODS
1) Missing description of microbial community study as well as generation of linkage

Powered by Editorial Manager® and ProduXion Manager® from Aries Systems Corporation

	map.—<br><br>Author response: We have now added more detailed information in the Methods section including genome assembly, construction of gene families, the microbiota analysis, genetic map construction and other related information.
**Additional Information:**	
**Question**	**Response**
Are you submitting this manuscript to a special series or article collection?	No
**Experimental design and statistics**<br><br>Full details of the experimental design and statistical methods used should be given in the Methods section, as detailed in our Minimum Standards Reporting Checklist. Information essential to interpreting the data presented should be made available in the figure legends.<br><br>Have you included all the information requested in your manuscript?	Yes
**Resources**<br><br>A description of all resources used, including antibodies, cell lines, animals and software tools, with enough information to allow them to be uniquely identified, should be included in the Methods section. Authors are strongly encouraged to cite Research Resource Identifiers (RRIDs) for antibodies, model organisms and tools, where possible.<br><br>Have you included the information requested as detailed in our Minimum Standards Reporting Checklist?	Yes
**Availability of data and materials**<br><br>All datasets and code on which the conclusions of the paper rely must be either included in your submission or deposited in publicly available repositories (where available and ethically appropriate), referencing such data using a unique identifier in the references and in the “Availability of Data and Materials” section of your manuscript.<br><br>Have you have met the above requirement as detailed in our Minimum Standards Reporting Checklist?	Yes

Powered by Editorial Manager® and ProduXion Manager® from Aries Systems Corporation

 Click here to download Manuscript 0 Response to Reviewers.docx

24 November 2016

Dr. Hans Zauner

Journal: GigaScience

Dear Dr. Zauner,

Manuscript No.: GIGA-D-16-00088

Title: **"The draft genome of *Megalobrama amblycephala* reveals the development of intermuscular bone and adaptation to herbivorous diet"**

Author(s): Han Liu, Chunhai Chen, Zexia Gao, Jiumeng Min, Yongming Gu, Jianbo Jian, Xiewu Jiang, Huimin Cai, Ingo Ebersberger, Meng Xu, Xinhui Zhang, Jianwei Chen, Wei Luo, Boxiang Chen, Junhui Chen, Hong Liu, Jiang Li, Ruifang Lai, Mingzhou Bai, Jin Wei, Shaokui Yi, Huanling Wang, Xiaojuan Cao, Xiaoyun Zhou, Yuhua Zhao, Kaijian Wei, Ruibin Yang, Bingnan Liu, Shancen Zhao, Xiaodong Fang, Manfred Schartl, Xueqiao Qian, Weimin Wang

We have carefully read the referees' comments which you forwarded to us with your email of 2 November 2016. We would like to express our sincere thanks to the reviewers for the constructive and positive comments. We have addressed all their suggestions, and the manuscript has been edited accordingly. Please find below our responses to the comments of the referees. The major amendments are highlighted in red in the revised manuscript. Responses to each of the reviewers' comments are detailed below in this letter. Because of the amendments, the page and the line numbers referred to by the referees have now changed in the edited version of the manuscript. Please note that all raw reads of genome sequencing and RAD-seq have been deposited at NCBI. Other data including assemblies, annotations, RNA-seq data, microbiome data and embedded image data (intermuscular bones and histological stainings) were uploaded to the indicated ftp. We hope that with the amendments made in response to the reviewers' comments, the manuscript is now acceptable for publication in GigaScience.

I look forward to hearing from you soon.

Yours sincerely,

Weimin Wang (PhD) (Correspondence author)

College of Fisheries

Huazhong Agricultural University

Wuhan 430070, P. R. China

E-mail address: wangwm@mail.hzau.edu.cn

Tel: +86-27-8728 4292; Fax: +86-27-8728 4292

Response to Reviewers

Reviewer Reports

Reviewer 1:

It is clear that a lot of work has gone into the creation of a draft genome for *Megalobrama amblycephala*. The creation of a genetic map to help anchor the assembly is significant and I am please to see it. Their results and discussion flow logically from the genome assembly and annotation as compared with other fish species. Although every bioinformatician has their favorite open source program, the software used in the construction of the assembly and annotation are well known and documented.

I recommend acceptance with the following comments and strong recommendations.

1) All raw data for the assembly and annotation be submitted to a public database.

Author response: The raw sequencing data have been deposited at NCBI under the accession number SRP090157 and raw data is available from the SRA under bioproject number PRJNA343584. We also have uploaded the assemblies, annotations, RNA-seq and microbiome data to the ftp (ftp://user28@climb.genomics.cn).

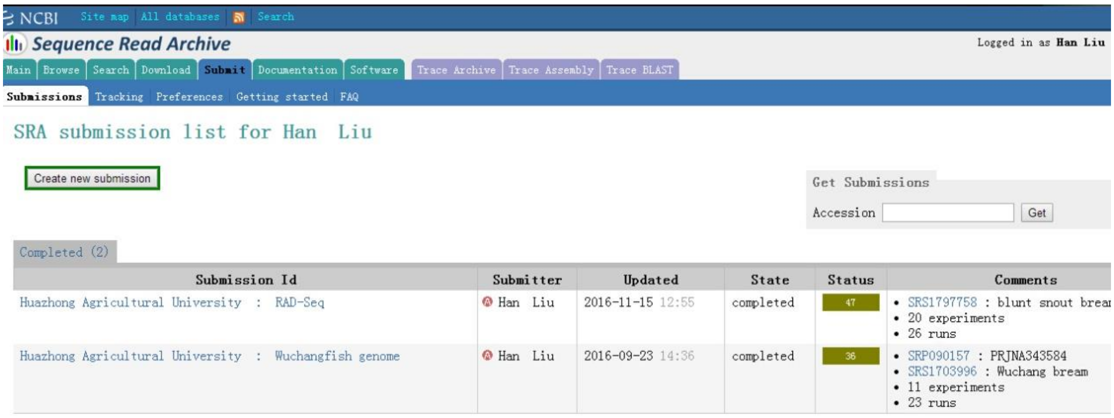

2) All raw data and markers for the genetic map be submitted to a public database or made readily downloadable.

Author response: All the raw data and markers for the genetic map have been deposited at NCBI in the SRA under bioproject number PRJNA343584 and biosample number SRS1797758.

3) The genome assembly be checked for contamination. This can be easily done with programs like blobtools and megablast. If contamination is found please remove it prior to submitting to the public databases and update the paper accordingly. In this way we do not contaminate and propagate contamination in our common public databases.

Author response: We have checked the genome assembly for contamination. The results are shown as bellow:

Scaffold_ID	Start	End	Cover_Len	Scaffold_Len	Coverage (%)	Tax_ID	Organism
scaffold16634	1	779	756	958	78.91	1148	*Synechocystis sp.*
scaffold19397	129	583	455	908	50.11	111780	*Stanieria cyanosphaera*
scaffold11890	102	394	293	523	56.02	1287681	*Eutypa lata*

Only one gene MamblycephalaGene23156.1 is involved in the contamination region. We have now shielded them and updated the gene and genome data in the indicated database (ftp://user28@climb.genomics.cn).

4) Consider swapping out some of the language of "plays a role" with phrases like "are involved in" or "have reported to" or "have a role in" etc.;

Author response: We have replaced the expression of "plays a role" by "be involved in" or "be associated with" throughout the whole manuscript.

5) On Line 241, you have written bittern, I suspect you either meant bitter or bitterness.

Author response: This was a language error on the out side and it should read bitterness. We now have amended it in the revised manuscript.

6) I downloaded and examined the annotation file Megalobrama_amblycephala.gff. It only contains transcript models and no gene models. You state in the paper there were 23,696 protein coding genes. This number corresponds to the number of mRNA models you have in this gff file. This struck me as odd as there are usually isoforms for gene models. This file also did not contain any functional annotation. Please create a gff3 file that contains genes models and include functional annotation as this will be of great value to researchers.

Author response: We have created a gff3 file that contains genes models and functional

annotations. This will be available in the GigaDB repository associated with the paper publication.

The format of this file as shown below:

1	Chr09	GLEAN	gene	11631717	11642632	0.984234	+	.	ID=MamblycephalaGene06643;name=MamblycephalaGene06643
2	Chr09	GLEAN	mRNA	11631717	11642632	0.984234	+	.	ID=MamblycephalaGene06643.1;name=MamblycephalaGene06643.1;symbol=atat1;Info=Alpha-tubulin N-acetyltransfer
3	Chr09	GLEAN	CDS	11631717	11631808	.	+	0	ID=MamblycephalaGene06643.1.CDS.1;Parent=MamblycephalaGene06643.1
4	Chr09	GLEAN	CDS	11634113	11634173	.	+	1	ID=MamblycephalaGene06643.1.CDS.2;Parent=MamblycephalaGene06643.1
5	Chr09	GLEAN	CDS	11634280	11634371	.	+	0	ID=MamblycephalaGene06643.1.CDS.3;Parent=MamblycephalaGene06643.1
6	Chr09	GLEAN	CDS	11634541	11634598	.	+	1	ID=MamblycephalaGene06643.1.CDS.4;Parent=MamblycephalaGene06643.1
7	Chr09	GLEAN	CDS	11634714	11634827	.	+	0	ID=MamblycephalaGene06643.1.CDS.5;Parent=MamblycephalaGene06643.1
8	Chr09	GLEAN	CDS	11636147	11636248	.	+	0	ID=MamblycephalaGene06643.1.CDS.6;Parent=MamblycephalaGene06643.1
9	Chr09	GLEAN	CDS	11639246	11639363	.	+	0	ID=MamblycephalaGene06643.1.CDS.7;Parent=MamblycephalaGene06643.1
10	Chr09	GLEAN	CDS	11642364	11642632	.	+	2	ID=MamblycephalaGene06643.1.CDS.8;Parent=MamblycephalaGene06643.1
11	Chr09	GLEAN	gene	11668751	11669943	0.9994	+	.	ID=MamblycephalaGene06645;name=MamblycephalaGene06645
12	Chr09	GLEAN	mRNA	11668751	11669943	0.9994	+	.	ID=MamblycephalaGene06645.1;name=MamblycephalaGene06645.1;symbol=C5orf49;Info=Uncharacterized protein C5or
13	Chr09	GeneWise	CDS	11668751	11668885	.	+	0	ID=MamblycephalaGene06645.1.CDS.1;Parent=MamblycephalaGene06645.1
14	Chr09	GLEAN	CDS	11669516	11669632	.	+	0	ID=MamblycephalaGene06645.1.CDS.2;Parent=MamblycephalaGene06645.1
15	Chr09	GLEAN	CDS	11669767	11669943	.	+	0	ID=MamblycephalaGene06645.1.CDS.3;Parent=MamblycephalaGene06645.1
16	Chr09	GLEAN	gene	11336195	11340397	0.9994	+	.	ID=MamblycephalaGene06633;name=MamblycephalaGene06633
17	Chr09	GLEAN	mRNA	11336195	11340397	0.9994	+	.	ID=MamblycephalaGene06633.1;name=MamblycephalaGene06633.1;symbol=RDBP;Info=Negative elongation factor E
18	Chr09	GeneWise	CDS	11336195	11336284	.	+	0	ID=MamblycephalaGene06633.1.CDS.1;Parent=MamblycephalaGene06633.1
19	Chr09	GLEAN	CDS	11336572	11336641	.	+	0	ID=MamblycephalaGene06633.1.CDS.2;Parent=MamblycephalaGene06633.1
20	Chr09	GLEAN	CDS	11336726	11336871	.	+	2	ID=MamblycephalaGene06633.1.CDS.3;Parent=MamblycephalaGene06633.1
21	Chr09	GLEAN	CDS	11336969	11337043	.	+	0	ID=MamblycephalaGene06633.1.CDS.4;Parent=MamblycephalaGene06633.1
22	Chr09	GLEAN	CDS	11337515	11337552	.	+	0	ID=MamblycephalaGene06633.1.CDS.5;Parent=MamblycephalaGene06633.1
23	Chr09	GLEAN	CDS	11337659	11337939	.	+	1	ID=MamblycephalaGene06633.1.CDS.6;Parent=MamblycephalaGene06633.1
24	Chr09	GLEAN	CDS	11338329	11338473	.	+	2	ID=MamblycephalaGene06633.1.CDS.7;Parent=MamblycephalaGene06633.1
25	Chr09	GLEAN	CDS	11339336	11339390	.	+	1	ID=MamblycephalaGene06633.1.CDS.8;Parent=MamblycephalaGene06633.1

This is an important work for the development of sustainable aquaculture. Thank you.

Reviewer 2:

The draft genome of *Megalobrama amblycephala* reveals the development of intermuscular bone and adaptation to herbivorous diet by Liu H., et al.

The present manuscript by Liu H., et al. can be divided into three main parts. The first part deals with the genome sequencing of one of the economically important cyprinid fish species *Megalobrama amblycephala*. Authors have generated a draft genome of 1.116 GB and managed to link 779.54 Mb to 24 linkage groups. Linkage groups were also constructed in the present work. Authors further investigate comparative genomics and phylogenetic analysis using the generated data. The second part investigates the characteristics of the feeding strategy of *Megalobrama amblycephala* being an herbivore species.

1) This comprised analysis of "expanded gene families" (this expression is rather unfortunate and not clear. It does not show which gene families finally were looked at and supplementary material like figure S11 does not say much) as well as the gut microbial community, but no differential expression analysis (mRNA). In the method section the experimental set up and analysis of the gut microbial study is missing.

Author response: We have now clarified these questions according to the reviewer's suggestions. The expanded genes in the *M. amblycephala* and *C. idellus* lineage are now listed in the Additional file 2: Data Note1. The gut microbial community of the larvae (LBSB), domestic adult (DBSB), wild adult (BSB) *M. amblycephala* and wild adult *C. idellus* (GC) at phylum level and genus level are shown in Figure 4E and Additional file 1: Table S16, respectively. The sequences

information and alpha diversity indexes are shown in Table S15 in the revised Additional file 1. We have added the experimental set up and analysis of the gut microbial study in the method section of the revised manuscript.

2) The third part studies the development of intermuscular bones comprising the analysis of “expanded gene families” (again figure 2B does not explain the “expanded gene families”) and transcriptome analysis of early developmental stages.

Author response: We apologize for the mistake. Reference should have been made to Figure 2C and not to Figure 2B. This has been corrected in the revised manuscript.

3) Authors present a huge amount of data and analysis but finally do describe and discuss only a little part of their analysis mainly in form of a very small set of genes. The manuscript is well, but not straightforward written. The structure has to be revised and the outcome better worked out.

Author response: We have revised our manuscript according to your suggestions and modified and renumbered the figures and tables clearly.

INTRODUCTION

1) LINE 64: Today many genomes (draft and nearly complete) are available. It is suggested to categorize them into fresh water, Mediterranean and Atlantic sea important aquacultured species. One species e.g. important for the Mediterranean aquaculture, The European sea bass (Dicentrarchus labrax) which was recently sequenced is not listed (European sea bass genome and its variation provide insights into adaptation to euryhalinity and speciation. Nature communications, 2014 5, 5770.)

Author response: This important species, the European sea bass, has been listed now in the revised manuscript (Line 65).

2) LINE 68-71: Please re-phrase the sentence. It is not true that the focus of aquaculture is focusing on herbivorous species. It is true however, that the usage of alternative feed is pursued but not the culture of herbivorous species. In contrast several projects are working on new species (including carnivores) for aquaculture purposes.

Author response: This sentence has been re-phrased as “Reports on draft genomes of some resource friendly herbivorous and omnivorous species, in particular cyprinid fish are scarce” in the revised manuscript (Line 69-70).

3) LINE 79 and LINE 84-85: Please refer to Wan et al., 2016 “Dynamic mRNA and miRNA expression analysis in response to intermuscular bone development of blunt snout bream (*Megalobrama amblycephala*)”, Scientific Reports.

Author response: We have added this research paper as a reference in the revised manuscript (Line 80-83 and Line 88-89).

DATA DESCRIPTION

1) Description of generated SNP linkage map appears here, but does not appear in the method section. Please accomplish the method section.

Author response: We have added such information in detail now in Data Description section (Line 128-133) and also added the description of the genetic map construction in the method section (Line 403-416) in the revised manuscript.

ANALYSES SECTION

1) According to GigaScience Authors guide: “This section should provide details of all of the experiments and analyses that are required to support the conclusions of the paper. The authors should make clear the goal of each analysis and state the basic findings”. Information about analyses (except the linkage map analysis) is partially found in the section named “Results” as well as in the “Method” section.

Author response: We have now modified this section according to your suggestions. We stated the goal and main finding of each analysis in the Results section and also removed the reporting of findings from the Method section.

2) LINES147-153: It is not clear why the enrichment analyses shows that the adaptation to herbivory goes “hand in hand” with the coping with plant secondary metabolites. Do authors have a comparable analysis of a carnivore or omnivore teleosts?

Author response: This sentence was ambiguous and we apologize for the confusion that it generated. We have now changed this expression to “These genes encoding proteins involved in biodegradation of xenobiotics would enhance the ability of an herbivore to detoxify the secondary compounds present in grasses that are adverse or even toxic to the organism.” (Line 161-164).

3) LINE 170: What do authors mean by the expression “comparative transcriptome analysis”?

Author response: This expression was not accurate. We have now modified the expression and write "differential genes expression (DGE) analysis" or "transcriptome analysis" at the appropriate positions of the manuscript.

4) LINE 174: "notable". This result is not really surprising. Many other transcriptome studies in teleost have also shown that at the later stages, where the larvae is mainly growing, mostly muscle genes are up-regulated when compared to earlier stages.

Author response: We agree. This word has been deleted in the revised manuscript.

5) LINE 183: change the expression "eventually extended".

Author response: This expression has been changed to "we performed differential genes expression (DGE) analysis of" in the revised manuscript (Line 192).

6) LINE 203. The link to Figure 3D is not clear. Is Figure 3D showing all 35 identified genes?

Author response: Figure 3D shows several developmental signals regulating key steps of osteoblast and osteoclast differentiation in the process of intramembranous ossification. In Figure 3D, only the colored boxes indicate these 35 identified genes. To make this more clear, we have amended this sentence to "35 bone formation regulatory genes were identified (shown in colored boxes in Figure 3D)" (Line 212).

DISCUSSION

Mainly a repetition of the previous paragraphs.

1) LINE 134 and 271: Authors did not show that Dre10 and Dre22 are 'ancestral', just that those two chromosomes fused to one chromosome in blunt snout bream as this species has 24 chromosomes while zebrafish has 25 chromosomes. Please take into account publications like Nakatani, Y., H. Takeda, Y. Kohara, and S. Morishita, 2007 Reconstruction of the vertebrate ancestral genome reveals dynamic genome reorganization in early vertebrates. Genome Res. 17: 1254–1265 and. Hufton, A. L., D. Groth, M. Vingron, H. Lehrach, A. J. Poustka et al., 2008 Early vertebrate whole genome duplications were predated by a period of intense genome rearrangement. Genome Res. 18: 1582–1591.

Author response: According to the referee's suggestion and taking into account the two references, we have modified this sentence to "The most prominent event is a chromosomal fusion in *M. amblycephala* LG02 that joined two *D. rerio* chromosomes, Dre10 and Dre22".

2) LINE 311: ‘comparative transcriptome” This expression leads the reader to the false impression that more than one species was studied. However authors investigated here in differential expression.

Author response: The expression “comparative transcriptome” has been amended to “differential genes expression (DGE) analysis” or “transcriptome analysis” in the appropriate positions of the manuscript.

METHODS

1) Missing description of microbial community study as well as generation of linkage map.—

Author response: We have now added more detailed information in the Methods section including genome assembly, construction of gene families, the microbiota analysis, genetic map construction and other related information.

1 Revised Manuscript (Text with changes Marked) Click here to download Manuscript 1 Revised Manuscript (Text with changes Marked).docx

The draft genome of *Megalobrama amblycephala* reveals the development of intermuscular bone and adaptation to herbivorous diet

Han Liu[1†], Chunhai Chen[2†], Zexia Gao[1†], Jiumeng Min[2†], Yongming Gu[3†], Jianbo Jian[2†], Xiewu Jiang[3], Huimin Cai[2], Ingo Ebersberger[4], Meng Xu[2], Xinhui Zhang[1], Jianwei Chen[2], Wei Luo[1], Boxiang Chen[1,3], Junhui Chen[2], Hong Liu[1], Jiang Li[2], Ruifang Lai[1], Mingzhou Bai[2], Jin Wei[1], Shaokui Yi[1], Huanling Wang[1], Xiaojuan Cao[1], Xiaoyun Zhou[1], Yuhua Zhao[1], Kaijian Wei[1], Ruibin Yang[1], Bingnan Liu[3], Shancen Zhao[2], Xiaodong Fang[2], Manfred Schartl[5,*], Xueqiao Qian[3,*], Weimin Wang[1,*]

[*]Equally contributing corresponding authors: wangwm@mail.hzau.edu.cn; xueqiaoqian@263.net; phch1@biozentrum.uni-wuerzburg.de

[†]Equal contributors

[1]College of Fisheries, Key Lab of Freshwater Animal Breeding, Ministry of Agriculture, Key Lab of Agricultural Animal Genetics, Breeding and Reproduction of Ministry of Education, Huazhong Agricultural University, Wuhan 430070, China

[2]Beijing Genomics Institute (BGI)-Shenzhen, Shenzhen 518083, China

[3]Guangdong Haid Group Co., Ltd., Guangzhou 511400, China

[4]Dept. for Applied Bioinformatics, Institute for Cell Biology and Neuroscience, Goethe University, Frankfurt D-60438, Germany

[5]Physiological Chemistry, University of Würzburg, Biozentrum, Am Hubland, and Comprehensive Cancer Center Mainfranken, University Clinic Würzburg, Würzburg 97070, Germany and Texas A&M Institute for Advanced Study and Department of Biology, Texas A&M University, College Station, TX 77843, USA

Abstract

Background: The blunt snout bream, *Megalobrama amblycephala*, is the economically most important cyprinid fish species. As an herbivore, it can be grown by eco-friendly and resource-conserving aquaculture. However, the large number of intermuscular bones in the trunk musculature is adverse to fish meat processing and consumption.

Results: As a first towards optimizing this aquatic livestock, we present a 1.116-Gb draft genome of *M. amblycephala*, with 779.54 Mb anchored on 24 linkage groups. Integrating spatiotemporal transcriptome analyses we show that intermuscular bone is formed in the more basal teleosts by intramembranous ossification, and may be involved in muscle contractibility and coordinating cellular events. Comparative analysis revealed that olfactory receptor genes, especially of the beta type, underwent an extensive expansion in herbivorous cyprinids, whereas the gene for the umami receptor *T1R1* was specifically lost in *M. amblycephala*. The composition of gut microflora, which contributes to the herbivorous adaptation of *M. amblycephala*, was found to be similar to that of other herbivores.

Conclusions: As a valuable resource for improvement of *M. amblycephala* livestock, the draft genome sequence offers new insights into the development of intermuscular bone and herbivorous adaptation.

Keywords: *Megalobrama amblycephala*, whole genome, herbivorous diet, intermuscular bone, transcriptome, gut microflora

Background

Fishery and aquaculture play an important role in global alimentation. Over the past decades food fish supply has been increasing with an annual rate of 3.6 percent, about 2 times faster than the human population [1]. This growth of fish production is meanwhile solely accomplished by an extension of aquaculture, as over the past thirty years the total mass of captured fish has remained almost constant [1]. As a consequence of this emphasis on fish breeding, the genomes of various economically important fish species, e.g. Atlantic cod (*Gadus morhua*) [2], rainbow trout (*Oncorhynchus mykiss*) [3], European sea bass (*Dicentrarchus labrax*) [4], yellow croaker (*Larimichthys crocea*) [5], half-smooth tongue sole (*Cynoglossus semilaevis*) [6], tilapia (*Oreochromis niloticus*) [7] and channel catfish (*Ictalurus punctatus*) [8] have been sequenced. Yet, the majority of these species are carnivorous requiring large inputs of protein from wild caught fish or other precious feed. Reports on draft genomes of some resource friendly herbivorous and omnivorous species, in particular cyprinid fish are scarce. It is well known that cyprinids are currently the economically most important group of teleosts for sustainable aquaculture. They grow to large population sizes in the wild and already now account for the majority of freshwater aquaculture production worldwide [1]. Among these, the herbivorous *Megalobrama amblycephala* (Yih, 1955), a particularly eco-friendly and resource-conserving species, is predominant in aquaculture and has been greatly developed in China (Additional file 1: Figure S1) [1]. However, most cyprinids, including *M. amblycephala*, have a large number of intermuscular bones (IBs) in the trunk musculature, which have an adverse effect on fish meat processing and consumption. IBs—a unique form of bone occurring only in the more basal teleosts—are completely embedded within the myosepta and are not connected to the vertebral column or any other bones [9, 10]. Our previous study on IB development of *M. amblycephala* revealed that some miRNA-mRNA interaction pairs may be involved in regulating bone development and differentiation [11]. However, the molecular genetic basis and the evolution of this unique structures remain obscure. Unfortunately, the recent sequencing of two cyprinid genomes common carp (*Cyprinus carpio*) [12] and grass carp (*Ctenopharyngodon idellus*) [13], which provided valuable information for their genetic breeding, contributed little to the understanding of IB formation.

In an initial genome survey of *M. amblycephala*, we identified 25,697 single-nucleotide polymorphism (SNP) [14], 347 conserved miRNAs [15], and many miRNA-mRNA interaction pairs [11]. However, lack of a whole genome sequence resource limited a thorough investigation of *M. amblycephala*. Here we report the first high-quality draft genome sequence of *M. amblycephala*. Integrating this novel genome resource with tissue- and developmental stage-specific gene expression information, as well as with meta-genome data to investigate the composition of the gut microbiome provides relevant insights into the function and evolution of two key features characterizing this species: The formation of IB and the adaptation to herbivory. By that our study lays the foundation for genetically optimizing *M. amblycephala* to further increase its relevance for securing human food supply.

Data description

Genome Assembly and Annotation

The *M. amblycephala* genome was sequenced and assembled by a whole-genome shotgun strategy using genomic DNA from a double-haploid fish (Additional file 1: Table S1). We assembled a 1.116 Gb reference genome sequence from 142.55 Gb (approximately 130-fold coverage) of clean data [16] (Additional file 1: Tables S1 and S2, Figure S2). The contig and scaffold N50 lengths reached 49 Kb and 839 Kb, respectively (Table 1). The largest scaffold spans 8,951 Kb and the 4,034 largest scaffolds cover 90% of the assembly. To assess the genome assembly quality, the mapping of paired end sequence data from the short-insert size WGS libraries, as well as of published ESTs [14] (Additional file 1: Tables S3 and S4) against the genome assembly indicated that the number and extent of misassemblies is low. To further estimate the completeness of the assembly and gene prediction, the benchmarking universal single-copy orthologs (BUSCO) [17] analysis was used and the results showed that the assembly contains 81.4% complete and 9.1% partial vertebrate BUSCO orthologues (Additional file 1: Table S5).

The *M. amblycephala* genome has an average GC content of 37.3%, similar to cyprinid *Cyprinus carpio* and *Danio rerio* (Additional file 1: Figures S3 and S4). Using a comprehensive annotation strategy combining RNA-seq derived transcript evidence, *de-novo* gene prediction and sequence similarity to proteins from five further fish species, we annotated a total of 23,696 protein-coding genes (Additional file 1: Table S6). Of the predicted genes, 99.44% (23,563 genes)

are annotated by functional database. In addition, we identified 1,796 non-coding RNAs including 474 miRNAs, 220 rRNA, 530 tRNAs, and 572 snRNAs. Transposable elements (TEs) comprise approximately 34.18% (381.3 Mb) of the *M. amblycephala* genome (Additional file 1: Table S7). DNA transposons (23.80%) and long terminal repeat retrotransposons (LTRs) (9.89%) are the most abundant TEs in *M. amblycephala*. The proportion of LTRs in *M. amblycephala* is highest in comparison with other teleosts: *G. morhua* (4.88%) [2], *L. crocea* (2.2%) [5], *C. semilaevis* (0.08%) [6], *C. carpio* (2.28%) [12], *C. idellus* (2.58%) [13] and stickleback (*Gasterosteus aculeatus*) (1.9%) [18] (Additional file 1: Tables S7 and S8, Figure S5). The distribution of divergence between the TEs in *M. amblycephala* peaks at only 7% (Additional file 1: Figure S6), indicating a more recent activity of these TEs when compared with *O. mykiss* (13%) [3] and *C. semilaevis* (9%) [6].

Anchoring Scaffolds and Shared Synteny Analysis

Sequencing data from 198 F1 specimens, including the parents, were used as the mapping population to anchor the scaffolds on to 24 psuedo-chromosomes of the *M. amblycephala* genome. Following RAD-Seq and sequencing protocol, 1883.5 Mb of 125-bp reads (on average 30.6 Mb and 9.3 Mb of read data for each parent and progeny, respectively) were generated on the HiSeq 2500 next-generation sequencing platform. Based on the SOAP bioinformatic pipeline, we generated 5,317 SNP markers for constructing a high-resolution genetic map. The map spans 1,701 cM with a mean marker distance of 0.33 cM and facilitated an anchoring of 1,434 scaffolds comprising 70% (779.54 Mb) of the *M. amblycephala* genome assembly to form 24 linkage groups (LG) (Additional file 1: Table S9). Of the anchored scaffolds, 598 could additionally be oriented (678.27 Mb, 87.01% of the total anchored sequences) (Figure 1A). A subsequent comparison of the gene order between *M. amblycephala* and its close relative *C.idellus* revealed 607 large shared syntenic blocks encompassing 11,259 genes, and 190 chromosomal rearrangements. The values change to 1,062 regions, 13,152 genes and 279 rearrangements when considering zebrafish (*Danio rerio*). The unexpected higher number of genes in syntenic regions shared with the more distantly related *D. rerio* is most likely an effect of the more complete genome assembly of this species compared to *C. idellus*. The rearrangement events are distributed across all *M. amblycephala* linkage groups without evidence for a local clustering (Figure 1B).

The most prominent event is a chromosomal fusion in *M. amblycephala* LG02 that joined two *D. rerio* chromosomes, Dre10 and Dre22. The same fusion is observed in *C. idellus* but not in *C. carpio* suggesting that it probably occurred in a last common ancestor of *M. amblycephala* and *C. idellus*, approximately 13.1 million years ago (Additional file 1: Figure S7).

Results

Evolutionary Analysis

A phylogenetic analysis of 316 single-copy genes with one to one orthologs in the genomes of 10 other fish species, and coelacanth (*Latimeria chalumnae*) and elephant shark (*Callorhinchus milii*), as out group served as a basis for investigating the evolutionary trajectory of *M. amblycephala* (Figure 2A, Additional file 1: Figure S8). To illuminate the evolutionary process resulting in the adaptation to a grass diet, we analyzed the functional properties of expanded gene families in the *M. amblycephala* and *C. idellus* lineage (Additional file 1: Figure S9, Additional file 2: Data Note1), two typical herbivores mainly feeding on aquatic and terrestrial grasses. Among the significantly over-represented KEGG pathways (Fisher's exact test, $P<0.01$), we find olfactory transduction (ko04740), immune-related pathways (ko04090, ko04672, ko04612 and ko04621), lipid metabolic related process (ko00590, ko03320, ko00591, ko00565, ko00592 and ko04975), as well as xenobiotics biodegradation and metabolism (ko00625 and ko00363) (Figure S10). These genes encoding proteins involved in biodegradation of xenobiotics would enhance the ability of an herbivore to detoxify the secondary compounds present in grasses that are adverse or even toxic to the organism. Furthermore, the high-fiber but low-energy grass diet requires a highly effective intermediate metabolism that accelerates carbohydrate and lipid catabolism and conversion into energy to maintain physiological functions. Indeed, when tracing positively selected genes (PSG) in *M. amblycephala* and *C. idellus* (Additional file 3: Date Note2), we identified many candidates involved in starch and sucrose metabolism (ko00500), citrate cycle (ko00020) and other types of O-glycan biosynthesis (ko00514). Moreover, 20 genes encoding enzymes involved in lipid and carbohydrate metabolism appear positively selected in both fish species (Additional file 1: Table S10).

Development of Intermuscular Bones

To explain the genetic basis of IB, their formation and their function in cyprinids, we first analyzed the functional annotation of gene families that expanded in this lineage (Figure 2C). Interestingly, many of these gene families are involved in cell adhesion (GO: 0007155, *P*=5.26E-32, 357 genes), myosin complex (GO:0016459, *P*=2.74E-08, 100 genes) and cell-matrix adhesion (GO:0007160, *P*=1.59E-21, 69 genes) (Figure 2C), which interact dynamically to mediate efficient cell motility, migration and muscle construction [19, 20].

As a second line of evidence, we performed transcriptome analyses of early developmental stages (stage1: whole larvae without IBs) and juvenile *M. amblycephala* (stage2: trunk muscle with partial IBs; stage3: trunk muscle with completed IBs) (Figure 3A). We found 249 genes significantly up-regulated in stages 2 and 3 (with IB) compared to stage 1 (no IB). Many of these genes belong to KEGG pathways involved in tight junction (ko04530), regulation of actin cytoskeleton (ko04810), cardiac muscle contraction (ko04260) and vascular smooth muscle contraction (ko04270) (Additional file 1: Figure S11). These genes are associated with cell motility and muscle contraction [19, 21, 22], which resembles the findings from the gene family expansion analysis. Specifically, some of these genes encoding proteins related to muscle contraction, including titin, troponin, myosin, actinin, calmodulin and other Ca^{2+} transporting ATPases (Figure 3A) point to a strong remodeling of the musculature compartment.

To confirm that the observed differences in gene expression are indeed linked to IB formation and function and are not simply due to the fact that different developmental stages were compared, we performed differential genes expression (DGE) analysis of muscle tissues, IB, and connective tissues from the same six months old individual of *M. amblycephala* (Figure 3B, Additional file 1: Figure S12). Among the genes that are significantly up-regulated in the IB samples many encode extracellular matrix (ECM) proteins (collagens and intergrin-binding protein), Rho GTPase family (*RhoA*, *Rho GAP*, *Rac*, *Ras*), motor proteins (myosin, dynein, actin), and calcium channel regulation protein (Additional file 1: Figure S13 and Table S11). Interestingly, it has been demonstrated that ECM proteins bound to integrins influence cell migration by actomyosin-generated contractile forces [20, 23]. Rho GTPases, acting as molecular switches, also is involved in regulating the actin cytoskeleton and cell migration, which in turn initiates intracellular signaling and contributes to tissue repair and regeneration [24-26].

During development of *M. amblycephala*, the first IB appears in muscles of caudal vertebrae as early as 28 days post fertilization (dpf) when body length is 12.95 mm (Additional file 1: Figure S14). The system then develops and ossifies predominantly from posterior to anterior (Additional file 1: Figure S15). IBs are present throughout the body within two months (Additional file 1: Figure S16) and develop into multiple morphological types in adults (Additional file 1: Figure S17). The bone is formed directly without an intermediate cartilaginous stage (Additional file 1: Figures S18 and S19). We also found a large number of mature osteoblasts distributed at the edge of the bone matrix while some osteocytes were apparent in the center of the mineralized bone matrix (Additional file 1: Figures S20 and S21). These primary bone-forming cells predominantly regulate bone formation and function throughout life. Notably, among the genes up-regulated in IB, 35 bone formation regulatory genes were identified (shown in colored boxes in Figure 3D). In particular, genes involved in bone morphogenetic protein (BMP) signaling including *Bmp3*, *Smad8*, *Smad9*, and *Id2*, in fibroblast growth factor (FGF) signaling including *Fgf2*, *Fgfr1a*, *Fgfbp2*, *Col6a3*, and *Col4a5*, and in Ca^{2+} channels including *Cacna1c*, *CaM*, *Creb5*, and *Nfatc* were highly expressed (>2-fold change) in IB (Additional file 1: Figure S22). It has been demonstrated that *Bmp*, *Fgf2*, and *Fgfr1* are involved in intramembranous bone development and affect the expression and activity of other osteogenesis related transcription factors [27, 28]. The calcium-sensitive transcription factor *NFATc1* together with *CREB* induces the expression of osteoclast-specific genes [29].

Adaptation to Herbivorous Diet

Next to the presence of IB, the herbivory of *M. amblycephala* is the second key feature in connection to the use of this species as aquatic livestock. Olfaction, the sense of smell, is crucial for animals to find food. The perception of smell is mediated by a large gene family of olfactory receptor (OR) genes. The ORs of teleosts are predominantly expressed in the main olfactory epithelium of the nasal cavity [30, 31] and can discriminate, like those of other vertebrates, different kinds of odor molecules. However, compared to mammals, e.g. humans having around 400 ORs [32] the OR repertoires in teleosts are considerably small. They range from only about 48 in *Fugu rubripes* up to 161 in *D. rerio* (Figure 4A). In the *M. amblycephala* genome, we

identified 179 functional olfactory receptor (OR) genes (Figure 4A), and based on the classification of Niimura [33], 158, 117 and 153 receptors for water-borne odorants were identified in *M. amblycephala*, *C. idellus* and *D. rerio*, respectively (Additional file 1: Table S12). Overall, these receptor repertoires are substantially larger than those of other and carnivorous teleosts (*G. morhua*, *C. semilaevis*, *O. latipes*, *X. maculatus*) (Additional file 1: Figures S23 and S24, Table S12). This suggests that olfaction—probably for food choice—has a particularly important role in the cyprinid species. Previous studies have demonstrated that the beta type OR genes are present in both aquatic and terrestrial vertebrates, indicating that the corresponding receptors detect both water-soluble and airborne odorants [31, 33]. Intriguingly, we found a massive expansion of beta-type OR genes in the genomes of the herbivorous *M. amblycephala* and *C. idellus*, while very few exist in other teleosts (Figure 4B, Additional file 1: Table S12).

Taste is also an important factor in the development of dietary habits. Most animals can perceive five basic tastes, namely sourness, sweetness, bitterness, saltiness and umami [34]. Interestingly, *T1R1*, the receptor gene necessary for sensing umami, has been lost in herbivorous *M. amblycephala* but is duplicated in carnivorous *G. morhua*, *C. semilaevis* and omnivorous *O. latipes* and *X. maculatus* (Figures 4C and 4D, Additional file 1: Figures S25-26 and Table S13). In contrast, *T1R2*, the receptor gene for sensing sweet, has been duplicated in herbivorous *M. amblycephala* and *C. idellus*, omnivorous *C. carpio* and *D. rerio*, while it has been lost in carnivorous *G. morhua* and *C. semilaevis* (Additional file 1: Figure S27 and Table S13). Bitterness sensed by the *T2R* is particularly crucial for animals to protect them from poisonous compounds [35]. Probably in the course switching to a diet that contains a larger fraction of bitterness containing food, also the *T2R* gene family in *M. amblycephala*, *C. idellus*, *C. carpio* and *D. rerio* has been expanded (Additional file 1: Figure S28).

To obtain further insights into the genetic adaptation to herbivorous diet, we focused on further genes that might be associated with digestion. Genes that encode proteases (including pepsin, trypsin, cathepsin and chymotrypsin) and amylases (including alpha-amylase and glucoamylase) were identified in the genomes of *M. amblycephala*, carnivorous *C. semilaevis*, *G. morhua* and omnivorous *D. rerio*, *O. latipes* and *X. maculatus*, indicating that herbivorous *M. amblycephala* has a protease repertoire that is not substantially different from those of carnivorous

and omnivorous fishes (Additional file 1: Table S14). We did not identify any genes encoding potentially cellulose-degrading enzymes including endoglucanase, exoglucanase and beta-glucosidase in the genome of *M. amblycephala*, suggesting that utilization of the herbivorous diet may largely depend on the gut microbiome. To elucidate this further, we determined the composition of the gut microbial communities of juvenile, domestic, wild adult *M. amblycephala* and wild adult *C. idellus* using bacterial 16S rRNA sequencing. A total of 549,020 filtered high quality sequence reads from 12 samples were clustered at a similarity level of 97%. The resulting 8,558 operational taxonomic units (OTUs) are dominated at phylum level by Proteobacteria, Fusobacteria, Bacteroidetes, Firmicutes and Actinobacteria (Additional file 1: Table S15, Figure 4E). Increasing the resolution to the genus level, the composition and relative abundance of the gut microbiota of wild adult *M. amblycephala* and *C. idellus* are still very similar (Additional file 1: Table S16) and we could identify more than 7% cellulose-degrading bacteria (Additional file 1: Table S17). This indicates that indeed the gut microbiome is crucial in the digestion of plant material, and thus in the adaptation to herbivory.

Discussion

M. amblycephala is the economically most important species for freshwater aquaculture. In addition to its various superior properties, especially its herbivorous diet, it is also an excellent model to study IB formation. Here we make available draft genome of *M. amblycephala* with more than 70% of genome data anchored on 24 linkage groups. Comparative analyses of genome structure revealed high synteny with three other cyprinid fish and uncovered a chromosomal fusion event in *M. amblycephala* that joined two *D. rerio* chromosomes (Figure 1B), which supports the previous results in *C. idellus* [13] and also provides novel scientific insights into the evolution of chromosome fusion events in cyprinids.

The evolutionary trajectory analysis of *M. amblycephala* and other teleosts revealed that *M. amblycephala* has the closest relationship to *C. idellus* (Figure 2A). Both the species are herbivorous fish but which endogenous and exogenous factors affected their feeding habits and how they adapted to their herbivorous diet is not known. Olfaction and taste are crucial for animals to find food and to distinguish whether potential food is edible or harmful [31, 35]. The

search for genes encoding OR showed that herbivorous *M. amblycephala* and *C. idellus* have a large number of beta-type OR, while other omnivorous and carnivorous fish only have one or two. This might be attributed to their particular herbivorous diet consisting not only of aquatic grasses but also the duckweed and terrestrial grasses, which they ingest from the water surface. Previous studies have demonstrated that the receptor for umami is formed by the T1R1/T1R3 heterodimer, while T1R2/T1R3 senses sweet taste [36]. We found that the umami gene *T1R1* was lost in herbivorous *M. amblycephala* but duplicated in the carnivorous *G. morhua* and *C. semilaevis* (Figure 4C). The loss of the *T1R1* gene in *M. amblycephala* might exclude the expression of a functional umami taste receptor. Such situations in other organism, e.g. the Chinese panda, have previously been related to feeding specialization [37]. Interestingly, the sweetness receptor *T1R2* and bitter receptor *T2R* genes are expanded in the herbivorous fish but few or no copy was found in carnivorous fish. Collectively, these results not only indicate the genetic adaptation to herbivorous diet of *M. amblycephala*, but also provided a clear and comprehensive picture of adaptive evolutionary mechanisms of sensory systems in other fish species with different trophic specializations.

Some insects such as *Tenebrio molitor* [38] and *Neotermes koshunensis* [39], and the mollusc *Corbicula japonica* [40] have genes encoding endogenous cellulose degradation-related enzymes. However, all so far analyzed herbivorous vertebrates lack these genes and always rely on their gut microbiome to digest food [37, 41]. In herbivorous *M. amblycephala* and *C. idellus*, we also did not find any homologues of digestive cellulase genes. Interestingly, our work on the composition of gut microbiota of the two fish species identifies more than 7% cellulose-degrading bacteria, suggesting that the cellulose degradation of herbivorous fish largely depend on their gut microbiome.

IB has evolved several times during teleost evolution [9, 42]. The developmental mechanisms and ossification processes forming IBs are dramatically distinct from other bones such as ribs, skeleton, vertebrae or spines. These usually develop from cartilaginous bone and are derived from the mesenchymal cell population by endochondral ossification [27, 43]. However, IBs form directly by intramembranous ossification and differentiate from osteoblasts within connective tissue, forming segmental, serially homologous ossifications in the myosepta. Although various

methods of ossification of IB have been proposed, few experiments have been conducted to confirm the ossification process and little is known about the potential role of IB in teleosts. Based on our findings of over-represented functional properties of expanded gene families in cyprinid lineage (Figure 2C) and evidence from DGE of early developmental stages of IB formation (Figure 3A), we provide molecular evidence that IB might play significant roles not only in regulating muscle contraction but also in active remodeling at the bone-muscle interface and coordination of cellular events.

It has previously been found that some major developmental signals including BMP, FGF, WNT, together with calcium/calmodulin signaling [27, 44-46], are essential for regulating the differentiation and function of osteoblasts and osteocytes and for regulating the RANKL signaling pathway for osteoclasts [47] in intramembranous bone development. In agreement with this concept, our DGE analysis of muscle, IB and connective tissues uncovered that 35 bone formation regulatory genes involved in these signals were highly up-regulated in IB. Taken together, these results suggest that IB indeed undergoes an intramembranous ossification process, is regulated by bone-specific signaling pathways, and underlies a homeostasis of maintenance, repair and remodeling.

Conclusions

Our results provide novel functional insights into the evolution of cyprinids. Importantly, the *M. amblycephala* genome data come up with novel insights shedding light on the adaptation to herbivorous nutrition and evolution and formation of IB. Our results on the evolution of gene families, digestive and sensory system, as well as our microbiome meta-analysis and transcriptome data provide powerful evidence and a key database for future investigations to increase the understanding of the specific characteristics of *M. amblycephala* and other fish species.

Methods

Sampling and DNA Extraction

DNA for genome sequencing was derived from a double haploid fish from the *M. amblycephala* genetic breeding center at Huazhong Agricultural University (Wuhan, Hubei, China). Fish blood

was collected from adult female fish caudal vein using sterile injectors with pre-added anticoagulant solutions following anesthetized with MS-222 and sterilization with 75% alcohol. Genomic DNA was extracted from the whole blood.

Genomic Sequencing and Assembly

Libraries with different insert sized inserts of 170 bp, 500 bp, 800 bp, 2 Kb, 5 Kb, 10 Kb and 20 Kb were constructed from the genomic DNA at BGI-Shenzhen. The libraries were sequenced using a HiSeq2000 instrument. In total, 11 libraries, sequenced in 23 lanes were constructed. To obtain high quality data, we applied filtering criteria for the raw reads. As a result, 142.55 Gb of filtered data were used to complete the genome assembly using SOAPdenovo_V2.04 [16]. Only filtered data were used in the genome assembly. First, the short insert size library data were used to construct a de Bruijn graph. The tips, merged bubbles and connections with low coverage were removed before resolving the small repeats. Second, all high-quality reads were realigned with the contig sequences. The number of shared paired-end relationships between pairs of contigs was calculated and weighted with the rate of consistent and conflicting paired ends before constructing the scaffolds in a stepwise manner from the short–insert size paired ends to the long–insert size paired ends. Third, the gaps between the constructed scaffolds were composed mainly of repeats, which were masked during scaffold construction. These gaps were closed using the paired-end information to retrieve read pairs in which one end mapped to a unique contig and the other was located in the gap region. Subsequently, local assembly was conducted for these collected reads. To assess the genome assembly quality, approximately 42.82 Gb Illumina reads generated from short-insert size libraries were mapped onto the genome. Bwa0.5.9-r16 software [48] with default parameters was used to assess the mapping ratio and Soap coverage 2.27 was used to calculate the sequencing depth. We also assessed the accuracy of the genome assembly by Trinity [49], including number of ESTs and new mRNA reads from early stages of embryos and multiple tissues, by aligning the scaffolds to the assembled transcriptome sequences.

After obtaining K-mers from the short-insert-size (<1Kb) reads with just one bp slide, frequencies of each K-mer were calculated. The K-mer frequency fits Poisson distribution when a sufficient amount of data is present. The total genome size was deduced from these data in the following way: Genome size = K-mer num / Peak_depth.

Genome Annotation

The genome was searched for repetitive elements using Tandem Repeats Finder (version 4.04) [50]. TEs were identified using homology-based approaches. The Repbase (version 16.10) [51] database of known repeats and a *de novo* repeat library generated by RepeatModeler were used. This database was mapped using the software of RepeatMasker (version 3.3.0). Four types of non-coding RNAs (microRNAs, transfer RNAs, ribosomal RNAs and small nuclear RNAs) were also annotated using tRNAscan-SE (version 1.23) and the Rfam database45 (Release 9.1) [52].

For gene prediction, *de novo* gene prediction, homology-based methods and RNA-seq data were used to perform gene prediction. For the sequence similarity based prediction, *D. rerio, G. aculeatus, O. niloticus, O. latipes* and *G. morhua* protein sequences were downloaded from Ensembl (release 73) and were aligned to the *M. amblycephala* genome using TBLASTN. Then homologous genome sequences were aligned against the matching proteins using GeneWise [53] to define gene models. Augustus was employed to predict coding genes using appropriate parameters in *de novo* prediction. For the RNA-seq based prediction, we mapped transcriptome reads to the genome assembly using TopHat [54]. Then, we combined TopHat mapping results together and applied Cufflinks [55] to predict transcript structures. All predicted gene structures were integrated by GLEAN [56] (http://sourceforge.net/projects/glean-gene/) to obtain a consensus gene set. Gene functions were assigned to the translated protein-coding genes using Blastp tool, based on their highest match to proteins in the SwissProt and TrEMBL [57] databases (Uniprot release 2011-01). Motifs and domains in the protein-coding genes were determined by InterProScan (version 4.7) searches against six different protein databases: ProDom, PRINTS, Pfam, SMART, PANTHER and PROSITE. Gene Ontology [58] IDs for each gene were obtained from the corresponding InterPro entries. All genes were aligned against KEGG [59] (Release 58) database, and the pathway in which the gene might be involved was derived from the matched genes in KEGG. tRNA genes were *de novo* predicted by tRNAscan-SE software [60], with eukaryote parameters on the repeat pre-masked genome. The rRNA fragments were identified by aligning the rRNA sequences using BlastN at E-value 1e-5. The snRNA and miRNA were searched by the method of aligning and searching with INFERNAL (version 0.81) [61] against Rfam database (release 9.1).

Genetic map construction

To anchor the scaffolds into pseudo-chromosomes, 198 F1 population individuals were used to obtain the genetic map. Each of the individual genomic DNA was digested with the restriction endonuclease EcoR I, following the RAD-Seq protocol [62]. The SNP calling process was carried out using the SOAP bioinformatic pipeline. The RAD-based SNP calling was done by SOAPsnp software [63] after each individual's paired-end RAD reads was mapped onto the assembled reference genome with the alignment software SOAP2 [64]. The potential SNP markers were used for the linkage analysis if the following criteria were satisfied: for parents - sequencing depth ≥ 8 and ≤ 100, base quality ≥ 25, copy number ≤ 1.5; for progeny - sequencing depth ≥ 5, base quality ≥ 20, copy number ≤ 1.5. If the markers were showing significantly distorted segregation (P-value < 0.01), they were excluded from the map construction. Linkage analysis was performed only for markers present in at least 80% of the genomes, using JoinMap 4.0 software with CP population type codes and applying the double pseudo-test cross strategy [65]. The linkage groups were formed at a logarithm of odds threshold of 6.0 and ordered using the regression mapping algorithm.

Construction of gene families

We identified gene families using TreeFam software [66] as follows: Blast was used to compare all the protein sequences from 13 species: *M. amblycephala*, *C. idellus*, *C. semilaevis*, *C. carpio*, *D. rerio*, *Callorhinchus milii*, *G. morhua*, *G. aculeatus*, *Latimeria chalumnae*, *Oncorhynchus mykiss*, *O. niloticus*, *O. latipes*, *Fugu rubripes*, with the E-value threshold set as 1e-7. In the next step, HSP segments of each protein pair were concatenated by Solar software. H-scores were computed based on Bit-scores and these were taken to evaluate the similarity among genes. Finally, gene families were obtained by clustering of homologous gene sequences using Hcluster_sg (Version 0.5.0). Specific genes of *M. amblycephala* were those that did not cluster with other vertebrates that were chosen for gene family construction, and those that did not have homologs in the predicted gene repertoire of the compared genomes. If these genes had functional motifs, they were annotated by GO.

Phylogenetic Tree Reconstruction and Divergence Time Estimation

The coding sequences of single-copy gene families conserved among *M. amblycephala*, *C. idellus*,

C. carpio, D. rerio, C. semilaevis, G. morhua, G. aculeatus, Latimeria chalumnae, O. mykiss, O. niloticus, O. latipes, C. milii and *Fugu rubripes* (Ensembl Gene v.77) were extracted and aligned with guidance from amino-acid alignments created by the MUSCLE program [67]. The individual sequence alignments were then concatenated to form one supermatrix. PhyML [68, 69] was applied to construct the phylogenetic tree under an HKY85+gamma model for nucleotide sequences. ALRT values were taken to assess the branch reliability in PhyML. The same set of codon sequences at position 2 was used for phylogenetic tree construction and estimation of the divergence time. The PAML mcmctree program (PAML version 4.5) [70, 71] was used to determine divergence times with the approximate likelihood calculation method and the 'correlated molecular clock' and 'REV' substitution model.

Gene Family Expansion and Contraction Analysis

Protein sequences of *M. amblycephala* and 11 other related species (Ensembl Gene v.77)) were used in BLAST searches to identify homologs. We identified gene families using CAFÉ [72], which employs a random birth and death model to study gene gains and losses in gene families across a user-specified phylogeny. The global parameter λ, which describes both the gene birth (λ) and death ($\mu = -\lambda$) rate across all branches in the tree for all gene families, was estimated using maximum likelihood. A conditional *P*-value was calculated for each gene family, and families with conditional *P*-values less than the threshold (0.05) were considered as having notable gain or loss. We identified branches responsible for low overall *P*-values of significant families.

Detection of Positively Selected Genes

We calculated Ka/Ks ratios for all single copy orthologs of *M. amblycephala* and *C. semilaevis, D. rerio, G. morhua, O. niloticus and C. carpio*. Alignment quality was essential for estimating positive selection. Thus, orthologous genes were first aligned by PRANK [73], which is considerably conservative for inferring positive selection. We used Gblocks [74] to remove ambiguously aligned blocks within PRANK alignments and employed ‘codeml’ in the PAML package with the free-ratio model to estimate Ka, Ks, and Ka/Ks ratios on different branches. The differences in mean Ka/Ks ratios for single-copy genes between *M. amblycephala* and each of the other species were compared using paired Wilcoxon rank sum tests. Genes that showed values of Ka/Ks higher than 1 along the branch leading to *M. amblycephala* were reanalyzed using the

codon based branch-site tests implemented in PAML. The branch-site model allowed ω to vary both among sites in the protein and across branches, and was used to detect episodic positive selection.

Developmental process of intermuscular bone in *M. amblycephala*

To better understand the number and morphological types of IBs in adult *M. amblycephala*, specimens with a body length ranging from 15.5 to 20.5 cm were collected and each individual was wrapped in gauze and boiled. The fish body was divided into two sections: anterior (snout to cloaca) and posterior (cloaca to the base of caudal fin), and the length of each section was measured. The IBs were retrieved, counted, arranged in order and photographed with a digital camera. Fertilized *M. amblycephala* eggs were brought from hatching facilities at Freshwater Fish Genetics Breeding Center of Huazhong Agricultural University (Wuhan, Hubei, China) to our laboratory. *M. amblycephala* larvae were maintained in a re-circulating aquaculture system at 23 ± 1°C with a 14-hr photoperiod. To explore the early development of IBs, larvae at different stages from 15 to 40 dpf were collected and fixed in 4% paraformaldehyde and transferred to 70% ethanol for storage. Specimens were stained with alizarin red for bone following the method described by Dawson [75]. The appearance of red color was recorded as the appearance of IB because bone ossification is accompanied by the uptake of alizarin red, resulting in red staining of the mineralized bone matrix. Myosepta, either not yet ossified, or poorly ossified, are not visible with alizarin red staining. For histologic analysis, specimens were paraffin-embedded and sectioned following standard protocols. Sections were stained with hematoxylin and eosin (HE) and Masson trichrome [76] and photographed using a Nikon microscope (Nikon, Tokyo, Japan) with a DP70 digital camera (Olympus, Japan). Scanning electron microscopy (SEM) and transmission electron microscopy (TEM) were also conducted to analyze the ultrastructure of IB. The specimens were fixed with 2.5% (v/v) glutaraldehyde in a solution of 0.1 M sodium cacodylate buffer (pH 7.3) for 2 h at room temperature. The SEM and TEM samples were prepared according to a standard protocol described by Ott [77]. The samples were then visualized with a JSM-6390LV scanning electron microscope (SEM, Japan) and the stained ultrathin sections with a H-7650 transmission electron microscope (Hitachi, Japan).

RNA Sequencing Analysis

M. amblycephala specimens belonging to three different developmental stages of IBs (stage 1: whole larvae without distribution of IB; stage 2: muscle tissues with partial distribution of IBs; stage 3: muscle tissues with completed distribution of IBs were identified under microscope and immediately frozen in liquid nitrogen. In addition, dorsal white muscle, IBs and connective tissue surrounding the IBs from six months old fish were also collected. RNA was extracted from total fish samples at different stages and from individual muscle, connective tissue, and intermuscular bone samples of *M. amblycephala* using RNAisoPlus Reagent (TaKaRa, China) according to the manufacturer's protocol. The integrity and purity of the RNA was determined by gel electrophoresis and Agilent 2100 BioAnalyzer (Agilent, USA) before preparing the libraries for sequencing. Paired-end RNA sequencing was performed using the Illumina HiSeq 2000 platform. Low quality score reads were filtered and the clean data were aligned to the reference genome using Bowtie [78]. Genes and isoforms expression level were quantified by a software package: RSEM (RNASeq by Expectation Maximization) [79]. Gene expression levels were calculated by using the RPKM method (Reads per kilobase transcriptome per million mapped reads) [80] and adjusted by a scaling normalization method [81]. DEGs were detected using DESeq [82]. Annotation of DEGs were mapped to GO categories in the database (http://www.geneontology.org/) and the number of genes for every term were calculated to identify GO terms that were significantly enriched in the input list of DEGs. The calculated *P*-value was adjusted by the Bonferroni Correction, taking corrected *P*-value ≤ 0.05 as a threshold. KEGG automatic annotation was used to perform pathway enrichment analysis of DEGs.

Prediction of Olfactory Receptor Genes

Olfactory receptor genes were identified by previously described methods [83], with the exception of a first-round TBLASTN [84] search, in which 1,417 functional olfactory receptor genes from *H. sapiens*, *D. rerio*, *L. chalumnae*, *Lepisosteus oculatus*, *L. vexillifer*, *O. niloticus*, *O. latipes*, *F. rubripes* and *Xenopus tropicalis* were used as queries. We then predicted the structure of sequenced genes using the blast-hit sequence with the software GeneWise extending in both 3' and 5' directions along the genome sequences. The results were further confirmed by NR annotation. Then the coding sequences from the start (ATG) to stop codons were extracted, while sequences that contained interrupting stop codons or frame-shifts were regarded as pseudogenes. To

construct phylogenetic trees, the amino-acid sequences encoded by olfactory receptor genes were first aligned using the program MUSCLE nested in MEGA 5.10 [85]. We then constructed the phylogenetic tree using the neighbor-joining method with Poisson correction distances using the program MEGA 5.10. We also identified and compared the genes for five basic tastes (sour, sweet, bitter, umami and salty) using a similar method as in OR gene identification.

Gut microbiota analysis

To characterize the microbial diversity of herbivorous *M. amblycephala*, a total of 12 juvenile (LBSB), domestic adult (DBSB), wild adult *M. amblycephala* (BSB) and wild adult *C. idellus* (GC) intestinal fecal samples were collected. Bacterial genomic DNA was extracted from 200 mg gut content of each sample using a QIAamp DNA Stool Mini Kit (Qiagen, Valencia, USA). Quality and integrity of each DNA sample were determined by 1% agarose gel electrophoresis in Tris-acetate-EDTA (TAE) buffer. DNA concentration was quantified using NanoDrop ND-2000 spectrophotometer (Thermo Scientific). To determine the diversity and composition of the bacterial communities of each sample, a total of 20 μg of genomic DNA were sequenced using the Illumina MiSeq sequencing platform. PCR amplifications were conducted from each sample to produce the V4 hypervariable region (515F and 806 R) of the 16S rRNA gene according to the previously described method [86]. We used the UPARSE pipeline [87] to pick operational taxonomic units (OTUs) at an identity threshold of 97% and picked representative sequences for each OTU and used the RDP classifier to assign taxonomic data to each representative sequence.

Additional files

Additional file 1: Tables S1 to S17 and Figures S1 to S28.

Additional file 2: Data Note1 Expanded genes in the *M. amblycephala* and *C. idellus* lineage.

Additional file 3: Data Note2 Positively selected genes in the *M. amblycephala* and *C. idellus* genomes.

Abbreviations

IB, intermuscular bone; SNP, single-nucleotide polymorphism; BUSCO, benchmarking universal single-copy orthologs; TE, transposable element; LTR, long terminal repeat retrotransposon; LG, linkage group; PSG, positively selected gene; ECM, extracellular matrix; dpf, days post fertilization; BMP, bone morphogenetic protein; FGF, fibroblast growth factor; OR, olfactory

receptor; OTU, operational taxonomic unit; DGE, differential genes expression; HE, hematoxylin and eosin; SEM, scanning electron microscopy; TEM, transmission electron microscopy

Acknowledgements

This work was supported by the Modern Agriculture Industry Technology System Construction Projects of China titled as—Staple Freshwater Fishes Industry Technology System (No. CARS-46-05), Guangdong Haid Group Co., Ltd, the Fundament Research Funds for the Central Universities (2662015PY019), the International Scientific and Technology Cooperation Program of Wuhan City (2015030809020365).

Availability of data and materials

Datasets supporting the results of this article are available in the GigaDB repository associated with this publication [88]. Raw whole genome sequencing, transcriptome and RAD-Seq data have been deposited at NCBI in the SRA under bioproject number PRJNA343584.

Authors' contributions

W.W. initiated and conceived the project and provided scientific input. X.Q. organized financial support and designed the project. M.S. discussed the data, wrote and modified the paper. H.L. and C.C. conducted the biological experiments, analyzed the data and wrote the paper with input from other authors. I.E. wrote and modified the manuscript and discussed the data. The RAD-Seq data analyses and the genetic map construction were performed by Z.G., Y.G., J.J. and X.J. Genome assembly and annotation were performed by J.M., H.C., M.X. and J.C. X.Z., W.L., R.L., B.C., J.W., H.L., S.Y., H.W., X.C., X.Z., Y.Z., K.W., R.Y. and B. L. carried out the samples preparation and data collection. J.L. and J.C. identified the gene families and analyzed the RNA-seq data. M.B. coordinated the project. S.Z. and X.F. modified the manuscript and discussed the data. All authors read the manuscript and provided comments and suggestions for improvements. The authors declare no competing financial interests.

Competing interests

The authors declare that they have no competing interests.

Ethics approval and consent to participate

All experimental procedures involving fish were performed in accordance with the guidelines and regulations of the National Institute of Health Guide for the Care and Use of Laboratory Animals and the Institutional Review Board on Bioethics and Biosafety of BGI (BGI-IRB).

References

1. FAO Fisheries and Aquaculture Department. FAO yearbook Fishery and Aquaculture Statistics 2014 (Food and Agriculture Organization of the United Nations, Rome, 2016).

2. Star B, Nederbragt AJ, Jentoft S, Grimholt U, Malmstrøm M, Gregers TF, et al. The genome sequence of Atlantic cod reveals a unique immune system. Nature. 2011; 477:207–10.

3. Berthelot C, Brunet F, Chalopin D, Juanchich A, Bernard M, Noël B, et al. The rainbow trout genome provides novel insights into evolution after whole-genome duplication in vertebrates. Nat. Commun. 2014; 5:3657.

4. Tine M., Kuhl H., Gagnaire PA, Louro B, Desmarais E, Martins RS, et al. European sea bass genome and its variation provide insights into adaptation to euryhalinity and speciation. Nat. Commun. 2014; 5:5770.

5. Wu C, Zhang D, Kan M, Lv Z, Zhu A, Su Y, et al. The draft genome of the large yellow croaker reveals well-developed innate immunity. Nat. Commun. 2014; 5:5227.

6. Chen S, Zhang G, Shao C, Huang Q, Liu G, Zhang P, et al. Whole-genome sequence of a flatfish provides insights into ZW sex chromosome evolution and adaptation to a benthic lifestyle. Nat. Genet. 2014; 46:253–60.

7. Brawand D, Wagner CE, Li YI, Malinsky M, Keller I, Fan S, et al. The genomic substrate for adaptive radiation in African cichlid fish. Nature. 2014; 513:375–82.

8. Chen X, Zhong L, Bian C, Xu P, Qiu Y, You X, et al. High-quality genome assembly of channel catfish, *Ictalurus punctatus*. GigaScience. 2016; 5:39.

9. Gemballa S, Britz R. Homology of intermuscular bones in acanthomorph fishes. Am. Mus. Novit. 1998; 3241:1–25.

10. Danos N, Ward AB. The homology and origins of intermuscular bones in fishes: Phylogenetic or biomechanical determinants? Biol. J. Linn. Soc. 2012; 106:607–22.

11. Wan SM, Yi SK, Zhong J, Nie CH, Guan NN, Zhang WZ, et al. Dynamic mRNA and miRNA expression analysis in response to intermuscular bone development of blunt snout bream (*Megalobrama amblycephala*). Sci. Rep. 2016; 6:31050.

12. Xu P, Zhang X, Wang X, Li J, Liu G, Kuang Y, et al. Genome sequence and genetic diversity of the common carp, *Cyprinus carpio*. Nat. Genet. 2014; 46:1212–9.

13. Wang Y, Lu Y, Zhang Y, Ning Z, Li Y, Zhao Q, et al. The draft genome of the grass carp (*Ctenopharyngodon idellus*) provides insights into its evolution and vegetarian adaptation. Nat. Genet. 2015; 47:625–31.

14. Gao Z, Luo W, Liu H, Zeng C, Liu X, Yi S, et al. Transcriptome analysis and SSR/SNP markers information of the blunt snout bream (*Megalobrama amblycephala*). PLoS One. 2012; 7:e42637.

15. Yi S, Gao ZX, Zhao H, Zeng C, Luo W, Chen B, et al. Identification and characterization of microRNAs involved in growth of blunt snout bream (*Megalobrama amblycephala*) by Solexa sequencing. BMC Genomics. 2013; 14:754.

16. Luo R, Liu B, Xie Y, Li Z, Huang W, Yuan J, et al. SOAPdenovo2: an empirically improved memory-efficient short-read de novo assembler. Gigascience. 2012;1:18.

17. Simao FA, Waterhouse RM, Ioannidis P, Kriventseva EV, Zdobnov EM. BUSCO: assessing genome assembly and annotation completeness with single-copy orthologs. Bioinformatics. 2015;31:3210–2.

18. Jones FC, Grabherr MG, Chan YF, Russell P, Mauceli E, Johnson J, et al. The genomic basis of adaptive evolution in threespine sticklebacks. Nature. 2012; 484:55–61.

19. Even-Ram S, Doyle AD, Conti MA, Matsumoto K, Adelstein RS, Yamada KM. Myosin IIA regulates cell motility and actomyosin-microtubule crosstalk. Nat. Cell Biol. 2007; 9:299–309.

20. Sheetz MP, Felsenfeld DP, Galbraith CG. Cell migration: Regulation of force on extracellular-complexes. Trends Cell Biol. 1998; 8:51–4.

21. Gunst SJ, Zhang W. Actin cytoskeletal dynamics in smooth muscle: a new paradigm for the regulation of smooth muscle contraction. Am. J. Physiol. Cell Physiol. 2008; 295:C576–87.

22. Webb RC. Smooth muscle contraction and relaxation. Adv. Physiol. Educ. 2003; 27:201–6.

23. Ulrich TA, De Juan Pardo EM, Kumar S. The mechanical rigidity of the extracellular matrix regulates the structure, motility, and proliferation of glioma cells. Cancer Res. 2009; 69:4167–74.

24. Ridley AJ. Rho GTPases and cell migration. J. Cell Sci. 2001; 114:2713–22.

25. Etienne-Manneville S, Hall A. Rho GTPases in Cell Biology. Nature. 2002; 420:629–35.

26. Ridley AJ. Cell Migration: Integrating Signals from Front to Back. Science. 2003; 302:1704–9.

27. Ornitz D, Marie P. FGF signaling pathways in endochondral and intramembranous bone development and human genetic disease. Genes Dev. 2002; 16:1446–65.

28. Fakhry A, Ratisoontorn C, Vedhachalam C, Salhab I, Koyama E, Leboy P, et al. Effects of FGF-2/-9 in calvarial bone cell cultures: Differentiation stage-dependent mitogenic effect, inverse regulation of BMP-2 and noggin, and enhancement of osteogenic potential. Bone. 2005; 36:254–66.

29. Sato K, Suematsu A, Nakashima T, Takemoto-Kimura S, Aoki K, Morishita Y, et al. Regulation of osteoclast differentiation and function by the CaMK-CREB pathway. Nat. Med. 2006; 12:1410–6.

30. Niimura Y, Nei M. Evolutionary dynamics of olfactory and other chemosensory receptor genes in vertebrates. J. Hum. Genet. 2006; 51:505–17.

31. Nei M, Niimura Y, Nozawa M. The evolution of animal chemosensory receptor gene repertoires: roles of chance and necessity. Nat. Rev. Genet. 2008; 9:951–63.

32. Lopez C, Raper J. Cloning and functional characterization of odorant receptors expressed in the zebrafish olfactory system. FASEB J. 2015; 29:727–37.

33. Niimura Y. On the origin and evolution of vertebrate olfactory receptor genes: comparative genome analysis among 23 chordate species. Genome Biol. Evol. 2009; 1:34–44.

34. Lindemann B. Receptors and transduction in taste. Nature. 2001; 413:219–25.

35. Chandrashekar J, Mueller KL, Hoon MA, Adler E, Feng L, Guo W, et al. T2Rs function as bitter taste receptors. Cell. 2000; 100:703–11.

36. Nelson G, Chandrashekar J, Hoon MA, Feng L, Zhao G, Ryba NJ, et al. An amino-acid taste receptor. Nature. 2002; 416:199–202.

37. Li R, Fan W, TianG, Zhu H, He L, Cai J, et al. The sequence and *de novo* assembly of the giant panda genome. Nature.2010; 463:311–7.

38. Ferreira AHP, Marana SR, Terra WR, Ferreira C. Purification, molecular cloning, and properties of a β-glycosidase isolated from midgut lumen of *Tenebrio molitor* (Coleoptera) larvae. Insect Biochem. Mol. Biol. 2001; 31:1065–76.

39. Tokuda G, Saito H, Watanabe H. A digestive β-glucosidase from the salivary glands of the termite, *Neotermes koshunensis* (Shiraki): Distribution, characterization and isolation of its precursor cDNA by 5'- and 3'-RACE amplifications with degenerate primers. Insect Biochem. Mol. Biol. 2002; 32:1681–9.

40. Sakamoto K, Uji S, Kurokawa T, Toyohara H. Molecular cloning of endogenous β-glucosidase from common Japanese brackish water clam *Corbicula japonica*. Gene. 2009; 435:72–9.

41. Zhu L, Wu Q, Dai J, Zhang S, Wei F. Evidence of cellulose metabolism by the giant panda gut microbiome. Proc. Natl. Acad. Sci. USA. 2011; 108:17714–9.

42. Bird NC, Mabee PM. Developmental morphology of the axial skeleton of the zebrafish, *Danio rerio* (Ostariophysi: Cyprinidae). Dev. Dyn. 2003; 228:337–57.

43. Ortega N, Behonick DJ, Werb Z. Matrix remodeling during endochondral ossification. Trends Cell Biol. 2004; 14:86–93.

44. Chen G, Deng C, Li YP. TGF-β and BMP signaling in osteoblast differentiation and bone formation. Int. J. Biol. Sci. 2012; 8:272–88.

45. Harada S, Rodan GA. Control of osteoblast function and regulation of bone mass. Nature. 2003; 423:349–55.

46. Long F. Building strong bones: molecular regulation of the osteoblast lineage. Nat. Rev. Mol. Cell Biol. 2011; 13:27–38.

47. Boyle WJ, Simonet WS, Lacey DL. Osteoclast differentiation and activation. Nature. 2003; 423:337–42.

48. Li H, Durbin R. Fast and accurate short read alignment with Burrows-Wheeler transform. Bioinformatics. 2009; 25:1754–60.

49. Grabherr MG, Haas BJ, Yassour M, Levin JZ, Thompson DA, Amit I, et al. Trinity: reconstructing a full-length transcriptome without a genome from RNA-Seq data . Nat. Biotechnol. 2011; 29:644–52.

50. Benson G. Tandem repeats finder: A program to analyze DNA sequences. Nucleic Acids Res. 1999; 27:573–80.

51. Jurka J, Kapitonov VV, Pavlicek A, Klonowski P, Kohany O, Walichiewicz J. Repbase Update, a database of eukaryotic repetitive elements. Cytogenet. Genome Res. 2005; 110:462–7.

52. Griffiths-Jones S, Moxon S, Marshall M, Khanna A, Eddy SR, Bateman A. Rfam: Annotating non-coding RNAs in complete genomes. Nucleic Acids Res. 2005; 33:121–4.

53. Birney E, Clamp M, Durbin R. GeneWise and Genomewise. Genome Res. 2004; 14:988–95.

54. Trapnell C, Pachter L, Salzberg SL. TopHat: Discovering splice junctions with RNA-Seq. Bioinformatics. 2009; 25:1105–11.

55. Trapnell C, Williams BA, Pertea G, Mortazavi A, Kwan G, Van Baren MJ, et al. Transcript assembly and quantification by RNA-Seq reveals unannotated transcripts and isoform switching during cell differentiation. Nat. Biotechnol. 2010; 28:511–5.

56. Elsik CG, Mackey AJ, Reese JT, Milshina NV, Roos DS, Weinstock GM. Creating a honey bee consensus gene set. Genome Biol. 2007; 8:R13.

57. Bairoch A, Apweiler R. The SWISS-PROT protein sequence database and its supplement TrEMBL in 2000. Nucleic Acids Res. 2000; 28:45–8.

58. Ashburner M, Ball CA, Blake JA, Botstein D, Butler H, Cherry JM, et al. Gene ontology: tool for the unification of biology. Nat. Genet. 2000; 25:25–9.

59. Kanehisa M, Goto S. KEGG: Kyoto encyclopedia of genes and genomes. Nucleic Acids Res. 2000; 28:27-30.

60. Lowe TM, Eddy SR. tRNAscan-SE: A program for improved detection of transfer RNA genes in genomic sequence. Nucleic Acids Res. 1997; 25:955–64.

61. Nawrocki EP, Kolbe DL, Eddy SR. Infernal 1.0: Inference of RNA alignments. Bioinformatics. 2009; 25:1335–7.

62. Baird NA, Etter PD, Atwood TS, Currey MC, Shiver AL, Lewis ZA, et al. Rapid SNP discovery and genetic mapping using sequenced RAD markers. PloS One. 2008; 3:e3376.

63. Li R, Li Y, Fang X, Yang H, Wang J, Kristiansen K, et al. SNP detection for massively parallel whole-genome resequencing. Genome Res. 2009; 19:1124–32.

64. Li R, Yu C, Li Y, Lam TW, Yiu SM, Kristiansen K, et al. SOAP2: An improved ultrafast tool for short read alignment. Bioinformatics. 2009; 25:1966–7.

65. Grattapaglia D, Sederoff R. Genetic linkage maps of Eucalyptus grandis and Eucalyptus urophylla using a pseudo-testcross: Mapping strategy and RAPD markers. Genetics. 1994; 137:1121–37.

66. Li H, Coghlan A, Ruan J, Coin LJ, Heriche JK, Osmotherly L, et al. TreeFam: a curated database of phylogenetic trees of animal gene families. Nucleic Acids Res. 2006; 34:D572–80.

67. Edgar RC. MUSCLE: Multiple sequence alignment with high accuracy and high throughput. Nucleic Acids Res. 2004; 32:1792–7.

68. Guindon S, Dufayard JF, Lefort V, Anisimova M, Hordijk W, Gascuel O. New algorithms and methods to estimate maximum-likelihood phylogenies: Assessing the performance of PhyML 3.0. Syst. Biol. 2010; 59:307–21.

69. Guindon S, Gascuel O. A simple, fast, and accurate algorithm to estimate large phylogenies by maximum likelihood. Syst. Biol. 2003; 52:696–704.

70. Yang Z. PAML 4: Phylogenetic analysis by maximum likelihood. Mol. Biol. Evol. 2007; 24:1586–91.

71. Yang Z, Rannala B. Bayesian estimation of species divergence times under a molecular clock using multiple fossil calibrations with soft bounds. Mol. Biol. Evol. 2006; 23:212–26.

72. Hahn MW, Demuth JP, Han SG. Accelerated rate of gene gain and loss in primates. Genetics. 2007; 177:1941–9.

73. Löytynoja A, Goldman N. An algorithm for progressive multiple alignment of sequences with insertions. Proc. Natl. Acad. Sci. USA. 2005; 102:10557–62.

74. Talavera G, Castresana J. Improvement of phylogenies after removing divergent and ambiguously aligned blocks from protein sequence alignments. Syst. Biol. 2007; 56:564–77.

75. Dawson AB. A note on the staining of the skeleton of cleared specimens with alizarin red S. Biotech. Histochem. 1926; 1:123-4.

76. Gruber HE. Adaptations of Goldner's Masson trichrome stain for the study of undecalcified plastic embedded bone. Biotech. Histochem. 1992; 67:30–4.

77. Ott HC, Matthiesen TS, Goh SK, Black LD, Kren SM, Netoff TI, et al. Perfusion-decellularized matrix: using nature's platform to engineer a bioartificial heart. Nat. Med. 2008; 14:213–21.

78. Langmead B, Salzberg SL. Fast gapped-read alignment with Bowtie 2. Nat. Methods. 2012; 9:357–9.

79. Li B, Dewey CN. RSEM: accurate transcript quantification from RNA-Seq data with or without a reference genome. BMC Bioinformatics. 2011; 12:323.

80. Mortazavi A, Williams BA, McCue K, Schaeffer L, Wold B. Mapping and quantifying mammalian transcriptomes by RNA-Seq. Nat. Methods. 2008; 5:621–8.

81. Robinson MD, Oshlack A. A scaling normalization method for differential expression analysis of RNA-seq data. Genome Biol. 2010; 11:R25.

82. Anders S, Huber W. Differential expression analysis for sequence count data. Genome Biol. 2010; 11:R106.

83. Niimura Y. Evolutionary dynamics of olfactory receptor genes in chordates: interaction between environments and genomic contents. Hum. Genomics. 2009; 4:107–18.

84. Altschul S, Madden T, Schaffer A, Zhang J, Zhang Z, Miller W, et al. Gapped BLAST and PSI-BLAST: a new generation of protein database search programs. Nucleic Acids Res. 1997; 25:3389–402.

85. Kumar S, Nei M, Dudley J, Tamura K. MEGA: A biologist-centric software for evolutionary analysis of DNA and protein sequences. Brief. Bioinform. 2008; 9:299–306.

86. Caporaso JG, Lauber CL, Walters WA, Berg-Lyons D, Lozupone CA, Turnbaugh PJ, et al. Global patterns of 16S rRNA diversity at a depth of millions of sequences per sample. Proc. Natl. Acad. Sci. USA. 2011; 108:4516–22.

87. Edgar, RC. UPARSE: highly accurate OTU sequences from microbial amplicon reads. Nat. Methods. 2013; 10:996–8.

88. Liu H, Chen CH, Gao ZX, Min JM, Gu YM, Jian JB, et al. The draft genome of *Megalobrama amblycephala* reveals the development of intermuscular bone and adaptation to herbivorous diet. 2016. GigaScience Database.

Figure Legends

Figure 1 Global view of the *M. amblycephala* genome and syntenic relationship between *Ctenopharyngodon idellus*, *M. amblycephala* and *Danio rerio*. (**A**) Global view of the *M. amblycephala* genome. From outside to inside, the genetic linkage map (a); Anchors between the genetic markers and the assembled scaffolds (b); Assembled chromosomes (c); GC content within a 50-kb sliding window (d); Repeat content within a 500-kb sliding window (e); Gene distribution on each chromosome (f); Different gene expression of three transcriptomes (g). (**B**) Syntenic relationship between *C. idellus* (a), *M. amblycephala* (b) and *D. rerio* (c) chromosomes.

Figure 2 Phylogenetic tree and comparison of orthologous genes in *M. amblycephala* and other fish species. (**A**) Phylogenetic tree of teleosts using 316 single copy orthologous genes. The color circles at the nodes shows the estimated divergence times using *O. latipes–F. rubripes* [96.9~150.9Mya], *F. rubripes–D. rerio* [149.85~165.2Mya], *F. rubripes–C. milii* [416~421.75Mya] (http://www.timetree.org/) as the calibration time. Pentagram represents four cyprinid fish with intermuscular bones. S, silurian period; D, devonian period; C, carboniferous period; P, permian periodin Paleozoic; T, triassic period; J, jurassic and k-cretaceous period in Mesozoic; Pg, paleogene in Cenozoic Era, N, Neogene. (**B**) Venn diagram of shared and unique orthologous gene families in *M. amblycephala* and four other teleosts. (**C**) Over-represented GO annotations of cyprinid-specific expansion gene families.

Figure 3 Regulation of genes related to intermuscular bone formation and function identified from developmental stages and adult tissues transcriptome data. (**A**) Gene expression pattern involved in muscle contraction regulated genes in early developmental stages corresponds to intermuscular bone formation of *M. amblycephala*, (alizarin red staining). M, myosepta; IB, intermusucular bone. (**B**) Scanning electron microscope photos of muscle tissues, connective tissues, and intermuscular bone. (**C**) Distribution of intermuscular bone specific genes in GO annotations indicative of abundance in protein binding, calcium ion binding, GTP binding functions. (**D**) Several developmental signals regulating key steps of osteoblast and osteoclast differentiation in the process of intramembranous ossification. Colored boxes indicate significantly up-regulated genes in these signals specifically occurred in intermuscular bone.

Figure 4 Molecular characteristics of sensory systems and the composition of gut microbiota in *M. amblycephala*. (**A**) Extensive expansion of olfactory receptor genes (ORs) in *M. amblycephala* compared with other teleosts. (**B**) Phylogeny of 'beta' type ORs in eight representative teleost species showing the significant expansion of 'beta' ORs in *M. amblycephala* and *C. idellus*. The pink background shows cyprinid-specific 'beta' types of ORs. (**C**) Umami, sweet and bitter tastes related gene families in teleosts with different feeding habits. (**D**) Structure of the umami receptor encoding T1R1 gene in cyprinid fish. (**E**) Relative abundance of microbial flora and taxonomic assignments in juvenile (LBSB), domestic adult (DBSB), wild adult (BSB) *M. amblycephala* and wild adult *C. idellus* (GC) samples at the phylum level.

Table

Table 1 Features of the *Megalobrama amblycephala* whole genome sequence

Total genome size (Mb)	1,116
N90 length of scaffold (bp)	20,422
N50 length of scaffold (bp)	838,704
N50 length of contig (bp)	49,400
Total GC content (%)	37.30
Protein-coding genes number	23,696
Average gene length (bp)	15,797
Content of transposable elements (%)	34.18
Number of chromosomes	24
Number of makers in genetic map	5,317
Scaffolds anchored on linkage groups (LGs)	1,434
Length of scaffolds anchored on LGs (Mb)	779.54 (70%)

1 Revised Manuscript (Text UNmarked) Click here to download Manuscript 1 Revised Manuscript (Text UNmarked).docx

The draft genome of *Megalobrama amblycephala* reveals the development of intermuscular bone and adaptation to herbivorous diet

Han Liu[1†], Chunhai Chen[2†], Zexia Gao[1†], Jiumeng Min[2†], Yongming Gu[3†], Jianbo Jian[2†], Xiewu Jiang[3], Huimin Cai[2], Ingo Ebersberger[4], Meng Xu[2], Xinhui Zhang[1], Jianwei Chen[2], Wei Luo[1], Boxiang Chen[1,3], Junhui Chen[2], Hong Liu[1], Jiang Li[2], Ruifang Lai[1], Mingzhou Bai[2], Jin Wei[1], Shaokui Yi[1], Huanling Wang[1], Xiaojuan Cao[1], Xiaoyun Zhou[1], Yuhua Zhao[1], Kaijian Wei[1], Ruibin Yang[1], Bingnan Liu[3], Shancen Zhao[2], Xiaodong Fang[2], Manfred Schartl[5,*], Xueqiao Qian[3,*], Weimin Wang[1,*]

[*]Equally contributing corresponding authors: wangwm@mail.hzau.edu.cn; xueqiaoqian@263.net; phch1@biozentrum.uni-wuerzburg.de

[†]Equal contributors

[1]College of Fisheries, Key Lab of Freshwater Animal Breeding, Ministry of Agriculture, Key Lab of Agricultural Animal Genetics, Breeding and Reproduction of Ministry of Education, Huazhong Agricultural University, Wuhan 430070, China

[2]Beijing Genomics Institute (BGI)-Shenzhen, Shenzhen 518083, China

[3]Guangdong Haid Group Co., Ltd., Guangzhou 511400, China

[4]Dept. for Applied Bioinformatics, Institute for Cell Biology and Neuroscience, Goethe University, Frankfurt D-60438, Germany

[5]Physiological Chemistry, University of Würzburg, Biozentrum, Am Hubland, and Comprehensive Cancer Center Mainfranken, University Clinic Würzburg, Würzburg 97070, Germany and Texas A&M Institute for Advanced Study and Department of Biology, Texas A&M University, College Station, TX 77843, USA

Abstract

Background: The blunt snout bream, *Megalobrama amblycephala*, is the economically most important cyprinid fish species. As an herbivore, it can be grown by eco-friendly and resource-conserving aquaculture. However, the large number of intermuscular bones in the trunk musculature is adverse to fish meat processing and consumption.

Results: As a first towards optimizing this aquatic livestock, we present a 1.116-Gb draft genome of *M. amblycephala*, with 779.54 Mb anchored on 24 linkage groups. Integrating spatiotemporal transcriptome analyses we show that intermuscular bone is formed in the more basal teleosts by intramembranous ossification, and may be involved in muscle contractibility and coordinating cellular events. Comparative analysis revealed that olfactory receptor genes, especially of the beta type, underwent an extensive expansion in herbivorous cyprinids, whereas the gene for the umami receptor *T1R1* was specifically lost in *M. amblycephala*. The composition of gut microflora, which contributes to the herbivorous adaptation of *M. amblycephala,* was found to be similar to that of other herbivores.

Conclusions: As a valuable resource for improvement of *M. amblycephala* livestock, the draft genome sequence offers new insights into the development of intermuscular bone and herbivorous adaptation.

Keywords: *Megalobrama amblycephala*, whole genome, herbivorous diet, intermuscular bone, transcriptome, gut microflora

Background

Fishery and aquaculture play an important role in global alimentation. Over the past decades food fish supply has been increasing with an annual rate of 3.6 percent, about 2 times faster than the human population [1]. This growth of fish production is meanwhile solely accomplished by an extension of aquaculture, as over the past thirty years the total mass of captured fish has remained almost constant [1]. As a consequence of this emphasis on fish breeding, the genomes of various economically important fish species, e.g. Atlantic cod (*Gadus morhua*) [2], rainbow trout (*Oncorhynchus mykiss*) [3], European sea bass (*Dicentrarchus labrax*) [4], yellow croaker (*Larimichthys crocea*) [5], half-smooth tongue sole (*Cynoglossus semilaevis*) [6], tilapia (*Oreochromis niloticus*) [7] and channel catfish (*Ictalurus punctatus*) [8] have been sequenced. Yet, the majority of these species are carnivorous requiring large inputs of protein from wild caught fish or other precious feed. Reports on draft genomes of some resource friendly herbivorous and omnivorous species, in particular cyprinid fish are scarce. It is well known that cyprinids are currently the economically most important group of teleosts for sustainable aquaculture. They grow to large population sizes in the wild and already now account for the majority of freshwater aquaculture production worldwide [1]. Among these, the herbivorous *Megalobrama amblycephala* (Yih, 1955), a particularly eco-friendly and resource-conserving species, is predominant in aquaculture and has been greatly developed in China (Additional file 1: Figure S1) [1]. However, most cyprinids, including *M. amblycephala*, have a large number of intermuscular bones (IBs) in the trunk musculature, which have an adverse effect on fish meat processing and consumption. IBs—a unique form of bone occurring only in the more basal teleosts—are completely embedded within the myosepta and are not connected to the vertebral column or any other bones [9, 10]. Our previous study on IB development of *M. amblycephala* revealed that some miRNA-mRNA interaction pairs may be involved in regulating bone development and differentiation [11]. However, the molecular genetic basis and the evolution of this unique structures remain obscure. Unfortunately, the recent sequencing of two cyprinid genomes common carp (*Cyprinus carpio*) [12] and grass carp (*Ctenopharyngodon idellus*) [13], which provided valuable information for their genetic breeding, contributed little to the understanding of IB formation.

In an initial genome survey of *M. amblycephala*, we identified 25,697 single-nucleotide polymorphism (SNP) [14], 347 conserved miRNAs [15], and many miRNA-mRNA interaction pairs [11]. However, lack of a whole genome sequence resource limited a thorough investigation of *M. amblycephala*. Here we report the first high-quality draft genome sequence of *M. amblycephala*. Integrating this novel genome resource with tissue- and developmental stage-specific gene expression information, as well as with meta-genome data to investigate the composition of the gut microbiome provides relevant insights into the function and evolution of two key features characterizing this species: The formation of IB and the adaptation to herbivory. By that our study lays the foundation for genetically optimizing *M. amblycephala* to further increase its relevance for securing human food supply.

Data description

Genome Assembly and Annotation

The *M. amblycephala* genome was sequenced and assembled by a whole-genome shotgun strategy using genomic DNA from a double-haploid fish (Additional file 1: Table S1). We assembled a 1.116 Gb reference genome sequence from 142.55 Gb (approximately 130-fold coverage) of clean data [16] (Additional file 1: Tables S1 and S2, Figure S2). The contig and scaffold N50 lengths reached 49 Kb and 839 Kb, respectively (Table 1). The largest scaffold spans 8,951 Kb and the 4,034 largest scaffolds cover 90% of the assembly. To assess the genome assembly quality, the mapping of paired end sequence data from the short-insert size WGS libraries, as well as of published ESTs [14] (Additional file 1: Tables S3 and S4) against the genome assembly indicated that the number and extent of misassemblies is low. To further estimate the completeness of the assembly and gene prediction, the benchmarking universal single-copy orthologs (BUSCO) [17] analysis was used and the results showed that the assembly contains 81.4% complete and 9.1% partial vertebrate BUSCO orthologues (Additional file 1: Table S5).

The *M. amblycephala* genome has an average GC content of 37.3%, similar to cyprinid *Cyprinus carpio* and *Danio rerio* (Additional file 1: Figures S3 and S4). Using a comprehensive annotation strategy combining RNA-seq derived transcript evidence, *de-novo* gene prediction and sequence similarity to proteins from five further fish species, we annotated a total of 23,696 protein-coding genes (Additional file 1: Table S6). Of the predicted genes, 99.44% (23,563 genes)

are annotated by functional database. In addition, we identified 1,796 non-coding RNAs including 474 miRNAs, 220 rRNA, 530 tRNAs, and 572 snRNAs. Transposable elements (TEs) comprise approximately 34.18% (381.3 Mb) of the *M. amblycephala* genome (Additional file 1: Table S7). DNA transposons (23.80%) and long terminal repeat retrotransposons (LTRs) (9.89%) are the most abundant TEs in *M. amblycephala*. The proportion of LTRs in *M. amblycephala* is highest in comparison with other teleosts: *G. morhua* (4.88%) [2], *L. crocea* (2.2%) [5], *C. semilaevis* (0.08%) [6], *C. carpio* (2.28%) [12], *C. idellus* (2.58%) [13] and stickleback (*Gasterosteus aculeatus*) (1.9%) [18] (Additional file 1: Tables S7 and S8, Figure S5). The distribution of divergence between the TEs in *M. amblycephala* peaks at only 7% (Additional file 1: Figure S6), indicating a more recent activity of these TEs when compared with *O. mykiss* (13%) [3] and *C. semilaevis* (9%) [6].

Anchoring Scaffolds and Shared Synteny Analysis

Sequencing data from 198 F1 specimens, including the parents, were used as the mapping population to anchor the scaffolds on to 24 psuedo-chromosomes of the *M. amblycephala* genome. Following RAD-Seq and sequencing protocol, 1883.5 Mb of 125-bp reads (on average 30.6 Mb and 9.3 Mb of read data for each parent and progeny, respectively) were generated on the HiSeq 2500 next-generation sequencing platform. Based on the SOAP bioinformatic pipeline, we generated 5,317 SNP markers for constructing a high-resolution genetic map. The map spans 1,701 cM with a mean marker distance of 0.33 cM and facilitated an anchoring of 1,434 scaffolds comprising 70% (779.54 Mb) of the *M. amblycephala* genome assembly to form 24 linkage groups (LG) (Additional file 1: Table S9). Of the anchored scaffolds, 598 could additionally be oriented (678.27 Mb, 87.01% of the total anchored sequences) (Figure 1A). A subsequent comparison of the gene order between *M. amblycephala* and its close relative *C.idellus* revealed 607 large shared syntenic blocks encompassing 11,259 genes, and 190 chromosomal rearrangements. The values change to 1,062 regions, 13,152 genes and 279 rearrangements when considering zebrafish (*Danio rerio*). The unexpected higher number of genes in syntenic regions shared with the more distantly related *D. rerio* is most likely an effect of the more complete genome assembly of this species compared to *C. idellus*. The rearrangement events are distributed across all *M. amblycephala* linkage groups without evidence for a local clustering (Figure 1B).

The most prominent event is a chromosomal fusion in *M. amblycephala* LG02 that joined two *D. rerio* chromosomes, Dre10 and Dre22. The same fusion is observed in *C. idellus* but not in *C. carpio* suggesting that it probably occurred in a last common ancestor of *M. amblycephala* and *C. idellus*, approximately 13.1 million years ago (Additional file 1: Figure S7).

Results

Evolutionary Analysis

A phylogenetic analysis of 316 single-copy genes with one to one orthologs in the genomes of 10 other fish species, and coelacanth (*Latimeria chalumnae*) and elephant shark (*Callorhinchus milii*), as out group served as a basis for investigating the evolutionary trajectory of *M. amblycephala* (Figure 2A, Additional file 1: Figure S8). To illuminate the evolutionary process resulting in the adaptation to a grass diet, we analyzed the functional properties of expanded gene families in the *M. amblycephala* and *C. idellus* lineage (Additional file 1: Figure S9, Additional file 2: Data Note1), two typical herbivores mainly feeding on aquatic and terrestrial grasses. Among the significantly over-represented KEGG pathways (Fisher's exact test, $P<0.01$), we find olfactory transduction (ko04740), immune-related pathways (ko04090, ko04672, ko04612 and ko04621), lipid metabolic related process (ko00590, ko03320, ko00591, ko00565, ko00592 and ko04975), as well as xenobiotics biodegradation and metabolism (ko00625 and ko00363) (Figure S10). These genes encoding proteins involved in biodegradation of xenobiotics would enhance the ability of an herbivore to detoxify the secondary compounds present in grasses that are adverse or even toxic to the organism. Furthermore, the high-fiber but low-energy grass diet requires a highly effective intermediate metabolism that accelerates carbohydrate and lipid catabolism and conversion into energy to maintain physiological functions. Indeed, when tracing positively selected genes (PSG) in *M. amblycephala* and *C. idellus* (Additional file 3: Date Note2), we identified many candidates involved in starch and sucrose metabolism (ko00500), citrate cycle (ko00020) and other types of O-glycan biosynthesis (ko00514). Moreover, 20 genes encoding enzymes involved in lipid and carbohydrate metabolism appear positively selected in both fish species (Additional file 1: Table S10).

Development of Intermuscular Bones

To explain the genetic basis of IB, their formation and their function in cyprinids, we first analyzed the functional annotation of gene families that expanded in this lineage (Figure 2C). Interestingly, many of these gene families are involved in cell adhesion (GO: 0007155, *P*=5.26E-32, 357 genes), myosin complex (GO:0016459, *P*=2.74E-08, 100 genes) and cell-matrix adhesion (GO:0007160, *P*=1.59E-21, 69 genes) (Figure 2C), which interact dynamically to mediate efficient cell motility, migration and muscle construction [19, 20].

As a second line of evidence, we performed transcriptome analyses of early developmental stages (stage1: whole larvae without IBs) and juvenile *M. amblycephala* (stage2: trunk muscle with partial IBs; stage3: trunk muscle with completed IBs) (Figure 3A). We found 249 genes significantly up-regulated in stages 2 and 3 (with IB) compared to stage 1 (no IB). Many of these genes belong to KEGG pathways involved in tight junction (ko04530), regulation of actin cytoskeleton (ko04810), cardiac muscle contraction (ko04260) and vascular smooth muscle contraction (ko04270) (Additional file 1: Figure S11). These genes are associated with cell motility and muscle contraction [19, 21, 22], which resembles the findings from the gene family expansion analysis. Specifically, some of these genes encoding proteins related to muscle contraction, including titin, troponin, myosin, actinin, calmodulin and other Ca^{2+} transporting ATPases (Figure 3A) point to a strong remodeling of the musculature compartment.

To confirm that the observed differences in gene expression are indeed linked to IB formation and function and are not simply due to the fact that different developmental stages were compared, we performed differential genes expression (DGE) analysis of muscle tissues, IB, and connective tissues from the same six months old individual of *M. amblycephala* (Figure 3B, Additional file 1: Figure S12). Among the genes that are significantly up-regulated in the IB samples many encode extracellular matrix (ECM) proteins (collagens and intergrin-binding protein), Rho GTPase family (*RhoA*, *Rho GAP*, *Rac*, *Ras*), motor proteins (myosin, dynein, actin), and calcium channel regulation protein (Additional file 1: Figure S13 and Table S11). Interestingly, it has been demonstrated that ECM proteins bound to integrins influence cell migration by actomyosin-generated contractile forces [20, 23]. Rho GTPases, acting as molecular switches, also is involved in regulating the actin cytoskeleton and cell migration, which in turn initiates intracellular signaling and contributes to tissue repair and regeneration [24-26].

During development of *M. amblycephala*, the first IB appears in muscles of caudal vertebrae as early as 28 days post fertilization (dpf) when body length is 12.95 mm (Additional file 1: Figure S14). The system then develops and ossifies predominantly from posterior to anterior (Additional file 1: Figure S15). IBs are present throughout the body within two months (Additional file 1: Figure S16) and develop into multiple morphological types in adults (Additional file 1: Figure S17). The bone is formed directly without an intermediate cartilaginous stage (Additional file 1: Figures S18 and S19). We also found a large number of mature osteoblasts distributed at the edge of the bone matrix while some osteocytes were apparent in the center of the mineralized bone matrix (Additional file 1: Figures S20 and S21). These primary bone-forming cells predominantly regulate bone formation and function throughout life. Notably, among the genes up-regulated in IB, 35 bone formation regulatory genes were identified (shown in colored boxes in Figure 3D). In particular, genes involved in bone morphogenetic protein (BMP) signaling including *Bmp3*, *Smad8*, *Smad9*, and *Id2*, in fibroblast growth factor (FGF) signaling including *Fgf2*, *Fgfr1a*, *Fgfbp2*, *Col6a3*, and *Col4a5*, and in Ca^{2+} channels including *Cacna1c*, *CaM*, *Creb5*, and *Nfatc* were highly expressed (>2-fold change) in IB (Additional file 1: Figure S22). It has been demonstrated that *Bmp*, *Fgf2*, and *Fgfr1* are involved in intramembranous bone development and affect the expression and activity of other osteogenesis related transcription factors [27, 28]. The calcium-sensitive transcription factor *NFATc1* together with *CREB* induces the expression of osteoclast-specific genes [29].

Adaptation to Herbivorous Diet

Next to the presence of IB, the herbivory of *M. amblycephala* is the second key feature in connection to the use of this species as aquatic livestock. Olfaction, the sense of smell, is crucial for animals to find food. The perception of smell is mediated by a large gene family of olfactory receptor (OR) genes. The ORs of teleosts are predominantly expressed in the main olfactory epithelium of the nasal cavity [30, 31] and can discriminate, like those of other vertebrates, different kinds of odor molecules. However, compared to mammals, e.g. humans having around 400 ORs [32] the OR repertoires in teleosts are considerably small. They range from only about 48 in *Fugu rubripes* up to 161 in *D. rerio* (Figure 4A). In the *M. amblycephala* genome, we

identified 179 functional olfactory receptor (OR) genes (Figure 4A), and based on the classification of Niimura [33], 158, 117 and 153 receptors for water-borne odorants were identified in *M. amblycephala*, *C. idellus* and *D. rerio*, respectively (Additional file 1: Table S12). Overall, these receptor repertoires are substantially larger than those of other and carnivorous teleosts (*G. morhua*, *C. semilaevis*, *O. latipes*, *X. maculatus*) (Additional file 1: Figures S23 and S24, Table S12). This suggests that olfaction—probably for food choice—has a particularly important role in the cyprinid species. Previous studies have demonstrated that the beta type OR genes are present in both aquatic and terrestrial vertebrates, indicating that the corresponding receptors detect both water-soluble and airborne odorants [31, 33]. Intriguingly, we found a massive expansion of beta-type OR genes in the genomes of the herbivorous *M. amblycephala* and *C. idellus*, while very few exist in other teleosts (Figure 4B, Additional file 1: Table S12).

Taste is also an important factor in the development of dietary habits. Most animals can perceive five basic tastes, namely sourness, sweetness, bitterness, saltiness and umami [34]. Interestingly, *T1R1*, the receptor gene necessary for sensing umami, has been lost in herbivorous *M. amblycephala* but is duplicated in carnivorous *G. morhua*, *C. semilaevis* and omnivorous *O. latipes* and *X. maculatus* (Figures 4C and 4D, Additional file 1: Figures S25-26 and Table S13). In contrast, *T1R2*, the receptor gene for sensing sweet, has been duplicated in herbivorous *M. amblycephala* and *C. idellus*, omnivorous *C. carpio* and *D. rerio*, while it has been lost in carnivorous *G. morhua* and *C. semilaevis* (Additional file 1: Figure S27 and Table S13). Bitterness sensed by the *T2R* is particularly crucial for animals to protect them from poisonous compounds [35]. Probably in the course switching to a diet that contains a larger fraction of bitterness containing food, also the *T2R* gene family in *M. amblycephala*, *C. idellus*, *C. carpio* and *D. rerio* has been expanded (Additional file 1: Figure S28).

To obtain further insights into the genetic adaptation to herbivorous diet, we focused on further genes that might be associated with digestion. Genes that encode proteases (including pepsin, trypsin, cathepsin and chymotrypsin) and amylases (including alpha-amylase and glucoamylase) were identified in the genomes of *M. amblycephala*, carnivorous *C. semilaevis*, *G. morhua* and omnivorous *D. rerio, O. latipes* and *X. maculatus*, indicating that herbivorous *M. amblycephala* has a protease repertoire that is not substantially different from those of carnivorous

and omnivorous fishes (Additional file 1: Table S14). We did not identify any genes encoding potentially cellulose-degrading enzymes including endoglucanase, exoglucanase and beta-glucosidase in the genome of *M. amblycephala,* suggesting that utilization of the herbivorous diet may largely depend on the gut microbiome. To elucidate this further, we determined the composition of the gut microbial communities of juvenile, domestic, wild adult *M. amblycephala* and wild adult *C. idellus* using bacterial 16S rRNA sequencing. A total of 549,020 filtered high quality sequence reads from 12 samples were clustered at a similarity level of 97%. The resulting 8,558 operational taxonomic units (OTUs) are dominated at phylum level by Proteobacteria, Fusobacteria, Bacteroidetes, Firmicutes and Actinobacteria (Additional file 1: Table S15, Figure 4E). Increasing the resolution to the genus level, the composition and relative abundance of the gut microbiota of wild adult *M. amblycephala* and *C. idellus* are still very similar (Additional file 1: Table S16) and we could identify more than 7% cellulose-degrading bacteria (Additional file 1: Table S17). This indicates that indeed the gut microbiome is crucial in the digestion of plant material, and thus in the adaptation to herbivory.

Discussion

M. amblycephala is the economically most important species for freshwater aquaculture. In addition to its various superior properties, especially its herbivorous diet, it is also an excellent model to study IB formation. Here we make available draft genome of *M. amblycephala* with more than 70% of genome data anchored on 24 linkage groups. Comparative analyses of genome structure revealed high synteny with three other cyprinid fish and uncovered a chromosomal fusion event in *M. amblycephala* that joined two *D. rerio* chromosomes (Figure 1B), which supports the previous results in *C. idellus* [13] and also provides novel scientific insights into the evolution of chromosome fusion events in cyprinids.

The evolutionary trajectory analysis of *M. amblycephala* and other teleosts revealed that *M. amblycephala* has the closest relationship to *C. idellus* (Figure 2A). Both the species are herbivorous fish but which endogenous and exogenous factors affected their feeding habits and how they adapted to their herbivorous diet is not known. Olfaction and taste are crucial for animals to find food and to distinguish whether potential food is edible or harmful [31, 35]. The

search for genes encoding OR showed that herbivorous *M. amblycephala* and *C. idellus* have a large number of beta-type OR, while other omnivorous and carnivorous fish only have one or two. This might be attributed to their particular herbivorous diet consisting not only of aquatic grasses but also the duckweed and terrestrial grasses, which they ingest from the water surface. Previous studies have demonstrated that the receptor for umami is formed by the T1R1/T1R3 heterodimer, while T1R2/T1R3 senses sweet taste [36]. We found that the umami gene *T1R1* was lost in herbivorous *M. amblycephala* but duplicated in the carnivorous *G. morhua* and *C. semilaevis* (Figure 4C). The loss of the *T1R1* gene in *M. amblycephala* might exclude the expression of a functional umami taste receptor. Such situations in other organism, e.g. the Chinese panda, have previously been related to feeding specialization [37]. Interestingly, the sweetness receptor *T1R2* and bitter receptor *T2R* genes are expanded in the herbivorous fish but few or no copy was found in carnivorous fish. Collectively, these results not only indicate the genetic adaptation to herbivorous diet of *M. amblycephala*, but also provided a clear and comprehensive picture of adaptive evolutionary mechanisms of sensory systems in other fish species with different trophic specializations.

Some insects such as *Tenebrio molitor* [38] and *Neotermes koshunensis* [39], and the mollusc *Corbicula japonica* [40] have genes encoding endogenous cellulose degradation-related enzymes. However, all so far analyzed herbivorous vertebrates lack these genes and always rely on their gut microbiome to digest food [37, 41]. In herbivorous *M. amblycephala* and *C. idellus*, we also did not find any homologues of digestive cellulase genes. Interestingly, our work on the composition of gut microbiota of the two fish species identifies more than 7% cellulose-degrading bacteria, suggesting that the cellulose degradation of herbivorous fish largely depend on their gut microbiome.

IB has evolved several times during teleost evolution [9, 42]. The developmental mechanisms and ossification processes forming IBs are dramatically distinct from other bones such as ribs, skeleton, vertebrae or spines. These usually develop from cartilaginous bone and are derived from the mesenchymal cell population by endochondral ossification [27, 43]. However, IBs form directly by intramembranous ossification and differentiate from osteoblasts within connective tissue, forming segmental, serially homologous ossifications in the myosepta. Although various

methods of ossification of IB have been proposed, few experiments have been conducted to confirm the ossification process and little is known about the potential role of IB in teleosts. Based on our findings of over-represented functional properties of expanded gene families in cyprinid lineage (Figure 2C) and evidence from DGE of early developmental stages of IB formation (Figure 3A), we provide molecular evidence that IB might play significant roles not only in regulating muscle contraction but also in active remodeling at the bone-muscle interface and coordination of cellular events.

It has previously been found that some major developmental signals including BMP, FGF, WNT, together with calcium/calmodulin signaling [27, 44-46], are essential for regulating the differentiation and function of osteoblasts and osteocytes and for regulating the RANKL signaling pathway for osteoclasts [47] in intramembranous bone development. In agreement with this concept, our DGE analysis of muscle, IB and connective tissues uncovered that 35 bone formation regulatory genes involved in these signals were highly up-regulated in IB. Taken together, these results suggest that IB indeed undergoes an intramembranous ossification process, is regulated by bone-specific signaling pathways, and underlies a homeostasis of maintenance, repair and remodeling.

Conclusions

Our results provide novel functional insights into the evolution of cyprinids. Importantly, the *M. amblycephala* genome data come up with novel insights shedding light on the adaptation to herbivorous nutrition and evolution and formation of IB. Our results on the evolution of gene families, digestive and sensory system, as well as our microbiome meta-analysis and transcriptome data provide powerful evidence and a key database for future investigations to increase the understanding of the specific characteristics of *M. amblycephala* and other fish species.

Methods

Sampling and DNA Extraction

DNA for genome sequencing was derived from a double haploid fish from the *M. amblycephala* genetic breeding center at Huazhong Agricultural University (Wuhan, Hubei, China). Fish blood

was collected from adult female fish caudal vein using sterile injectors with pre-added anticoagulant solutions following anesthetized with MS-222 and sterilization with 75% alcohol. Genomic DNA was extracted from the whole blood.

Genomic Sequencing and Assembly

Libraries with different insert sized inserts of 170 bp, 500 bp, 800 bp, 2 Kb, 5 Kb, 10 Kb and 20 Kb were constructed from the genomic DNA at BGI-Shenzhen. The libraries were sequenced using a HiSeq2000 instrument. In total, 11 libraries, sequenced in 23 lanes were constructed. To obtain high quality data, we applied filtering criteria for the raw reads. As a result, 142.55 Gb of filtered data were used to complete the genome assembly using SOAPdenovo_V2.04 [16]. Only filtered data were used in the genome assembly. First, the short insert size library data were used to construct a de Bruijn graph. The tips, merged bubbles and connections with low coverage were removed before resolving the small repeats. Second, all high-quality reads were realigned with the contig sequences. The number of shared paired-end relationships between pairs of contigs was calculated and weighted with the rate of consistent and conflicting paired ends before constructing the scaffolds in a stepwise manner from the short–insert size paired ends to the long–insert size paired ends. Third, the gaps between the constructed scaffolds were composed mainly of repeats, which were masked during scaffold construction. These gaps were closed using the paired-end information to retrieve read pairs in which one end mapped to a unique contig and the other was located in the gap region. Subsequently, local assembly was conducted for these collected reads. To assess the genome assembly quality, approximately 42.82 Gb Illumina reads generated from short-insert size libraries were mapped onto the genome. Bwa0.5.9-r16 software [48] with default parameters was used to assess the mapping ratio and Soap coverage 2.27 was used to calculate the sequencing depth. We also assessed the accuracy of the genome assembly by Trinity [49], including number of ESTs and new mRNA reads from early stages of embryos and multiple tissues, by aligning the scaffolds to the assembled transcriptome sequences.

After obtaining K-mers from the short-insert-size (<1Kb) reads with just one bp slide, frequencies of each K-mer were calculated. The K-mer frequency fits Poisson distribution when a sufficient amount of data is present. The total genome size was deduced from these data in the following way: Genome size = K-mer num / Peak_depth.

Genome Annotation

The genome was searched for repetitive elements using Tandem Repeats Finder (version 4.04) [50]. TEs were identified using homology-based approaches. The Repbase (version 16.10) [51] database of known repeats and a *de novo* repeat library generated by RepeatModeler were used. This database was mapped using the software of RepeatMasker (version 3.3.0). Four types of non-coding RNAs (microRNAs, transfer RNAs, ribosomal RNAs and small nuclear RNAs) were also annotated using tRNAscan-SE (version 1.23) and the Rfam database45 (Release 9.1) [52].

For gene prediction, *de novo* gene prediction, homology-based methods and RNA-seq data were used to perform gene prediction. For the sequence similarity based prediction, *D. rerio, G. aculeatus, O. niloticus, O. latipes* and *G. morhua* protein sequences were downloaded from Ensembl (release 73) and were aligned to the *M. amblycephala* genome using TBLASTN. Then homologous genome sequences were aligned against the matching proteins using GeneWise [53] to define gene models. Augustus was employed to predict coding genes using appropriate parameters in *de novo* prediction. For the RNA-seq based prediction, we mapped transcriptome reads to the genome assembly using TopHat [54]. Then, we combined TopHat mapping results together and applied Cufflinks [55] to predict transcript structures. All predicted gene structures were integrated by GLEAN [56] (http://sourceforge.net/projects/glean-gene/) to obtain a consensus gene set. Gene functions were assigned to the translated protein-coding genes using Blastp tool, based on their highest match to proteins in the SwissProt and TrEMBL [57] databases (Uniprot release 2011-01). Motifs and domains in the protein-coding genes were determined by InterProScan (version 4.7) searches against six different protein databases: ProDom, PRINTS, Pfam, SMART, PANTHER and PROSITE. Gene Ontology [58] IDs for each gene were obtained from the corresponding InterPro entries. All genes were aligned against KEGG [59] (Release 58) database, and the pathway in which the gene might be involved was derived from the matched genes in KEGG. tRNA genes were *de novo* predicted by tRNAscan-SE software [60], with eukaryote parameters on the repeat pre-masked genome. The rRNA fragments were identified by aligning the rRNA sequences using BlastN at E-value 1e-5. The snRNA and miRNA were searched by the method of aligning and searching with INFERNAL (version 0.81) [61] against Rfam database (release 9.1).

Genetic map construction

To anchor the scaffolds into pseudo-chromosomes, 198 F1 population individuals were used to obtain the genetic map. Each of the individual genomic DNA was digested with the restriction endonuclease EcoR I, following the RAD-Seq protocol [62]. The SNP calling process was carried out using the SOAP bioinformatic pipeline. The RAD-based SNP calling was done by SOAPsnp software [63] after each individual's paired-end RAD reads was mapped onto the assembled reference genome with the alignment software SOAP2 [64]. The potential SNP markers were used for the linkage analysis if the following criteria were satisfied: for parents - sequencing depth ≥8 and ≤ 100, base quality ≥ 25, copy number ≤ 1.5; for progeny - sequencing depth ≥ 5, base quality ≥ 20, copy number ≤ 1.5. If the markers were showing significantly distorted segregation (*P*-value < 0.01), they were excluded from the map construction. Linkage analysis was performed only for markers present in at least 80% of the genomes, using JoinMap 4.0 software with CP population type codes and applying the double pseudo-test cross strategy [65]. The linkage groups were formed at a logarithm of odds threshold of 6.0 and ordered using the regression mapping algorithm.

Construction of gene families

We identified gene families using TreeFam software [66] as follows: Blast was used to compare all the protein sequences from 13 species: *M. amblycephala*, *C. idellus*, *C. semilaevis*, *C. carpio*, *D. rerio*, *Callorhinchus milii*, *G. morhua*, *G. aculeatus*, *Latimeria chalumnae*, *Oncorhynchus mykiss*, *O. niloticus*, *O. latipes*, *Fugu rubripes*, with the E-value threshold set as 1e-7. In the next step, HSP segments of each protein pair were concatenated by Solar software. H-scores were computed based on Bit-scores and these were taken to evaluate the similarity among genes. Finally, gene families were obtained by clustering of homologous gene sequences using Hcluster_sg (Version 0.5.0). Specific genes of *M. amblycephala* were those that did not cluster with other vertebrates that were chosen for gene family construction, and those that did not have homologs in the predicted gene repertoire of the compared genomes. If these genes had functional motifs, they were annotated by GO.

Phylogenetic Tree Reconstruction and Divergence Time Estimation

The coding sequences of single-copy gene families conserved among *M. amblycephala*, *C. idellus*,

C. carpio, D. rerio, C. semilaevis, G. morhua, G. aculeatus, Latimeria chalumnae, O. mykiss, O. niloticus, O. latipes, C. milii and *Fugu rubripes* (Ensembl Gene v.77) were extracted and aligned with guidance from amino-acid alignments created by the MUSCLE program [67]. The individual sequence alignments were then concatenated to form one supermatrix. PhyML [68, 69] was applied to construct the phylogenetic tree under an HKY85+gamma model for nucleotide sequences. ALRT values were taken to assess the branch reliability in PhyML. The same set of codon sequences at position 2 was used for phylogenetic tree construction and estimation of the divergence time. The PAML mcmctree program (PAML version 4.5) [70, 71] was used to determine divergence times with the approximate likelihood calculation method and the 'correlated molecular clock' and 'REV' substitution model.

Gene Family Expansion and Contraction Analysis

Protein sequences of *M. amblycephala* and 11 other related species (Ensembl Gene v.77)) were used in BLAST searches to identify homologs. We identified gene families using CAFÉ [72], which employs a random birth and death model to study gene gains and losses in gene families across a user-specified phylogeny. The global parameter λ, which describes both the gene birth (λ) and death ($\mu = -\lambda$) rate across all branches in the tree for all gene families, was estimated using maximum likelihood. A conditional *P*-value was calculated for each gene family, and families with conditional *P*-values less than the threshold (0.05) were considered as having notable gain or loss. We identified branches responsible for low overall *P*-values of significant families.

Detection of Positively Selected Genes

We calculated Ka/Ks ratios for all single copy orthologs of *M. amblycephala* and *C. semilaevis, D. rerio, G. morhua, O. niloticus and C. carpio*. Alignment quality was essential for estimating positive selection. Thus, orthologous genes were first aligned by PRANK [73], which is considerably conservative for inferring positive selection. We used Gblocks [74] to remove ambiguously aligned blocks within PRANK alignments and employed ‘codeml’ in the PAML package with the free-ratio model to estimate Ka, Ks, and Ka/Ks ratios on different branches. The differences in mean Ka/Ks ratios for single-copy genes between *M. amblycephala* and each of the other species were compared using paired Wilcoxon rank sum tests. Genes that showed values of Ka/Ks higher than 1 along the branch leading to *M. amblycephala* were reanalyzed using the

codon based branch-site tests implemented in PAML. The branch-site model allowed ω to vary both among sites in the protein and across branches, and was used to detect episodic positive selection.

Developmental process of intermuscular bone in *M. amblycephala*

To better understand the number and morphological types of IBs in adult *M. amblycephala*, specimens with a body length ranging from 15.5 to 20.5 cm were collected and each individual was wrapped in gauze and boiled. The fish body was divided into two sections: anterior (snout to cloaca) and posterior (cloaca to the base of caudal fin), and the length of each section was measured. The IBs were retrieved, counted, arranged in order and photographed with a digital camera. Fertilized *M. amblycephala* eggs were brought from hatching facilities at Freshwater Fish Genetics Breeding Center of Huazhong Agricultural University (Wuhan, Hubei, China) to our laboratory. *M. amblycephala* larvae were maintained in a re-circulating aquaculture system at 23 ± 1°C with a 14-hr photoperiod. To explore the early development of IBs, larvae at different stages from 15 to 40 dpf were collected and fixed in 4% paraformaldehyde and transferred to 70% ethanol for storage. Specimens were stained with alizarin red for bone following the method described by Dawson [75]. The appearance of red color was recorded as the appearance of IB because bone ossification is accompanied by the uptake of alizarin red, resulting in red staining of the mineralized bone matrix. Myosepta, either not yet ossified, or poorly ossified, are not visible with alizarin red staining. For histologic analysis, specimens were paraffin-embedded and sectioned following standard protocols. Sections were stained with hematoxylin and eosin (HE) and Masson trichrome [76] and photographed using a Nikon microscope (Nikon, Tokyo, Japan) with a DP70 digital camera (Olympus, Japan). Scanning electron microscopy (SEM) and transmission electron microscopy (TEM) were also conducted to analyze the ultrastructure of IB. The specimens were fixed with 2.5% (v/v) glutaraldehyde in a solution of 0.1 M sodium cacodylate buffer (pH 7.3) for 2 h at room temperature. The SEM and TEM samples were prepared according to a standard protocol described by Ott [77]. The samples were then visualized with a JSM-6390LV scanning electron microscope (SEM, Japan) and the stained ultrathin sections with a H-7650 transmission electron microscope (Hitachi, Japan).

RNA Sequencing Analysis

M. amblycephala specimens belonging to three different developmental stages of IBs (stage 1: whole larvae without distribution of IB; stage 2: muscle tissues with partial distribution of IBs; stage 3: muscle tissues with completed distribution of IBs were identified under microscope and immediately frozen in liquid nitrogen. In addition, dorsal white muscle, IBs and connective tissue surrounding the IBs from six months old fish were also collected. RNA was extracted from total fish samples at different stages and from individual muscle, connective tissue, and intermuscular bone samples of *M. amblycephala* using RNAisoPlus Reagent (TaKaRa, China) according to the manufacturer's protocol. The integrity and purity of the RNA was determined by gel electrophoresis and Agilent 2100 BioAnalyzer (Agilent, USA) before preparing the libraries for sequencing. Paired-end RNA sequencing was performed using the Illumina HiSeq 2000 platform. Low quality score reads were filtered and the clean data were aligned to the reference genome using Bowtie [78]. Genes and isoforms expression level were quantified by a software package: RSEM (RNASeq by Expectation Maximization) [79]. Gene expression levels were calculated by using the RPKM method (Reads per kilobase transcriptome per million mapped reads) [80] and adjusted by a scaling normalization method [81]. DEGs were detected using DESeq [82]. Annotation of DEGs were mapped to GO categories in the database (http://www.geneontology.org/) and the number of genes for every term were calculated to identify GO terms that were significantly enriched in the input list of DEGs. The calculated *P*-value was adjusted by the Bonferroni Correction, taking corrected *P*-value ≤ 0.05 as a threshold. KEGG automatic annotation was used to perform pathway enrichment analysis of DEGs.

Prediction of Olfactory Receptor Genes

Olfactory receptor genes were identified by previously described methods [83], with the exception of a first-round TBLASTN [84] search, in which 1,417 functional olfactory receptor genes from *H. sapiens*, *D. rerio*, *L. chalumnae*, *Lepisosteus oculatus*, *L. vexillifer*, *O. niloticus*, *O. latipes*, *F. rubripes* and *Xenopus tropicalis* were used as queries. We then predicted the structure of sequenced genes using the blast-hit sequence with the software GeneWise extending in both 3' and 5' directions along the genome sequences. The results were further confirmed by NR annotation. Then the coding sequences from the start (ATG) to stop codons were extracted, while sequences that contained interrupting stop codons or frame-shifts were regarded as pseudogenes. To

construct phylogenetic trees, the amino-acid sequences encoded by olfactory receptor genes were first aligned using the program MUSCLE nested in MEGA 5.10 [85]. We then constructed the phylogenetic tree using the neighbor-joining method with Poisson correction distances using the program MEGA 5.10. We also identified and compared the genes for five basic tastes (sour, sweet, bitter, umami and salty) using a similar method as in OR gene identification.

Gut microbiota analysis

To characterize the microbial diversity of herbivorous *M. amblycephala*, a total of 12 juvenile (LBSB), domestic adult (DBSB), wild adult *M. amblycephala* (BSB) and wild adult *C. idellus* (GC) intestinal fecal samples were collected. Bacterial genomic DNA was extracted from 200 mg gut content of each sample using a QIAamp DNA Stool Mini Kit (Qiagen, Valencia, USA). Quality and integrity of each DNA sample were determined by 1% agarose gel electrophoresis in Tris-acetate-EDTA (TAE) buffer. DNA concentration was quantified using NanoDrop ND-2000 spectrophotometer (Thermo Scientific). To determine the diversity and composition of the bacterial communities of each sample, a total of 20 μg of genomic DNA were sequenced using the Illumina MiSeq sequencing platform. PCR amplifications were conducted from each sample to produce the V4 hypervariable region (515F and 806 R) of the 16S rRNA gene according to the previously described method [86]. We used the UPARSE pipeline [87] to pick operational taxonomic units (OTUs) at an identity threshold of 97% and picked representative sequences for each OTU and used the RDP classifier to assign taxonomic data to each representative sequence.

Additional files

Additional file 1: Tables S1 to S17 and Figures S1 to S28.

Additional file 2: Data Note1 Expanded genes in the *M. amblycephala* and *C. idellus* lineage.

Additional file 3: Data Note2 Positively selected genes in the *M. amblycephala* and *C. idellus* genomes.

Abbreviations

IB, intermuscular bone; SNP, single-nucleotide polymorphism; BUSCO, benchmarking universal single-copy orthologs; TE, transposable element; LTR, long terminal repeat retrotransposon; LG, linkage group; PSG, positively selected gene; ECM, extracellular matrix; dpf, days post fertilization; BMP, bone morphogenetic protein; FGF, fibroblast growth factor; OR, olfactory

receptor; OTU, operational taxonomic unit; DGE, differential genes expression; HE, hematoxylin and eosin; SEM, scanning electron microscopy; TEM, transmission electron microscopy

Acknowledgements

This work was supported by the Modern Agriculture Industry Technology System Construction Projects of China titled as—Staple Freshwater Fishes Industry Technology System (No. CARS-46-05), Guangdong Haid Group Co., Ltd, the Fundament Research Funds for the Central Universities (2662015PY019), the International Scientific and Technology Cooperation Program of Wuhan City (2015030809020365).

Availability of data and materials

Datasets supporting the results of this article are available in the GigaDB repository associated with this publication [88]. Raw whole genome sequencing, transcriptome and RAD-Seq data have been deposited at NCBI in the SRA under bioproject number PRJNA343584.

Authors' contributions

W.W. initiated and conceived the project and provided scientific input. X.Q. organized financial support and designed the project. M.S. discussed the data, wrote and modified the paper. H.L. and C.C. conducted the biological experiments, analyzed the data and wrote the paper with input from other authors. I.E. wrote and modified the manuscript and discussed the data. The RAD-Seq data analyses and the genetic map construction were performed by Z.G., Y.G., J.J. and X.J. Genome assembly and annotation were performed by J.M., H.C., M.X. and J.C. X.Z., W.L., R.L., B.C., J.W., H.L., S.Y., H.W., X.C., X.Z., Y.Z., K.W., R.Y. and B. L. carried out the samples preparation and data collection. J.L. and J.C. identified the gene families and analyzed the RNA-seq data. M.B. coordinated the project. S.Z. and X.F. modified the manuscript and discussed the data. All authors read the manuscript and provided comments and suggestions for improvements. The authors declare no competing financial interests.

Competing interests

The authors declare that they have no competing interests.

Ethics approval and consent to participate

All experimental procedures involving fish were performed in accordance with the guidelines and regulations of the National Institute of Health Guide for the Care and Use of Laboratory Animals and the Institutional Review Board on Bioethics and Biosafety of BGI (BGI-IRB).

References

1. FAO Fisheries and Aquaculture Department. FAO yearbook Fishery and Aquaculture Statistics 2014 (Food and Agriculture Organization of the United Nations, Rome, 2016).

2. Star B, Nederbragt AJ, Jentoft S, Grimholt U, Malmstrøm M, Gregers TF, et al. The genome sequence of Atlantic cod reveals a unique immune system. Nature. 2011; 477:207–10.

3. Berthelot C, Brunet F, Chalopin D, Juanchich A, Bernard M, Noël B, et al. The rainbow trout genome provides novel insights into evolution after whole-genome duplication in vertebrates. Nat. Commun. 2014; 5:3657.

4. Tine M., Kuhl H., Gagnaire PA, Louro B, Desmarais E, Martins RS, et al. European sea bass genome and its variation provide insights into adaptation to euryhalinity and speciation. Nat. Commun. 2014; 5:5770.

5. Wu C, Zhang D, Kan M, Lv Z, Zhu A, Su Y, et al. The draft genome of the large yellow croaker reveals well-developed innate immunity. Nat. Commun. 2014; 5:5227.

6. Chen S, Zhang G, Shao C, Huang Q, Liu G, Zhang P, et al. Whole-genome sequence of a flatfish provides insights into ZW sex chromosome evolution and adaptation to a benthic lifestyle. Nat. Genet. 2014; 46:253–60.

7. Brawand D, Wagner CE, Li YI, Malinsky M, Keller I, Fan S, et al. The genomic substrate for adaptive radiation in African cichlid fish. Nature. 2014; 513:375–82.

8. Chen X, Zhong L, Bian C, Xu P, Qiu Y, You X, et al. High-quality genome assembly of channel catfish, *Ictalurus punctatus*. GigaScience. 2016; 5:39.

9. Gemballa S, Britz R. Homology of intermuscular bones in acanthomorph fishes. Am. Mus. Novit. 1998; 3241:1–25.

10. Danos N, Ward AB. The homology and origins of intermuscular bones in fishes: Phylogenetic or biomechanical determinants? Biol. J. Linn. Soc. 2012; 106:607–22.

11. Wan SM, Yi SK, Zhong J, Nie CH, Guan NN, Zhang WZ, et al. Dynamic mRNA and miRNA expression analysis in response to intermuscular bone development of blunt snout bream (*Megalobrama amblycephala*). Sci. Rep. 2016; 6:31050.

12. Xu P, Zhang X, Wang X, Li J, Liu G, Kuang Y, et al. Genome sequence and genetic diversity of the common carp, *Cyprinus carpio*. Nat. Genet. 2014; 46:1212–9.

13. Wang Y, Lu Y, Zhang Y, Ning Z, Li Y, Zhao Q, et al. The draft genome of the grass carp (*Ctenopharyngodon idellus*) provides insights into its evolution and vegetarian adaptation. Nat. Genet. 2015; 47:625–31.

14. Gao Z, Luo W, Liu H, Zeng C, Liu X, Yi S, et al. Transcriptome analysis and SSR/SNP markers information of the blunt snout bream (*Megalobrama amblycephala*). PLoS One. 2012; 7:e42637.

15. Yi S, Gao ZX, Zhao H, Zeng C, Luo W, Chen B, et al. Identification and characterization of microRNAs involved in growth of blunt snout bream (*Megalobrama amblycephala*) by Solexa sequencing. BMC Genomics. 2013; 14:754.

16. Luo R, Liu B, Xie Y, Li Z, Huang W, Yuan J, et al. SOAPdenovo2: an empirically improved memory-efficient short-read de novo assembler. Gigascience. 2012;1:18.

17. Simao FA, Waterhouse RM, Ioannidis P, Kriventseva EV, Zdobnov EM. BUSCO: assessing genome assembly and annotation completeness with single-copy orthologs. Bioinformatics. 2015;31:3210–2.

18. Jones FC, Grabherr MG, Chan YF, Russell P, Mauceli E, Johnson J, et al. The genomic basis of adaptive evolution in threespine sticklebacks. Nature. 2012; 484:55–61.

19. Even-Ram S, Doyle AD, Conti MA, Matsumoto K, Adelstein RS, Yamada KM. Myosin IIA regulates cell motility and actomyosin-microtubule crosstalk. Nat. Cell Biol. 2007; 9:299–309.

20. Sheetz MP, Felsenfeld DP, Galbraith CG. Cell migration : Regulation of force on extracellular-complexes. Trends Cell Biol. 1998; 8:51–4.

21. Gunst SJ, Zhang W. Actin cytoskeletal dynamics in smooth muscle: a new paradigm for the regulation of smooth muscle contraction. Am. J. Physiol. Cell Physiol. 2008; 295:C576–87.

22. Webb RC. Smooth muscle contraction and relaxation. Adv. Physiol. Educ. 2003; 27:201–6.

23. Ulrich TA, De Juan Pardo EM, Kumar S. The mechanical rigidity of the extracellular matrix regulates the structure, motility, and proliferation of glioma cells. Cancer Res. 2009; 69:4167–74.

24. Ridley AJ. Rho GTPases and cell migration. J. Cell Sci. 2001; 114:2713–22.

25. Etienne-Manneville S, Hall A. Rho GTPases in Cell Biology. Nature. 2002; 420:629–35.

26. Ridley AJ. Cell Migration: Integrating Signals from Front to Back. Science. 2003; 302:1704–9.

27. Ornitz D, Marie P. FGF signaling pathways in endochondral and intramembranous bone development and human genetic disease. Genes Dev. 2002; 16:1446–65.

28. Fakhry A, Ratisoontorn C, Vedhachalam C, Salhab I, Koyama E, Leboy P, et al. Effects of FGF-2/-9 in calvarial bone cell cultures: Differentiation stage-dependent mitogenic effect, inverse regulation of BMP-2 and noggin, and enhancement of osteogenic potential. Bone. 2005; 36:254–66.

29. Sato K, Suematsu A, Nakashima T, Takemoto-Kimura S, Aoki K, Morishita Y, et al. Regulation of osteoclast differentiation and function by the CaMK-CREB pathway. Nat. Med. 2006; 12:1410–6.

30. Niimura Y, Nei M. Evolutionary dynamics of olfactory and other chemosensory receptor genes in vertebrates. J. Hum. Genet. 2006; 51:505–17.

31. Nei M, Niimura Y, Nozawa M. The evolution of animal chemosensory receptor gene repertoires: roles of chance and necessity. Nat. Rev. Genet. 2008; 9:951–63.

32. Lopez C, Raper J. Cloning and functional characterization of odorant receptors expressed in the zebrafish olfactory system. FASEB J. 2015; 29:727–37.

33. Niimura Y. On the origin and evolution of vertebrate olfactory receptor genes: comparative genome analysis among 23 chordate species. Genome Biol. Evol. 2009; 1:34–44.

34. Lindemann B. Receptors and transduction in taste. Nature. 2001; 413:219–25.

35. Chandrashekar J, Mueller KL, Hoon MA, Adler E, Feng L, Guo W, et al. T2Rs function as bitter taste receptors. Cell. 2000; 100:703–11.

36. Nelson G, Chandrashekar J, Hoon MA, Feng L, Zhao G, Ryba NJ, et al. An amino-acid taste receptor. Nature. 2002; 416:199–202.

37. Li R, Fan W, TianG, Zhu H, He L, Cai J, et al. The sequence and *de novo* assembly of the giant panda genome. Nature.2010; 463:311–7.

38. Ferreira AHP, Marana SR, Terra WR, Ferreira C. Purification, molecular cloning, and properties of a β-glycosidase isolated from midgut lumen of *Tenebrio molitor* (Coleoptera) larvae. Insect Biochem. Mol. Biol. 2001; 31:1065–76.

39. Tokuda G, Saito H, Watanabe H. A digestive β-glucosidase from the salivary glands of the termite, *Neotermes koshunensis* (Shiraki): Distribution, characterization and isolation of its precursor cDNA by 5’- and 3’-RACE amplifications with degenerate primers. Insect Biochem. Mol. Biol. 2002; 32:1681–9.

40. Sakamoto K, Uji S, Kurokawa T, Toyohara H. Molecular cloning of endogenous β-glucosidase from common Japanese brackish water clam *Corbicula japonica*. Gene. 2009; 435:72–9.

41. Zhu L, Wu Q, Dai J, Zhang S, Wei F. Evidence of cellulose metabolism by the giant panda gut microbiome. Proc. Natl. Acad. Sci. USA. 2011; 108:17714–9.

42. Bird NC, Mabee PM. Developmental morphology of the axial skeleton of the zebrafish, *Danio rerio* (Ostariophysi: Cyprinidae). Dev. Dyn. 2003; 228:337–57.

43. Ortega N, Behonick DJ, Werb Z. Matrix remodeling during endochondral ossification. Trends Cell Biol. 2004; 14:86–93.

44. Chen G, Deng C, Li YP. TGF-β and BMP signaling in osteoblast differentiation and bone formation. Int. J. Biol. Sci. 2012; 8:272–88.

45. Harada S, Rodan GA. Control of osteoblast function and regulation of bone mass. Nature. 2003; 423:349–55.

46. Long F. Building strong bones: molecular regulation of the osteoblast lineage. Nat. Rev. Mol. Cell Biol. 2011; 13:27–38.

47. Boyle WJ, Simonet WS, Lacey DL. Osteoclast differentiation and activation. Nature. 2003; 423:337–42.

48. Li H, Durbin R. Fast and accurate short read alignment with Burrows-Wheeler transform. Bioinformatics. 2009; 25:1754–60.

49. Grabherr MG, Haas BJ, Yassour M, Levin JZ, Thompson DA, Amit I, et al. Trinity: reconstructing a full-length transcriptome without a genome from RNA-Seq data . Nat. Biotechnol. 2011; 29:644–52.

50. Benson G. Tandem repeats finder: A program to analyze DNA sequences. Nucleic Acids Res. 1999; 27:573–80.

51. Jurka J, Kapitonov VV, Pavlicek A, Klonowski P, Kohany O, Walichiewicz J. Repbase Update, a database of eukaryotic repetitive elements. Cytogenet. Genome Res. 2005; 110:462–7.

52. Griffiths-Jones S, Moxon S, Marshall M, Khanna A, Eddy SR, Bateman A. Rfam: Annotating non-coding RNAs in complete genomes. Nucleic Acids Res. 2005; 33:121–4.

53. Birney E, Clamp M, Durbin R. GeneWise and Genomewise. Genome Res. 2004; 14:988–95.

54. Trapnell C, Pachter L, Salzberg SL. TopHat: Discovering splice junctions with RNA-Seq. Bioinformatics. 2009; 25:1105–11.

55. Trapnell C, Williams BA, Pertea G, Mortazavi A, Kwan G, Van Baren MJ, et al. Transcript assembly and quantification by RNA-Seq reveals unannotated transcripts and isoform switching during cell differentiation. Nat. Biotechnol. 2010; 28:511–5.

56. Elsik CG, Mackey AJ, Reese JT, Milshina NV, Roos DS, Weinstock GM. Creating a honey bee consensus gene set. Genome Biol. 2007; 8:R13.

57. Bairoch A, Apweiler R. The SWISS-PROT protein sequence database and its supplement TrEMBL in 2000. Nucleic Acids Res. 2000; 28:45–8.

58. Ashburner M, Ball CA, Blake JA, Botstein D, Butler H, Cherry JM, et al. Gene ontology: tool for the unification of biology. Nat. Genet. 2000; 25:25–9.

59. Kanehisa M, Goto S. KEGG: Kyoto encyclopedia of genes and genomes. Nucleic Acids Res. 2000; 28:27-30.

60. Lowe TM, Eddy SR. tRNAscan-SE: A program for improved detection of transfer RNA genes in genomic sequence. Nucleic Acids Res. 1997; 25:955–64.

61. Nawrocki EP, Kolbe DL, Eddy SR. Infernal 1.0: Inference of RNA alignments. Bioinformatics. 2009; 25:1335–7.

62. Baird NA, Etter PD, Atwood TS, Currey MC, Shiver AL, Lewis ZA, et al. Rapid SNP discovery and genetic mapping using sequenced RAD markers. PloS One. 2008; 3:e3376.

63. Li R, Li Y, Fang X, Yang H, Wang J, Kristiansen K, et al. SNP detection for massively parallel whole-genome resequencing. Genome Res. 2009; 19:1124–32.

64. Li R, Yu C, Li Y, Lam TW, Yiu SM, Kristiansen K, et al. SOAP2: An improved ultrafast tool for short read alignment. Bioinformatics. 2009; 25:1966–7.

65. Grattapaglia D, Sederoff R. Genetic linkage maps of Eucalyptus grandis and Eucalyptus urophylla using a pseudo-testcross: Mapping strategy and RAPD markers. Genetics. 1994; 137:1121–37.

66. Li H, Coghlan A, Ruan J, Coin LJ, Heriche JK, Osmotherly L, et al. TreeFam: a curated database of phylogenetic trees of animal gene families. Nucleic Acids Res. 2006; 34:D572–80.

67. Edgar RC. MUSCLE: Multiple sequence alignment with high accuracy and high throughput. Nucleic Acids Res. 2004; 32:1792–7.

68. Guindon S, Dufayard JF, Lefort V, Anisimova M, Hordijk W, Gascuel O. New algorithms and methods to estimate maximum-likelihood phylogenies: Assessing the performance of PhyML 3.0. Syst. Biol. 2010; 59:307–21.

69. Guindon S, Gascuel O. A simple, fast, and accurate algorithm to estimate large phylogenies by maximum likelihood. Syst. Biol. 2003; 52:696–704.

70. Yang Z. PAML 4: Phylogenetic analysis by maximum likelihood. Mol. Biol. Evol. 2007; 24:1586–91.

71. Yang Z, Rannala B. Bayesian estimation of species divergence times under a molecular clock using multiple fossil calibrations with soft bounds. Mol. Biol. Evol. 2006; 23:212–26.

72. Hahn MW, Demuth JP, Han SG. Accelerated rate of gene gain and loss in primates. Genetics. 2007; 177:1941–9.

73. Löytynoja A, Goldman N. An algorithm for progressive multiple alignment of sequences with insertions. Proc. Natl. Acad. Sci. USA. 2005; 102:10557–62.

74. Talavera G, Castresana J. Improvement of phylogenies after removing divergent and ambiguously aligned blocks from protein sequence alignments. Syst. Biol. 2007; 56:564–77.

75. Dawson AB. A note on the staining of the skeleton of cleared specimens with alizarin red S. Biotech. Histochem. 1926; 1:123-4.

76. Gruber HE. Adaptations of Goldner's Masson trichrome stain for the study of undecalcified plastic embedded bone. Biotech. Histochem. 1992; 67:30–4.

77. Ott HC, Matthiesen TS, Goh SK, Black LD, Kren SM, Netoff TI, et al. Perfusion-decellularized matrix: using nature's platform to engineer a bioartificial heart. Nat. Med. 2008; 14:213–21.

78. Langmead B, Salzberg SL. Fast gapped-read alignment with Bowtie 2. Nat. Methods. 2012; 9:357–9.

79. Li B, Dewey CN. RSEM: accurate transcript quantification from RNA-Seq data with or without a reference genome. BMC Bioinformatics. 2011; 12:323.

80. Mortazavi A, Williams BA, McCue K, Schaeffer L, Wold B. Mapping and quantifying mammalian transcriptomes by RNA-Seq. Nat. Methods. 2008; 5:621–8.

81. Robinson MD, Oshlack A. A scaling normalization method for differential expression analysis of RNA-seq data. Genome Biol. 2010; 11:R25.

82. Anders S, Huber W. Differential expression analysis for sequence count data. Genome Biol. 2010; 11:R106.

83. Niimura Y. Evolutionary dynamics of olfactory receptor genes in chordates: interaction between environments and genomic contents. Hum. Genomics. 2009; 4:107–18.

84. Altschul S, Madden T, Schaffer A, Zhang J, Zhang Z, Miller W, et al. Gapped BLAST and PSI-BLAST: a new generation of protein database search programs. Nucleic Acids Res. 1997; 25:3389–402.

85. Kumar S, Nei M, Dudley J, Tamura K. MEGA: A biologist-centric software for evolutionary analysis of DNA and protein sequences. Brief. Bioinform. 2008; 9:299–306.

86. Caporaso JG, Lauber CL, Walters WA, Berg-Lyons D, Lozupone CA, Turnbaugh PJ, et al. Global patterns of 16S rRNA diversity at a depth of millions of sequences per sample. Proc. Natl. Acad. Sci. USA. 2011; 108:4516–22.

87. Edgar, RC. UPARSE: highly accurate OTU sequences from microbial amplicon reads. Nat. Methods. 2013; 10:996–8.

88. Liu H, Chen CH, Gao ZX, Min JM, Gu YM, Jian JB, et al. The draft genome of *Megalobrama amblycephala* reveals the development of intermuscular bone and adaptation to herbivorous diet. 2016. GigaScience Database.

Figure Legends

Figure 1 Global view of the *M. amblycephala* genome and syntenic relationship between *Ctenopharyngodon idellus*, *M. amblycephala* and *Danio rerio*. (**A**) Global view of the *M. amblycephala* genome. From outside to inside, the genetic linkage map (a); Anchors between the genetic markers and the assembled scaffolds (b); Assembled chromosomes (c); GC content within a 50-kb sliding window (d); Repeat content within a 500-kb sliding window (e); Gene distribution on each chromosome (f); Different gene expression of three transcriptomes (g). (**B**) Syntenic relationship between *C. idellus* (a), *M. amblycephala* (b) and *D. rerio* (c) chromosomes.

Figure 2 Phylogenetic tree and comparison of orthologous genes in *M. amblycephala* and other fish species. (**A**) Phylogenetic tree of teleosts using 316 single copy orthologous genes. The color circles at the nodes shows the estimated divergence times using *O. latipes–F. rubripes* [96.9~150.9Mya], *F. rubripes–D. rerio* [149.85~165.2Mya], *F. rubripes–C. milii* [416~421.75Mya] (http://www.timetree.org/) as the calibration time. Pentagram represents four cyprinid fish with intermuscular bones. S, silurian period; D, devonian period; C, carboniferous period; P, permian periodin Paleozoic; T, triassic period; J, jurassic and k-cretaceous period in Mesozoic; Pg, paleogene in Cenozoic Era, N, Neogene. (**B**) Venn diagram of shared and unique orthologous gene families in *M. amblycephala* and four other teleosts. (**C**) Over-represented GO annotations of cyprinid-specific expansion gene families.

Figure 3 Regulation of genes related to intermuscular bone formation and function identified from developmental stages and adult tissues transcriptome data. (**A**) Gene expression pattern involved in muscle contraction regulated genes in early developmental stages corresponds to intermuscular bone formation of *M. amblycephala*, (alizarin red staining). M, myosepta; IB, intermusucular bone. (**B**) Scanning electron microscope photos of muscle tissues, connective tissues, and intermuscular bone. (**C**) Distribution of intermuscular bone specific genes in GO annotations indicative of abundance in protein binding, calcium ion binding, GTP binding functions. (**D**) Several developmental signals regulating key steps of osteoblast and osteoclast differentiation in the process of intramembranous ossification. Colored boxes indicate significantly up-regulated genes in these signals specifically occurred in intermuscular bone.

Figure 4 Molecular characteristics of sensory systems and the composition of gut microbiota in *M. amblycephala*. (**A**) Extensive expansion of olfactory receptor genes (ORs) in *M. amblycephala* compared with other teleosts. (**B**) Phylogeny of 'beta' type ORs in eight representative teleost species showing the significant expansion of 'beta' ORs in *M. amblycephala* and *C. idellus*. The pink background shows cyprinid-specific 'beta' types of ORs. (**C**) Umami, sweet and bitter tastes related gene families in teleosts with different feeding habits. (**D**) Structure of the umami receptor encoding T1R1 gene in cyprinid fish. (**E**) Relative abundance of microbial flora and taxonomic assignments in juvenile (LBSB), domestic adult (DBSB), wild adult (BSB) *M. amblycephala* and wild adult *C. idellus* (GC) samples at the phylum level.

Table

Table 1 Features of the *Megalobrama amblycephala* whole genome sequence

Total genome size (Mb)	1,116
N90 length of scaffold (bp)	20,422
N50 length of scaffold (bp)	838,704
N50 length of contig (bp)	49,400
Total GC content (%)	37.30
Protein-coding genes number	23,696
Average gene length (bp)	15,797
Content of transposable elements (%)	34.18
Number of chromosomes	24
Number of makers in genetic map	5,317
Scaffolds anchored on linkage groups (LGs)	1,434
Length of scaffolds anchored on LGs (Mb)	779.54 (70%)

Additional file 1

Click here to access/download
Supplementary Material
Additional file 1 Tables S1 to S17 and Figures S1 to S28.pdf

Additional file 2

Click here to access/download
Supplementary Material
Additional file 2 Data note1.xlsx

Additional file 3

Click here to access/download
Supplementary Material
Additional file 3 Data note2.xlsx
