# Supplementary material for: The draft genome of blunt snout bream (Megalobrama amblycephala) reveals the development of intermuscular bone and adaptation to herbivorous diet
Source: Gigascience. 2017 May 23;6(7):1–13. doi: 10.1093/gigascience/gix039 (PMC5570040; doi:10.1093/gigascience/gix039)
Supplement: Reviewer-2_Attachment-(Original-Submission).pdf [file gix039_Reviewer-2_Attachment-(Original-Submission).pdf]

REVIEW

The draft genome of *Megalobrama amblycephala* reveals the development of intermuscular bone and adaptation to herbivorous diet by Liu H., et al.

The present manuscript by Liu H., et al. can be divided into three main parts. The first part deals with the genome sequencing of one of the economically important cyprinid fish species *Megalobrama amblycephala.* Authors have generated a draft genome of 1.116 GB and managed to link 779.54 Mb to 24 linkage groups. Linkage groups were also constructed in the present work. Authors further investigate comparative genomics and phylogenetic analysis using the generated data. The second part investigates the characteristics of the feeding strategy of *Megalobrama amblycephala* being an herbivore species. This comprised analysis of "expanded gene families" (this expression is rather unfortunate and not clear. It does not show which gene families finally were looked at and supplementary material like figure S11 does not say much) as well as the gut microbial community, but no differential expression analysis (mRNA). In the method section the experimental set up and analysis of the gut microbial study is missing. The third part studies the development of intermuscular bones comprising the analysis of "expanded gene families" (again figure 2B does not explain the "expanded gene families") and transcriptome analysis of early developmental stages.

Authors present a huge amount of data and analysis but finally do describe and discuss only a little part of their analysis mainly in form of a very small set of genes.

The manuscript is well, but not straightforward written. The structure has to be revised and the outcome better worked out.

INTRODUCTION:

LINE 64: Today many genomes (draft and nearly complete) are available. It is suggested to categorize them into fresh water, Mediterranean and Atlantic sea important aquacultured species. One species e.g. important for the Mediterranean aquaculture, The European sea bass (*Dicentrarchus labrax*) which was recently sequenced is not listed (European sea bass genome and its variation provide insights into adaptation to euryhalinity and speciation. Nature communications, 2014 5, 5770.)

LINE 68-71: Please re-phrase the sentence. It is not true that the focus of aquaculture is focusing on herbivorous species. It is true however, that the usage of alternative feed is pursued but not the culture of herbivorous species. In contrast several projects are working on new species (including carnivores) for aquaculture purposes.

LINE 79 and LINE 84-85: Please refer to Wan et al., 2016 "Dynamic mRNA and miRNA expression analysis in response to intermuscular bone development of blunt snout bream (*Megalobrama amblycephala*)", Scientific Reports.

DATA DESCRIPTION

Description of generated SNP linkage map appears here, but does not appear in the method section. Please accomplish the method section.

ANALYSES SECTION

According to GigaScience Authors guide: "This section should provide details of all of the experiments and analyses that are required to support the conclusions of the paper. The authors should make clear the goal of each analysis and state the basic findings″

Information about analyses (except the linkage map analysis) is partially found in the section named "Results" as well as in the "Method" section.

LINES147-153: It is not clear why the enrichment analyses shows that the adaptation to herbivory goes "hand in hand" with the coping with plant secondary metabolites. Do authors have a comparable analysis of a carnivore or omnivore teleosts?

LINE 170: What do authors mean by the expression "comparative transcriptome analysis"?

LINE 174: "notable". This result is not really surprising. Many other transcriptome studies in teleost have also shown that at the later stages, where the larvae is mainly growing, mostly muscle genes are up-regulated when compared to earlier stages.

LINE 183: change the expression "eventually extended".

LINE 203. The link to Figure 3D is not clear. Is Figure 3D showing all 35 identified genes?

DISCUSSION

Mainly a repetition of the previous paragraphs.

LINE 134 and 271: Authors did not show that Dre10 and Dre22 are 'ancestral', just that those two chromosomes fused to one chromosome in blunt snout bream as this species has 24 chromosomes while zebrafish has 25 chromosomes. Please take into account publications like Nakatani, Y., H. Takeda, Y. Kohara, and S. Morishita, 2007 Reconstruction of the vertebrate ancestral genome reveals dynamic genome reorganization in early vertebrates. Genome Res. 17: 1254–1265 and. Hufton, A. L., D. Groth, M. Vingron, H. Lehrach, A. J. Poustka *et al.*, 2008 Early vertebrate whole genome duplications were predated by a period of intense genome rearrangement. Genome Res. 18: 1582–1591.

LINE 311: 'comparative transcriptome" This expression leads the reader to the false impression that more than one species was studied. However authors investigated here in differential expression.

METHODS

Missing description of microbial community study as well as generation of linkage map.
