# Supplementary material for: The draft genome of blunt snout bream (Megalobrama amblycephala) reveals the development of intermuscular bone and adaptation to herbivorous diet
Source: Gigascience. 2017 May 23;6(7):1–13. doi: 10.1093/gigascience/gix039 (PMC5570040; doi:10.1093/gigascience/gix039)
Supplement: Additional files [file gix039_Supp.zip › 3 Additional file 1 Tables S1 to S17 and Figures S1 to S28.pdf]

The draft genome of blunt snout bream (*Megalobrama amblycephala*) reveals the development of intermuscular bone and adaptation to herbivorous diet

Additional file 1

Supplementary Figures....1
Figure S1....1
Figure S2....2
Figure S3....1
Figure S4....2
Figure S5....3
Figure S6....4
Figure S7....5
Figure S8....6
Figure S9....7
Figure S10....8
Figure S11....9
Figure S12....10
Figure S13....11
Figure S14....12
Figure S15....13
Figure S16....14
Figure S17....15
Figure S18....16
Figure S19....17
Figure S20....18
Figure S21....19
Figure S22....20
Figure S23....21
Figure S24....22
Figure S25....23
Figure S26....24
Figure S27....25
Figure S28....26
Supplementary Tables....27
Table S1....27
Table S2....28
Table S3....29
Table S4....30
Table S5....31
Table S6....32

Table S7...33
Table S8...34
Table S9...35
Table S10...36
Table S11...38
Table S12...40
Table S13...41
Table S14...42
Table S15...43
Table S16...44
Table S17...45

Supplementary Figures

Figure S1

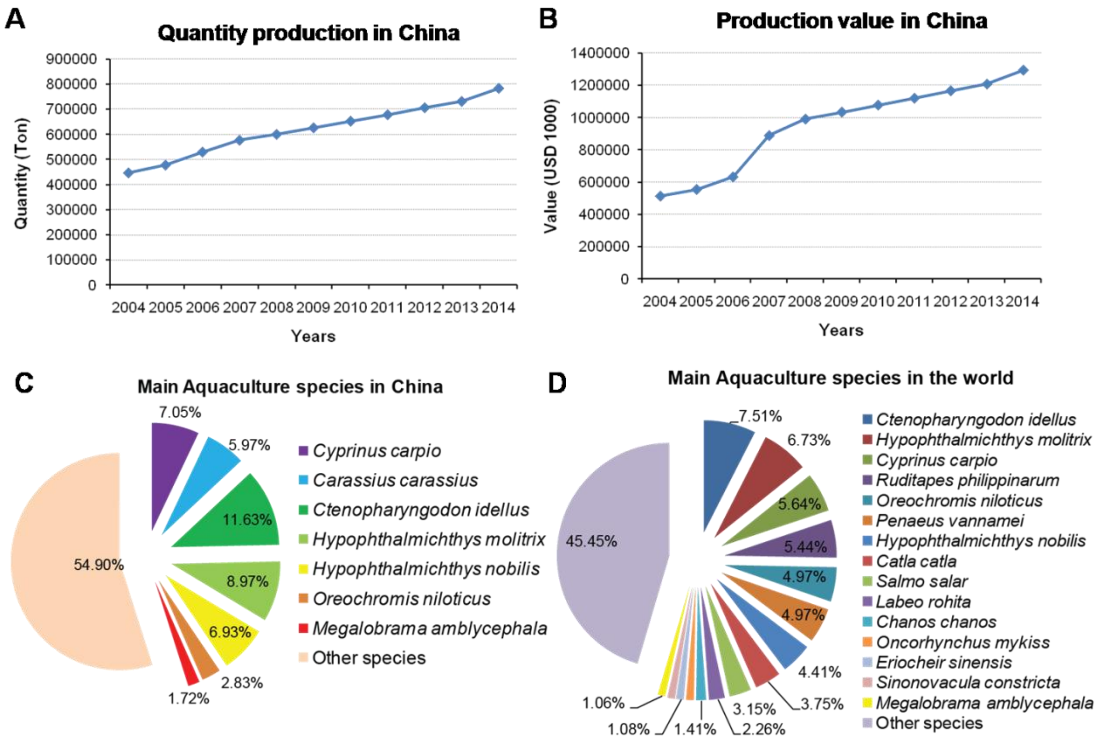

The quantity production and production value of *M. amblycephala.* (A) The quantity production and (B) the production value of *M. amblycephala* in China in the past few years. (C) The quantity production of *M. amblycephala* is 1.72% in China in 2014. (D) The main aquaculture species in the world in 2014.

Figure S2

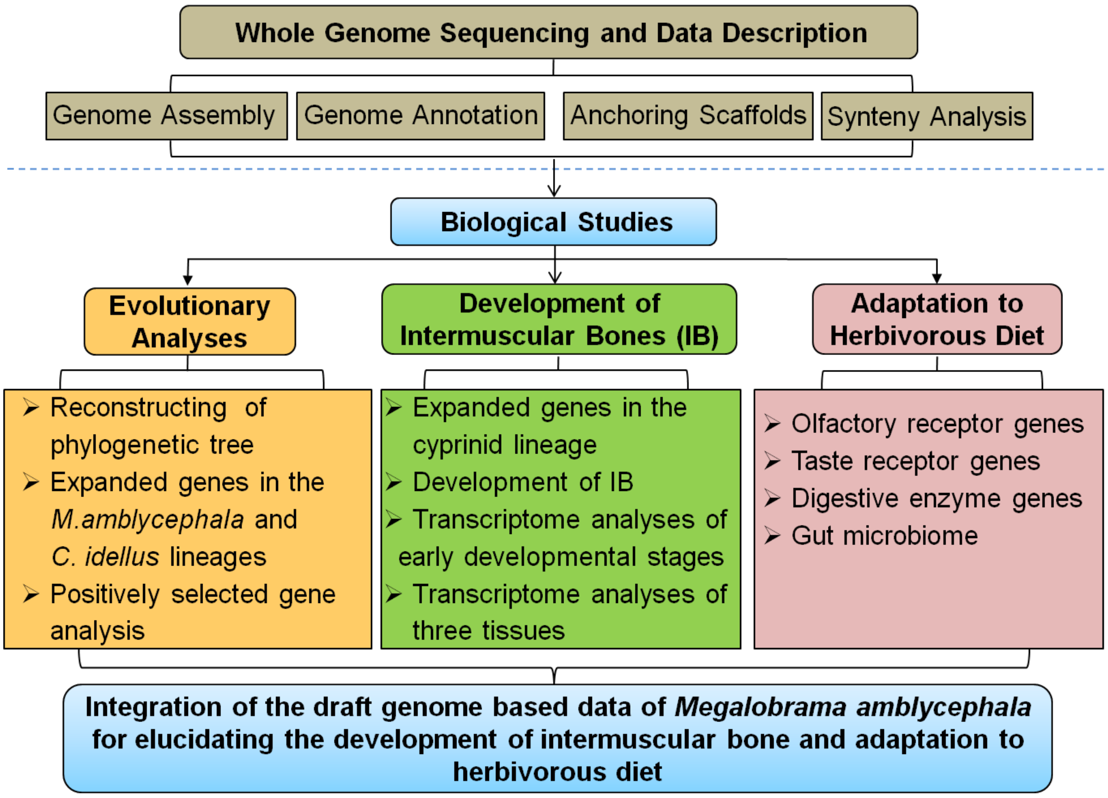

Schematic diagram illustrating the workflow of *M. Amblycephala* genome.

Figure S3

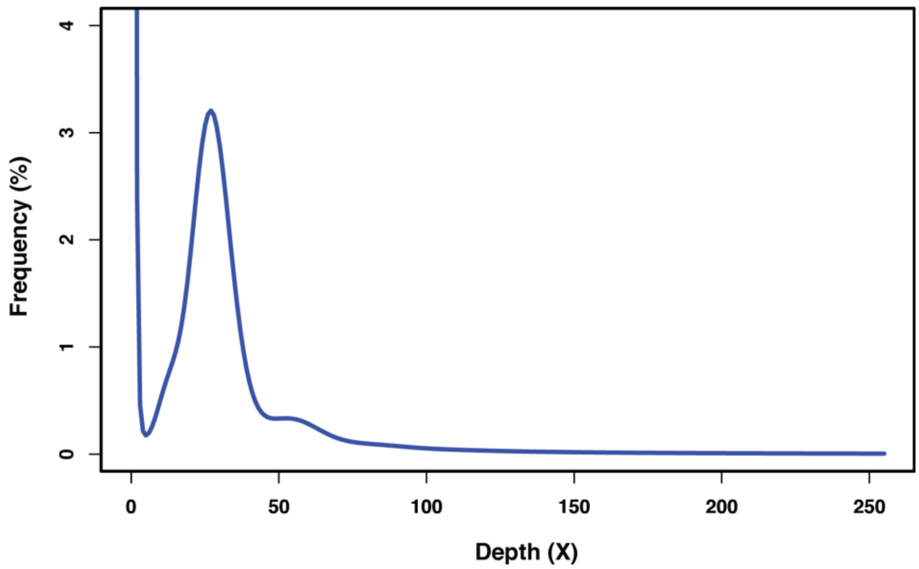

Estimation of the *M. amblycephala* genome size based on 17-mer analysis. The x-axis is Kmer depth; the y-axis is the Kmer proportion.

Figure S4

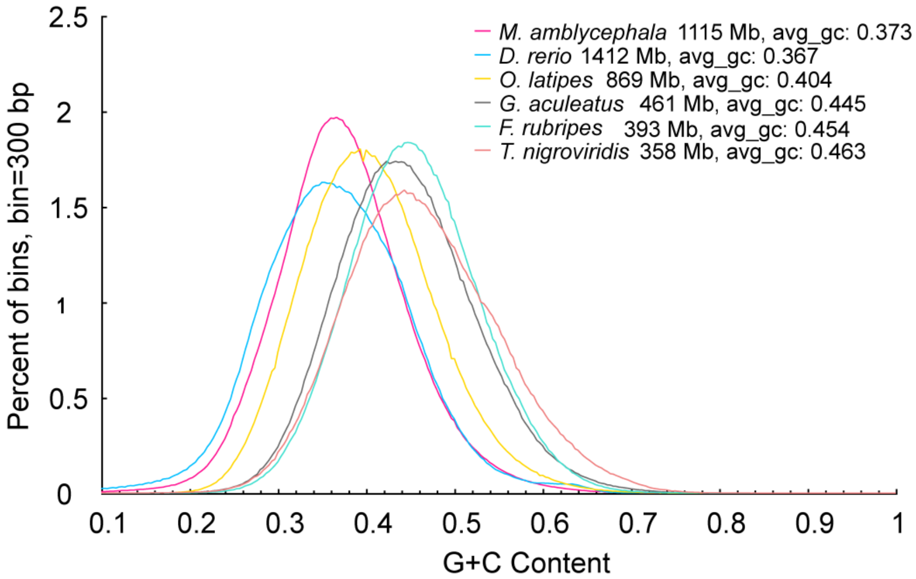

GC content in the genomes of *M. amblycephala* and five other sequenced teleosts. The x-axis is GC content and the y-axis is the proportion of the bins number divided by the total windows. We used 300bp bins (with 150bp overlap) sliding along the genome. According to this graph, we can compare the GC content distribution with the related species.

Figure S5

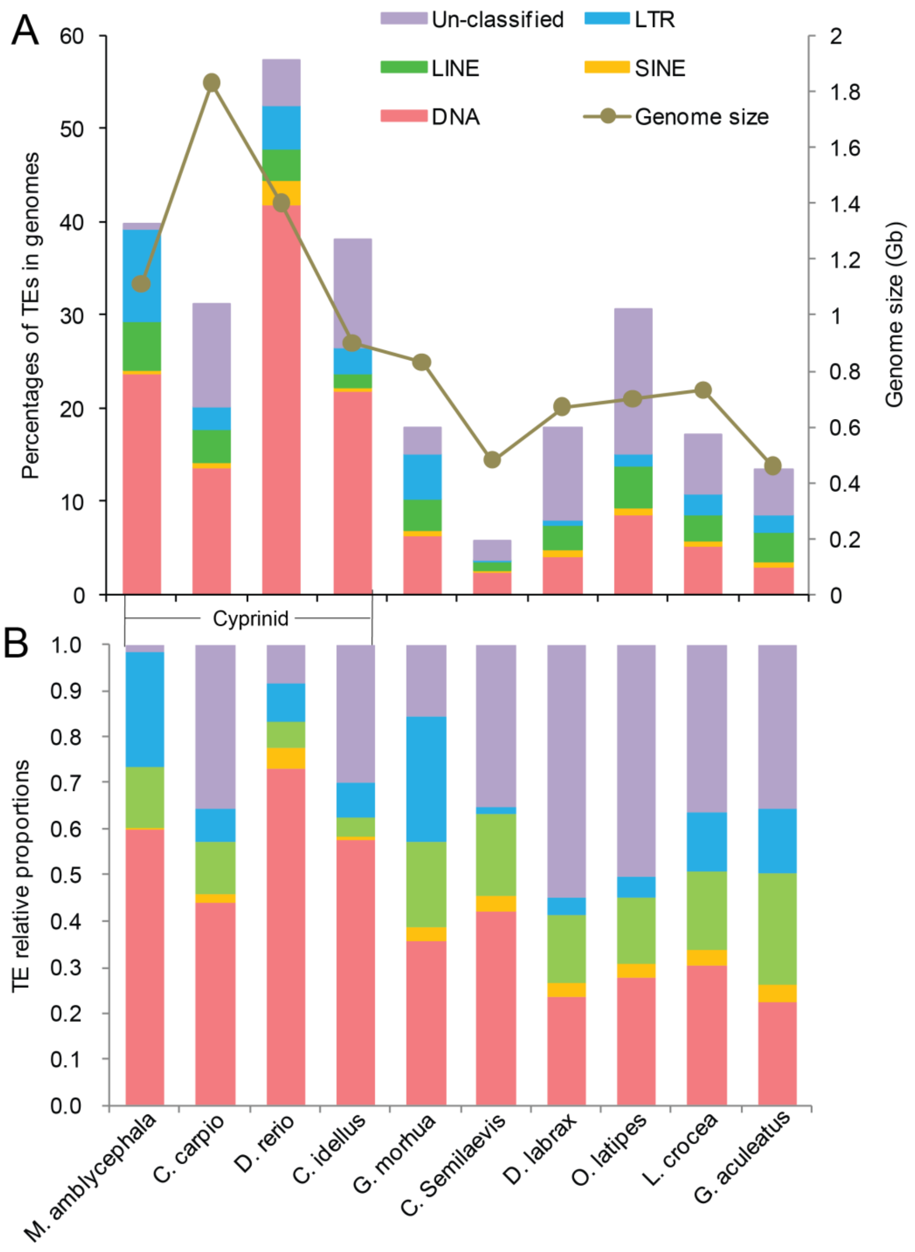

Comparison of the total proportions (A) and proportions of each TE class (B) of DNA transposons, LTR, LINE and SINE retrotransposons components of TEs in ten teleosts genomes.

Figure S6

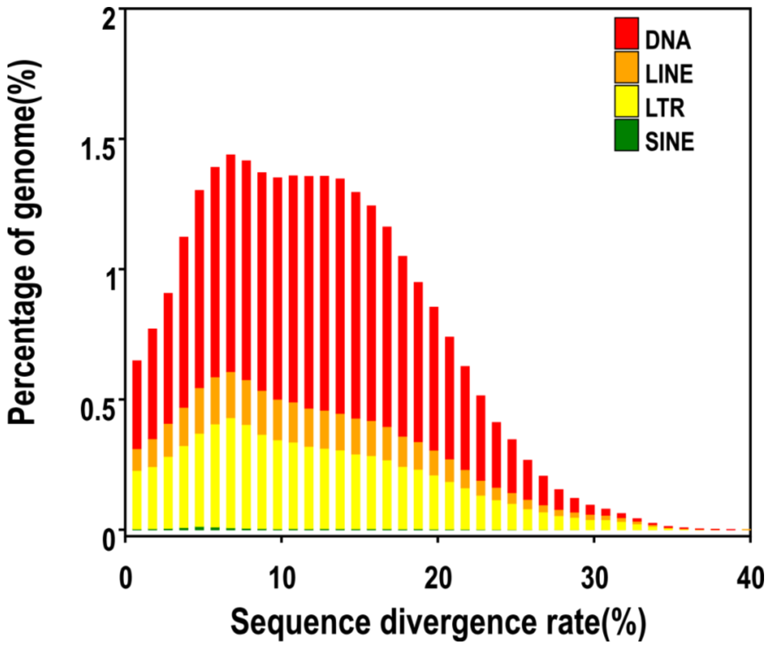

Distribution of divergence rate of TE in *M. amblycephala*. The divergence rate was calculated between the identified TE elements in the genome by *de novo* method and the consensus sequence in the predicted TE library.

Figure S7

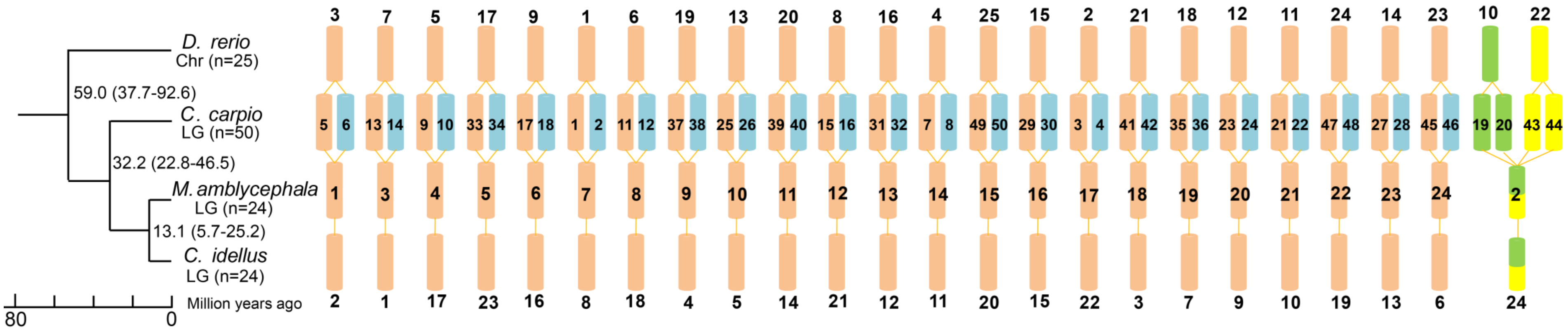

Syntenic relationship between *D. rerio* chromosomes, *C. carprio*, *C. idellus* and *M. amblycephala* linkage groups. *M. amblycephala* linkage group 2 and *C. idellus* linkage group 24 are aligned to *D. rerio* chromosome 10 and 22, *C. carprio* linkage group 19, 20, 43 and 44, respectively.

Figure S8

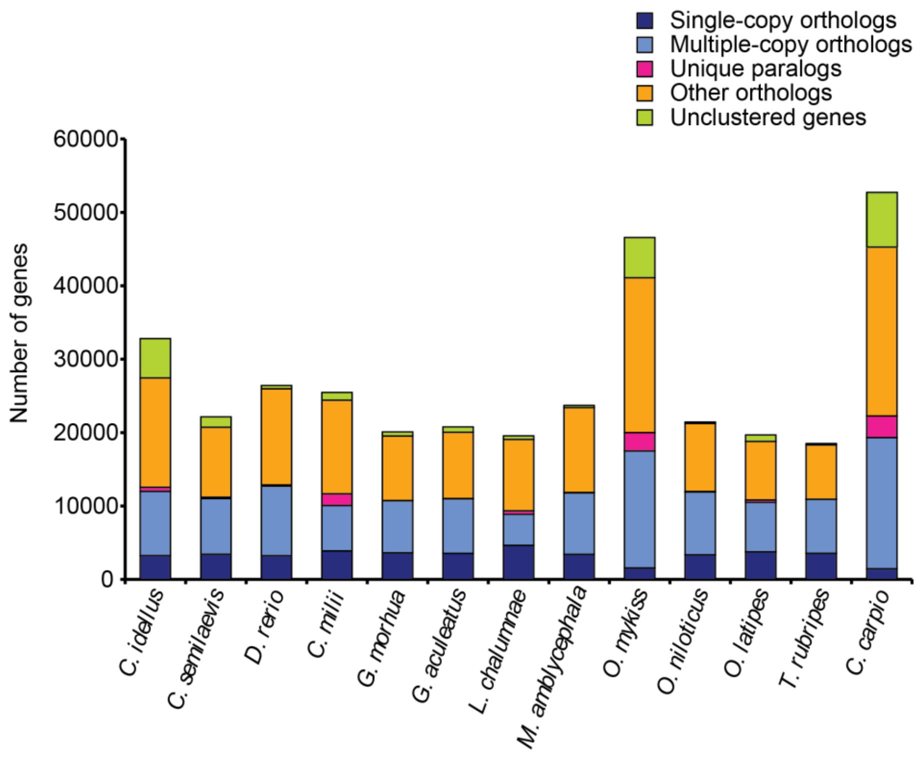

Statistics of orthologous families in 13 fish species.

Figure S9

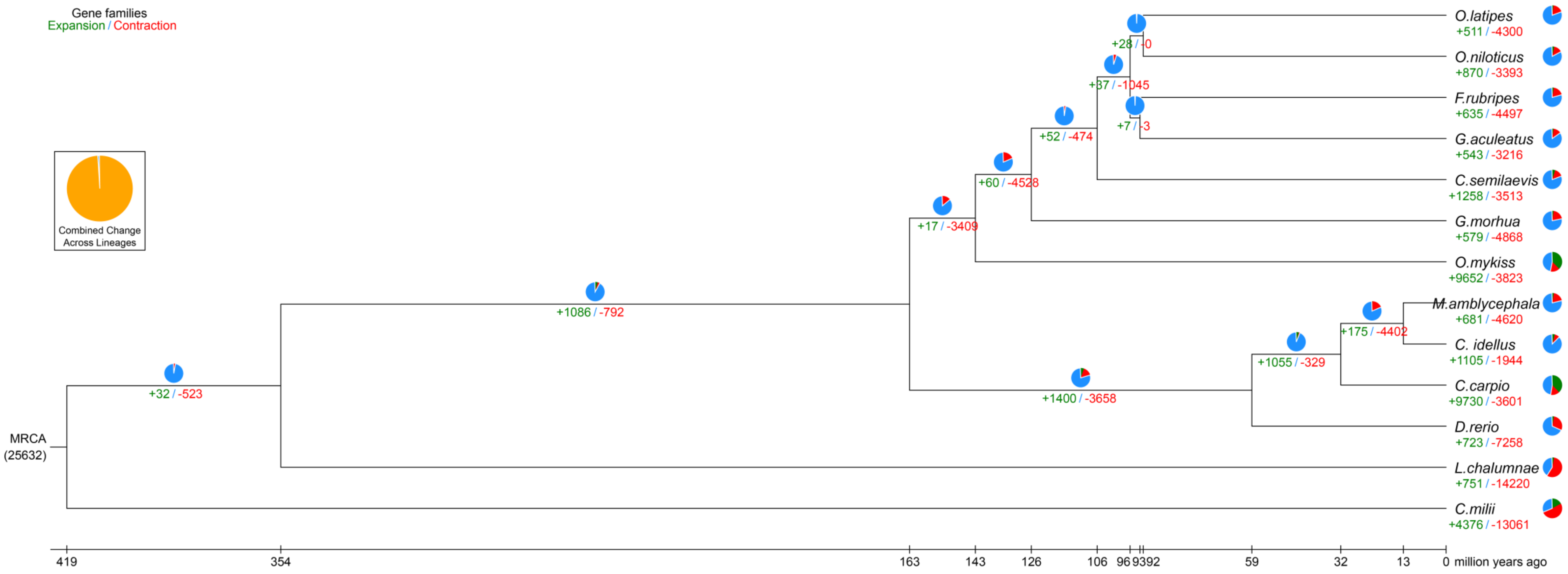

Statistical analysis of contraction and expansion of gene clusters.

Figure S10

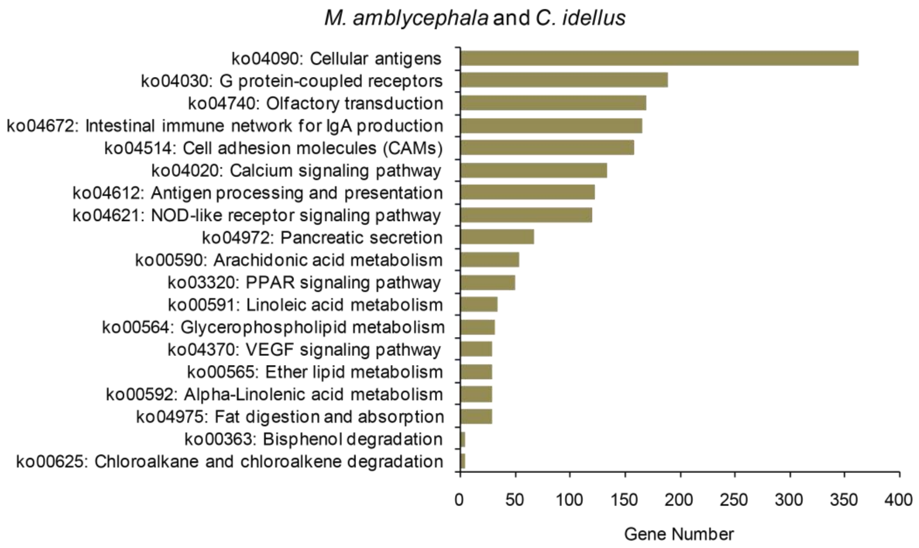

Over-represented KEGG pathways for *M. amblycephala* and *C. idellus* expanded gene families.

Figure S11

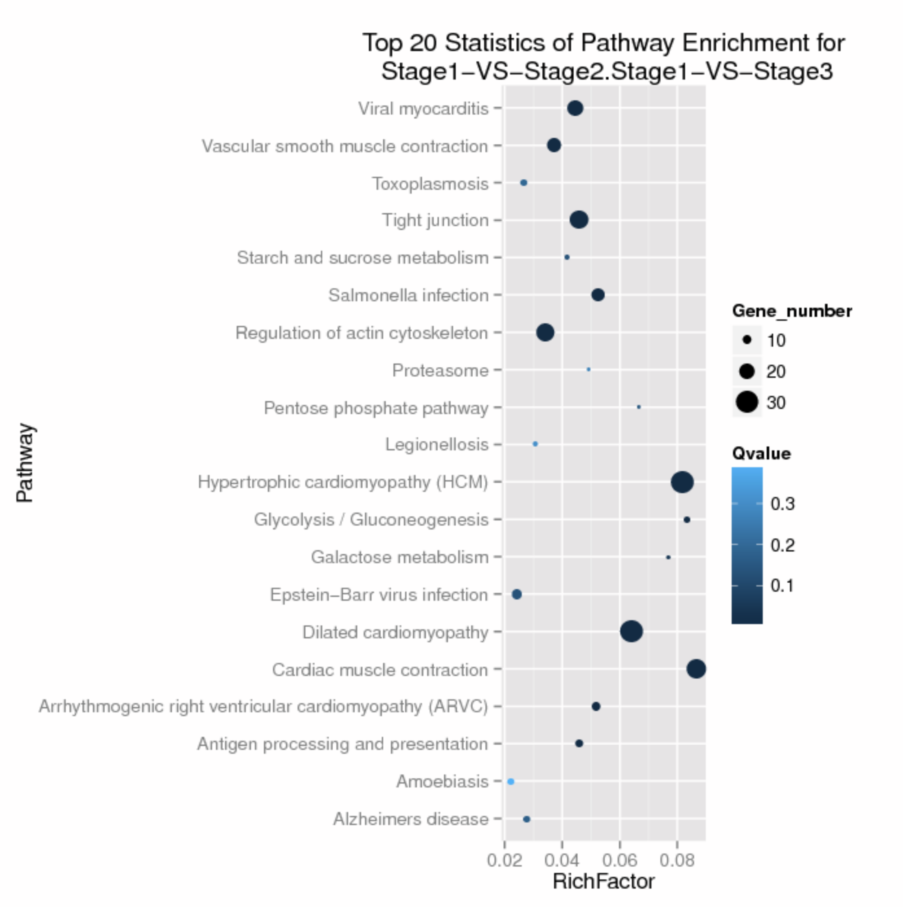
Top 20 Statistics of Pathway Enrichment for
Stage1−VS−Stage2.Stage1−VS−Stage3
Viral myocarditis
Vascular smooth muscle contraction
Toxoplasmosis
Tight junction
Starch and sucrose metabolism
Salmonella infection
Regulation of actin cytoskeleton
Proteasome
Pentose phosphate pathway
Legionellosis
Hypertrophic cardiomyopathy (HCM)
Glycolysis / Gluconeogenesis
Galactose metabolism
Epstein−Barr virus infection
Dilated cardiomyopathy
Cardiac muscle contraction
Arrhythmogenic right ventricular cardiomyopathy (ARVC)
Antigen processing and presentation
Amoebiasis
Alzheimers disease
Pathway
0.02
0.04
0.06
0.08
RichFactor
Gene_number
10
20
30
Qvalue
0.3
0.2
0.1

Figure S12

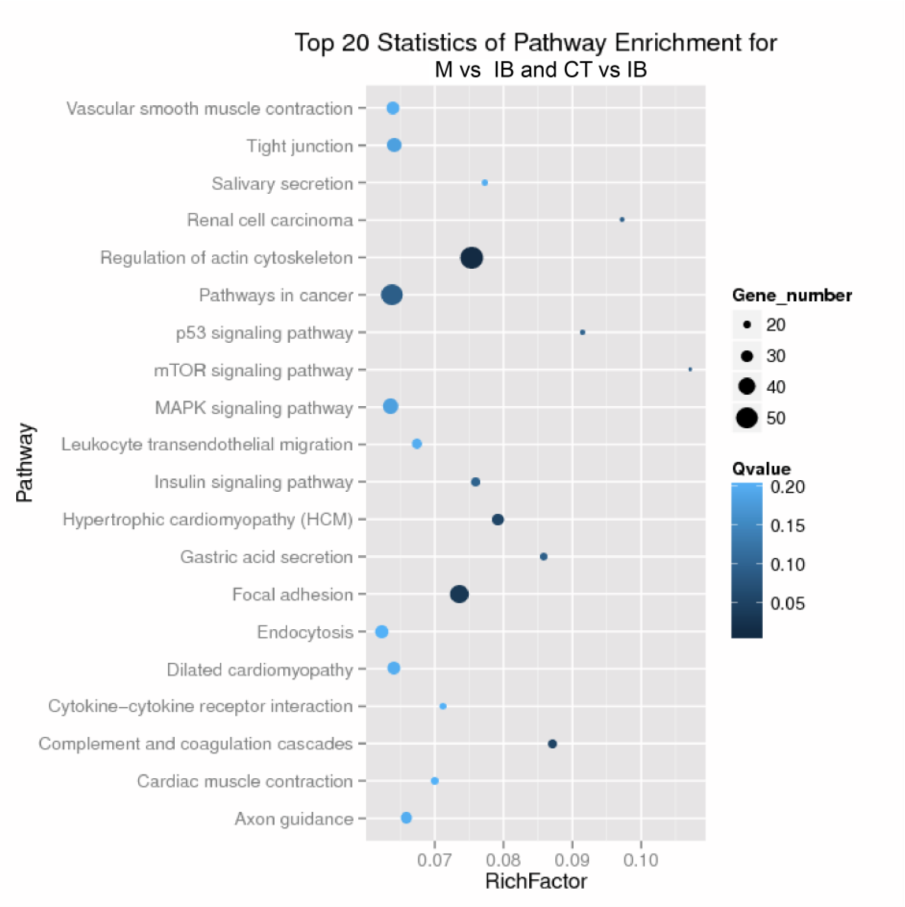

Top 20 Statistics of Pathway Enrichment for
M vs IB and CT vs IB
Vascular smooth muscle contraction
Tight junction
Salivary secretion
Renal cell carcinoma
Regulation of actin cytoskeleton
Pathways in cancer
p53 signaling pathway
mTOR signaling pathway
MAPK signaling pathway
Leukocyte transendothelial migration
Insulin signaling pathway
Hypertrophic cardiomyopathy (HCM)
Gastric acid secretion
Focal adhesion
Endocytosis
Dilated cardiomyopathy
Cytokine–cytokine receptor interaction
Complement and coagulation cascades
Cardiac muscle contraction
Axon guidance
Pathway
0.07
0.08
0.09
0.10
RichFactor
Gene_number
20
30
40
50
Qvalue
0.20
0.15
0.10
0.05

Figure S13

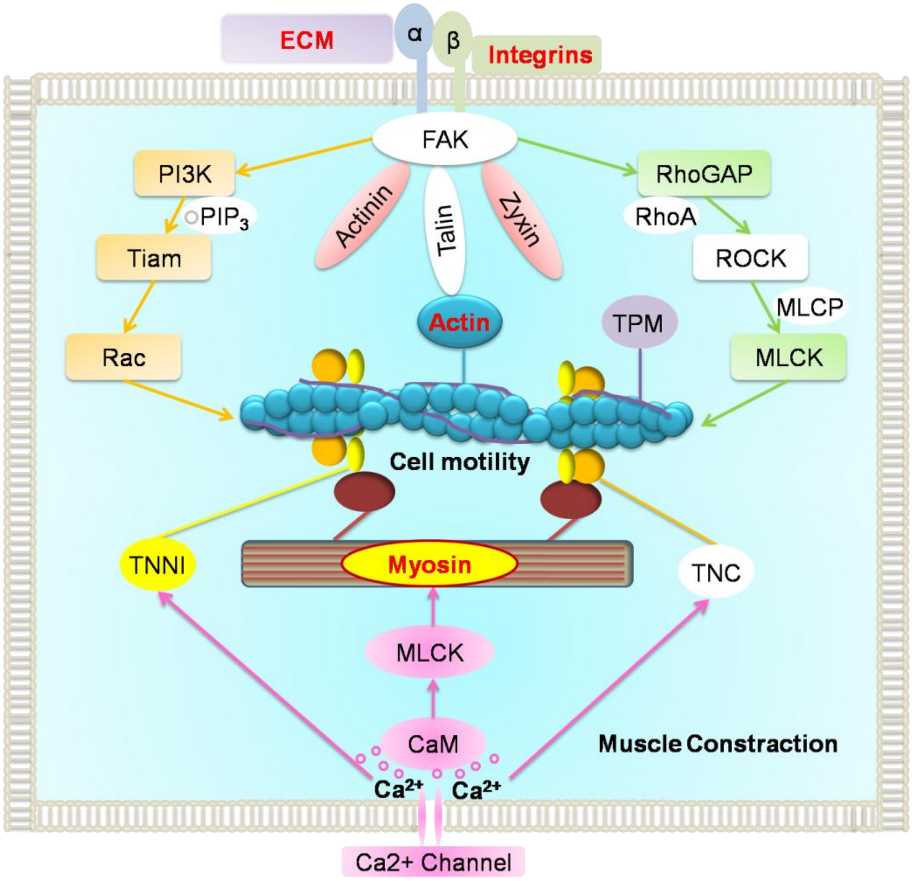

Summary of up-regulated genes in intermuscular bone (IB) in the KEGG pathways when compared with muscle (M) and connective tissues (CT). The up-regulated genes (color boxes) mainly involved in ‘cell motility’ and and ‘muscle constraction’. ECM, extracellular matrix protein; PIK3C, phosphatidylinositol-4,5-bisphosphate 3-kinase; Tiam, T-cell lymphoma invasion and metastasis; RAC, Ras-related C3 botulinum toxin substrate; RhoGAP, Rho GTPase-activating protein; RhoA, Ras homolog gene family; ROCK, Rho-associated kinase; MLCK, myosin-light-chain kinase; MLCP, MLC phosphatase; TPM, tropomyosin; TNNI, troponin I; TNC, tenascin; CaM, calmodulin.

Figure S14

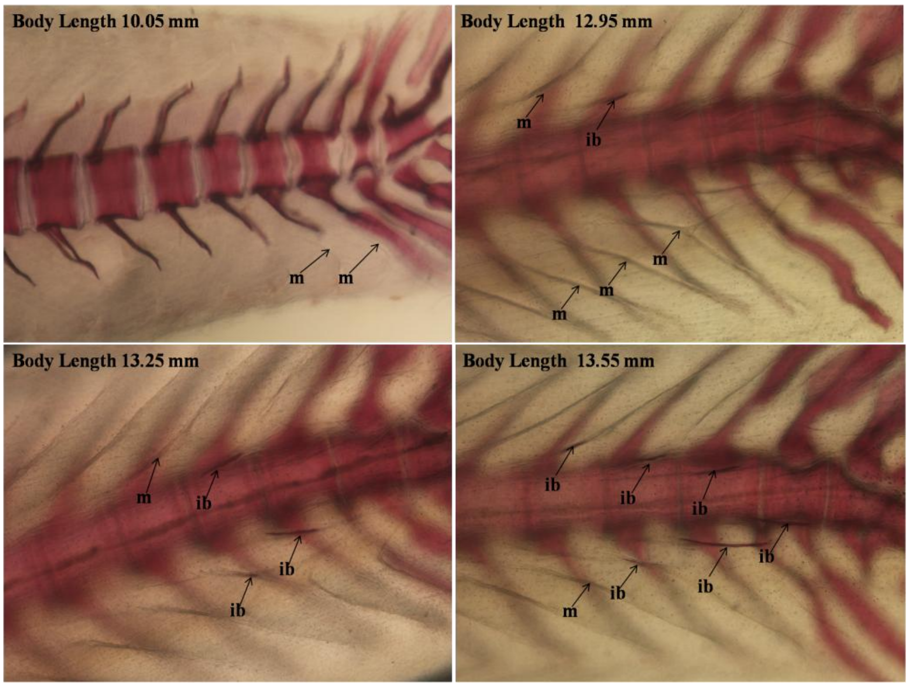

Developmental process of intermuscular bone (IB) in *M. amblycephala.* The first IB of *M. amblycephala* appeared in the caudal vertebra, and then ossified and developed form the caudal vertebra to trunk vertebra. Myosepta either not ossified yet, or poorly ossified was not visible with alizarin red staining. Ossified myosepta were stained red. m, myosepta; ib, intermuscular bone.

Figure S15

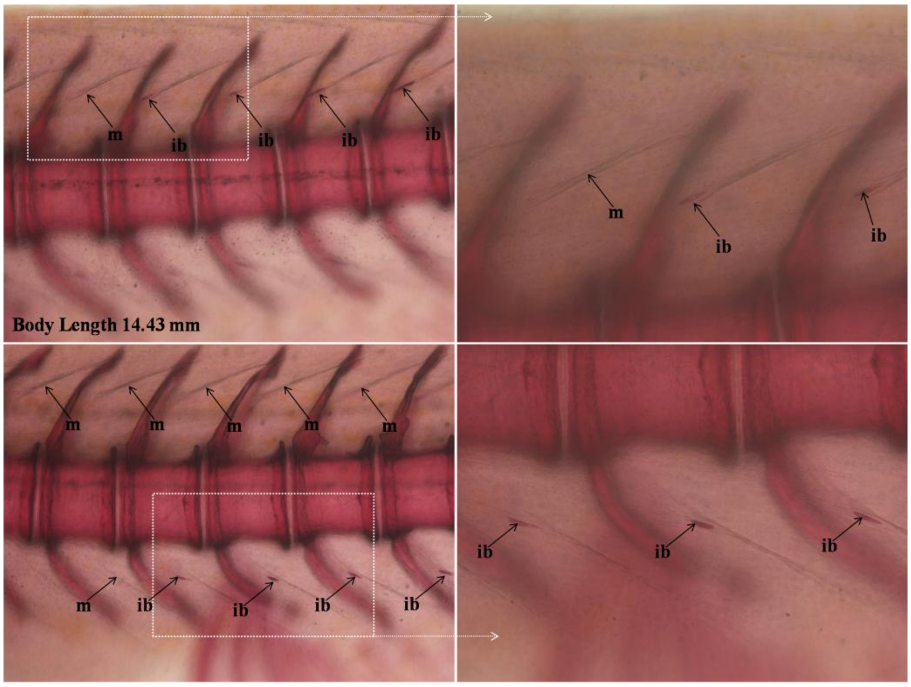

Intermuscular bone (IB) of *M. amblycephala* appeared in the trunk vertebra. Enlarged image focusing on the hypaxial IBs were ossified and lengthened from the myosepta.

Figure S16

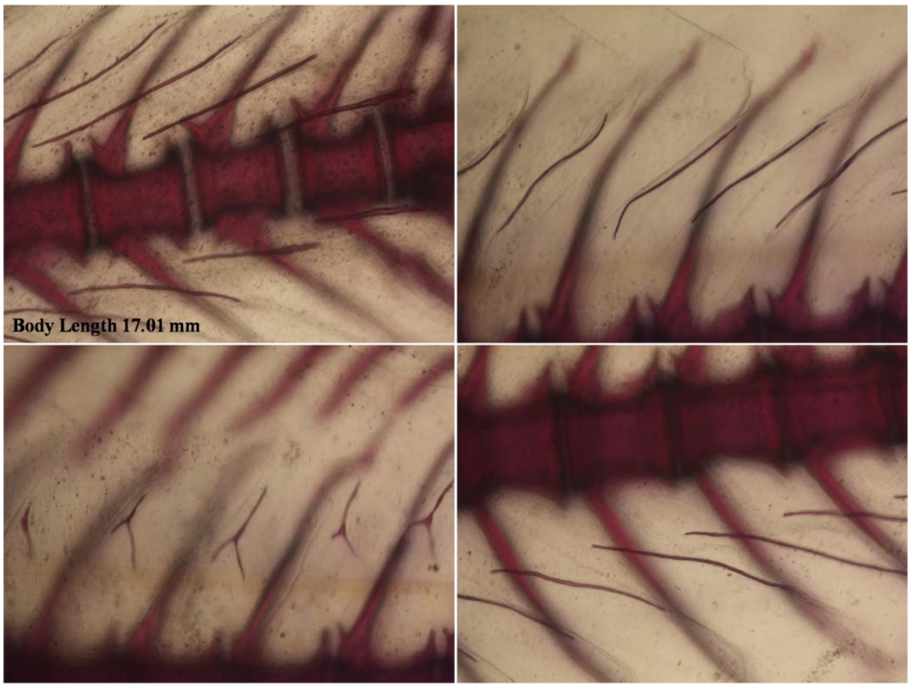

IB of *M. amblycephala* appeared in the whole body. The IBs were lengthening and widening and showed the Y-shaped appearance with the body length increasing.

Figure S17

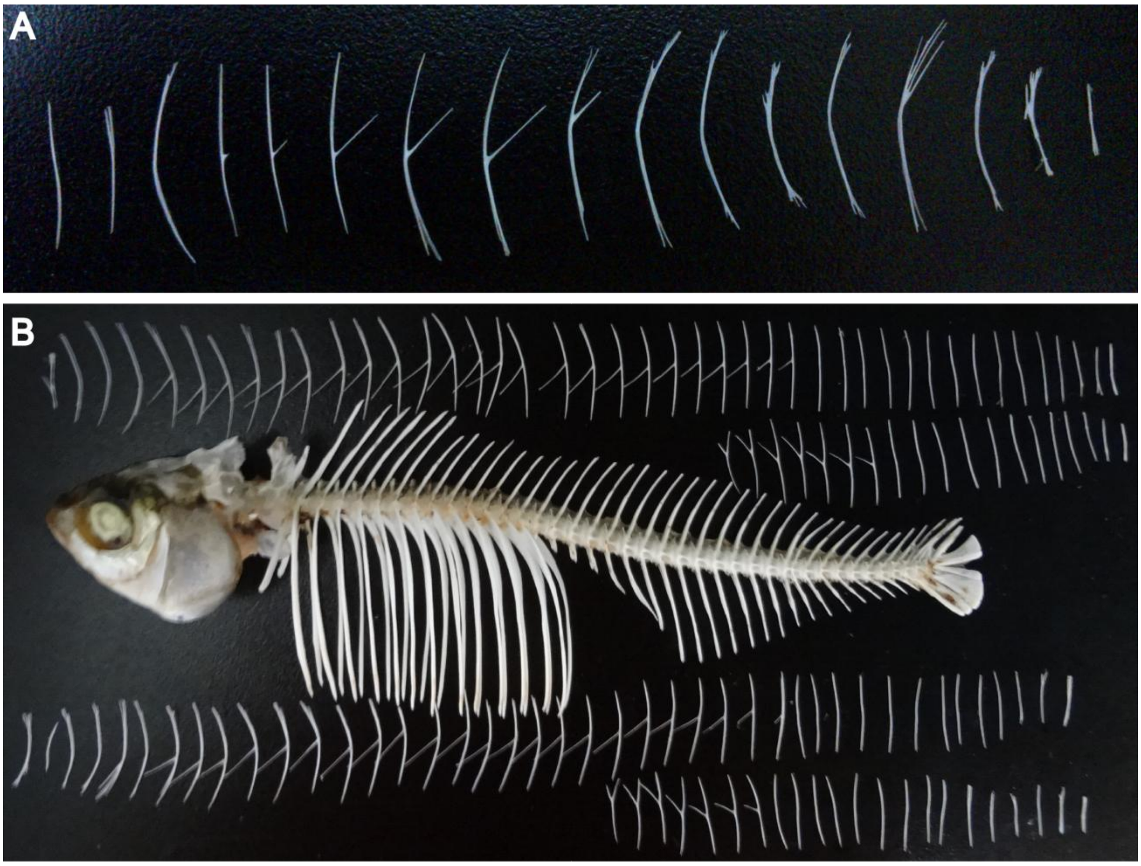

IB in adult *M. amblycephala*. (A) Different morphology types of IBs in adult *M. amblycephala*. (B) Distribution of IBs in adult *M. amblycephala*.

Figure S18

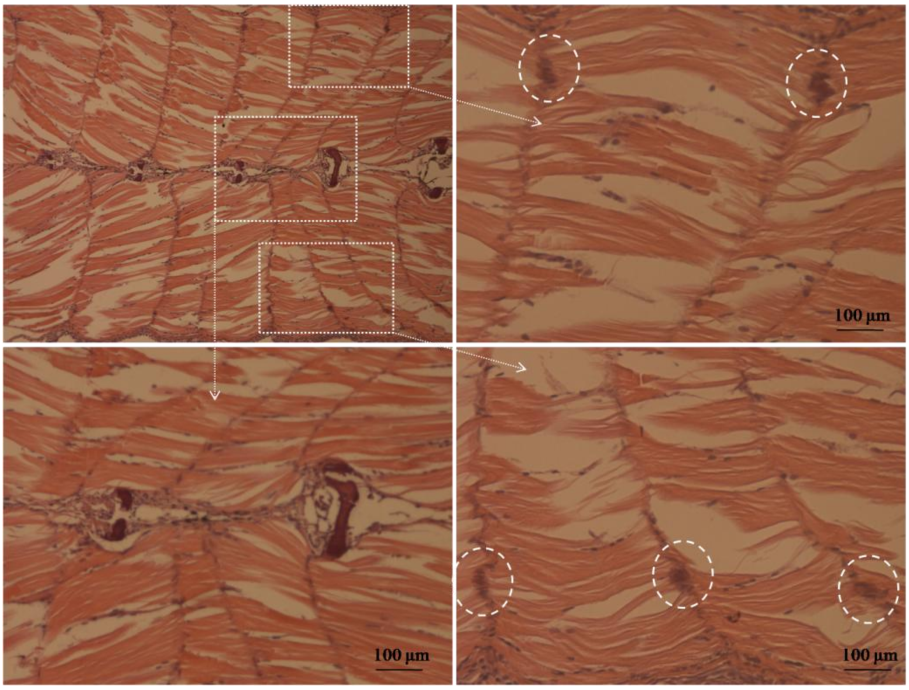

Histological analysis of IB. H&E staining of muscle showed red and rib and IB (white circle) showed deep red.

Figure S19

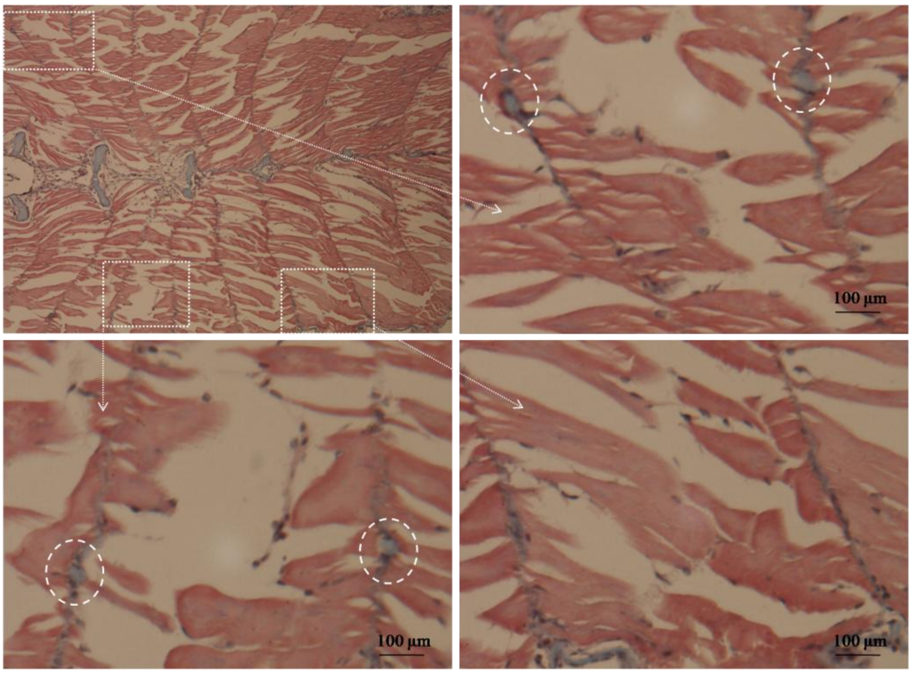

Masson trichrome staining analysis of IB and other tissues. Ribs and IB (white circle) were stained in blue.

Figure S20

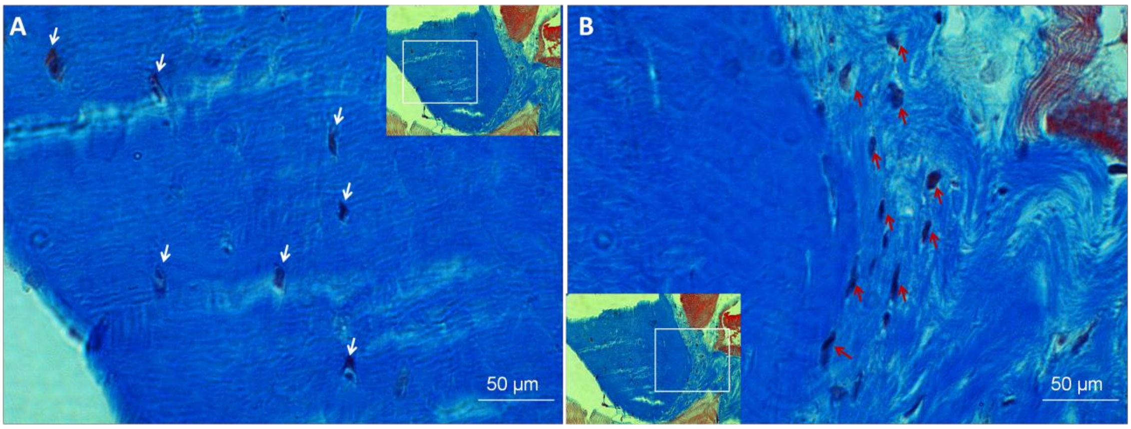

Masson trichrome staining of ossific intermuscular bone. A, White arrows showed the osteocytes; B, red arrows showed the osteoblasts.

Figure S21

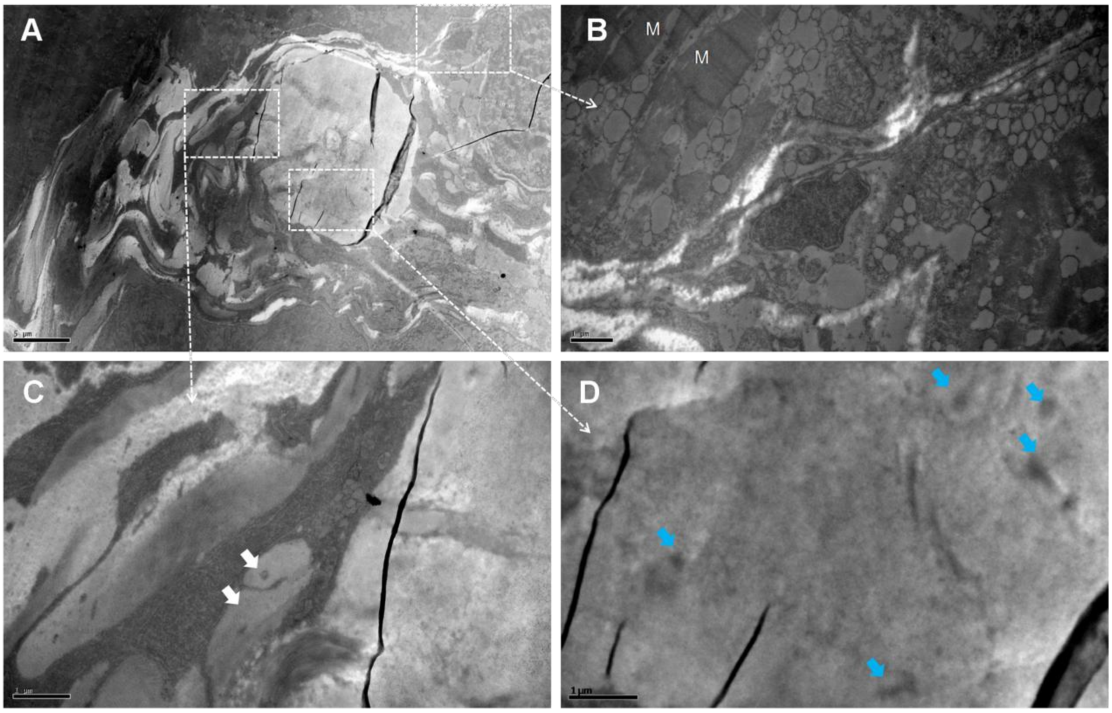

Transmission electron microscopy (TEM) of intermuscular bone embedded in the myosepta showed some osteoblasts (white arrows) distributed in the edge of the bone matrix and some osteocytes (blue arrows) distributed in the center of bone matrix. M, muscle tissues.

Figure S22

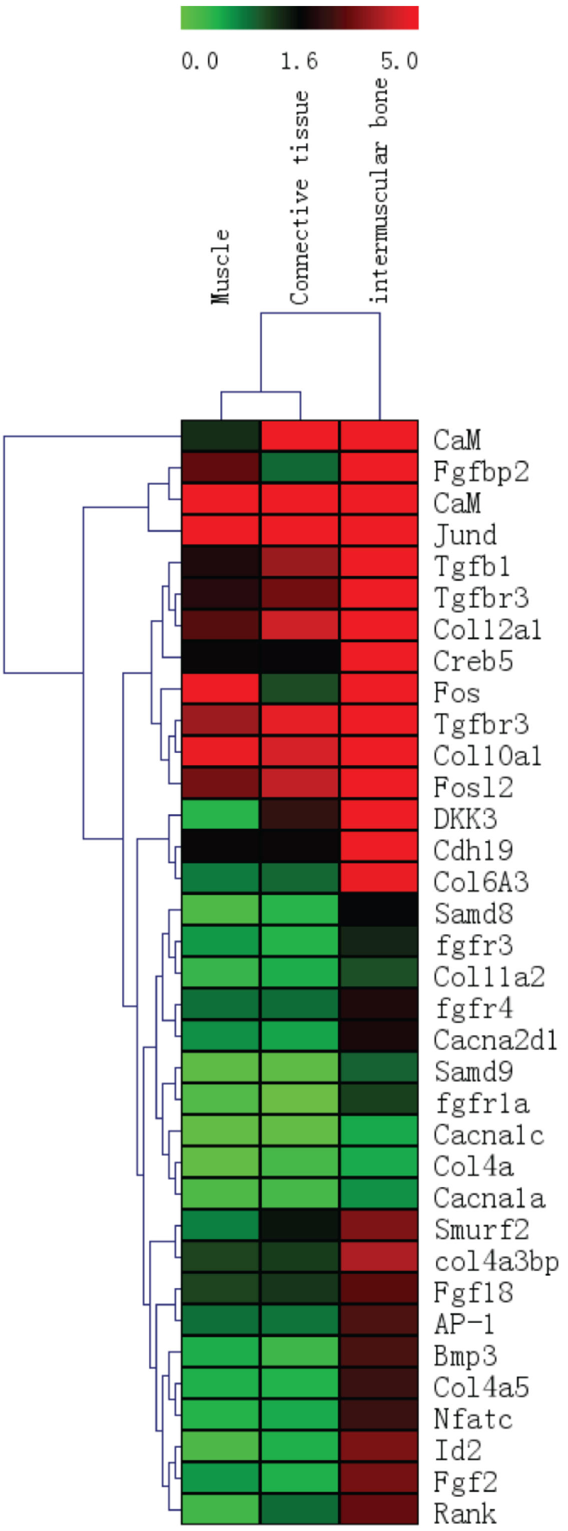

Heatmap shows the expression pattern of indentified 35 genes (shown in colored boxes of Figure 3D) involved in regulation process of intramembranous ossification.

Figure S23

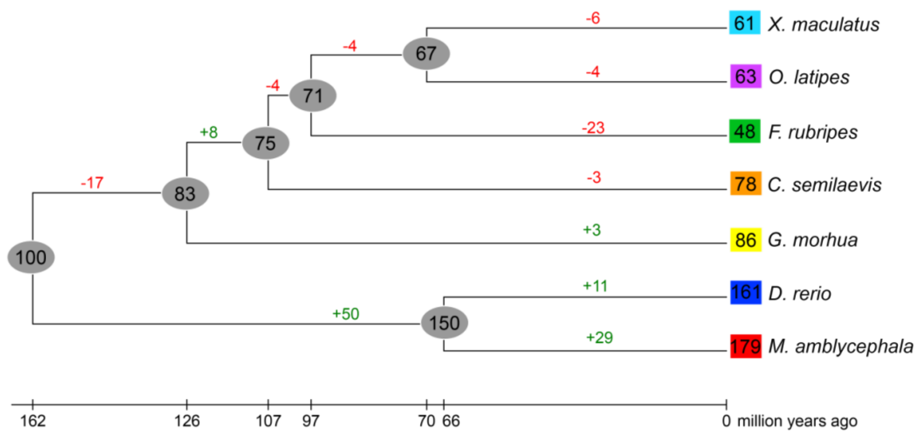

Expansion of olfactory receptor genes (ORs) in seven fish species. Numbers in the color boxes indicate the total number of ORs in each fish species. The number of OR gene in an ancestral species are showing in the gray ellipse at each node. The red numbers with minus signs and green numbers with plus signs on each branch indicate the gene losses and gains, respectively.

Figure S24

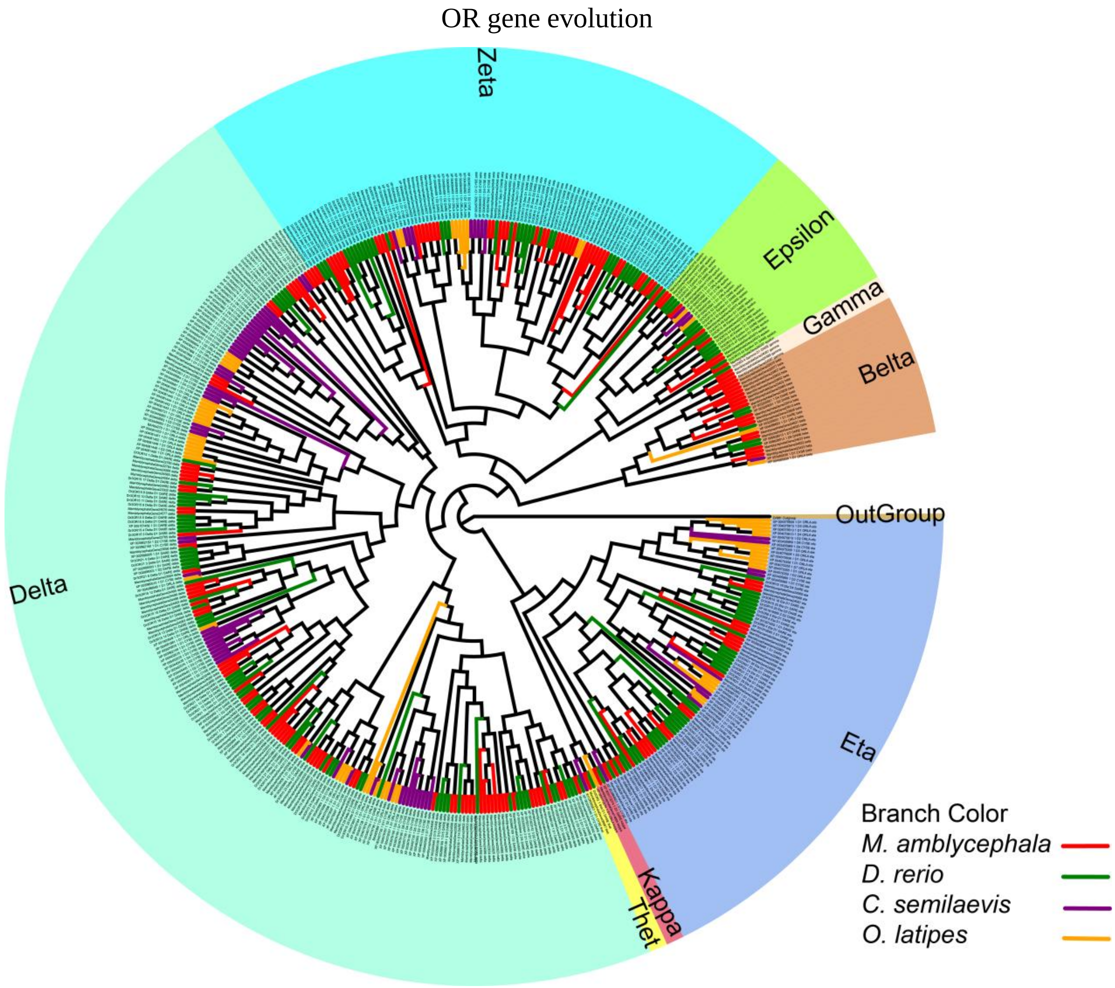

Neighbor joining tree of olfactory receptor genes (ORs) in *M. amblycephala*, *D. rerio*, *C. semilaevis*, *O. latipes*. A total of 481 sequences from above-mentioned fish genomes were used to construct the circular cladogram tree. Different OR types were distinguished by the different color labels and sequences from different species were distinguished by color branches.

Figure S25

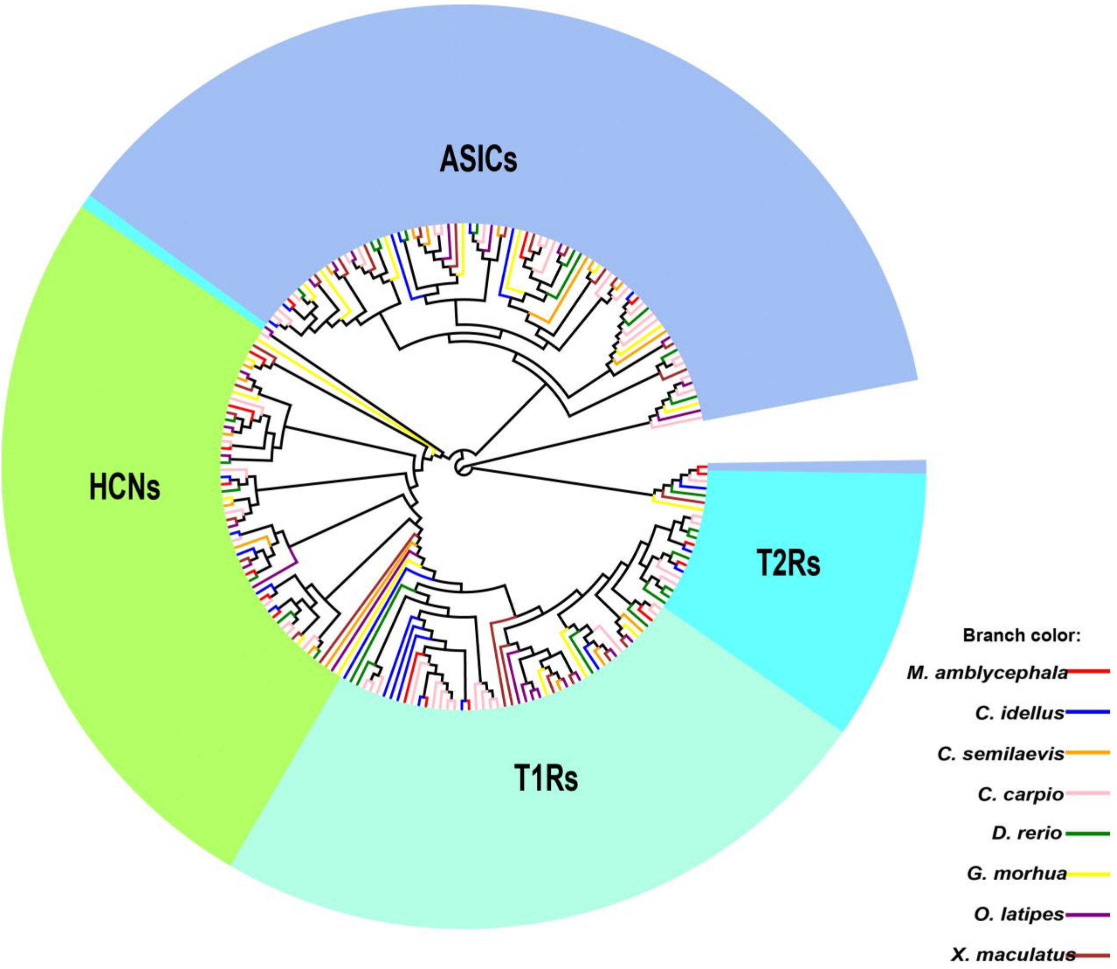

Neighbor joining tree of different taste receptors including bitter (T2Rs), sweet (T1R2/T1R3), umami (T1R1/T1R3) and sour (HCNs and ASICs) genes in eight teleosts.

Figure S26

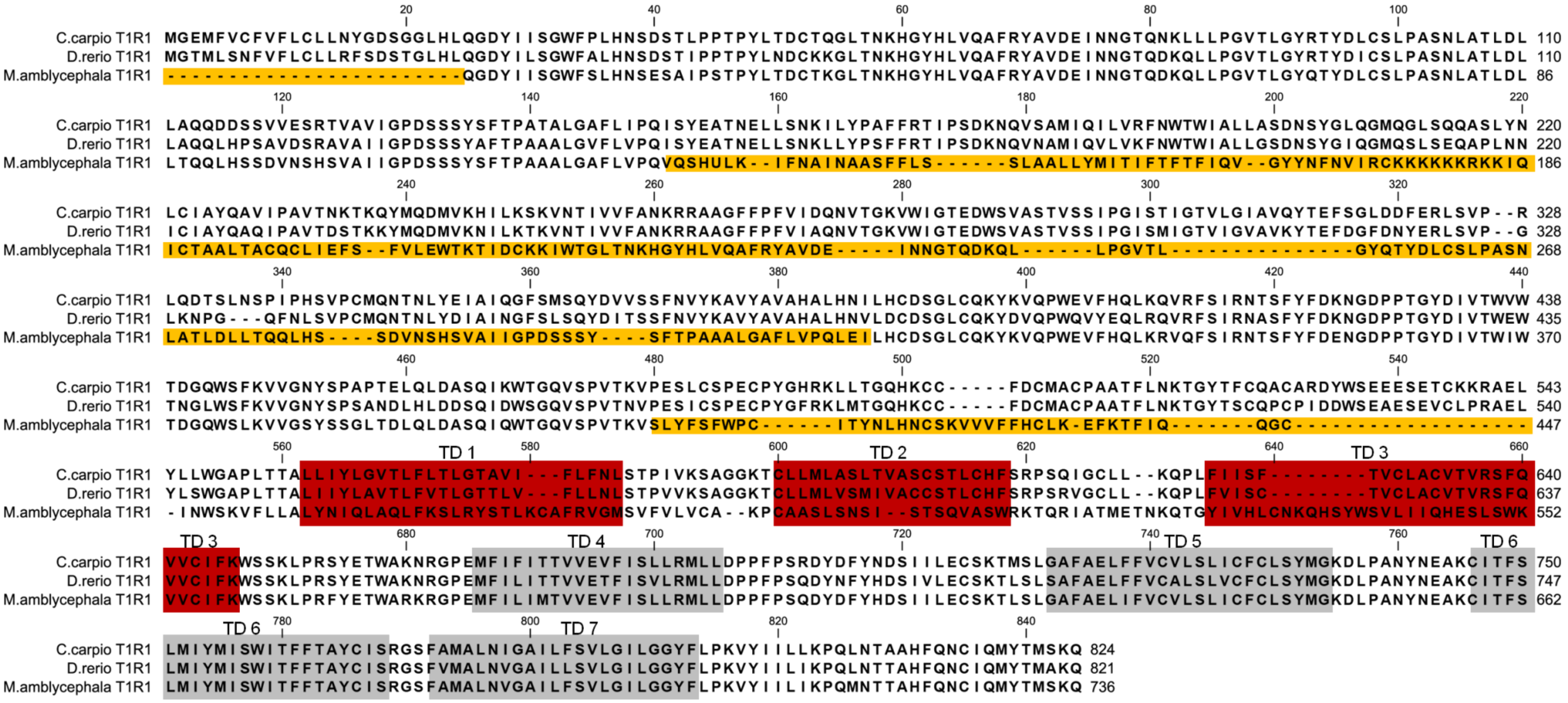

Multiple sequence alignment of T1R1 genes of *C. carpio, D. rerio* and *M. amblycephala*. Orange, amino acid changed in *M. amblycephala*; Red, transmembrane domains changed in *M. amblycephala*; Gray, other four transmembrane domains. TD represents transmembrane domain.

Figure S27

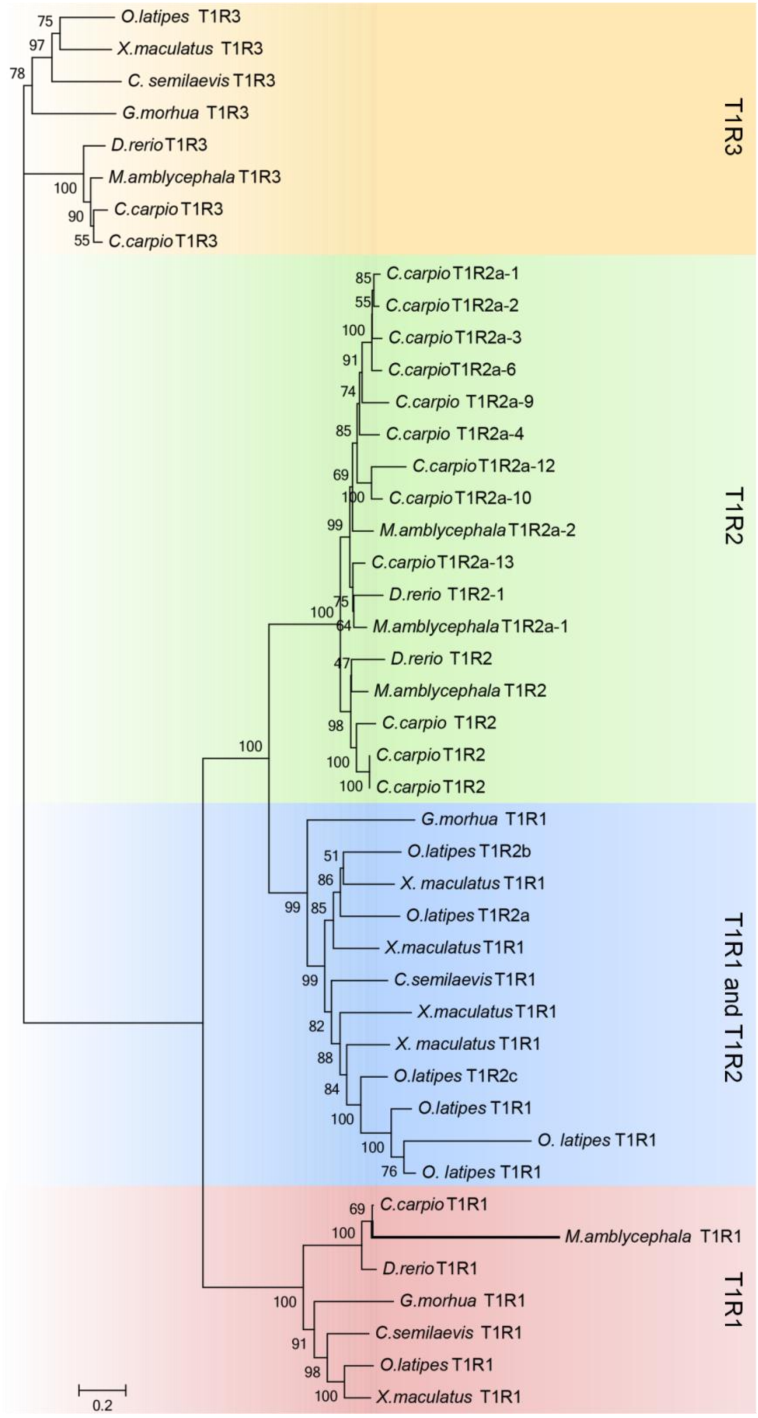

Phylogenetic analysis of T1Rs gene in teleosts. The tree describes the relationships of T1Rs genes among the seven teleosts. Branch bolded showed that the T1R1 gene sequence in *M. amblycephala* is not intact. Tree was constructed by MEGA 5 using NJ method. Numbers at branches indicate the bootstrap probabilities (≥90%) with 1,000 replicates.

Figure S28

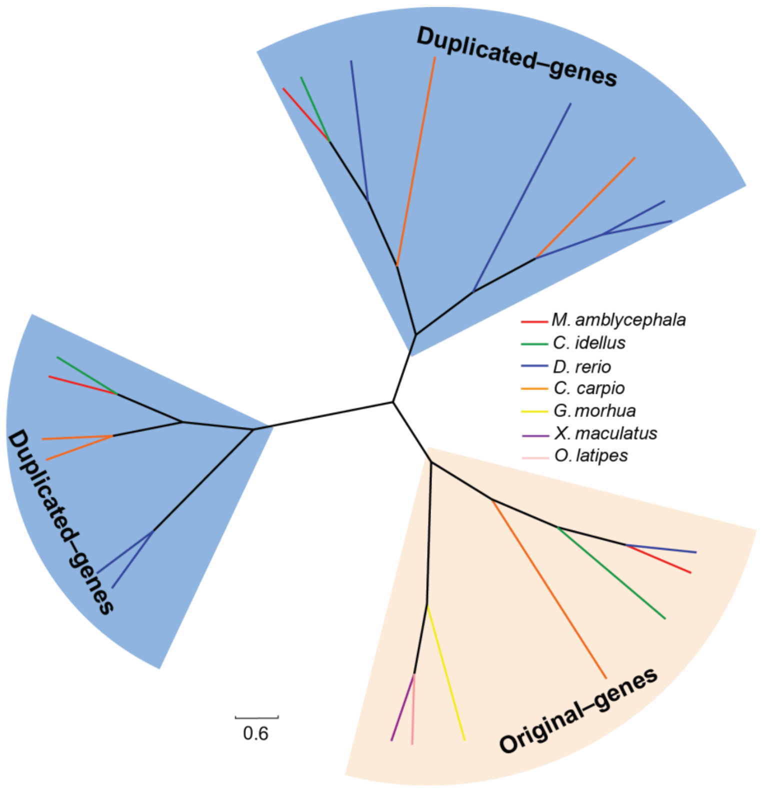

Phylogeny of T2R genes in seven representative fish species showing an expansion of T2R in *M. amblycephala* and other three cyprinid fishes *D. rerio*, *C. idellus* and *C. carpio*.

Supplementary Tables

Table S1

Details of the raw data and filter data of the *M. amblycephala* genome *de novo* assembly.

Insert size	Reads Length	Raw data			Filter Data		
		Total Data (Gb)	Sequence Depth (X)	Physical Depth (X)	Total Data (Gb)	Sequence Depth (X)	Physical Depth (X)
170bp	100_100	38.22	34.74	29.53	35.91	32.64	27.75
500bp	100_100	27.44	24.95	62.37	24.28	22.07	55.18
800bp	100_100	15.9	14.46	57.82	13.78	12.53	50.12
2Kb	49_49	38.01	34.56	705.26	26.48	24.07	491.2
5Kb	49_49	30.9	28.09	1433.21	19.6	17.81	908.92
10Kb	49_49	21.37	19.43	1982.57	9.72	8.83	901.33
20Kb	49_49	24.89	22.63	4618.6	12.79	11.62	2372.26
**Total**	**---**	**196.74**	**178.86**	**8889.37**	**142.55**	**129.59**	**4806.76**

Table S2

Genome size estimation based on 17-mer frequencies.

K	K-mer Num	Peak_Depth	Genome Size	Used Bases	Used Reads	X
17	30,152,065,104	27	1,116,743,152	35,895,315,600	358,953,156	32.14

Table S3

Reads mapping results of *M. amblycephala* genome.

Species	Genome size (bp)	Effective size (bp)	Covered base (bp)	Total reads# (M)	Mapped reads# (M)	Reads map (%)	Coverage (%)
M.amblycephala	1,115,678,790	1,082,731,470	1,068,773,627	428221.17	41187.48	96.2	98.7

Table S4

Assessment of the gene coverage with transcriptome data assembled by 454 sequencing.

Dataset	Number	Total Length (bp)	Base covered by Assembly (%)	Sequence covered by Assembly (%)	With >90% Sequence in one Scaffold		With >50% Sequence in one Scaffold	
					Number	Percent (%)	Number	Percent (%)
All	73675	28094321	89.79	94.98	54773	74.34	68988	93.64
>200bp	61756	26688607	90.40	96.76	48608	78.71	58967	95.48
>500bp	12415	6464440	64.77	68.21	7520	60.57	8345	67.22

Table S5

The result of assessing genome assembly completeness using BUSCO.

Number	Categories
2461	Complete BUSCOs
2313	Complete and single-copy BUSCOs
148	Complete and duplicated BUSCOs
276	Fragmented BUSCOs
286	Missing BUSCOs
3023	Total BUSCO groups searched

Table S6

Statistics of predicted protein-coding genes. CT, connective tissues; IB, intermuscular bone; M, muscle.

**Gene set**		**Number**	**Average gene length (bp)**	**Average CDS length (bp)**	**Average exon per gene**	**Average exon length (bp)**	**Average intron length (bp)**
De novo	AUGUSTUS	55,459	9,177.16	1,043.65	5.63	185.26	1,755.34
**Homolog**	*D. rerio*	38,985	8,430.79	1,237.26	5.92	209.13	1,463.19
	G. aculeatus	35,167	7,751.38	1,055.76	5.45	193.88	1,506.20
	O. niloticus	42,897	7,493.45	1,052.56	5.07	207.42	1,580.80
	O. latipes	38,148	6,761.52	1,022.98	5.03	203.42	1,424.37
	G. morhua	32,747	7,986.29	1,051.14	5.53	190.01	1,530.25
**Transcriptome**	CT	41,298	10,159.98	349.34	1.68	207.46	14,345.50
	IB	29,744	5,256.72	1,273.80	3.86	330.07	1,393.00
	M	20,187	5,542.25	1,196.30	4.18	286.43	1,368.14
**GLEAN**		23,696	15,797	1,637	9	186	1,812

Table S7

TE Content in the *M. amblycephala* Genome.

	**RepBase TE**		**TE Proteins**		***De novo***		**Combined TE**	
	**Length (bp)**	**% in Genome**	**Length (bp)**	**% in Genome**	**Length (bp)**	**% in Genome**	**Length (bp)**	**% in Genome**
DNA	136,648,377	12.25	18,323,601	1.64	176,368,350	15.81	265,557,169	23.80
LINE	18,505,523	1.66	18,472,077	1.66	39,150,665	3.51	58,410,686	5.24
SINE	1,223,277	0.11	0	0	1,614,176	0.14	2,705,886	0.24
LTR	33,717,187	3.02	27,447,149	2.46	86,516,298	7.75	110,388,569	9.89
Other	12,944	0.0012	0	0	0	0	12,944	0.0012
Unknown	0	0	0	0	7,390,437	0.66	7,390,437	0.66
Total	186,984,973	16.76	64,209,925	5.76	272,292,311	24.41	381,302,121	34.18

Table S8

Repeat content and classification.

Classes	Copies	Length (bp)	Percent
**DNA transposons**			
DNA/hAT	503654	72551513	6.50
DNA/TcMar-Tc1	409528	66734234	5.98
DNA/CMC-EnSpm	253341	31275680	2.80
DNA/others	962050	148543268	8.51
**Retrotransposons**			
**LINE**			
LINE/L2	218550	35773866	3.21
LINE/L1	33759	7329355	0.66
LINE/others	99282	18482742	1.37
**LTR**			
LTR/Gypsy	202329	43870463	3.93
LTR/DIRS	138347	27002034	2.42
LTR/others	227354	46796126	3.54
**SINE**	18322	2839059	0.24
**Others**	174925	33675196	0.00
**Unknown**	21401	7390437	0.66

Table S9

Markers, genetic distance, genetic density, pseudochromosome length and number of scaffolds from the assembled *M. amblycephala* genome.

Linkage Group	Number of Markers	Genetic distance (cM)	Genetic density (cM)	Number of scaffolds	Length of pseudo-chromosomes (Kb)
**LG01**	288	61.93	0.22	149	53,058
**LG02**	283	97.21	0.34	73	49,405
**LG03**	322	94.33	0.29	54	49,778
**LG04**	240	93.42	0.39	42	44,463
**LG05**	273	81.74	0.300	103	31,264
**LG06**	264	63.22	0.24	45	38,987
**LG07**	258	101.07	0.39	53	44,791
**LG08**	254	77.25	0.30	37	32,199
**LG09**	250	70.57	0.28	83	29,826
**LG10**	242	52.00	0.22	32	33,886
**LG11**	238	65.70	0.28	60	26,356
**LG12**	230	64.99	0.28	37	35,382
**LG13**	220	72.22	0.33	47	31,477
**LG14**	214	78.73	0.37	67	34,595
**LG15**	204	61.61	0.30	67	24,897
**LG16**	200	67.59	0.34	102	25,704
**LG17**	199	87.13	0.44	62	35,978
**LG18**	186	60.08	0.32	43	29,345
**LG19**	160	68.53	0.43	46	29,749
**LG20**	155	62.19	0.40	70	19,324
**LG21**	130	62.73	0.48	43	17,255
**LG22**	140	48.47	0.35	53	21,731
**LG23**	98	53.95	0.55	15	22,271
**LG24**	89	54.62	0.61	51	17,820
**Total**	**5137**	**1701.24**	**0.33**	**1434**	**779,541**

Table S10

PSGs involved in glycometabolism (green) and lipid metabolism (red) in the *M. amblycephala* and *C. idellus* genome (FDR<0.05, *P*<=0.01).

M. amblycephala GeneID	*C. idellus* GeneID	Gene Symbol	Gene description	*P*-value	FDR
MamblycephalaGene11979	CI01000027_10580514_10590458	PIGV	GPI mannosyltransferase 2	4.07E-11	4.59E-09
MamblycephalaGene12793	CI01000321_04082880_04096700	SLC2A3	Solute carrier family 2, facilitated glucose transporter member 3	0.000659	0.010611
MamblycephalaGene10115	CI01000001_07558102_07564471	SLC2A5	Solute carrier family 2, facilitated glucose transporter member 5	1.26E-06	4.95E-05
MamblycephalaGene16736	CI01000026_01090877_01106876	Gba2	Non-lysosomal glucosylceramidase	1.10E-05	0.000329
MamblycephalaGene12749	CI01000037_00208728_00215104	Glb1	Beta-galactosidase	8.10E-05	0.001769
MamblycephalaGene11817	CI01000012_10639383_10640319	B3gat2	Galactosylgalactosylxylosylprotein 3-beta-glucuronosyltransferase 2	3.38E-14	8.42E-12
MamblycephalaGene09280	CI01000189_04657758_04673288	Acss1	Acetyl-coenzyme A synthetase 2-like, mitochondrial	4.20E-06	0.000148
MamblycephalaGene18414	CI01000340_12556528_12561142	H6pd	GDH/6PGL endoplasmic bifunctional protein	0.004096	0.04363
MamblycephalaGene09939	CI01000330_01043094_01051071	IdhA	L-lactate dehydrogenase A chain	3.57E-06	0.000128
MamblycephalaGene05371	CI01000127_02569419_02574322	Athl1	Acid trehalase-like protein 1	3.98E-05	0.000979
MamblycephalaGene22137	CI01000080_02113135_02116388	FabG	3-oxoacyl-[acyl-carrier-protein] reductase FabG	2.64E-07	1.21E-05
MamblycephalaGene08780	CI01000086_01838372_01840689	Fabp10a	Fatty acid-binding protein 10-A, liver basic	0.00309	0.035983
MamblycephalaGene21005	CI01000319_00143560_00150809	Cyp51A1	Lanosterol 14-alpha demethylase	0.004638	0.048083

MamblycephalaGene06395	CI01000274_00049106_00060472	Lpcat4	Lysophospholipid acyltransferase LPCAT4	0.001364	0.019058
MamblycephalaGene11566	CI01000353_00609948_00648017	Agps	Alkyldihydroxyacetonephosphate synthase, peroxisomal	9.24E-07	3.75E-05
MamblycephalaGene05387	CI01000306_01633237_01635512	Hrasls	HRAS-like suppressor	0.003983	0.042823
MamblycephalaGene07834	CI01000213_00050675_00058255	Ptpla	3-hydroxyacyl-CoA dehydratase 1	5.21E-06	0.000172
MamblycephalaGene14980	CI01000027_08713799_08734144	Dgat1	Diacylglycerol O-acyltransferase 1	4.59E-06	0.000159
MamblycephalaGene17154	CI01000340_19181273_19189266	SOAT2	Sterol O-acyltransferase 2	0.000388	0.006785
MamblycephalaGene02504	CI01000214_00365143_00371990	Srebf2	Sterol regulatory element-binding protein 2	0.002243	0.028288

Table S11

Significantly up-regulated genes in IB in the KEGG pathways involved in 'cell motility' and 'muscle constraction' showing in Additional file 1: Figure S13. M, Muscle; IB, Intermuscular bone; CT, Connective tissues.

GeneID	Symbol	Description	RPKM			log2 Ratio	
			M	IB	CT	IB/CT	IB/M
MamblycephalaGene13162	*Col6A*	collagen, type VI, alpha	0.826	4.742	0.952	2.316	2.521
MamblycephalaGene05619	*ECM2*	extracellular matrix protein 2	0.464	5.682	2.698	1.075	3.615
MamblycephalaGene10847	*Itga1*	integrin alpha 1	1.801	8.598	3.643	1.239	2.255
MamblycephalaGene15511	*Itga10*	integrin alpha 10	0.849	5.542	2.737	1.018	2.706
MamblycephalaGene05063	*Itgb1bp3*	integrin beta 1 binding protein	1.936	20.52	2.604	2.979	3.406
MamblycephalaGene11169	*Itga5*	integrin alpha 5	0.697	6.174	1.202	2.36	3.147
MamblycephalaGene15649	*PI3K*	phosphatidylinositol-4,5-bisphosphate 3-kinase	0.764	1.94	0.431	2.171	1.344
MamblycephalaGene00698	*Tiam*	T-cell lymphoma invasion and metastasis	0.404	1.273	0.304	2.065	1.654
MamblycephalaGene00373	*Rac1*	Ras-related C3 botulinum toxin substrate 1	1.059	4.949	1.962	1.335	2.225
MamblycephalaGene16958	*Actn*	actinin alpha	0.384	1.448	0.165	3.135	1.917
MamblycephalaGene09009	*Zyx*	zyxin	2.167	9.468	4.7	1.01	2.128
MamblycephalaGene13817	*act1*	actin, alpha skeletal muscle	9.113	264.2	60.89	2.118	4.858
MamblycephalaGene13011	*Tpm4*	tropomyosin 4	3.022	34.61	15.54	1.155	3.518
MamblycephalaGene18418	*TNNI2*	troponin I, fast skeletal muscle	0.18	4.195	0.305	3.782	4.541
MamblycephalaGene01716	*RhoGAP*	Rho GTPase-activating protein 4	1.692	6.739	3.251	1.052	1.994
MamblycephalaGene11597	*Rhoa*	Ras homolog gene family, member A	6.922	37.49	17.57	1.094	2.437
MamblycephalaGene19235	*Myo1*	myosin I	1.109	7.388	2.731	1.436	2.736
MamblycephalaGene06827	*Myo9*	myosin IX	1.833	10.83	0.065	2.584	2.563

MamblycephalaGene08455	*Myo9*	myosin IX	1.579	9.547	3.831	1.318	2.596
MamblycephalaGene13721	*MYH11*	myosin heavy chain	2.929	21.36	8.052	1.407	2.866
MamblycephalaGene22573	*MYH*	myosin heavy chain	41.08	156.1	47.48	1.717	1.926
MamblycephalaGene00898	*MLCK*	myosin-light-chain kinase	0.147	2.901	0.530	2.543	4.301
MamblycephalaGene19463	*CaM*	calmodulin	13.22	616.5	256.6	1.265	5.544
MamblycephalaGene16713	*Cacna1c*	voltage-dependent calcium channel L type alpha-1C	0.222	2.551	0.214	3.573	3.524

Table S12

Comparison of ORs in different teleosts and mammals.

	**Species**	**Water**				**Air**		**Air/Water**	**Non-OR**		**Total**
		Delta (δ)	Epsilon (ε)	Zeta (ζ)	Eta (η)	Alpha (α)	Gamma (γ)	Beta (β)	Kappa (κ)	Thet (θ)	
**Aquatic**	***M. amblycephala***	**56**	**8**	**47**	**47**	**0**	**3**	**16**	**1**	**1**	**179**
	C. idellus	62	11	24	20	0	1	14	1	1	134
	D. rerio	66	13	37	37	0	1	5	1	1	161
	G. morhua	55	2	13	14	0	0	1	0	1	86
	C. semilaevis	55	3	9	9	0	0	1	0	1	78
	F. rubripes	29	2	4	10	0	0	1	1	1	48
	O. latipes	31	2	9	18	0	0	2	1	0	63
	X. maculatus	38	4	4	12	0	0	1	1	1	61
**Terrestrial**	*B. taurus*	0	0	0	0	140	828	2	1	--	971
	H. sapiens	0	0	0	0	58	329	0	--	1	388

Table S13

Comparison of taste senses genes in fish species with different trophic level.

Feeding habits	Species	Umami		Sweet	Bitter	Sour	
		T1R1	T1R3	T1R2	T2Rs	HCN	ASIC
Herbivorous	***M. amblycephala***	**0**	**1**	**3**	**3**	**7**	**4**
	C. idellus	1	1	6	3	5	7
Omnivorous	*C. carpio*	1	2	12	5	10	21
	D. rerio	1	1	2	7	6	10
	O. latipes	4	1	3	1	7	9
	X. maculatus	5	1	0	1	7	12
Carnivorous	*G. morhua*	2	1	0	1	6	8
	C. semilaevis	2	1	0	0	7	8

Table S14

Comparison of amylases and proteases encoding genes in seven teleosts.

**Feeding Habits**	**Species**	**Amylase**		**Protease**			
		Alpha-amylase	Glucoamylase	Pepsin	Trypsin	Cathepsin	Chymotrypsin
Herbivorous	***M. amblycephala***	**2**	**2**	**0**	**38**	**22**	**12**
	C. idellus	2	5	0	53	31	12
Carnivorous	*C. semilaevis*	1	1	0	18	24	8
	G. morhua	1	1	3	17	17	12
Omnivorous	*D. rerio*	9	1	0	41	65	10
	O. latipes	1	1	0	27	19	7
	X. maculatus	2	1	0	20	23	4

Table S15

Sequences and alpha diversity indexes of the larvae (LBSB), domestic adult (DBSB), wild adult (BSB) *M. amblycephala* and wild adult *C. idellus* (GC) samples. Operational taxonomic units (OTUs) are defined at 97% sequence similarity.

Sample	Reads		Sequences		Alpha diversity		
	Raw reads	Valid	Qualified	Normalization	OTU	Chao1 estimated richness	Shannon diversity
LBSB1	65,992	59,924	64,582	54,430	479	830	4.03
LBSB2	94,866	93,704	93,176	86,422	408	646	4.01
LBSB3	30,306	23,507	23,337	21,512	865	1029	4.96
DBSB1	61,278	52,353	51,922	45,835	843	1246	5.31
DBSB2	31,901	25,640	24,889	21,499	1051	1334	7.00
DBSB3	88,510	76,203	75,662	65,905	485	733	4.22
BSB1	36,893	34,574	34,347	31,539	819	1166	5.43
BSB2	130,775	93,871	90,299	77,172	654	972	5.57
BSB3	59,924	38,672	36,502	31,415	1275	1410	8.47
GC1	31,150	30,076	29,834	25,193	818	1280	6.13
GC2	64,129	61,489	61,080	55,265	453	825	4.70
GC3	37,878	37,517	37,230	32,833	408	689	4.53

Table S16

The relative abundance (%) of gut microbial flora of the larvae (LBSB), domestic adult (DBSB), wild adult *M. amblycephala* (BSB) and wild adult *C. idellus* (GC) at the genus level (top 15).

Taxonomy	LBSB	DBSB	BSB	GC
Clostridium	2.362	3.014	4.883	3.611
Bacteroides	0.124	1.446	0.945	2.828
Xiphinematobacter	0.496	0.096	0.231	1.122
Cetobacterium	36.982	24.045	2.994	3.794
Leptotrichia	0.001	0.001	6.254	7.908
Shewanella	1.548	1.037	0.568	0.642
Citrobacter	0.002	0.991	1.421	1.045
Halomonas	1.596	2.059	0.845	0.732
u114	4.970	4.906	3.205	3.010
Streptococcus	0.013	1.104	0.495	2.723
Ruminococcus	0.021	1.145	0.247	0.080
Rhabdochlamydia	0.003	0.008	0.624	1.268
Sphingomonas	0.512	0.410	0.286	0.147
Acinetobacter	0.423	0.712	0.358	0.263
Nitrosopumilus	0.049	0.072	0.771	0.015

Table S17

Relative abundance (%) of potential cellulose-degrading bacteria in LBSB, DBSB, BSB and GC gut samples.

Taxonomy	LBSB	DBSB	BSB	GC
Clostridium	2.362	3.014	4.883	3.611
Citrobacter	0.002	0.991	1.421	1.045
Streptococcus	0.013	1.104	0.495	2.723
Ruminococcus	0.021	1.145	0.247	0.080
Brevundimonas	0.010	0.045	0.010	0.001
Paenibacillus	0.002	0.003	0.045	0.001
Pseudoxanthomonas	0.001	0.017	0.037	0.010
Pseudomonas	0.010	0.037	0.171	0.020
Methylobacterium	0.021	0.028	0.030	0.020
Actinomyces	0.006	0.010	0.020	0.010
