# Supplementary material for: The draft genome of blunt snout bream (Megalobrama amblycephala) reveals the development of intermuscular bone and adaptation to herbivorous diet
Source: Gigascience. 2017 May 23;6(7):1–13. doi: 10.1093/gigascience/gix039 (PMC5570040; doi:10.1093/gigascience/gix039)
Supplement: Response-to-Reviewer-Comments_Original-Submission.pdf [file gix039_Response-to-Reviewer-Comments_Original-Submission.pdf]

24 November 2016
Dr. Hans Zauner
Journal: GigaScience

Dear Dr. Zauner,
Manuscript No.: GIGA-D-16-00088
Title: "The draft genome of Megalobrama amblycephala reveals the development of intermuscular bone and adaptation to herbivorous diet"
Author(s): Han Liu, Chunhai Chen, Zexia Gao, Jiumeng Min, Yongming Gu, Jianbo Jian, Xiewu Jiang, Huimin Cai, Ingo Ebersberger, Meng Xu, Xinhui Zhang, Jianwei Chen, Wei Luo, Boxiang Chen, Junhui Chen, Hong Liu, Jiang Li, Ruifang Lai, Mingzhou Bai, Jin Wei, Shaokui Yi, Huanling Wang, Xiaojuan Cao, Xiaoyun Zhou, Yuhua Zhao, Kaijian Wei, Ruibin Yang, Bingnan Liu, Shancen Zhao, Xiaodong Fang, Manfred Schartl, Xueqiao Qian, Weimin Wang

We have carefully read the referees' comments which you forwarded to us with your email of 2 November 2016. We would like to express our sincere thanks to the reviewers for the constructive and positive comments. We have addressed all their suggestions, and the manuscript has been edited accordingly. Please find below our responses to the comments of the referees. The major amendments are highlighted in red in the revised manuscript. Responses to each of the reviewers' comments are detailed below in this letter. Because of the amendments, the page and the line numbers referred to by the referees have now changed in the edited version of the manuscript. Please note that all raw reads of genome sequencing and RAD-seq have been deposited at NCBI. Other data including assemblies, annotations, RNA-seq data, microbiome data and embedded image data (intermuscular bones and histological stainings) were uploaded to the indicated ftp. We hope that with the amendments made in response to the reviewers' comments, the manuscript is now acceptable for publication in GigaScience.
I look forward to hearing from you soon.

Yours sincerely,

Weimin Wang (PhD) (Correspondence author)
College of Fisheries
Huazhong Agricultural University
Wuhan 430070, P. R. China
E-mail address: wangwm@mail.hzau.edu.cn
Tel: +86-27-8728 4292; Fax: +86-27-8728 4292

Response to Reviewers
Reviewer Reports
Reviewer 1:
It is clear that a lot of work has gone into the creation of a draft genome for Megalobrama amblycephala. The creation of a genetic map to help anchor the assembly is significant and I am please to see it. Their results and discussion flow logically from the genome assembly and annotation as compared with other fish species. Although every bioinformatician has their favorite open source program, the software used in the construction of the assembly and annotation are well known and documented.
I recommend acceptance with the following comments and strong recommendations.
1) All raw data for the assembly and annotation be submitted to a public database.

Author response: The raw sequencing data have been deposited at NCBI under the accession number SRP090157 and raw data is available from the SRA under bioproject number PRJNA343584. We also have uploaded the assemblies, annotations, RNA-seq and microbiome data to the ftp (ftp://user28@climb.genomics.cn).

2) All raw data and markers for the genetic map be submitted to a public database or made readily downloadable.

Author response: All the raw data and markers for the genetic map have been deposited at NCBI in the SRA under bioproject number PRJNA343584 and biosample number SRS1797758.

3) The genome assembly be checked for contamination. This can be easily done with programs like blobtools and megablast. If contamination is found please remove it prior to submitting to the public databases and update the paper accordingly. In this way we do not contaminate and propagate contamination in our common public databases.

Author response: We have checked the genome assembly for contamination. The results are shown as bellow:
Scaffold_ID Start End Cover_Len Scaffold_Len Coverage (%) Tax_ID Organism
scaffold16634 1 779 756 958 78.91 1148 Synechocystis sp.
scaffold19397 129 583 455 908 50.11 111780 Stanieria cyanosphaera
scaffold11890 102 394 293 523 56.02 1287681 Eutypa lata
Only one gene MamblycephalaGene23156.1 is involved in the contamination region. We have now shielded them and updated the gene and genome data in the indicated database (ftp://user28@climb.genomics.cn).

4) Consider swapping out some of the language of "plays a role" with phrases like "are involved in" or "have reported to" or "have a role in" etc.;

Author response: We have replaced the expression of "plays a role" by "be involved in" or "be associated with" throughout the whole manuscript.

5) On Line 241, you have written bittern, I suspect you either meant bitter or bitterness.

Author response: This was a language error on the out side and it should read bitterness. We now have amended it in the revised manuscript.

6) I downloaded and examined the annotation file Megalobrama_amblycephala.gff. It only contains transcript models and no gene models. You state in the paper there were 23,696 protein coding genes. This number corresponds to the number of mRNA models you have in this gff file. This struck me as odd as there are usually isoforms for gene models. This file also did not contain any functional annotation. Please create a gff3 file that contains genes models and include functional annotation as this will be of great value to researchers.

Author response: We have created a gff3 file that contains genes models and functional annotations. This will be available in the GigaDB repository associated with the paper publication. The format of this file as shown below:

This is an important work for the development of sustainable aquaculture. Thank you.

Reviewer 2:
The draft genome of Megalobrama amblycephala reveals the development of intermuscular bone and adaptation to herbivorous diet by Liu H., et al. The present manuscript by Liu H., et al. can be divided into three main parts. The first part deals with the genome sequencing of one of the economically important

cyprinid fish species Megalobrama amblycephala. Authors have generated a draft genome of 1.116 GB and managed to link 779.54 Mb to 24 linkage groups. Linkage groups were also constructed in the present work. Authors further investigate comparative genomics and phylogenetic analysis using the generated data. The second part investigates the characteristics of the feeding strategy of Megalobrama amblycephala being an herbivore species.

1) This comprised analysis of “expanded gene families” (this expression is rather unfortunate and not clear. It does not show which gene families finally were looked at and supplementary material like figure S11 does not say much) as well as the gut microbial community, but no differential expression analysis (mRNA). In the method section the experimental set up and analysis of the gut microbial study is missing.

Author response: We have now clarified these questions according to the reviewer's suggestions. The expanded genes in the M. amblycephala and C. idellus lineage are now listed in the Additional file 2: Data Note1. The gut microbial community of the larvae (LBSB), domestic adult (DBSB), wild adult (BSB) M. amblycephala and wild adult C. idellus (GC) at phylum level and genus level are shown in Figure 4E and Additional file 1: Table S16, respectively. The sequences information and alpha diversity indexes are shown in Table S15 in the revised Additional file 1. We have added the experimental set up and analysis of the gut microbial study in the method section of the revised manuscript.

2) The third part studies the development of intermuscular bones comprising the analysis of “expanded gene families” (again figure 2B does not explain the “expanded gene families”) and transcriptome analysis of early developmental stages.

Author response: We apologize for the mistake. Reference should have been made to Figure 2C and not to Figure 2B. This has been corrected in the revised manuscript.

3) Authors present a huge amount of data and analysis but finally do describe and discuss only a little part of their analysis mainly in form of a very small set of genes. The manuscript is well, but not straightforward written. The structure has to be revised and the outcome better worked out.

Author response: We have revised our manuscript according to your suggestions and modified and renumbered the figures and tables clearly.

INTRODUCTION
1) LINE 64: Today many genomes (draft and nearly complete) are available. It is suggested to categorize them into fresh water, Mediterranean and Atlantic sea important aquacultured species. One species e.g. important for the Mediterranean aquaculture, The European sea bass (Dicentrarchus labrax) which was recently sequenced is not listed (European sea bass genome and its variation provide insights into adaptation to euryhalinity and speciation. Nature communications, 2014 5, 5770.)

Author response: This important species, the European sea bass, has been listed now in the revised manuscript (Line 65).

2) LINE 68-71: Please re-phrase the sentence. It is not true that the focus of aquaculture is focusing on herbivorous species. It is true however, that the usage of alternative feed is pursued but not the culture of herbivorous species. In contrast several projects are working on new species (including carnivores) for aquaculture purposes.

Author response: This sentence has been re-phrased as “Reports on draft genomes of some resource friendly herbivorous and omnivorous species, in particular cyprinid fish are scarce” in the revised manuscript

(Line 69-70).

3) LINE 79 and LINE 84-85: Please refer to Wan et al., 2016 "Dynamic mRNA and miRNA expression analysis in response to intermuscular bone development of blunt snout bream (Megalobrama amblycephala)", Scientific Reports.

Author response: We have added this research paper as a reference in the revised manuscript (Line 80-83 and Line 88-89).

DATA DESCRIPTION

1) Description of generated SNP linkage map appears here, but does not appear in the method section. Please accomplish the method section.

Author response: We have added such information in detail now in Data Description section (Line 128-133) and also added the description of the genetic map construction in the method section (Line 403-416) in the revised manuscript.

ANALYSES SECTION

1) According to GigaScience Authors guide: "This section should provide details of all of the experiments and analyses that are required to support the conclusions of the paper. The authors should make clear the goal of each analysis and state the basic findings". Information about analyses (except the linkage map analysis) is partially found in the section named "Results" as well as in the "Method" section.

Author response: We have now modified this section according to your suggestions. We stated the goal and main finding of each analysis in the Results section and also removed the reporting of findings from the Method section.

2) LINES147-153: It is not clear why the enrichment analyses shows that the adaptation to herbivory goes "hand in hand" with the coping with plant secondary metabolites. Do authors have a comparable analysis of a carnivore or omnivore teleosts?

Author response: This sentence was ambiguous and we apologize for the confusion that it generated. We have now changed this expression to "These genes encoding proteins involved in biodegradation of xenobiotics would enhance the ability of an herbivore to detoxify the secondary compounds present in grasses that are adverse or even toxic to the organism." (Line 161-164).

3) LINE 170: What do authors mean by the expression "comparative transcriptome analysis"?

Author response: This expression was not accurate. We have now modified the expression and write "differential genes expression (DGE) analysis" or "transcriptome analysis" at the appropriate positions of the manuscript.

4) LINE 174: "notable". This result is not really surprising. Many other transcriptome studies in teleost have also shown that at the later stages, where the larvae is mainly growing, mostly muscle genes are up-regulated when compared to earlier stages.

Author response: We agree. This word has been deleted in the revised manuscript.

5) LINE 183: change the expression “eventually extended”.

Author response: This expression has been changed to “we performed differential genes expression (DGE) analysis of” in the revised manuscript (Line 192).

6) LINE 203. The link to Figure 3D is not clear. Is Figure 3D showing all 35 identified genes?

Author response: Figure 3D shows several developmental signals regulating key steps of osteoblast and osteoclast differentiation in the process of intramembranous ossification. In Figure 3D, only the colored boxes indicate these 35 identified genes. To make this more clear, we have amended this sentence to “35 bone formation regulatory genes were identified (shown in colored boxes in Figure 3D)” (Line 212).

DISCUSSION
Mainly a repetition of the previous paragraphs.
1) LINE 134 and 271: Authors did not show that Dre10 and Dre22 are ‘ancestral’, just that those two chromosomes fused to one chromosome in blunt snout bream as this species has 24 chromosomes while zebrafish has 25 chromosomes. Please take into account publications like Nakatani, Y., H. Takeda, Y. Kohara, and S. Morishita, 2007 Reconstruction of the vertebrate ancestral genome reveals dynamic genome reorganization in early vertebrates. Genome Res. 17: 1254–1265 and. Hufton, A. L., D. Groth, M. Vingron, H. Lehrach, A. J. Poustka et al., 2008 Early vertebrate whole genome duplications were predated by a period of intense genome rearrangement. Genome Res. 18: 1582–1591.

Author response: According to the referee’s suggestion and taking into account the two references, we have modified this sentence to “The most prominent event is a chromosomal fusion in M. amblycephala LG02 that joined two D. rerio chromosomes, Dre10 and Dre22”.

2) LINE 311: ‘comparative transcriptome” This expression leads the reader to the false impression that more than one species was studied. However authors investigated here in differential expression.

Author response: The expression “comparative transcriptome” has been amended to “differential genes expression (DGE) analysis” or “transcriptome analysis” in the appropriate positions of the manuscript.

METHODS
1) Missing description of microbial community study as well as generation of linkage map.—

Author response: We have now added more detailed information in the Methods section including genome assembly, construction of gene families, the microbiota analysis, genetic map construction and other related information.
