# Supplementary material for: The draft genome of blunt snout bream (Megalobrama amblycephala) reveals the development of intermuscular bone and adaptation to herbivorous diet
Source: Gigascience. 2017 May 23;6(7):1–13. doi: 10.1093/gigascience/gix039 (PMC5570040; doi:10.1093/gigascience/gix039)
Supplement: Response-to-Reviewer-Comments_Revision-1.pdf [file gix039_Response-to-Reviewer-Comments_Revision-1.pdf]

16 March 2017
Dr. Hans Zauner
Journal: GigaScience

Dear Dr. Zauner,

Manuscript No.: GIGA-D-16-00088R1
Title: "The draft genome of Megalobrama amblycephala reveals the development of intermuscular bone and adaptation to herbivorous diet"
Author(s): Han Liu, Chunhai Chen, Zexia Gao, Jiumeng Min, Yongming Gu, Jianbo Jian, Xiewu Jiang, Huimin Cai, Ingo Ebersberger, Meng Xu, Xinhui Zhang, Jianwei Chen, Wei Luo, Boxiang Chen, Junhui Chen, Hong Liu, Jiang Li, Ruifang Lai, Mingzhou Bai, Jin Wei, Shaokui Yi, Huanling Wang, Xiaojuan Cao, Xiaoyun Zhou, Yuhua Zhao, Kaijian Wei, Ruibin Yang, Bingnan Liu, Shancen Zhao, Xiaodong Fang, Manfred Schartl, Xueqiao Qian, Weimin Wang

We have carefully read the referee's comments which you forwarded to us with your email of 20 January 2017. We would like to express our sincere thanks to the reviewer for the constructive comments. We have now addressed all the suggestions, and the manuscript has been edited accordingly. The major amendments are highlighted in red in the revised manuscript. Responses to the reviewer's comments are detailed below in this letter. Because of the amendments, the page and the line numbers referred to by the referee have now changed in the edited version of the manuscript. Please, note that all raw reads of genome sequencing and RAD-seq have been deposited at NCBI. Other data including assemblies, annotations, RNA-seq data, microbiome data, embedded image data and SNP markers were uploaded to the indicated ftp. We hope that with the amendments made in response to the reviewer's comments, the manuscript is now acceptable for publication in GigaScience.

I look forward to hearing from you soon.

Yours sincerely,
Weimin Wang (PhD) (Correspondence author)
College of Fisheries
Huazhong Agricultural University
Wuhan 430070, P. R. China
E-mail address: wangwm@mail.hzau.edu.cn
Tel: +86-27-8728 4292; Fax: +86-27-8728 4292

Response to Reviewer
Reviewer Report
Reviewer #2: It is quite demanding to properly review a paper with this amount of data and therefore the structure of the manuscript is very important. In addition, a figure showing the overview of the workflow would have been helpful.
Author response: We have now included a schematic figure to show the workflow that should be helpful

for a better understanding of the workflow behind the data presented in the supporting information as additional file 1: Figure S2. (Line 92)

Concerns of Reviewer 1 and 2 are partially answered and altered accordingly in the manuscript. However some important aspects have to be addressed prior to acceptance.
1. Database submission: All raw data and molecular markers have to be available. The submission to the NCBI SRA database is not sufficient as this database is for sequencing data. There are specific databases for molecular markers such as SNPs.
Author response: We have uploaded the assemblies, annotations, RNA-seq, microbiome data and molecular markers (SNPs) to the ftp (ftp://user28@climb.genomics.cn). The URL is included in the revised manuscript.

2. Still the structure has to be revised and the outcome better worked out. The result section comprises parts of the discussion and the discussion contains again descriptions of the results. Line 227 -228 is just one example.
Author response: We have now carefully revised the manuscript according to your suggestions. The parts of the discussion in the results section have been removed and we also refined the discussion section.

3. The reading of the manuscript is still tedious as no structure /concept of the work is given.
Author response: According to your suggestions, we have now carefully revised the manuscript. We hope it is now clear and structured for reading and understanding with these changes and the additional file 1: Figure S2 showing the workflow.

4. Description of the differential expression analysis remains poor. No numbers of significant expressed transcripts are given nor was any further meta-analysis performed. In addition the threshold for significance has not been provided. The sentence at line 502 "DEGs were detected using DESeq" is not informative enough.
Author response: We are sorry for the simple description about differential expression analysis. In the first submitted Supplementary Note, we had detailed the transcriptome analyses. Because there is no Supplementary Note section in the Journal, we deleted it and selectively added some necessary information in the Methods section of the manuscript. We have now clarified these questions in the new revised manuscript according to your suggestions. (Line 175-177, 184-193, 492-496)

5. Line 167 the word "many" in the results section is not an appropriate expression here.
Author response: This sentence has been modified. (Line 163-166)

6. Please provide revised Figure files.
Author response: The revised Figure files are now attached with this new revised manuscript.

7. Still authors do not explain what is meant by "expanded gene families". Figure 2C does not show "gene families" but GO categories. E.g myosin complex belongs to GO:0016459. A gene family comprises

similar genes, formed by duplication of a single original gene. Those genes normally have also similar biochemical functions. The most known example is the family comprising the human hemoglobin subunits.
Author response: We apologize for the confusion. Indeed, Figure 2C does not show expanded gene families but the over-represented GO annotations of cyprinid-specific expansion genes. This has been corrected now in the manuscript and the figure legends. (Line 169-170, 787)

8. What is meant by "resource friendly"? It is still not clear what the authors want to pinpoint to.
Author response: This sentence was ambiguous and we apologize for the confusion that it generated. This sentence has been re-phrased and reads now: “Reports on draft genomes of herbivorous and omnivorous species……” in the revised manuscript. (Line 69)

9. Line 83 change expression: "remain obscure"
Author response: This sentence has been re-phrased and reads now: “the molecular genetic basis and the evolution of this unique structure are still unclear”. (Line 82)

10. Line 88: "many miRNA-mRNA interactions" not really informative.
Author response: This sentence has been re-phrased and reads now: “1,136 miRNA-mRNA interaction pairs”. (Line 87)

11. Line 129" "psuedo-chromosomes". Most probably "pseudo-chromosomes" are meant. Please carefully proof read the manuscript for typos and inadequate expressions.
Author response: We apologize for the spelling mistake. It should be "pseudo-chromosomes". This has been corrected in the revised manuscript. (Line 129)
