# Supplementary material for: The draft genome of blunt snout bream (Megalobrama amblycephala) reveals the development of intermuscular bone and adaptation to herbivorous diet
Source: Gigascience. 2017 May 23;6(7):1–13. doi: 10.1093/gigascience/gix039 (PMC5570040; doi:10.1093/gigascience/gix039)
Supplement: Response-to-Reviewer-Comments_Revision-2.pdf [file gix039_Response-to-Reviewer-Comments_Revision-2.pdf]

2 May 2017
Dr. Hans Zauner
Journal: GigaScience

Dear Dr. Zauner,
Manuscript No.: GIGA-D-16-00088R2
Title: "The draft genome of blunt snout bream (Megalobrama amblycephala) reveals the development of intermuscular bone and adaptation to herbivorous diet"
Author(s): Han Liu, Chunhai Chen, Zexia Gao, Jiumeng Min, Yongming Gu, Jianbo Jian, Xiewu Jiang, Huimin Cai, Ingo Ebersberger, Meng Xu, Xinhui Zhang, Jianwei Chen, Wei Luo, Boxiang Chen, Junhui Chen, Hong Liu, Jiang Li, Ruifang Lai, Mingzhou Bai, Jin Wei, Shaokui Yi, Huanling Wang, Xiaojuan Cao, Xiaoyun Zhou, Yuhua Zhao, Kaijian Wei, Ruibin Yang, Bingnan Liu, Shancen Zhao, Xiaodong Fang, Manfred Schartl, Xueqiao Qian, Weimin Wang
We have carefully read the referee's comments which you forwarded to us of 27 April 2017. We would like to express our sincere thanks for the positive comments. We have addressed all the suggestions. The common name of the species has been included in the title (Line 1 to 2). The Fishbase number and image of an adult blunt snout bream have also been added in the revised manuscript (Line 73 to 74). The amendments are highlighted in red in the revised manuscript. Responses to the reviewer's comments are detailed below in this letter. We hope that with the amendments made in response to you and the reviewer's comments, the manuscript is now acceptable for publication in GigaScience.
I look forward to hearing from you soon.

Yours sincerely,
Weimin Wang (PhD) (Correspondence author)
College of Fisheries
Huazhong Agricultural University
Wuhan 430070, P. R. China
E-mail address: wangwm@mail.hzau.edu.cn
Tel: +86-27-8728 4292; Fax: +86-27-8728 4292

Response to Reviewer
Reviewer Report
Reviewer #2:
1. Have a look at the sentence at line 104-107: "to assess the genome assembly quality ….."
Author response: This sentence has been modified as "To assess the quality of genome assembly, the short-insert size paired-end libraries reads and published ESTs [14] (Additional file 1: Tables S3 and S4) were mapped onto the genome. The results indicated that the assembled error is low." (Line 105 to 107)

2. Line 151 "single –copy genes"?
Author response: This expression has been changed as "single-copy orthologous genes". (Line 152)

3. 153: "outgroup" not "out group"
Author response: This has been corrected.

4. Rephrase line 552.
Author response: The SRP accession number has been added in the revised manuscript. This sentence has been rephrased as "Raw whole genome sequencing and RAD-Seq data have been deposited at NCBI in the SRA under accession number SRP090157 (BioProject Number: PRJNA343584)". (Line 553 to 554)

5. 3 Additional file 2 Data note 1. Typo: "Expansion" instead of "Expasion"
Author response: This has been corrected.

6. Figure 2B not mentioned in the text.
Author response: We have now mentioned it in the text and expressed as "We found 9349 orthologous gene

families shared among five fish species. 246 are specific in the M. Amblycephala (Figure 3B)”. (Line 155 to 156)
