# Supplementary material for: The draft genome of blunt snout bream (Megalobrama amblycephala) reveals the development of intermuscular bone and adaptation to herbivorous diet
Source: Gigascience. 2017 May 23;6(7):1–13. doi: 10.1093/gigascience/gix039 (PMC5570040; doi:10.1093/gigascience/gix039)
Supplement: Reviewer-1-Report-(Original-Submission)-.pdf [file gix039_Reviewer-1-Report-(Original-Submission)-.pdf]

Reviewer Report

Title: "The draft genome of blunt snout bream (Megalobrama amblycephala) reveals the development of intermuscular bone and adaptation to herbivorous diet"

Version: Original Submission **Date:** 9/27/2016

Reviewer name: Andrew Severin

Reviewer Comments to Author:

It is clear that a lot of work has gone into the creation of a draft genome for Megalobrama amblycephala. The creation of a genetic map to help anchor the assembly is significant and I am please to see it. Their results and discussion flow logically from the genome assembly and annotation as compared with other fish species. Although every bioinformatician has their favorite open source program, the software used in the construction of the assembly and annotation are well known and documented.

I recommend acceptance with the following comments and strong recommendations.

1) All raw data for the assembly and annotation be submitted to a public database.

2) All raw data and markers for the genetic map be submitted to a public database or made readily downloadable.

3) The genome assembly be checked for contamination. This can be easily done with programs like blobtools and megablast. If contamination is found please remove it prior to submitting to the public databases and update the paper accordingly. In this way we do not contaminate and propagate contamination in our common public databases.

4) Consider swapping out some of the language of "plays a role" with phrases like "are involved in" or "have reported to" or "have a role in" etc.

5) On Line 241, you have written bittern, I suspect you either meant bitter or bitterness.

6) I downloaded and examined the annotation file Megalobrama_amblycephala.gff. It only contains transcript models and no gene models. You state in the paper there were 23,696 protein coding genes. This number corresponds to the number of mRNA models you have in this gff file. This struck me as odd as there are usually isoforms for gene models. This file also did not contain any functional annotation. Please create a gff3 file that contains genes models and include functional annotation as this will be of great value to researchers.

This is an important work for the development of sustainable aquaculture. Thank you.

Methods

Are the methods appropriate to the aims of the study, are they well described, and are necessary controls included? Yes

Conclusions

Are the conclusions adequately supported by the data shown? Yes

Reporting Standards

Does the manuscript adhere to the journal's guidelines on minimum standards of reporting? Yes

Statistics

Are you able to assess all statistics in the manuscript, including the appropriateness of statistical tests used? Yes, and I have assessed the statistics in my report.

Quality of Written English

Please indicate the quality of language in the manuscript: Needs some language corrections before being published

Declaration of Competing Interests

Please complete a declaration of competing interests, considering the following questions:

- Have you in the past five years received reimbursements, fees, funding, or salary from an organisation that may in any way gain or lose financially from the publication of this manuscript, either now or in the future?
- Do you hold any stocks or shares in an organisation that may in any way gain or lose financially from the publication of this manuscript, either now or in the future?
- Do you hold or are you currently applying for any patents relating to the content of the manuscript?
- Have you received reimbursements, fees, funding, or salary from an organization that holds or has applied for patents relating to the content of the manuscript?
- Do you have any other financial competing interests?
- Do you have any non-financial competing interests in relation to this paper?

If you can answer no to all of the above, write 'I declare that I have no competing interests' below. If your reply is yes to any, please give details below.

I declare that I have no competing interests

I agree to the open peer review policy of the journal. I understand that my name will be included on my report to the authors and, if the manuscript is accepted for publication, my named report including any attachments I upload will be posted on the website along with the authors' responses. I agree for my report to be made available under an Open Access Creative Commons CC-BY license (http://creativecommons.org/licenses/by/4.0/). I understand that any comments which I do not wish to be included in my named report can be included as confidential comments to the editors, which will not be published.

I agree to the open peer review policy of the journal

To further support our reviewers, we have joined with Publons, where you can gain additional credit to further highlight your hard work (see: https://publons.com/journal/530/gigascience). On publication of this paper, your review will be automatically added to Publons, you can then choose whether or not to claim your Publons credit. I understand this statement.

Yes
