# Supplementary material for: The draft genome of blunt snout bream (Megalobrama amblycephala) reveals the development of intermuscular bone and adaptation to herbivorous diet
Source: Gigascience. 2017 May 23;6(7):1–13. doi: 10.1093/gigascience/gix039 (PMC5570040; doi:10.1093/gigascience/gix039)
Supplement: Reviewer-2-Report-(Revision-1).pdf [file gix039_Reviewer-2-Report-(Revision-1).pdf]

Reviewer Report

Title: "The draft genome of blunt snout bream (Megalobrama amblycephala) reveals the development of intermuscular bone and adaptation to herbivorous diet"

Version: Revision 1 **Date:** 1/8/2017

Reviewer name: Elena Sarropoulou

Reviewer Comments to Author:

It is quite demanding to properly review a paper with this amount of data and therefore the structure of the manuscript is very important. In addition, a figure showing the overview of the workflow would have been helpful.

Concerns of Reviewer 1 and 2 are partially answered and altered accordingly in the manuscript. However some important aspects have to be addressed prior to acceptance.

1. Database submission: All raw data and molecular markers have to be available. The submission to the NCBI SRA database is not sufficient as this database is for sequencing data. There are specific databases for molecular markers such as SNPs.

2. Still the structure has to be revised and the outcome better worked out. The result section comprises parts of the discussion and the discussion contains again descriptions of the results. Line 227 -228 is just one example.

3. The reading of the manuscript is still tedious as no structure /concept of the work is given.

4. Description of the differential expression analysis remains poor. No numbers of significant expressed transcripts are given nor was any further meta-analysis performed. In addition the threshold for significance has not been provided. The sentence at line 502 "DEGs were detected using DESeq" is not informative enough.

5. Line 167 the word "many" in the results section is not an appropriate expression here.

6. Please provide revised Figure files.

7. Still authors do not explain what is meant by "expanded gene families". Figure 2C does not show "gene families" but GO categories. E.g myosin complex belongs to GO:0016459. A gene family comprises similar genes, formed by duplication of a single original gene. Those genes normally have also similar biochemical functions. The most known examples is the family comprising the human hemoglobin subunits.

8. What is meant by "resource friendly"? It is still not clear what the authors want to pinpoint to.

9. Line 83 change expression : "remain obscure"

10. Line 88: "many miRNA-mRNA interactions" not really informative.

11. Line 129" "psuedo-chromosomes". Most probably "pseudo-chromosomes" are meant. Please carefully proof read the manuscript for typos and inadequate expressions.

Methods

Are the methods appropriate to the aims of the study, are they well described, and are necessary controls included? Yes

Conclusions

Are the conclusions adequately supported by the data shown? No

Reporting Standards

Does the manuscript adhere to the journal's guidelines on minimum standards of reporting? Yes

Statistics

Are you able to assess all statistics in the manuscript, including the appropriateness of statistical tests used? No, and I do not feel adequately qualified to assess the statistics.

Quality of Written English

Please indicate the quality of language in the manuscript: Not suitable for publication unless extensively edited

Declaration of Competing Interests

Please complete a declaration of competing interests, considering the following questions:

- Have you in the past five years received reimbursements, fees, funding, or salary from an organisation that may in any way gain or lose financially from the publication of this manuscript, either now or in the future?
- Do you hold any stocks or shares in an organisation that may in any way gain or lose financially from the publication of this manuscript, either now or in the future?
- Do you hold or are you currently applying for any patents relating to the content of the manuscript?
- Have you received reimbursements, fees, funding, or salary from an organization that holds or has applied for patents relating to the content of the manuscript?
- Do you have any other financial competing interests?
- Do you have any non-financial competing interests in relation to this paper?

If you can answer no to all of the above, write 'I declare that I have no competing interests' below. If your reply is yes to any, please give details below.

‘I declare that I have no competing interests’ below.

I agree to the open peer review policy of the journal. I understand that my name will be included on my report to the authors and, if the manuscript is accepted for publication, my named report including any attachments I upload will be posted on the website along with the authors' responses. I agree for my report to be made available under an Open Access Creative Commons CC-BY license (http://creativecommons.org/licenses/by/4.0/). I understand that any comments which I do not wish to be included in my named report can be included as confidential comments to the editors, which will not be published.

I agree to the open peer review policy of the journal

To further support our reviewers, we have joined with Publons, where you can gain additional credit to further highlight your hard work (see: https://publons.com/journal/530/gigascience). On publication of this paper, your review will be automatically added to Publons, you can then choose whether or not to claim your Publons credit. I understand this statement.

Yes
